# Entropy-driven order-to-disorder transition in perovskite anodes for high-performance solid oxide fuel cells

**DOI:** 10.1038/s41467-026-74549-0

**Published:** 2026-06-19

**Authors:** Gaige Wang, Rongzheng Ren, Xiaodan Yu, Chunming Xu, Jinshuo Qiao, Wang Sun, Zhenhua Wang, Kening Sun

**Affiliations:** Beijing Key Laboratory of Green Hydrogen and Fuel Cells, School of Chemistry and Chemical Engineering, Beijing Institute of Technology, Beijing, China

**Keywords:** Fuel cells, Fuel cells, Fuel cells

## Abstract

Solid oxide fuel cells (SOFCs) enable direct and efficient conversion of transportable hydrocarbons into electricity, offering a scalable pathway for carbon-neutral energy systems. A critical challenge in SOFC development lies in the atomic-scale structural regulation of perovskite-type anodes, which is essential for enhancing hydrocarbon oxidation kinetics while mitigating carbon deposition issues. To overcome this fundamental limitation, we propose an entropy-driven strategy to induce order-to-disorder transitions in perovskite oxides. This strategy is demonstrated in the layered ordered perovskite PrBaFe_2_O_5+δ_, where the introduction of five equimolar rare-earth cations at the Pr site results in the formation of a disordered A-site high-entropy perovskite anode with the composition La_0.2_Pr_0.2_Sm_0.2_Gd_0.2_Y_0.2_BaFe_2_O_5+δ_ (HEP). Such atomic-scale order-to-disorder transitions facilitate oxygen vacancy formation and improve anode hydration capacity, thereby accelerating both hydrocarbon steam reforming and carbon elimination processes. The designed HEP anode exhibits a peak power density of 774.53 mW·cm^–2^ and stability over 1000 hours under wet methane (3 vol% H_2_O) at 700 °C. The present work contributes a new strategy for controlling ion ordering in perovskite oxides, addressing key challenges in SOFC operating with hydrocarbon fuels.

## Abstract (web summary)

Carbon deposition and insufficient reactivity hinder the development of anodes in solid oxide fuel cells. Here, authors propose an entropy-induced order–disorder transition strategy to fabricate high-entropy anodes to attain improved catalytic activity and durable coking resistance.

## Introduction

The global pursuit of carbon neutrality is accelerating the adoption of clean, efficient power generation technologies, among which fuel cell technology stands out for its exceptionally high energy conversion efficiency^1,2^. Currently, pure hydrogen serves as the predominant fuel for these cells, yet its exorbitant storage and transportation costs pose significant barriers to large-scale deployment^3^. Solid oxide fuel cells (SOFCs) emerge as a breakthrough solution, leveraging oxygen-ion-conducting oxide electrolytes to achieve remarkable fuel versatility^2,4,5^. Unlike conventional systems restricted to hydrogen, SOFCs can directly utilize readily available hydrocarbons like methane, whose stable storage and low-cost transport properties address the core limitations of hydrogen-based systems. This fundamental advantage enables SOFCs to unlock unprecedented technological potential for carbon-neutral energy applications^6^.

Despite their considerable potential for direct hydrocarbon fuel utilization, the development of anode materials with both high catalytic activity and long-term stability presents formidable technical challenges^7^. The anode-side reaction mechanisms are extremely complex, involving fuel reforming, cracking, and electrochemical processes^8,9^. These reactions can be categorized into two types based on reactant species. The first class is the water-involved reactions, such as the steam reforming and water-gas shift (Eqs. (1–4)). These reactions can convert methane into electrochemically active CO and H_2_, and positively contribute to cell performance. The another is the water-free reactions, including the hydrocarbon cracking and CO disproportionation (Eqs. (5, 6)). These reactions can generate non-reactive carbon deposits that are detrimental to SOFC performance^10^. Conventional Ni-based cermet anodes are prone to hydrocarbon fuel cracking and CO disproportionation, leading to carbon deposition that covers active sites and blocks anode pores, causing significant performance degradation^11^. To mitigate carbon deposition in Ni-based cermet anodes, various strategies have been employed to enhance the carbon resistance of anode, including incorporating alkaline oxides (e.g., BaO, CaO) on the Ni/cermet surface and elevating the water-to-carbon ratio in the anode chamber^7,12–15^. Unfortunately, these methods increase both the fabrication complexity of the cells and the system management challenges, thereby hindering the large-scale commercialization of SOFC technology^16^.

Water-involved reactions

1 $${{\rm{CH}}}_{4}({\rm{g}})+{{\rm{H}}}_{2}{\rm{O}}({\rm{g}})\to {\rm{CO}}({\rm{g}})+{3{\rm{H}}}_{2}({\rm{g}})$$

2 $${\rm{CO}}({\rm{g}})+{{\rm{H}}}_{2}{\rm{O}}({\rm{g}})\to {\rm{C}}{{\rm{O}}}_{2}({\rm{g}})+{{\rm{H}}}_{2}({\rm{g}})$$

3 $${{\rm{H}}}_{2}{\rm{O}}({\rm{g}})+{\rm{C}}({\rm{s}})\to {\rm{CO}}({\rm{g}})+{{\rm{H}}}_{2}({\rm{g}})$$

4 $${{\rm{CH}}}_{4}({\rm{g}})+{2{\rm{O}}}_{2}({\rm{g}})\to {{\rm{CO}}}_{2}({\rm{g}})+{2{\rm{H}}}_{2}({\rm{g}})$$

Water-free reactions

5 $${{\rm{CH}}}_{4}({\rm{g}})\to {\rm{C}}({\rm{s}})+{2{\rm{H}}}_{2}({\rm{g}})$$

6 $$2{\rm{CO}}({\rm{g}})\to {\rm{C}}({\rm{s}})+{{\rm{CO}}}_{2}({\rm{g}})$$

In the past few years, developing non-Ni alternatives such as perovskite oxides has emerged as a new direction for designing anode materials with both high activity and stability^17^. Due to the complexity of anode reactions, atomic-scale structural engineering of perovskite anodes is crucial for optimizing oxygen ion transport properties and surface characteristics of electrocatalysts, thereby enhancing hydrocarbon oxidation kinetics and eliminating detrimental carbon deposits‌^18–20^. The developmental trajectory of perovskite anodes has evolved from the initial ABO_3-δ_ single perovskite^21^, through the A_2_BB’O_6-δ_ double perovskite pioneered by Goodenough^22^, to the AA’B_2_O_5 + δ_ layered double perovskite proposed by Irvine^23^. These reported deliberate atomic-scale structural modulations have enabled significant breakthroughs in both electrochemical performance and coking resistance‌ of SOFC. Among these non-Ni oxides, PrBaFe_2_O_5 + δ_ (PBF) is a representative layered double perovskite anode material^24^, featuring a unique arrangement of Pr^3+^ and Ba^2+^ ions ordered along the c-axis at the A and A’ sites, respectively. This specific cationic ordering significantly impacts the electronic/ionic conductivity and the activity of catalyzing electrochemical oxidation of hydrocarbon fuels^17,25^.‌ Recent studies have highlighted that regulating cation ordering in layered double perovskites has emerged as a promising strategy to enhance their electrochemical performance, offering significant potential for further enhancing anode performance^26–28^. Nevertheless, exerting effective control over cation ordering at the atomic scale proves exceptionally difficult, owing to the considerable thermodynamic stability of the ordered phases^28–30^. To date, only limited studies have explored ion ordering modulation via A-site deficiency and B-site doping, with even fewer investigations extending these strategies to SOFC anode applications^26,27,31,32^. In addition‌, the underlying mechanism linking cation ordering to SOFC anode performance, particularly its impact on catalytic activity and carbon resistance, remains poorly understood. This knowledge gap significantly hinders the rational design of high-performance anode materials^30^.

Recently, high-entropy engineering has emerged as a powerful strategy for tailoring the atomic-scale structure and electrochemical properties of functional oxides, with its unique configurational entropy-driven structural modulation effect enabling the rational regulation of cation ordering and defect chemistry in perovskites^33,34^. Stimulated by the aforementioned advances, we herein present a novel (practical) entropy-driven strategy to induce the order-to-disorder transitions in layered double perovskite oxides, achieving atomic-level structural modulation of the perovskite oxide and significant enhancement in SOFC performance. We demonstrate this entropy-driven strategy by incorporating five rare-earth cations in equimolar proportions at the Pr site of PBF, resulting in an A-site high-entropy perovskite with the nominal composition La_0.2_Pr_0.2_Sm_0.2_Gd_0.2_Y_0.2_BaFe_2_O_5 + δ_ (see Supplementary Note 1 for material design rationale). Our findings reveal that high-entropy introduction disrupts the A-site ordered structure in layered perovskites, enabling oxygen vacancy formation to shift from the two-dimensional planes in ordered structures to the three-dimensional space‌ in disordered configurations. This structural transformation substantially boosts electrochemical oxidation activity for hydrocarbon fuels. Moreover, the order-to-disorder transition enhances the hydration capacity of the anode, accelerating both steam reforming of hydrocarbon fuels and carbon elimination processes, thereby conferring enhanced coking resistance. The designed HEP anode achieves a peak power density of 774.53 mW·cm^–2^ and stability over 1000 h in wet methane (3% H_2_O). The present work establishes a fundamental paradigm for manipulating ion ordering via entropy engineering, offering transformative solutions to simultaneously enhance catalytic activity and coking resistance in SOFC anodes.

## Results

### Phase structure and ion ordering

**Fig. 1:**
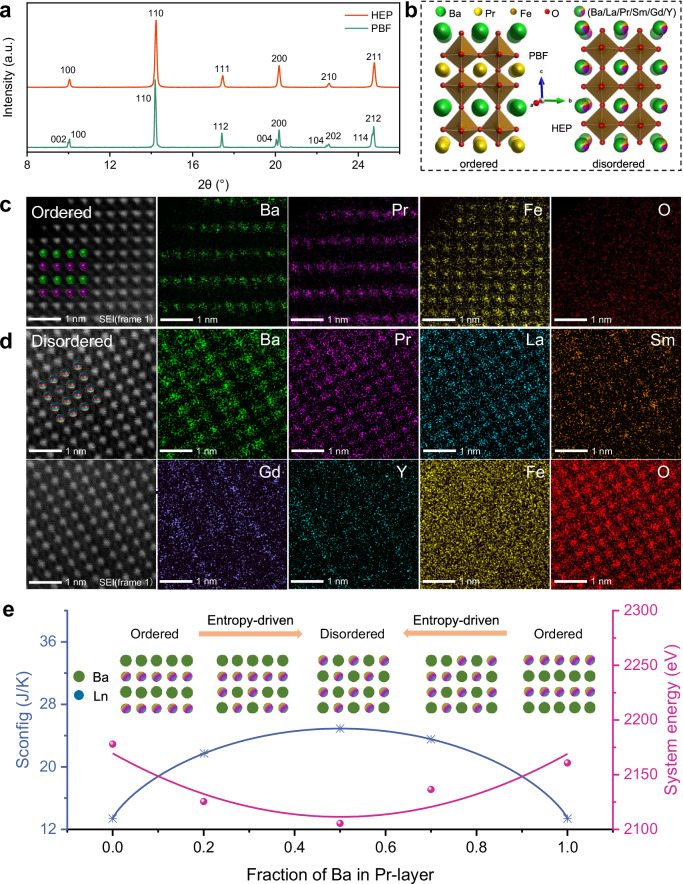
Crystalline structure and ion distribution of HEP and PBF. **a** SXRPD patterns of HEP and PBF. **b** Crystal structure models for PBF and HEP. HAADF-STEM elemental maps of **c** PBF and **d** HEP. **e** Entropy and system energy values as a function of fraction of Ba in Pr-layer. Source data for Fig. 1 are provided as a Source Data file.

The crystallographic phase and cationic ordering in PBF were characterized using synchrotron X-ray powder diffraction (SXRPD). As shown in Fig. 1a, the presence of split diffraction peaks at key Bragg positions, such as (100) and (200), indicates a tetragonal phase. This symmetry reduction is attributed to the ordered arrangement of Pr and Ba cations, confirming the formation of a layered perovskite structure^24^. This tetragonal structure was further corroborated by XRD patterns (Fig. S1a), which matched well with the tetragonal layered perovskite oxide (JCPDS, no. 00-057-0089). Rietveld refinement of the SXRPD data (Fig. S2a, Table S1), performed in the space group P4/mmm, yielded lattice parameters of a = b = 3.9327 Å and c = 7.9154 Å. As illustrated by the refined structural model (Fig. 1b), FeO_6_ octahedra are interconnected via corner-sharing to form a continuous network, while Pr and Ba cations are arranged in an alternating ordered manner along the c-axis. Energy-dispersive X-ray spectroscopy (EDS) elemental mapping and aberration-corrected high-angle annular dark-field scanning transmission electron microscopy (HAADF-STEM) observations unambiguously verified the formation of this well-ordered double perovskite structure (Figs. 1c, S3a).

The high-entropy perovskite was synthesized via the sol-gel combustion method. Transmission Electron Microscopy-EDS elemental mappings confirm that all constituent elements are homogeneously distributed throughout the material (Fig. S4). The stoichiometry of the resulting HEP was further determined by inductively coupled plasma mass spectrometry, with the measured composition showing consistent agreement with the nominal stoichiometry (Table S2). SXRPD patterns (Fig. 1a) reveal the merging of characteristic double peaks into single peaks, indicative of enhanced structural symmetry^24,35^. This merging reflects the loss of cationic order in PBF, leading to the transformation of HEP into a single-phase cubic perovskite (space group ${\mathrm{Pm}}\bar{3}\mathrm{m}$), as further confirmed by XRD and the SXRPD refinement results (Figs. S1b, S2b and Table S3). The highly symmetric and homogeneous crystal structure of HEP is directly evidenced by HAADF-STEM imaging (Fig. S3b), which reveals uniform lattice fringes with interplanar spacings of about 0.393 nm along both the (001) and (100) planes, consistent with a cubic perovskite structure. Moreover, HAADF-STEM elemental maps verify that all rare-earth elements (La, Pr, Sm, Gd, Y) and Ba are randomly distributed throughout the lattice, devoid of any layered ordering (Fig. 1d). These results demonstrate the successful order-to-disorder transition from the PBF to the HEP at the atomic scale.

In fact, our original design anticipated complete incorporation of five rare-earth cations into the entire Pr layer, thereby forming an ordered structure where rare-earth layers alternate with Ba layers^36,37^. However, experimental results demonstrate that these cations are found to be completely intermixed with Ba in HEP, ultimately forming a fully disordered cationic arrangement. This intriguing order-to-disorder transition prompts us to investigate the underlying mechanisms driving such structural disorder^38^. Regarding the ionic arrangement, the potential incorporation of Ba^2^⁺ ions into the Pr-layer could lead to a structural configuration of [(La_0.2_Pr_0.2_Sm_0.2_Gd_0.2_Y_0.2_)_1–*x*_Ba_*x*_][Ba_1–*x*_(La_0.2_Pr_0.2_Sm_0.2_Gd_0.2_Y_0.2_)_*x*_]Fe_2_O_5 + δ_, where the first square bracket represents the elemental composition of the Pr-layer, the second square bracket corresponds to the Ba-layer composition, and *x* denotes the fraction of Ba^2+^ substitution for rare-earth ions. This ionic arrangement was adopted primarily to simplify the mechanistic modeling framework, while also being consistent with experimental observations demonstrating the uniform distribution of all five rare-earth ions within the crystal lattice. Based on these considerations, several representative structure models with varying Ba^2+^ substitution in the Pr-layer (*x* = 0, 0.2, 0.5, 0.7, 1) have been constructed (Fig. S5).

The system energy of these established models was calculated by density functional theory (DFT) to evaluate the most stable structure model. Alternatively‌, in high-entropy oxides, ‌configurational entropy‌ (*S*_config_) is conventionally utilized as a quantitative measure to evaluate the degree of elemental disorder within the crystal lattice. An increased configurational entropy means the thermodynamic tendency toward single-phase formation becomes more pronounced^29,33^. Consequently, the configurational entropy‌ of these structural models in Fig. S5 was also systematically calculated to identify the optimal structural model‌. Based on our calculations, the variation of configuration entropy with *x* is determined to follow the relation:

7 $${S}_{{\rm{config}}}={\rm{\hbox{-}}}2R[(1{\rm{\hbox{-}}}x)\mathrm{ln}(1{\rm{\hbox{-}}}x)+{xlnx}]+R\mathrm{ln}5$$

In which *R* is the universal gas constant. Figure 1e illustrates the correlation between entropy and system energy across these structure models. The configurational entropy initially increases with *x* and subsequently decreases, peaking at *x* = 0.5 where cationic disorder reaches its maximum. Notably, the system energy first decreases and then increases with *x*, reaching a minimum around *x* = 0.5. In other words, the system energy decreases with increasing configurational entropy, indicating enhanced thermodynamic stability in more disordered states. These results confirm that configurational entropy serves as the key driving force behind the order-to-disorder transition in HEP. Therefore, when introducing five rare-earth cations, these cations and the Ba layers tend to mix and arrange to maximize configurational entropy, thereby minimizing the energy of the whole system and forming the most stable disordered structure.

To further understand the relationship between configurational entropy and order-to-disorder transitions, the number of A-site elements was reduced to lower the configurational entropy of the perovskite. A series of A-site binary, ternary, and quaternary rare-earth low/medium-entropy perovskite derivatives were synthesized and investigated, including La_0.5_Pr_0.5_BaFe_2_O_5 + δ_ (LPBF, *S*_config_ = 0.69 *R*), La_0.33_Pr_0.33_Sm_0.33_BaFe_2_O_5 + δ_ (LPSBF, *S*_config_ = 1.10 *R*), La_0.25_Pr_0.25_Sm_0.25_Gd_0.25_BaFe_2_O_5 + δ_ (LPSGBF, *S*_config_ = 1.39 *R*). XRD characterizations (Fig. S6a–c) confirm that all these samples adopt a pure single-phase perovskite structure. HAADF-STEM (Fig. S6d–f) and EDS elemental mappings (Figs. S7–9) reveal a clear entropy-dependent structural evolution. Binary LPBF and ternary LPSBF with lower entropy retain the typical layered ordering of the parent PBF, whereas the high-entropy quaternary LPSGBF exhibits a disordered structure. These results confirm that configurational entropy acts as the key driving force regulating the A-site ordering in layered double perovskites.

In addition, to verify the generality of the entropy-driven strategy, we extended the high-entropy design to A-site layered perovskite materials PrBaCo_2_O_5 + δ_ (PBC) and PrBaMn_2_O_5 +δ_ (PBM), synthesizing the high-entropy compositions La_0.2_Pr_0.2_Sm_0.2_Gd_0.2_Y_0.2_BaCo_2_O_5 + δ_ (LPSGYBC) and La_0.2_Pr_0.2_Sm_0.2_Gd_0.2_Y_0.2_BaMn_2_O_5 + δ_ (LPSGYBM). As shown in the XRD patterns (Fig. S10), the characteristic split peaks of layered PBC merged and vanished in LPSGYBC, resulting in a single perovskite phase. Similarly, a structural transition from the layered phase of PBM to a disordered mixed phase (cubic/hexagonal) was observed in LPSGYBM (Fig. S13)^23^. Furthermore, HAADF-STEM images (Figs. S11, S14) revealed alternating bright and dark contrasts indicative of a layered structure in PBC and PBM, which disappeared in their high-entropy counterparts. EDS elemental mapping further confirmed the cationic disorder in LPSGYBC and LPSGYBM (Figs. S12, S15). These results collectively demonstrate that the entropy-driven strategy is also applicable to other layered perovskite systems.

The operation of SOFC anodes under high-temperature reducing atmospheres demands exceptional structural stability from the anode materials^39^. To evaluate this property in the developed disordered HEP anode, its structural evolution was examined in a methane atmosphere. XRD analysis (Fig. S16) confirms that the HEP maintains its structural integrity throughout the entire testing period. In contrast, the reference material PBF undergoes rapid phase decomposition, forming secondary phases after only 40 h. These results provide strong evidence that the disordered HEP possesses enhanced structural stability under high-temperature methane atmospheres.

### Oxygen vacancy content and hydration capacity

**Fig. 2:**
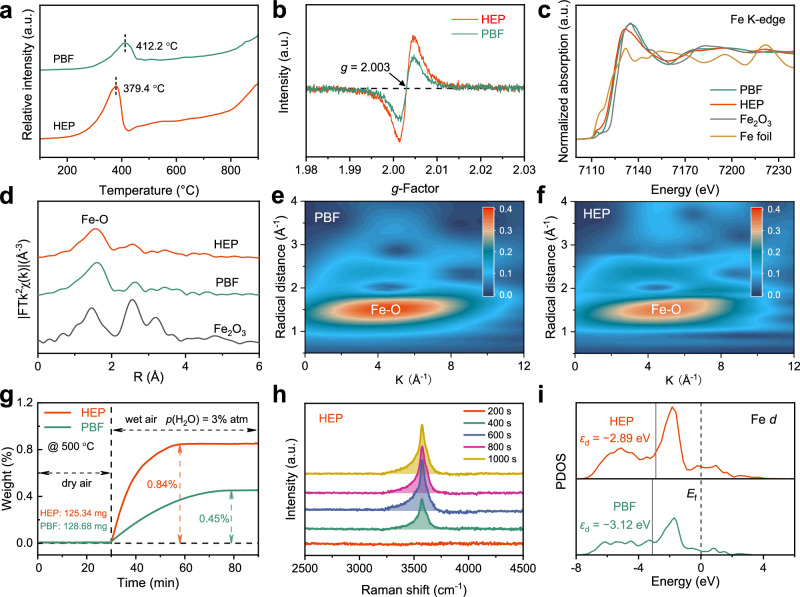
Oxygen vacancy content and hydration capacity. **a** H_2_-TPR spectra and **b** EPR plots of PBF and HEP, respectively. **c** Normalized Fe K-edge XANES spectra of PBF and HEP. **d** Fourier transformed EXAFS spectra of Fe K-edge for PBF, HEP, and Fe_2_O_3_. Wavelet transforms of the k^3^-weighted Fe K-edge EXAFS spectra of **e** PBF and **f** HEP. **g** Thermogravimetric relaxation curves upon changing *p*H_2_O from dry states to *p*H_2_O = 3% atm at 500 °C. The flow rate is 30 ml min^−1^. **h** Time-resolved Raman analysis of surface functional groups on HEP, during the exposure to wet N_2_ (3% H_2_O) at 500 °C (flow rate of N_2_: 50 ml min^−1^). **i** Corresponding PDOS band centers of Fe 3 d orbitals from PBF and HEP. Source data for Fig. 2 are provided as a Source Data file.

To elucidate the influence of ionic disorder on oxygen vacancy concentration in HEP, H_2_ temperature-programmed reduction (H_2_-TPR) analysis was performed. As presented in Fig. 2a, the H_2_-TPR spectra of HEP exhibit a high-temperature H_2_ consumption peak, which corresponds to the stepwise reduction of Fe^4+^/Fe^3+^ to Fe^2+^^40^. Notably, compared with pristine PBF, the H_2_ consumption peak of HEP shifts significantly toward lower temperatures, indicating that the lattice oxygen in HEP is more easily consumed. This observation further confirms that the disordered structure weakens the Fe–O bonds, thereby facilitating the generation of oxygen vacancies in the HEP lattice. This increase in oxygen vacancy defects was further corroborated by electron paramagnetic resonance (EPR) and thermogravimetric analysis (TGA)^41^. As illustrated in Fig. 2b, the HEP specimen exhibits a significantly more intense EPR signal than the PBF sample, indicating a higher concentration of oxygen vacancies. Consistent with this, TGA results (Fig. S17) reveal a weight loss of 1.14% for HEP, substantially exceeding that of PBF (0.62%), implying that ionic disorder enhances the density of oxygen vacancy defects at elevated temperatures^42^. To further understand the variation in oxygen vacancy concentration, we computed the Fe–O bonding energy using DFT (Fig. S18). The results reveal lower bond energies in HEP (6.34 eV) than in PBF (7.19 eV), indicating that ionic disorder weakens the Fe–O bond. This reduction directly correlates with facilitated oxygen vacancy formation^43^. To further verify the influence of cation ordering on the Fe–O bond energy, we constructed an ordered high-entropy perovskite model (computationally designed ordered HEP) with the same unit-cell parameters as the experimentally disordered HEP, as illustrated in Fig. S18. The calculated Fe–O bond energy for computationally designed ordered-HEP is 6.96 eV, which is significantly higher than that of experimentally disordered HEP (6.34 eV). This directly demonstrates that the order-to-disorder transition can promote the formation of oxygen vacancies.

To probe the local coordination of iron, we performed X-ray absorption spectroscopy (XAS). The normalized Fe K-edge XANES spectra (Fig. 2c) show that HEP exhibits a shift of the absorption edge to lower energy and a reduced white-line intensity relative to PBF^44,45^. These changes collectively indicate a lower Fe oxidation state in the cation-disordered HEP, which is consistent with the reduction in the average Fe valence state indicated by XPS (Fig. S19)^46^. The local coordination environment of Fe was further investigated by Fe K-edge EXAFS spectroscopy (Fig. 2d). The dominant peak near 1.5 Å, attributed to the Fe–O scattering paths, shows a lower intensity in HEP than in PBF, suggesting a reduced oxygen coordination number around Fe (Table S4)^47,48^. This finding is corroborated by wavelet transform (WT) analysis of the EXAFS oscillations (Fig. 2e, f), where the lower maximum intensity in HEP further confirms the decreased Fe–O coordination number resulting from the structure transition of order-to-disorder^45,49^.

Previous studies have demonstrated that oxygen vacancies in layered PBF perovskites are predominantly localized in the Pr layer due to its lower coordination number‌^23,50^. This leads to oxygen-ion transport channels being largely restricted to two-dimensional planes in ordered structures‌. Moreover, ordered perovskites tend to trap vacancies in energetically favorable yet spatially confined sites, which significantly obstructs ion migration pathways‌^51^. In contrast, when five different rare-earth cations are introduced at the Pr site, this ordered structure is disrupted in HEP, enabling oxygen vacancies to distribute uniformly across three-dimensional space‌, thus reducing vacancy trapping and enhancing interconnected diffusion pathways^26^. Therefore, disordered HEP is expected to exhibit a fast oxygen-ion diffusion coefficient. To validate this, electrical conductivity relaxation (ECR) measurements were employed to determine the bulk oxygen-ion diffusion coefficient of HEP (Fig. S20). The results show that the oxygen-ion bulk diffusion coefficient (*D*_chem_) increases from 6.51 × 10^–6^ cm^2^ s^–1^ for PBF to 3.51 × 10^–5^ cm^2^ s^–1^ for HEP at 700 °C, reflecting the inherently fast oxygen-ion diffusion nature in the disordered HEP^52^.

Previous studies have demonstrated that the surface hydration properties of anodes play a critical role in methane reforming reactions and carbon elimination processes^7,53,54^. To further elucidate the interaction between the HEP anode and water, the hydration behaviors of ordered PBF and disordered HEP perovskites were comparatively analyzed using thermogravimetric (TG) relaxation techniques. As shown in Fig. 2g, a sudden increase in water vapor partial pressure (*p*H_2_O) under constant *p*O_2_ induced rapid mass gains in both materials due to surface hydration. The more pronounced mass increase in HEP suggests a greater extent of water adsorption compared to PBF. To further visualize the hydration kinetics, in situ Raman spectroscopy was carried out on both materials. Under a wet gas mixture (3% H_2_O, 97% N_2_) at 500 °C, a distinct −OH band at 3580 cm^–1^ was observed for HEP within 400 s, whereas a detectable hydroxyl signal was not observed in PBF until 800 s (Figs. 2h, S21)^54^. This distinct kinetic behavior confirms that water dissociation and hydroxyl group formation occur more rapidly on the HEP surface, validating its enhanced water adsorption and activation capacity.

EPR was further employed to identify the adsorption sites of water molecules (Fig. S22). The significant decrease in the EPR signal intensity of HEP after hydration indicates the consumption of oxygen vacancies during the hydration reaction. This finding suggests that the hydration process takes place at the surface oxygen vacancy sites. The high concentration of oxygen vacancies in HEP thus explains its enhanced hydration capacity. To further investigate the origin of the enhanced hydration performance in HEP, DFT calculations were performed to probe the electronic structure of Fe sites. As shown in the projected density of states (PDOS) in Fig. 2i, the Fe *d*-band center (*ε*_d_) in HEP (–2.89 eV) is upshifted relative to that in PBF (–3.12 eV). This electronic configuration, being closer to the Fermi level (*E*_f_), renders the Fe sites in a more electron-deficient state, thereby enhancing their adsorption capacity^48^. The calculated dissociative adsorption energies of water further support this, showing a more negative value for HEP than for PBF (Fig. S23), which aligns perfectly with its enhanced hydration performance.

Based on the above analysis, the enhanced hydration capability of HEP can be attributed to the following two factors. First, the order-to-disorder transition promotes the generation of a higher concentration of oxygen vacancies, which provide more active adsorption sites for water molecules. Second, the order-to-disorder transition modifies the coordination environment of Fe and induces an upshift of the Fe d-band center toward the Fermi level, rendering Fe sites more electron-deficient and thus strengthening their interaction with the electron-donating oxygen atoms in water. Accordingly, the synergistic effect of increased oxygen vacancy concentration, favorable local coordination, and optimized electronic structure collectively contributes to the enhanced hydration reactivity of disordered HEP relative to ordered PBF.

### Electrochemical performance of HEP-anode SOFC

To elucidate the influence of structural disorder on the electrochemical performance of anode materials, electrochemical impedance spectroscopy (EIS) was first employed to characterize HEP and PBF. EIS measurements were carried out on SSZ (Sc_0.1_Zr_0.9_O_1.95_) electrolyte-supported symmetrical cells under realistic operating conditions (3% H_2_O-CH_4_ atmosphere) at various temperatures. This fuel composition was chosen mainly because it is readily accessible and easy to control, as the saturated vapor pressure of water at 25 °C (room temperature) is close to 3%. As presented in Fig. S24a, the HEP anode shows consistently lower polarization resistance (*R*_p_) than the PBF anode over the entire temperature range, demonstrating the promising potential of disordered HEP as a highly active anode material. A detailed performance comparison is provided in Fig. S24b. Notably, for the two anodes prepared under similar conditions with comparable specific surface areas (Fig. S25), the observed differences in electrochemical properties can be attributed to the intrinsic nature of the samples themselves.

To further understand the effect of the order‑to‑disorder transition on electrochemical performance, we also compared the EIS spectra of perovskites with different entropy values (LPBF, LPSBF, and LPSGBF). As shown in Fig. S26, the results reveal that disordered HEP exhibits the lowest *R*_p_ values. This indicates that anodes with a disordered structure possess enhanced electrochemical properties.

**Fig. 3:**
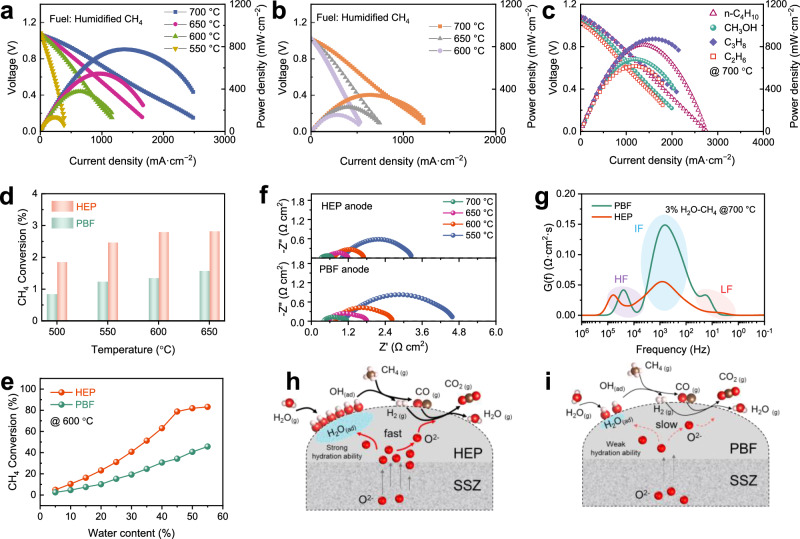
Electrochemical performance of HEP-Anode SOFC. I − V − P curves of single cells using **a** HEP and **b** PBF as the anodes in humidified methane (3 vol% H_2_O), at a methane flow rate of 20 ml min^–1^. **c** I-V-P curves of SOFCs using HEP as the anode operated on different fuels. **d** Comparison of methane reforming activity of HEP and PBF. The flow rate of humidified methane is 10 ml min^–1^. **e** Comparison of methane conversion rates under different water contents at 600 °C. **f** Electrochemical impedance of HEP and PBF single cells in 3% H_2_O-CH_4_ (flow rate of CH_4_: 20 ml min^−1^). **g** Distribution of relaxation time (DRT) analyses of EIS tested at 700 °C. Schematic illustration of the steam reforming mechanism for **h** HEP and **i** PBF. Brown, red and white spheres represent carbon, oxygen and hydrogen atoms, respectively. Source data for Fig. 3 are provided as a Source Data file.

The output performance of the disordered HEP anode was evaluated in an electrolyte-supported SOFC with a dense SSZ electrolyte layer (~300 μm) and a porous LSCF (La_0.6_Sr_0.4_Co_0.2_Fe_0.8_O_3-δ_) cathode (Fig. S27). A thin GDC interlayer was applied between the cathode/anode and the electrolyte to prevent phase reactions (Fig. S28). Figure 3a presents the performance characteristics of the HEP-Anode SOFC system operating with humidified methane (3 vol% H_2_O). The cell demonstrates notable power output across the tested temperature range, achieving peak power densities of 774.53, 545.14, 377.77, and 136.19 mW·cm^–2^ at 700, 650, 600, and 550 °C, respectively. In contrast, the power density of the ordered PBF-based anode under identical cell configuration (Fig. S29) and fuel conditions exhibits significantly inferior performance, with power outputs of 345.89, 230.10 and 157.20 mW·cm^–2^ at 700, 650 and 600 °C, respectively (Fig. 3b). This substantial performance disparity at all operational temperatures clearly highlights the enhanced catalytic activity of the disordered HEP anode toward methane oxidation. Compared to the anodes with different *S*_config_ (LPBF, LPSBF, and LPSGBF) and the most recent advancements in the field (Fig. S30, Table S5), our disordered HEP anode exhibits significantly competitive power density performance when benchmarked against both single-phase and ordered double perovskite oxide systems.

To further validate the catalytic performance of the disordered HEP anode for hydrocarbon fuels, the output characteristics of four fuels, n-butane, methanol, propane, and ethane, were systematically evaluated on the HEP-anode SOFC. All tests were conducted under near-identical steam-to-carbon ratios to ensure consistent reforming conditions and facilitate direct comparison of catalytic activity across these structurally diverse hydrocarbons. Peak power densities at 700 °C range from 872.73 mW cm^–2^ for propane to 615.2 mW cm^–2^ for ethane (Figs. 3c, S31). These results highlight the potential of the HEP anode for efficient hydrocarbon fuel utilization in SOFC systems. Figures S32, S33 present the EIS spectra of symmetrical cells collected under various fuel atmospheres. The results demonstrate that the HEP anode maintains a consistently lower *R*_p_ than the PBF anode under all tested conditions, further validating its enhanced intrinsic electrochemical activity and favorable fuel adaptability.

To gain a deeper understanding of the enhanced electrocatalytic capability of HEP anodes toward hydrocarbon fuels, the influence of HEP materials on methane conversion efficiency was investigated in a fixed-bed reactor during methane steam reforming (MSR), a pivotal reaction that significantly impacts the performance of SOFC anodes^45,55^. As depicted in Fig. 3d, the methane conversion rates for both PBF and HEP exhibit a positive temperature dependence under a fixed methane-to-steam ratio of 97:3 (vol%). HEP exhibits substantially higher conversion efficiencies than PBF across all tested temperatures, indicating that HEP possesses enhanced catalytic activity. The observed conversion plateau above 600 °C further underscores the system’s water content limitation (3 vol%), which governs the reaction thermodynamics at elevated temperatures. A particularly noteworthy observation is the pronounced water vapor concentration dependence of methane conversion on HEP. As demonstrated in Fig. 3e, when the temperature is held constant at 600 °C, both PBF and HEP exhibit an increase in methane conversion with rising water vapor pressure, indicating that hydration plays a promoting role in methane steam reforming. Due to its enhanced hydration capability, disordered HEP can effectively catalyze methane into CO and H_2_ (Fig. S34), thereby showing significantly higher methane conversion efficiency compared to PBF^56^. This finding elucidates the improved power density of the HEP anode.

In addition, for hydrocarbon-fueled SOFC anodes, the primary electrochemical reactions of CO + O^2–^ → CO_2_ + 2e^–^ and H_2_ + O^2–^ → H_2_O + 2e^–^ occurring on the anode surface also play a pivotal role in determining the power output of SOFC^20,38^. Notably, the disordered HEP exhibits accelerated oxygen-ion mobility, which further enhances anode performance. To substantiate this, we performed EIS and distribution of relaxation times (DRT) analysis on the full cells under operating conditions. The impedance analysis (Fig. 3f) demonstrates that the *R*_p_ of the cell with HEP as the anode is significantly lower than that of the cell using pristine PBF under identical conditions, further verifying the notable electrochemical performance of the HEP anode. The intrinsic origin of the reduced *R*_p_ for HEP was further elucidated by DRT analysis of the EIS data. As shown in Fig. 3g, each DRT profile displays three well-resolved peaks associated with gas diffusion, ion surface exchange, and interfacial charge transfer in the low-frequency (LF), intermediate-frequency (IF), and high-frequency (HF) regions, respectively. The most prominent difference in the DRT peak areas between HEP and PBF appears in the intermediate-frequency range, verifying that ion surface exchange processes are greatly enhanced in the HEP anode, consistent with its accelerated oxygen ion migration. To further support these findings, power output tests were systematically conducted on the SOFC, employing CO and H_2_ as sole fuels, respectively. The results demonstrate that disordered HEP achieves a substantially higher power density than ordered PBF materials (Fig. S35). Based on the above analysis, the mechanism for the enhanced performance is illustrated in Fig. 3h, i. Compared to PBF, the HEP features stronger hydration, which facilitates the chemical reforming of methane to generate more CO and H_2_. Additionally, the higher bulk oxygen ion diffusion coefficient *D*_chem_ in HEP enables fast reaction of oxygen ions with CO and H_2_. Consequently, HEP exhibits a much higher peak power density.

### Long-term stability and anti-coking capability of HEP-anode

**Fig. 4:**
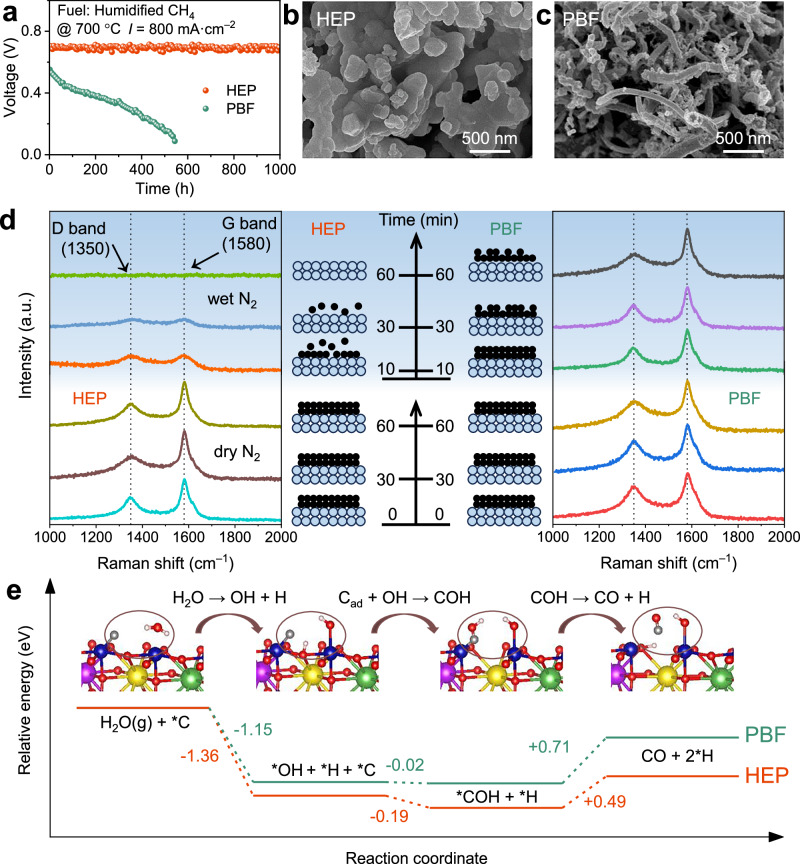
Long-term stability and anti-coking capability of HEP-anode. **a** Long-term stability at the constant current density 800 mA cm^−2^ at 700 °C (flow rate of CH_4_: 20 ml min^−1^). Cross-sectional SEM images of **b** HEP and **c** PBF anode after 1000 h durability testing under 800 mA cm^−2^ at 700 °C. **d** Raman spectra of carbon-contaminated HEP and PBF exposed to dry N_2_ and wet N_2_ (3 vol% H_2_O + 97% N_2_) for different times. The blue and black spheres represent the anode and carbon deposits, respectively. **e** Water-assisted removal of carbon from the PBF and HEP interface (carbon atom is labeled by gray color). The energy of water adsorption with the formation of intermediate surface species (*OH and *COH) and final CO desorption is presented. Gray, red, white, blue, purple, yellow, and green spheres represent carbon, oxygen, hydrogen, iron, gadolinium, praseodymium and barium atoms, respectively. Source data for Fig. 4 are provided as a Source Data file.

For SOFCs utilizing hydrocarbon fuels, anode carbon deposition remains a critical challenge to long-term stability. To evaluate the stability and anti-coking capability of the disordered HEP anode, a 1000-h durability test was conducted under humidified methane fuel (3% H_2_O). As shown in Fig. 4a, the single cell with the disordered HEP anode maintained a stable voltage under constant current operation, whereas the conventional ordered PBF anode suffered from significant voltage degradation. Notably, the stability of HEP surpasses most of the reported SOFC anodes operating under methane fuel (Fig. S36, Table S5). To further elucidate the reaction mechanism, we analyzed the exhaust gas composition before and after applying a constant current load during the stability test (Fig. S37). Specifically, the methane concentration remained constant upon current loading, while H_2_ and CO were rapidly consumed, confirming that methane is primarily converted via steam reforming rather than direct electrochemical oxidation. Notably, the HEP-based cell demonstrated a faster consumption rate of H_2_ and CO compared to the PBF-based cell, providing additional evidence for the enhanced electrochemical activity and stability of the HEP anode. Post-test scanning electron microscopy (SEM) analysis (Fig. 4b, c) confirmed that the disordered HEP anode maintained a smooth surface morphology, while the ordered PBF anode displayed extensive fibrous carbon deposition on its surface and within pores. This observation demonstrates the enhanced anti-coking performance of the disordered HEP anode, which likely stems from its high catalytic activity in MSR^10^. Moreover, XRD analysis of the post-test HEP sample (Fig. S38) revealed a monophasic structure, indicating excellent structural stability of HEP under the testing conditions.

To further elucidate the anti-coking mechanism of the disordered HEP anode, constant-current voltage tests were performed using dry methane as the fuel. As shown in Fig. S39, both anode types experienced voltage degradation upon methane exposure, confirming carbon deposition. However, when the fuel was switched back to humidified methane, the disordered HEP anode’s voltage rapidly recovered to its original level, while the ordered PBF anode continued to degrade. Furthermore, Raman spectroscopy was employed to detect possible carbon deposition on the SOFC anodes after operation (Fig. S40). While the PBF anode surface exhibited two characteristic coke bands (D-band at 1350 cm^–1^ and G-band at 1580 cm^–1^), no such signals were detected on HEP. This phenomenon suggests that water introduction facilitates carbon removal on the disordered HEP anode, highlighting the critical role of its strong hydration properties in mitigating coking.

In-situ high-temperature Raman spectroscopy was conducted to directly probe the hydration effect on carbon elimination. A carbon-contaminated disordered HEP anode (intentionally exposed to dry methane for 5 h at 700 °C) was loaded into the Raman chamber under dry N_2_ at 500 °C. The Raman spectra (Fig. 4d) revealed characteristic G and D bands of carbon, corresponding to graphitic and disordered carbon structures, respectively. Notably, exposure to dry N_2_ failed to reduce these signals, confirming the persistence of carbon. However, after switching to a wet N_2_ atmosphere, the carbon signals on the disordered HEP anode vanished within 60 min, while those on the ordered PBF anode remained intact. Figures S41, S42 provide the corresponding post-tested SEM images after exposure in dry and wet atmospheres for different time. After 60 min in a wet N_2_ atmosphere, carbon deposits on the HEP sample surface were completely removed, whereas carbon residues remained visible on the PBF sample surface throughout the entire test. The corresponding transformation process of carbon deposits on the anode surface is schematically illustrated in the middle of Fig. 4d. These results strongly demonstrate that the hydration-enabled disordered HEP anode plays a pivotal role in carbon removal, offering a promising solution for SOFC anode durability.

DFT calculations were employed to further elucidate the mechanism of coking tolerance in this disordered HEP anode, with the corresponding models provided in Figs. S43, S44. Figure 4e compares the reaction pathways for carbon oxidation between HEP and PBF models^53,57^. Notably, H_2_O exhibits stronger adsorption on the HEP surface, releasing a lower energy of –1.36 eV compared to PBF (–1.15 eV), enabling barrierless O–H bond cleavage and hydroxylation of HEP. Subsequent steps, including *OH migration to interfacial carbon, *COH formation at the interface, and *H release (leading to CO generation), remain exothermic for both systems with moderate kinetic barriers. Importantly, *COH formation on HEP releases significantly lower energy (–0.19 eV) than on PBF (–0.02 eV). While the final CO desorption step is endothermic, the overall carbon oxidation reaction maintains exothermicity at –1.06 eV for HEP versus –0.46 eV for PBF. The above results further highlight the distinct contribution of high hydration to anti-coking capability, elucidating the molecular basis for the enhanced long-term stability of the HEP anode.

## Discussion

In this work, we demonstrate that introducing five distinct rare-earth elements into the A-site of the PBF perovskite induces a structural transition from a layered ordered structure to a disordered configuration, resulting in the formation of a high-entropy perovskite (HEP). Compared to the ordered PBF, the disordered HEP possesses a higher oxygen vacancy concentration and accelerated oxygen exchange kinetics, which substantially boost its electrocatalytic activity. Additionally, the disordered configuration enhances the hydration capability of the anode, facilitating methane steam reforming and coking removal. Owing to the synergy of these beneficial properties, the SOFC single cell employing the HEP anode attains a maximum power density of 774.53 mW·cm^–2^ at 700 °C. Our findings provide valuable insights into the role of ionic ordering in determining perovskite anode properties, thereby establishing a foundation for the rational design of next-generation SOFC anode materials with enhanced performance.

## Methods

### Powder synthesis and characterization

The series anode powder materials, PrBaFe_2_O_5 + δ_ (PBF), La_0.5_Pr_0.5_BaFe_2_O_5 + δ_ (LPBF), La_0.33_Pr_0.33_Sm_0.33_BaFe_2_O_5 + δ_ (LPSBF), La_0.25_Pr_0.25_Sm_0.25_Gd_0.25_BaFe_2_O_5 + δ_ (LPSGBF), La_0.2_Pr_0.2_Sm_0.2_Gd_0.2_Y_0.2_BaFe_2_O_5 + δ_ (HEP), PrBaCo_2_O_5 + δ_ (PBC), PrBaMn_2_O_5 + δ_ (PBM) La_0.2_Pr_0.2_Sm_0.2_Gd_0.2_Y_0.2_BaCo_2_O_5 + δ_ (LPSGYBC) and La_0.2_Pr_0.2_Sm_0.2_Gd_0.2_Y_0.2_BaMn_2_O_5 + δ_ (LPSGYBM) were synthesized by the widely used sol-gel method. Taking the synthesis of HEP as an example, firstly, several nitrates such as Pr(NO_3_)_3_·6H_2_O (99.9%, Aladdin), La(NO_3_)_3_·6H_2_O (99.9%, Aladdin), Ba(NO_3_)_2_ (99.99%, Aladdin), etc. were weighed according to the chemical formula corresponding to the stoichiometric ratio and placed in a beaker, and 500 ml of deionized water was added. A certain amount of ethylenediaminetetraacetic acid (EDTA, 99.5%, Aladdin) as a complexing agent and citric acid (CA, 99.5%, Aladdin) as a reducing agent was then added to synthesize 0.01 mol of the sample, corresponding to 10 g of EDTA and CA, respectively, and the pH of the solution was then adjusted with ammonia to make it clear. The mixed solution was heated in a water bath at 80 °C with continuous stirring until it became a dark-colored transparent gel state. The gel was placed in a combustion oven and self-combusted at 250 °C for 2 h to obtain the precursor in the form of black powder. After simple grinding, the precursor was calcined in a muffle furnace at 1100 °C in an air atmosphere for 5 h to obtain the black powder, which is the anode material, with a single perovskite structure. In particular, the PBM and LPSGYBM oxide anodes were obtained by annealing in H_2_ at 800 °C.

### Fabrication of single cells

We chose to prepare button cells for use in electrochemical testing of materials. An electrolyte-supported single cell was prepared using SSZ (Sc_0.1_Zr_0.9_O_1.95_, 99.9% trace metals basis, Fuel Cell Materials) as the electrolyte. A certain amount of polyvinyl alcohol (PVA) solution was taken as a binder with a mass ratio of 1:0.6 between SSZ powder and PVA, and grinded to a dry powder. 0.32 g of powder was taken through a stainless steel mold and held at 16 MPa for 5 min to obtain round sheet blanks, which were calcined at 1450 °C for 5 h in a high temperature muffle furnace to obtain dense electrolyte sheets with a diameter around 1 cm. Ce_0.8_Gd_0.2_O_2-δ_ (GDC, 99.9%, Fuel Cell Materials) was spin-coated on both sides of the electrolyte as a barrier layer and calcined at 1400 °C for 5 h to densify it. HEP and La_0.6_Sr_0.4_Co_0.2_Fe_0.8_O_3_ (LSCF, 99.9%, Fuel Cell Materials) were used as the anode and cathode of the cell, respectively, and were coated on both sides of the GDC by screen printing, with an electrode area of 0.7 cm^2^. The slurry used for screen printing was formulated as 100 mg of electrode powder, 12 mg of soluble starch, 8 mg of ethyl cellulose and 0.2 ml of terpineol. The paste was uniformly dispersed in a mortar and applied to the cell with a 200-mesh screen, dried and calcined in a muffle furnace at 1100 °C for 2 h. Silver paste and silver wire were selected for the preparation of the collector. The silver paste was first uniformly covered on the electrode surface, and the silver wire was adhered to it after one-side winding treatment, followed by calcination at 800 °C for 5 h in a muffle furnace to prepare a single cell with the structure of HEP|GDC|SSZ|GDC|LSCF. For electrochemical impedance spectroscopy measurements, symmetric cells were fabricated by applying identical electrode deposition, coating and heat-treatment procedures to both sides of the electrolyte substrate, with consistent raw material ratios and calcination parameters.

### Electrochemical test

The single-cell tests described in this paper all use ceramic tubes as an auxiliary tool. The single cell was fixed to one end of the ceramic tube with ceramic adhesive and allowed to dry until the ceramic adhesive had completely set. The ceramic tube was then mounted in a muffle furnace, and the prepared anode-supported single cells were tested for discharge capacity and cycle life using an ARBIN BT-2 × 43 cell test system at a temperature of 550–700 °C (or 600–700 °C as indicated). All measured cell voltages were recorded directly without iR compensation or ohmic resistance correction. The gas at the outlet of the system was detected by gas chromatography (GC-2014C). When different fuels were used, ambient air was used as the oxidant, argon as the shielding gas (flow rate 50 ml min^–1^), and the anode test gas composition and flow rate were as follows

(1) Methane: 97 vol% CH_4_ + 3 vol% H_2_O, with a methane flow rate of 20 ml min^–1^.

(2) Methanol: 97 vol% CH_3_OH + 3 vol% H_2_O, the fuel gas stream is an evaporated mixture of methanol and water, the vapor flow rate is 20 ml min^–1^.

(3) Ethane: 95 vol% C_2_H_6_ + 5 vol% H_2_O, the ethane flow rate is 14 ml min^–1^.

(4) Propane: 90 vol% C_3_H_8_ + 10 vol% H_2_O, propane flow rate is 10 ml min^–1^.

(5) n-Butane: 85 vol% n-C_4_H_10_ + 15 vol% H_2_O, the flow rate of n-butane was 5 ml min^−1^ and the test temperature was between 600 °C and 700 °C.

### Characterization of physicochemical properties

The phase structure of the powders was characterized using an X-ray diffractometer (Rigaku Ulti mA IV) in the angular range of 20–80° with a Cu (Kα) diffraction source. Synchrotron X-ray powder diffraction (SXRPD) measurements, performed with a wavelength of 0.6888 Å, were carried out at the BL02U beamline of the Shanghai Synchrotron Radiation Facility (SSRF), and the SXRPD data were processed using GSAS software for Rietveld refinement. In addition, the microscopic morphology of the powders was observed by transmission electron microscopy (TEM, FEI TECNAI F20) and the corresponding energy dispersive X-ray spectra (EDS) of the materials as well as the selected area electron diffraction (SAED) maps were obtained. The single-cell cross-section and the carbon structure of the anode material were observed by scanning electron microscopy (SEM, FEI QUANTA-250). The collection of atomic-level HAADF-STEM images and EDS mapping of the materials was performed using a JEM-ARM300F type microscope. The surface chemical elemental information of the samples was analyzed by X-ray (MULTILAB2000, VG (Al Kα radiation)) photoelectron spectroscopy (XPS). Thermogravimetric analysis (TGA) was carried out to investigate the weight versus temperature variation of the samples under different atmospheres using a Netzsch STA449 F5 synchronous thermal analyzer, with test temperatures ranging from 200 to 800 °C and a ramp rate of 5 °C min^–1^. The relaxation kinetics of the sample were investigated by thermogravimetric analysis. After being pre-annealed in an 80% Ar / 20% O_2_ atmosphere at 500 °C for 12 h to reach equilibrium, the gas environment was abruptly switched to a wet stream (pH_2_O = 0.03 atm). The The nitrogen adsorption-desorption isotherm plots and Brunauer-Emmett-Teller (BET) were performed using a ChemBET chemical adsorption analyzer (Quantachrome). The mass change was monitored in situ until a new equilibrium was established. Electron paramagnetic resonance (EPR) spectra were recorded using a Bruker A300-10/12 spectrometer (Bruker GmbH, Germany). The temperature-programmed reduction of H_2_ (H_2_-TPR) was assessed by adding 120 mg of the anode powder in a U-type quartztube. The XANES and EXAFS spectra of Fe K-edge were obtained at BL11B beamline in Shanghai Synchrotron Radiation Facility (SSRF), and the energy was calibrated using the standard absorption edge data of Fe foil. The ECR tests were conducted using a Keithley 2460 source meter at 700 °C. During the test, the oxygen partial pressure was adjusted from 0.21 to 0.1 atm, and the normalized conductivity was recorded. Electrochemical impedance spectroscopy (EIS) measurements were performed on symmetric cells using an Autolab PGSTAT302 electrochemical workstation (Metrohm). The resulting EIS spectra were analyzed via two complementary approaches: the equivalent circuit method (ECM) and the distribution of relaxation time (DRT) method. The methane stability of the sample was evaluated by treating 0.3 g of it at 700 °C for different time in a tube furnace under a flowing gas mixture of 50 vol% CH_4_/N_2_. The phase stability before and after testing was examined by XRD.

### Catalytic reforming tests

Methane conversion tests at different temperatures and water contents were evaluated using a fixed-bed reactor. A 50 mg anode catalyst HEP or PBF was loaded into the center of the fixed-bed reactor and a mixed gas stream of wet methane (3 vol% ~ 50 vol% H_2_O) was passed through the inlet at a flow rate of 10 ml min^−1^, with nitrogen used as the inert gas (flow rate 20 ml min^–1^). After stabilization at 650 °C for 1.5 h, at which point the reaction reached steady state, the gas at the outlet of the system was detected by gas chromatography (GC-2014C).

### Carbon removal test with in Raman spectroscopy

The materials were treated in a tube furnace to investigate the carbon removal mechanism of high-entropy anode materials. To simulate carbon deposition on the anode surface, the materials were exposed to a methane atmosphere at 700 °C for 5 h. Raman spectroscopy showed successful coking of the material surface. After carbon deposition, the samples were treated in a dry or wet N_2_ (~ 3vol% H_2_O) atmosphere (flow rate: 50 ml min^–1^) at 500 °C for different times. The experiments were carried out using a Raman spectrometer (Renishaw RM 2000) to characterize the Raman spectra of the treated materials using a 633 nm laser source.

### Theoretical calculation method

First-principles density functional theory (DFT) computations were implemented within the Vienna Ab initio Simulation Package (VASP). The exchange-correlation energy was described using the generalized gradient approximation (GGA) with the Perdew–Burke–Ernzerhof (PBE) functional. A plane-wave basis set with a kinetic energy cutoff of 400 eV was adopted to expand the valence electron wave functions. Structural relaxation was performed until the total energy difference fell below 1 × 10⁻^4^ eV and the residual force on each atom was less than 0.02 eV Å⁻^1^. A Monkhorst–Pack k-point mesh of 1 × 1 × 1 was employed to sample the Brillouin zone. To eliminate artificial interactions between adjacent periodic images, a vacuum region thicker than 12 Å was introduced along the direction perpendicular to the slab surface. The (110) surface of HEP was chosen to represent the catalytic surface (see Supplementary Data 1, 2). We employed total energy calculations to determine the stable elemental distributions in five tailored high-entropy perovskite (HEP) compositions. The study utilized supercell models (size: 2√2a × 2√2a × 4a; content: 32 A-site, 32 B-site, and 192 O atoms) to describe the bulk properties. The compositions studied were: [(La_0.2_Pr_0.2_Sm_0.2_Gd_0.2_Y_0.2_)_1–*x*_Ba_*x*_][Ba_1–*x*_(La_0.2_Pr_0.2_Sm_0.2_Gd_0.2_Y_0.2_)_*x*_]Fe_2_O_5 + δ_ (*x* = 0, 0.2, 0.5, 0.7, 1). The most stable configuration for each material was identified as the one with the lowest system energy. Transition states were located using the climbing image-nudged elastic band (CI-NEB) method, and the corresponding potential energy surfaces were constructed. The adsorption energy (Δ*E*_ads_) was defined as

8 $$\Delta {E}_{{\rm{ads}}}={E}_{{\rm{ads}}}{\rm{\hbox{-}}}({E}_{{\rm{sur}}}+{E}_{{\rm{adsorbates}}})$$

where *E*_ads_, *E*_sur_, and *E*_adsorbates_ represent the total electronic energy of the adsorbed species on the surface, the bare surface, and the isolated gas-phase molecule (e.g., H_2_O), respectively.

It should be noted that DFT calculations adopt an idealized structural model, which cannot fully replicate the complex atmosphere and interface evolution under practical catalytic conditions.

### Calculation of configurational entropy

There are various concepts of high entropy materials, one of which is defined based on mixing entropy. The mixed entropy (*S*_mix_) consists of two parts, namely the ideal configuration entropy (*S*_config_) and the excess entropy (*S*_excess_), as shown in Eq. (9).

9 $${S}_{{\rm{mix}}}={S}_{{\rm{config}}}+{S}_{{\rm{excess}}}$$

However, excess entropy is difficult to quantify, so only the ideal configuration entropy is usually considered. When the configuration entropy is ≥ 1.61 *R*, it can be referred to as high entropy. For PBF perovskite materials with a structure of AA’B_2_O_5 + δ_, the calculation of the configuration entropy is as follows:

10 $${S}_{{\rm{config}}}={\rm{\hbox{-}}}R\left[\left(\sum {C}_{i}\mathrm{ln}{C}_{i}\right)_{\Pr -{\rm{site}}}+\left(\sum {C}_{i}\mathrm{ln}{C}_{i}\right)_{{\rm{Ba}}-{\rm{site}}}+\left(\sum {C}_{i}\mathrm{ln}{C}_{i}\right)_{{\rm{Fe}}-{\rm{site}}}\right]$$

Where *R* is the gas molar constant and *C*_*i*_ is the fraction of the i-th component.

In the case of HEP, when a fraction of Ba (0 < *x* < 1) occupies the A-site, the chemical formula can be expressed as [(La_0.2_Pr_0.2_Sm_0.2_Gd_0.2_Y_0.2_)_1–*x*_Ba_*x*_][Ba_1–*x*_(La_0.2_Pr_0.2_Sm_0.2_Gd_0.2_Y_0.2_)_*x*_]Fe_2_O_5 + δ_, and the corresponding configurational entropy is given by:

11 $${S}_{{\rm{config}}} 	={\rm{\hbox{-}}}R[(\sum {C}_{i}{\mathrm{ln}}{C}_{i})_{\Pr -{\rm{site}}}+(\sum {C}_{i}{\mathrm{ln}}{C}_{i})_{{\rm{Ba}}-{\rm{site}}}+(\sum {C}_{i}{\mathrm{ln}}{C}_{i})_{{\rm{Fe}}-{\rm{site}}}]\\ 	={\rm{\hbox{-}}}R[\left(\right.(5\times 0.2\times (1{\rm{\hbox{-}}}x)\times {\mathrm{ln}}(0.2\times (1{\rm{\hbox{-}}}x))+x\times {\mathrm{ln}}x)+\left(\right.(1{\rm{\hbox{-}}}x) \\ 	 \quad \times {\mathrm{ln}}(1{\rm{\hbox{-}}}x)+5\times 0.2x\times {\mathrm{ln}}(0.2x)+0]\\ 	={\rm{\hbox{-}}}2R[(1{\rm{\hbox{-}}}x){\mathrm{ln}}(1{\rm{\hbox{-}}}x)+x{\mathrm{ln}}x]+R{\mathrm{ln}}5$$

## Supplementary information

Supplementary Information

Description of Additional Supplementary Files

Supplementary Data 1

Supplementary Data 2

Transparent Peer Review file

## Source data

Source Data

## Data Availability

All data that support the findings of this study are provided within the paper and its Supplementary Information. All additional information is available from the corresponding authors upon request. Source data are provided with this paper.
