# Supplementary material for: Entropy-driven order-to-disorder transition in perovskite anodes for high-performance solid oxide fuel cells
Source: Nat Commun. 2026 Jun 19;17:7721. doi: 10.1038/s41467-026-74549-0 (PMC13434558; doi:10.1038/s41467-026-74549-0)
Supplement: Supplementary file 1 [file 41467_2026_74549_MOESM1_ESM.pdf]

Supplementary information

Entropy-Driven Order-to-Disorder Transition in Perovskite Anodes for High-Performance Solid Oxide Fuel Cells

Gaige Wang[1], Rongzheng Ren[1]*, Xiaodan Yu[1], Chunming Xu[1], Jinshuo Qiao[1], Wang Sun[1], Zhenhua Wang[1]*, and Kening Sun[1]*

[1]Beijing Key Laboratory of Green Hydrogen and Fuel Cells, School of Chemistry and Chemical Engineering, Beijing Institute of Technology, Beijing 100081, China

E-mail: renrongzheng@bit.edu.cn (Rongzheng Ren). wangzh@bit.edu.cn (Zhenhua Wang). sunkn@bit.edu.cn (Kening Sun)

Supplementary Note 1

Design Rationale of the High-Entropy Perovskite Anode Material

La, Pr, Sm, Gd, Y are selected as equimolar A-site rare-earth elements for constructing $La_{0.2}Pr_{0.2}Sm_{0.2}Gd_{0.2}Y_{0.2}BaFe_2O_{5+\delta}$ (HEP) based on three key considerations:

These elements have been successfully incorporated into layered double perovskite lattices in previous studies, verifying their stable existence without severe lattice distortion or phase decomposition, and providing a reliable experimental foundation for synthesis.[1-4]

As non-radioactive rare-earth elements with low cost and easy accessibility, their selection ensures experimental repeatability and potential for subsequent practical application.

The core goal of this work is to elucidate configurational entropy regulation on Ln/Ba ordering and electrochemical properties. Equimolar doping of five elements maximizes A-site configurational entropy, which is the key to complete disruption of the original ordered layered structure and formation of a disordered high-entropy perovskite, directly serving the study of entropy-driven structural modulation mechanisms.

Supplementary Figure

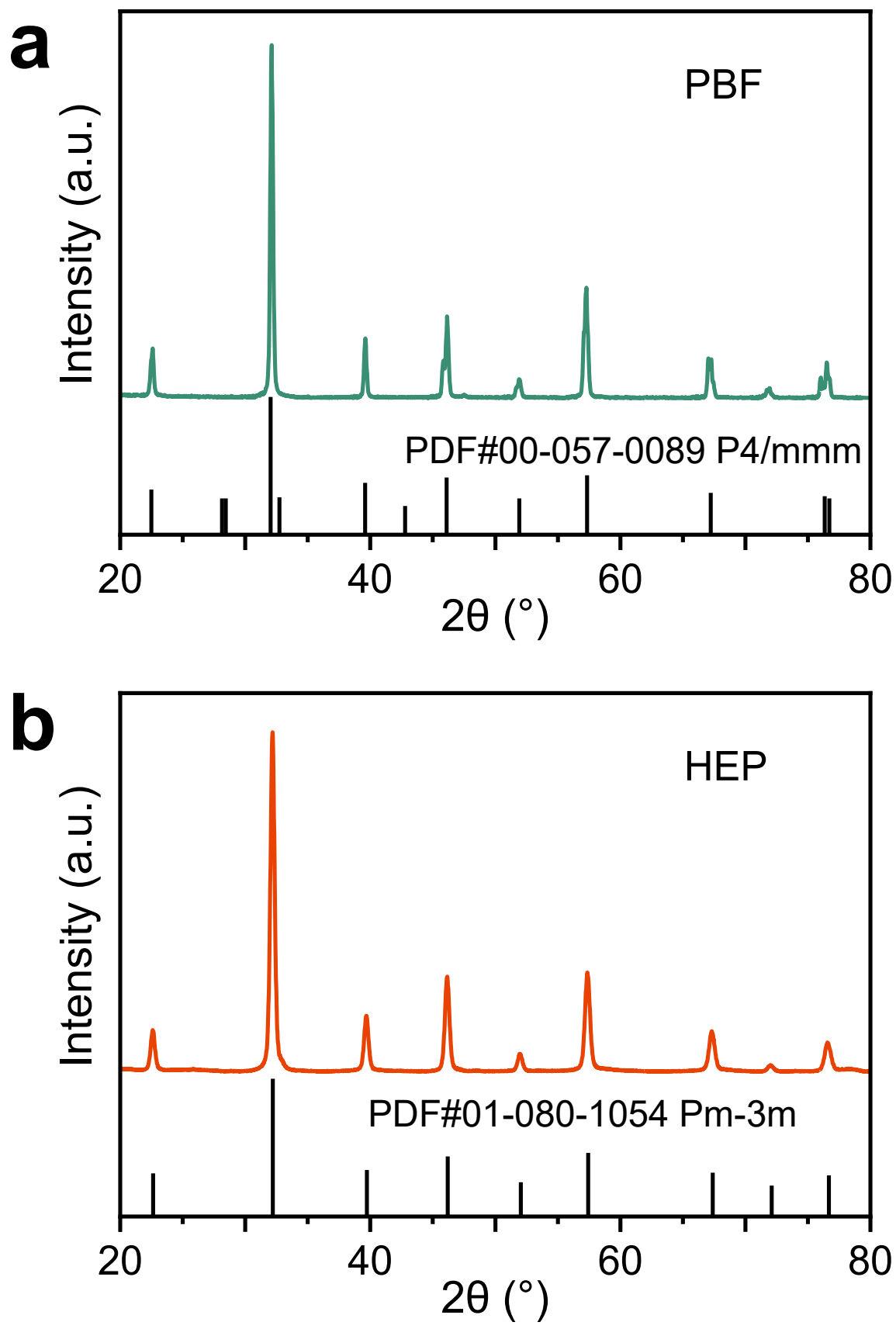

Fig. S1. XRD patterns of (**a**) PBF and (**b**) HEP. Source data for **Fig. S1** are provided as a Source Data file.

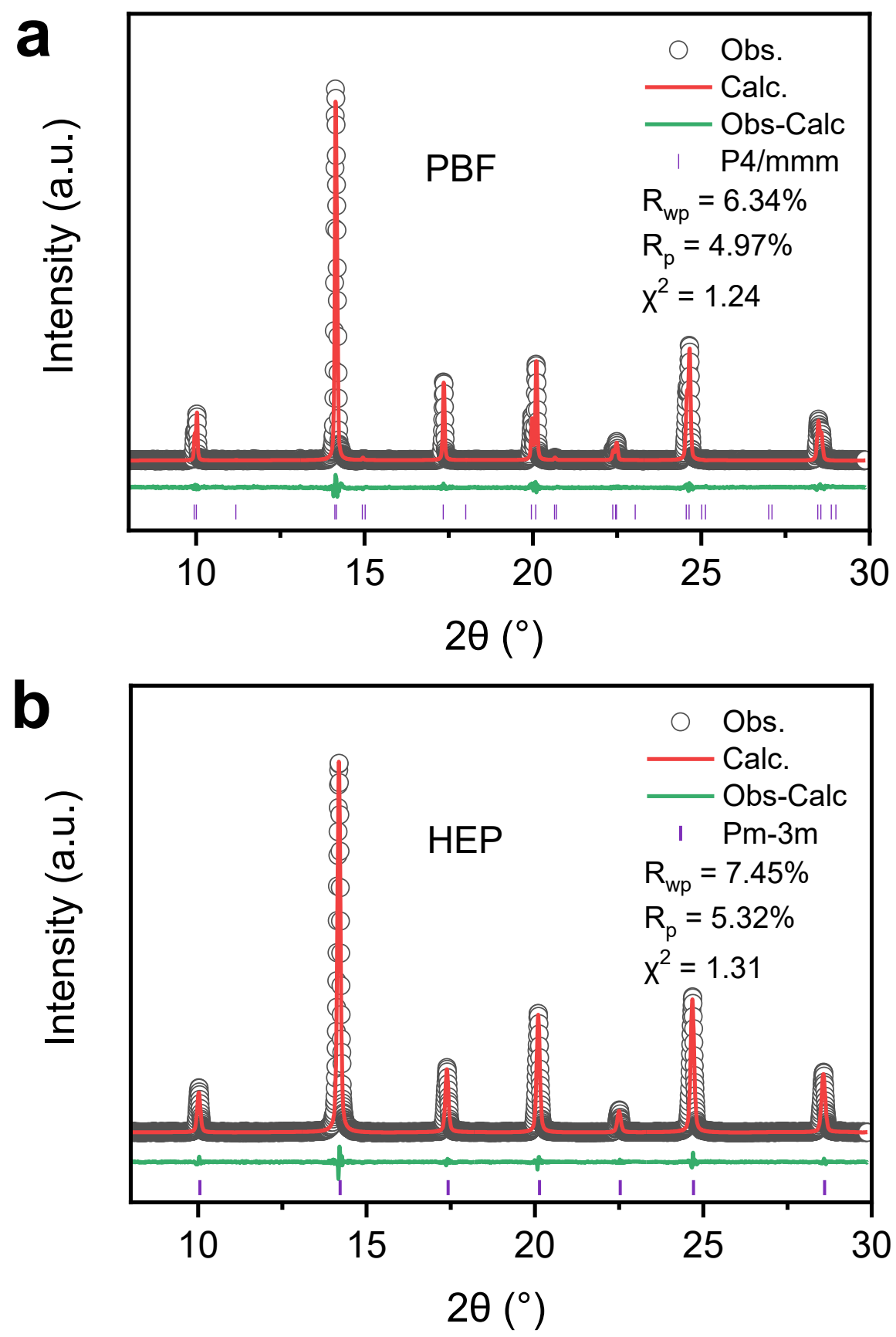

Fig. S2. Rietveld refinement of (**a**) PBF and (**b**) HEP SXRPD data. Source data for **Fig. S2** are provided as a Source Data file.

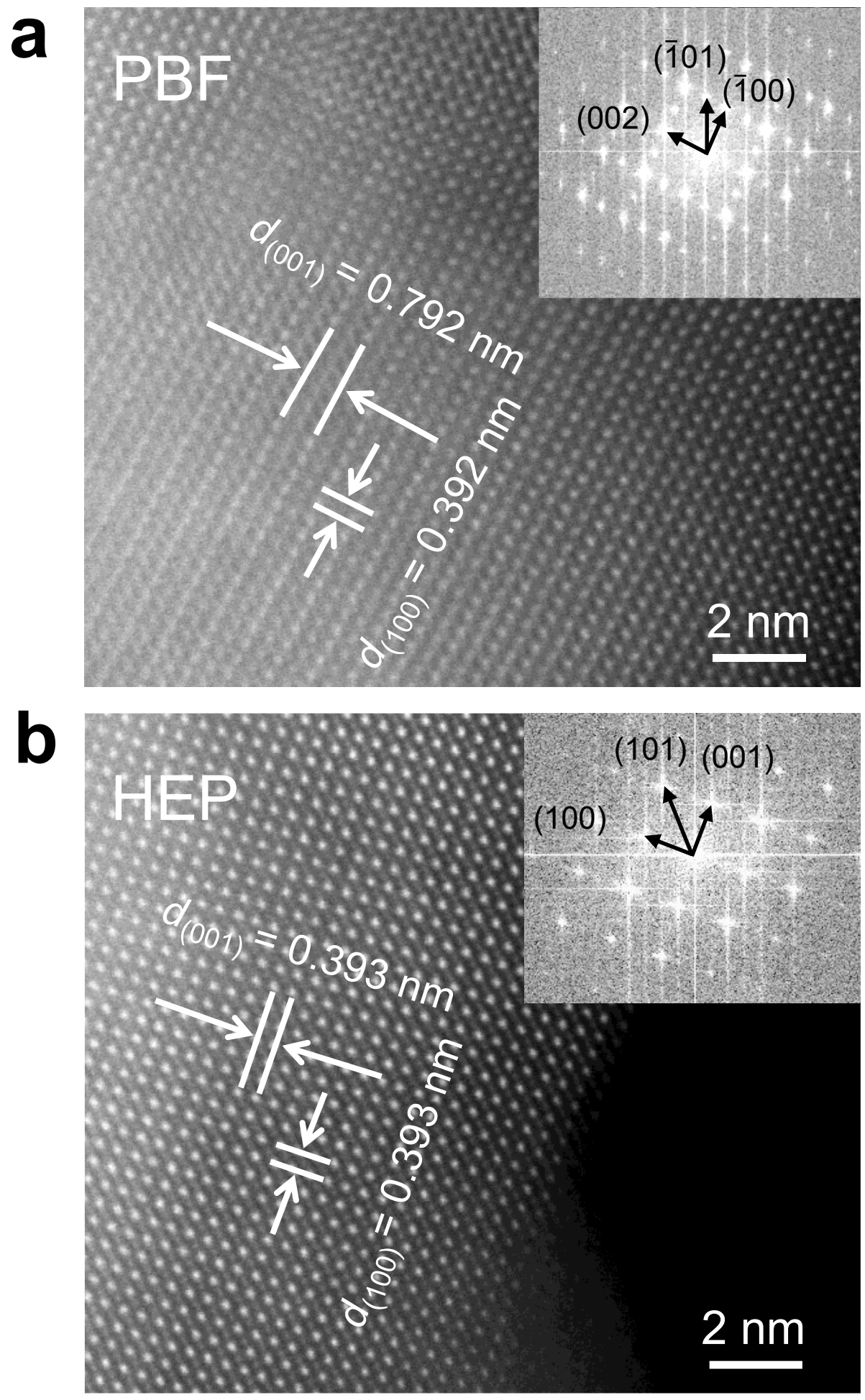

Fig. S3. HAADF-STEM images and Fast Fourier transform patterns of (**a**) PBF and (**b**) HEP.

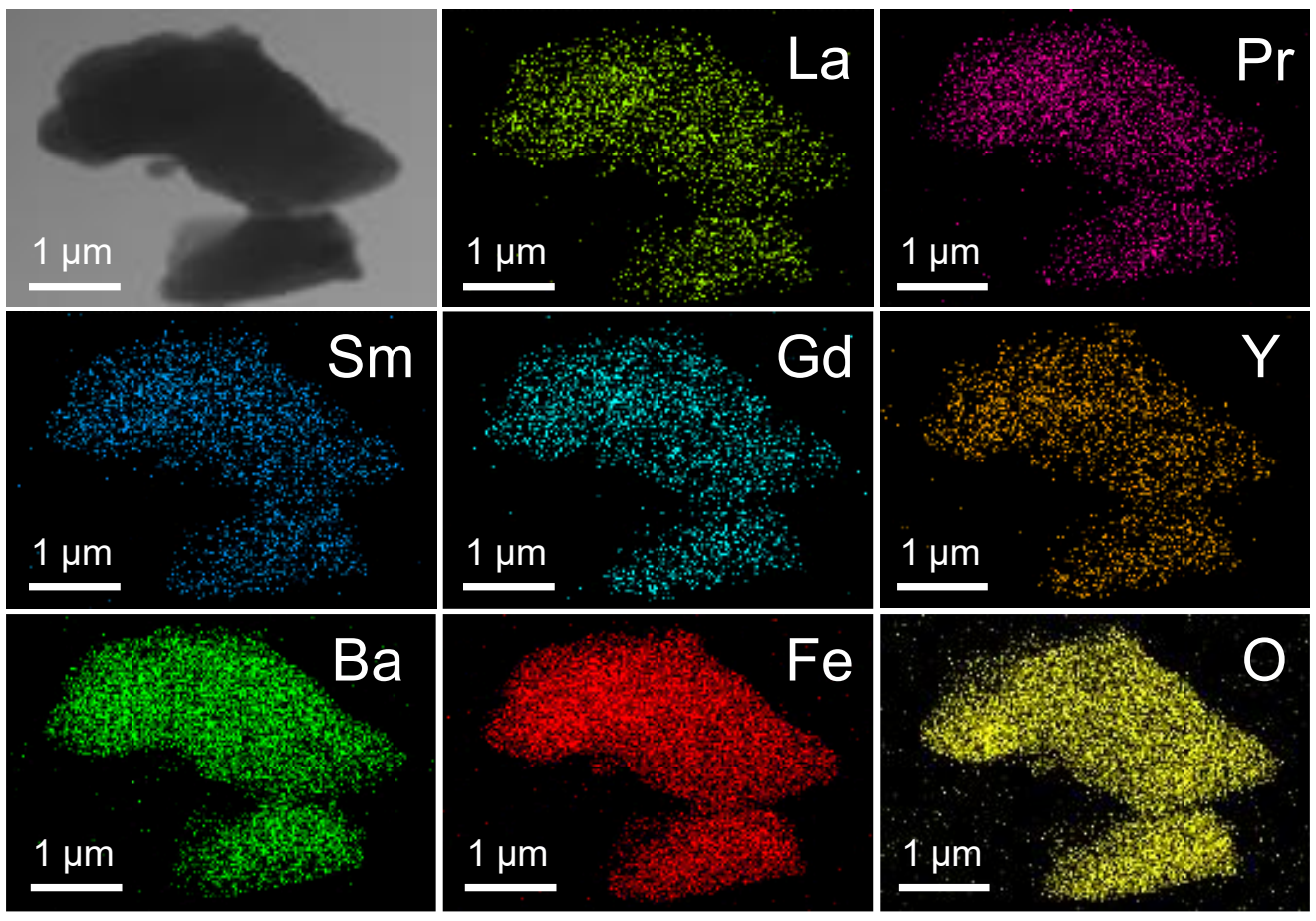

Fig. S4. TEM-EDS mapping of element distributions in the HEP sample.

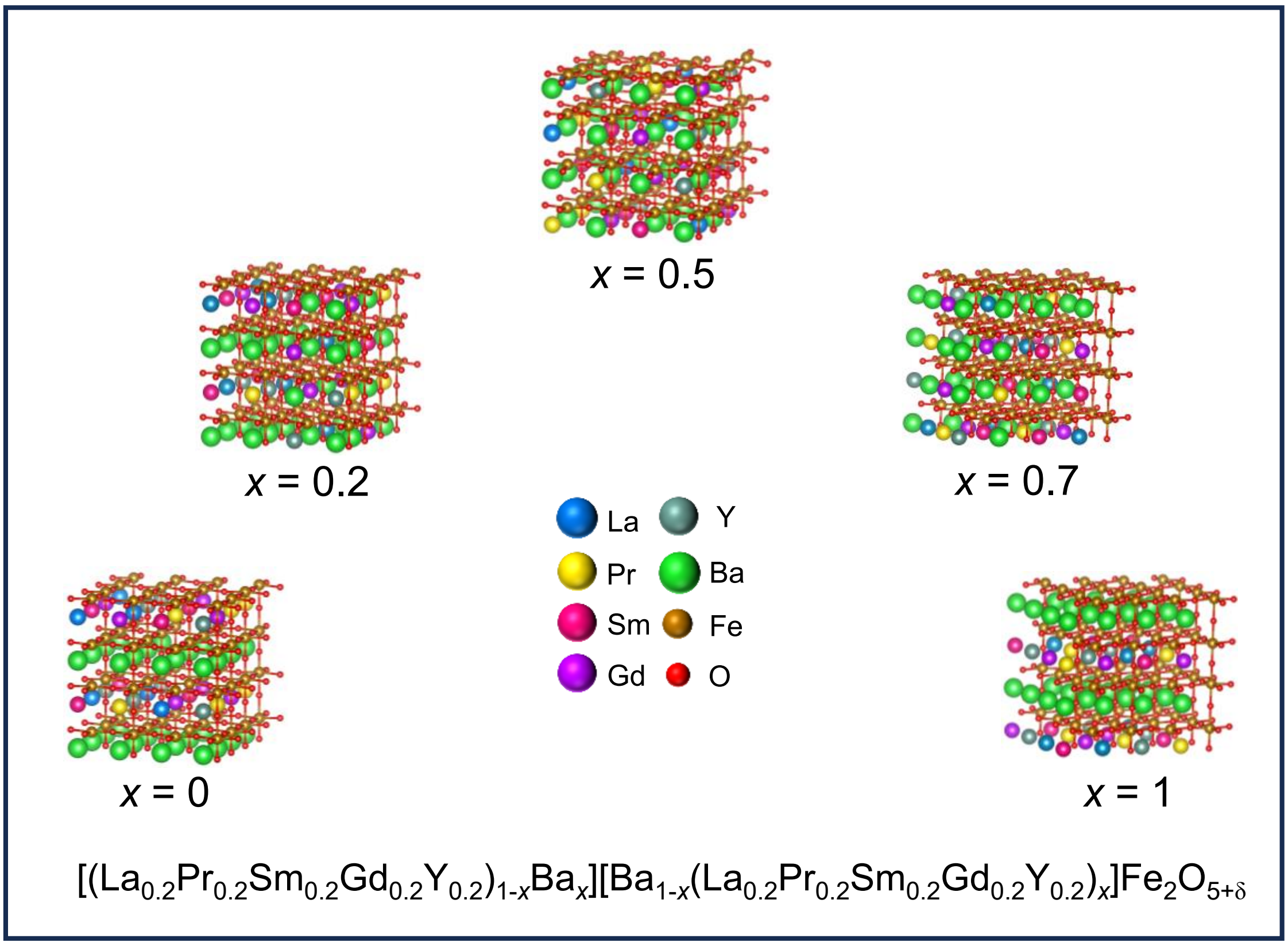

Fig. S5. Several representative structure models with varying Ba^{2+} substitution in the Pr-layer ($x = 0, 0.2, 0.5, 0.7, 1$).

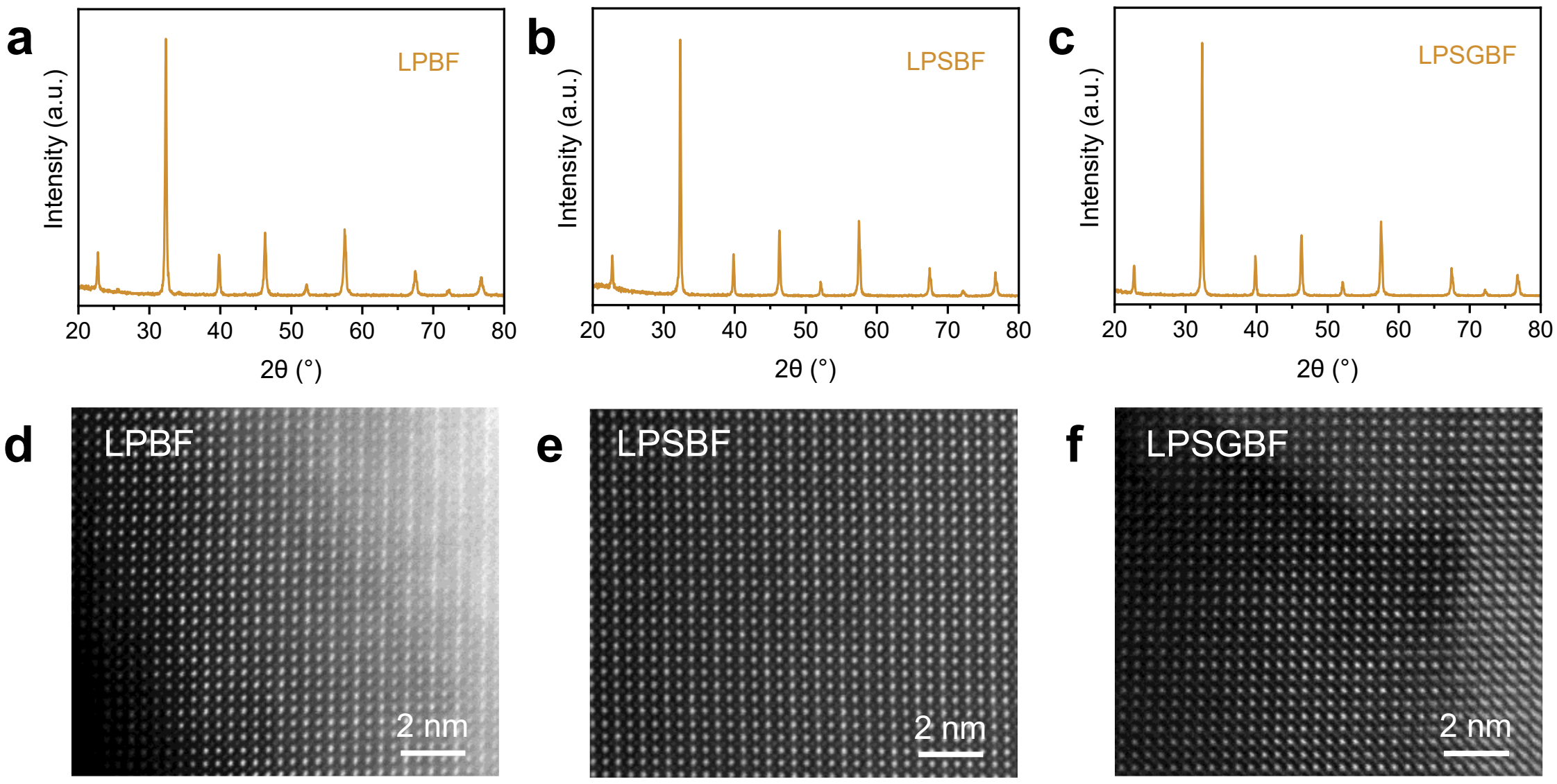

Fig. S6. XRD patterns of (**a**) LPBF, (**b**) LPSBF and (**c**) LPSGBF. HAADF-STEM images of (**d**) LPBF, (**e**) LPSBF and (**f**) LPSGBF. Source data for **Fig. S6** are provided as a Source Data file.

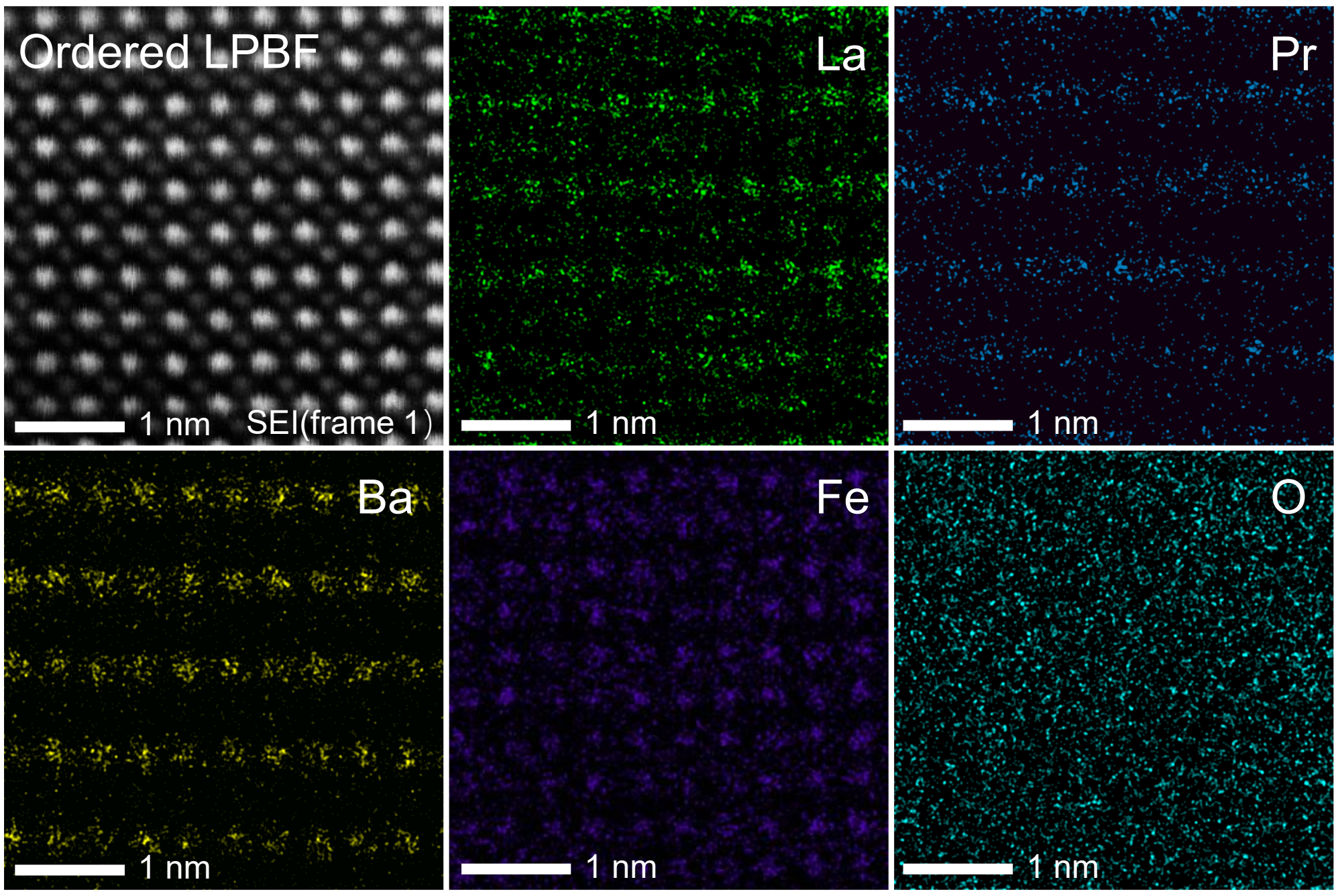

Fig. S7. HAADF-STEM elemental maps of LPBF.

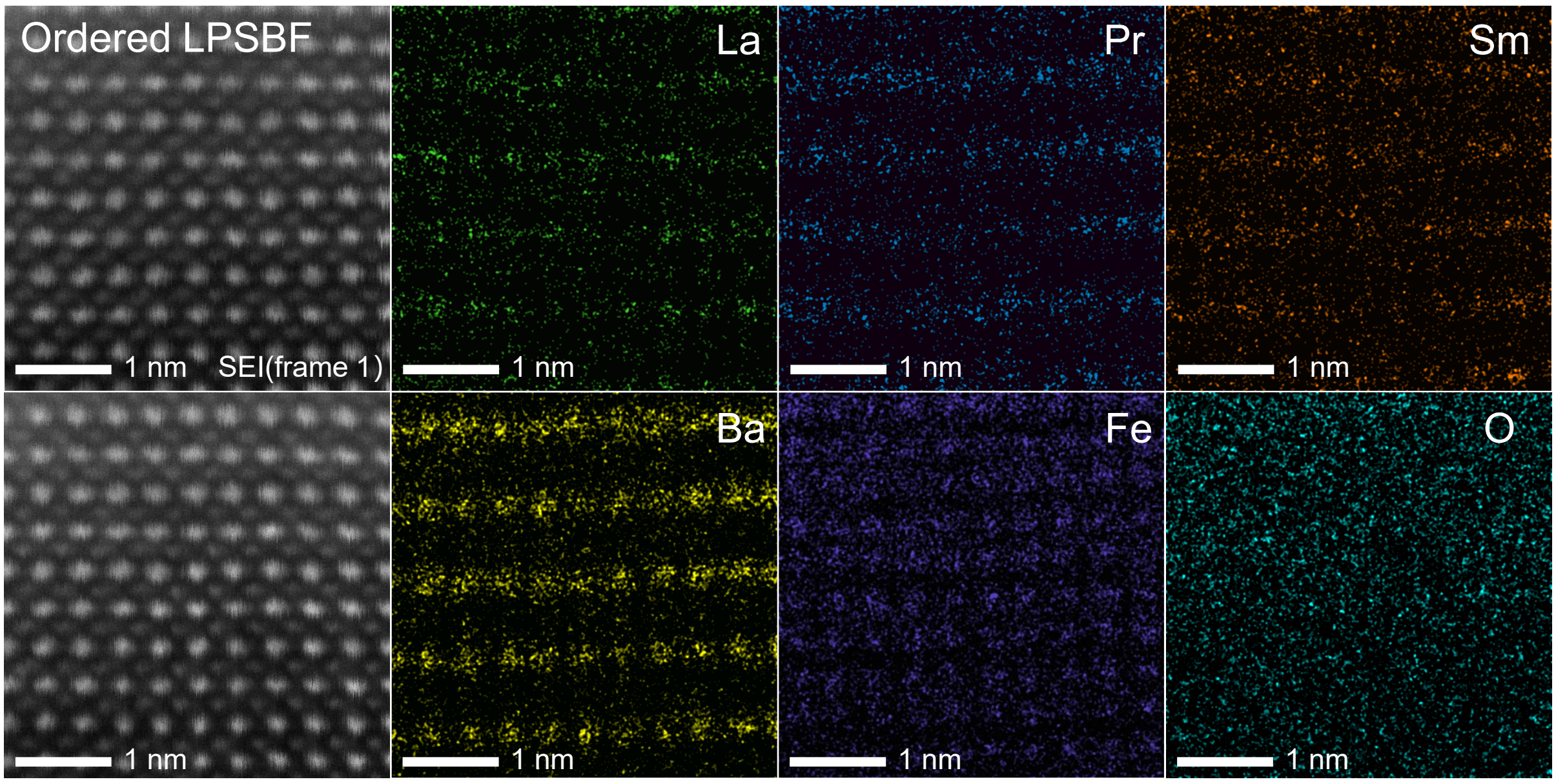

Fig. S8. HAADF-STEM elemental maps of LPSBF.

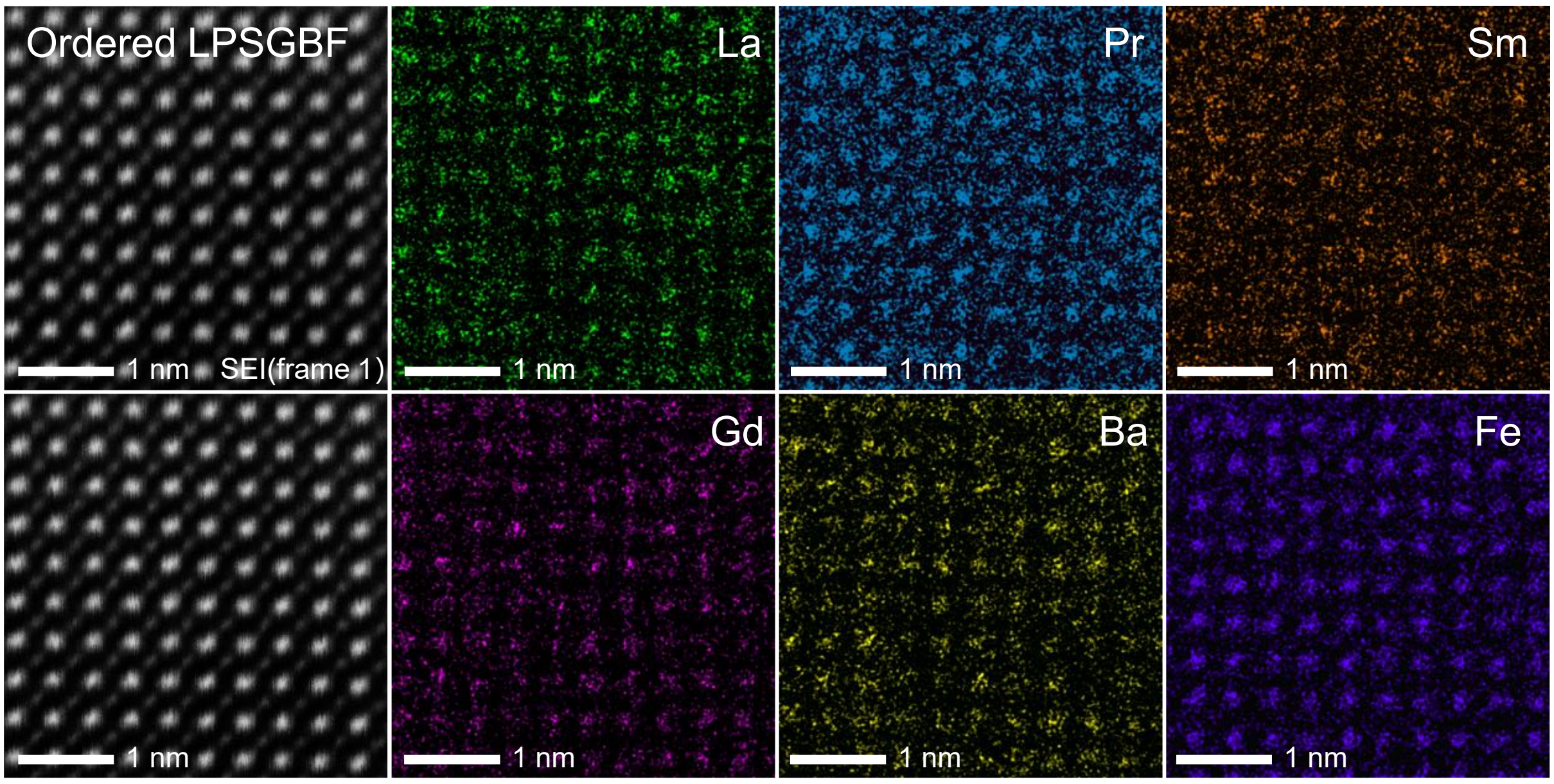

Fig. S9. HAADF-STEM elemental maps of LPSGBF.

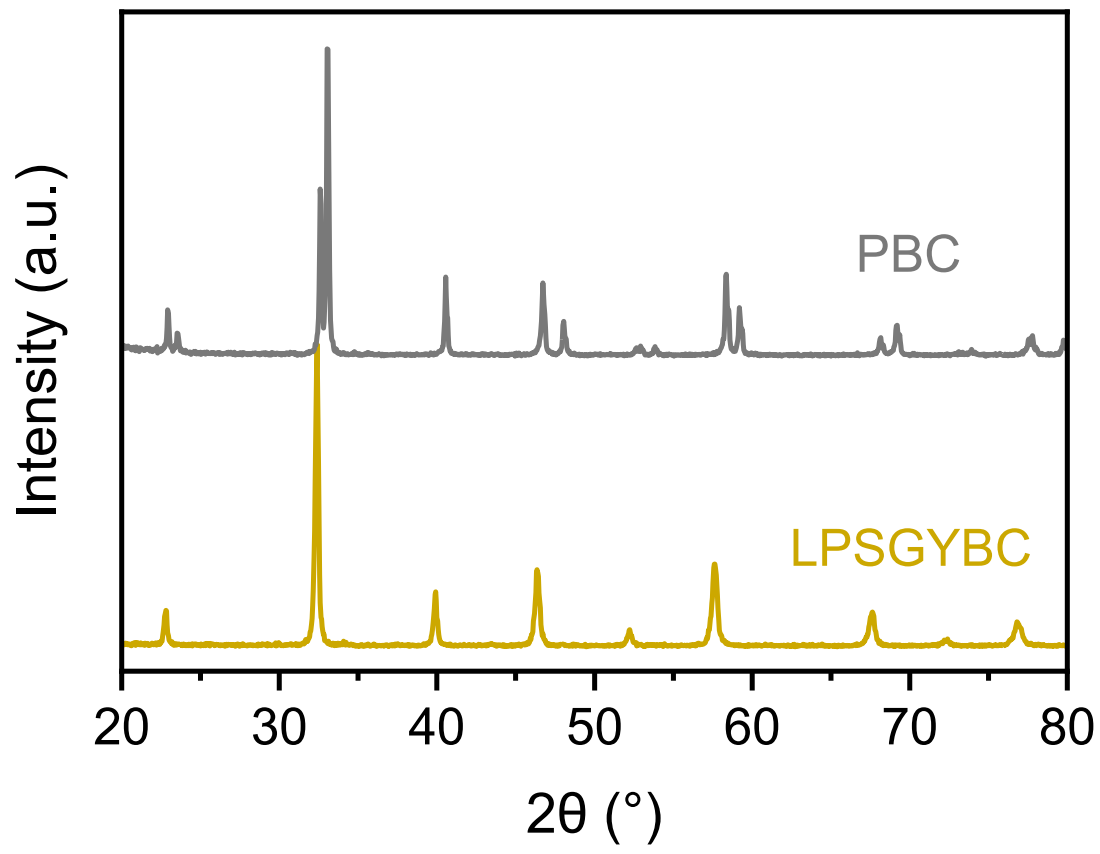

Fig. S10. XRD patterns of PBC and LPSGYBC. Source data for **Fig. S10** are provided as a Source Data file.

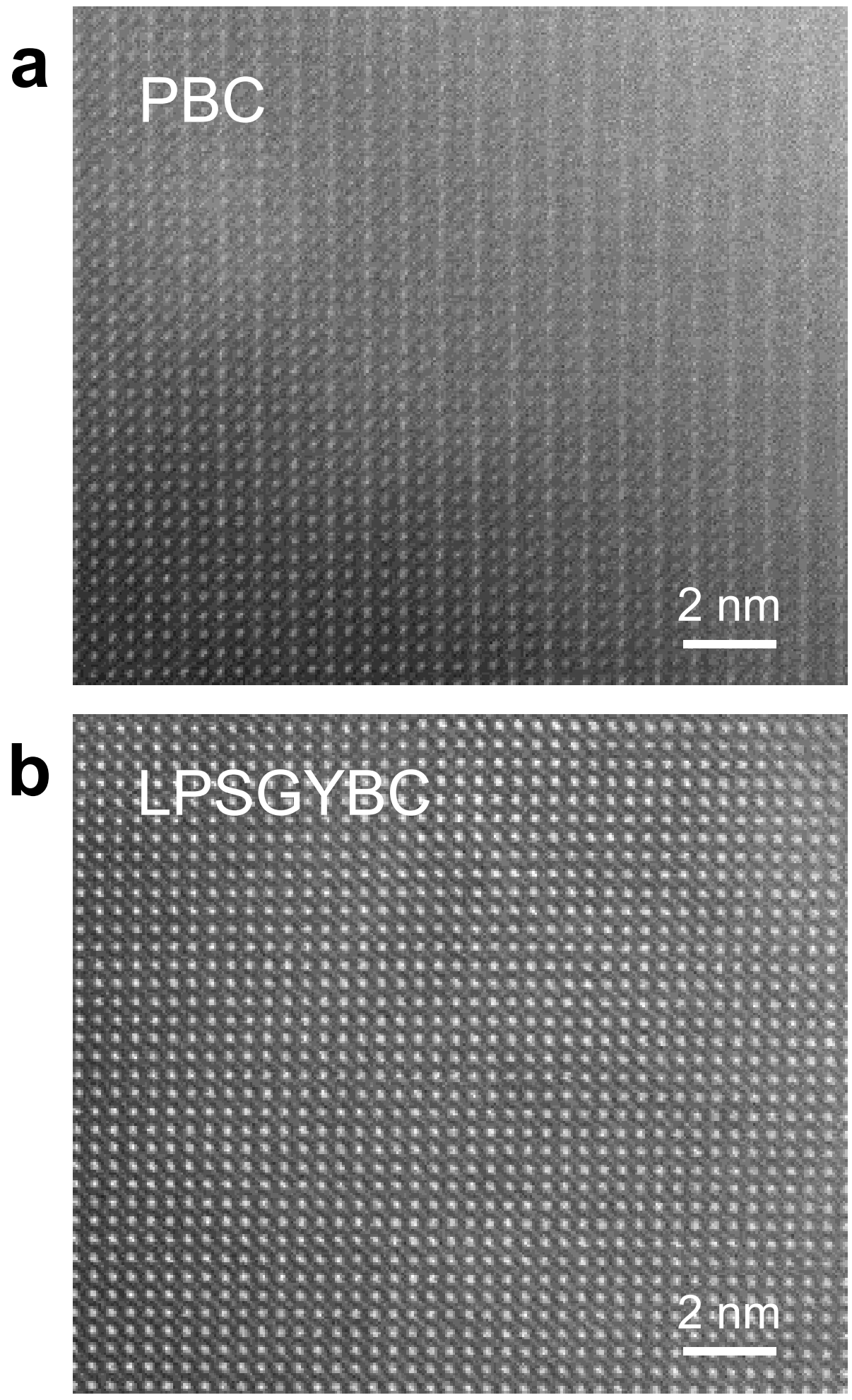

Fig. S11. HAADF-STEM images of (**a**) PBC and (**b**) LPSGYBC.

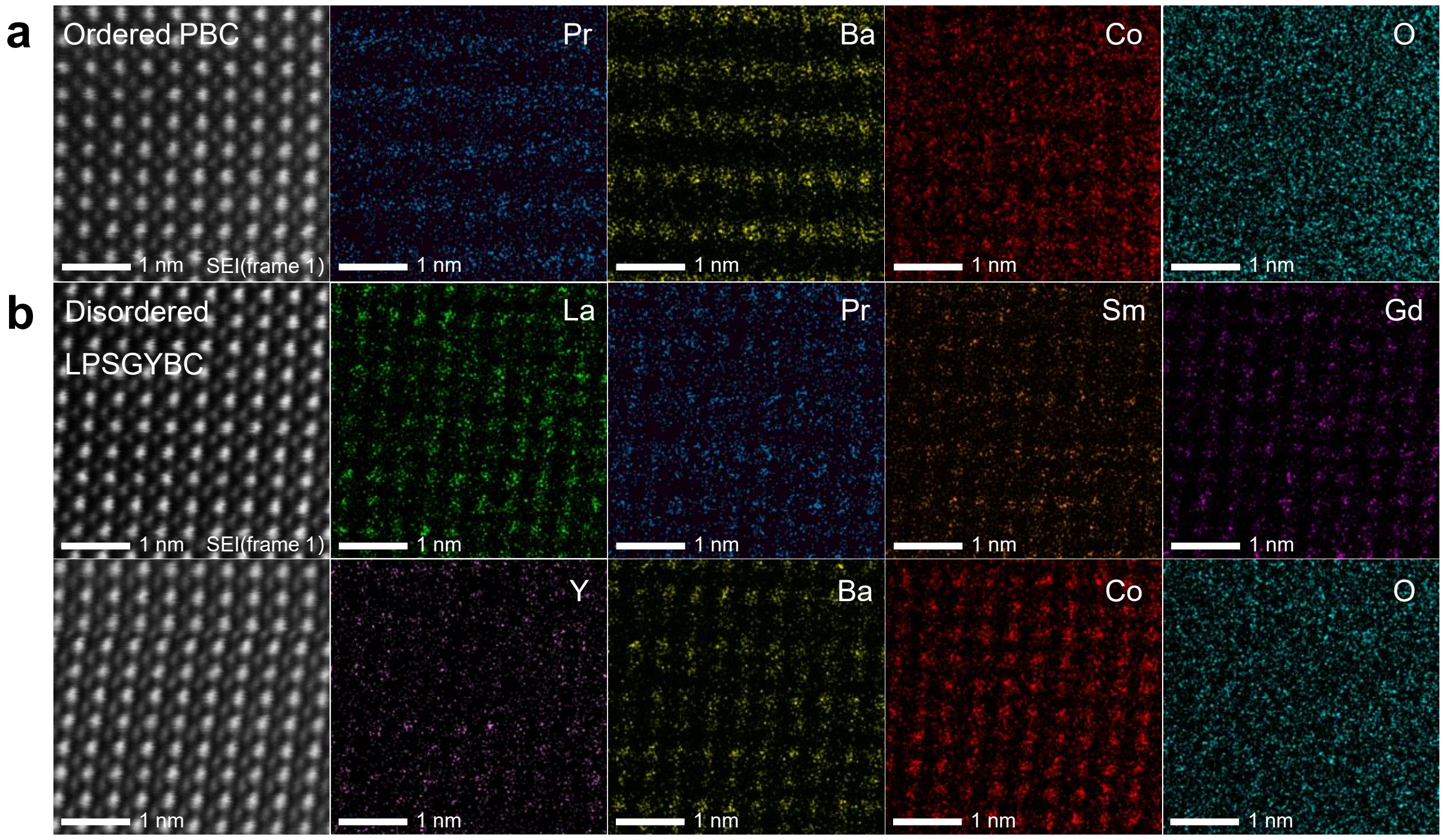

Fig. S12. HAADF-STEM elemental maps of (**a**) PBC and (**b**) LPSGYBC.

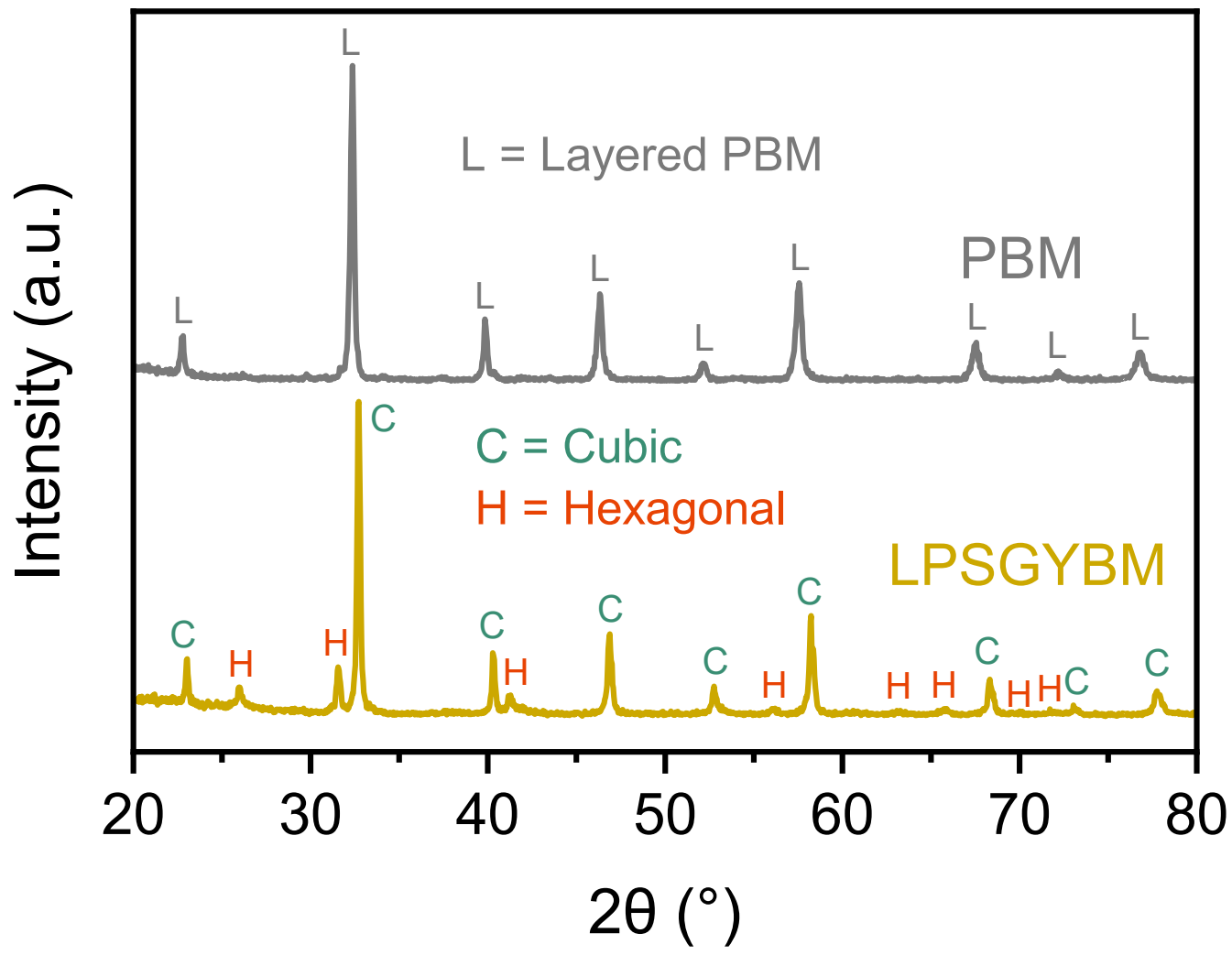

Fig. S13. XRD patterns of PBM and LPSGYBM. Source data for **Fig. S13** are provided as a Source Data file.

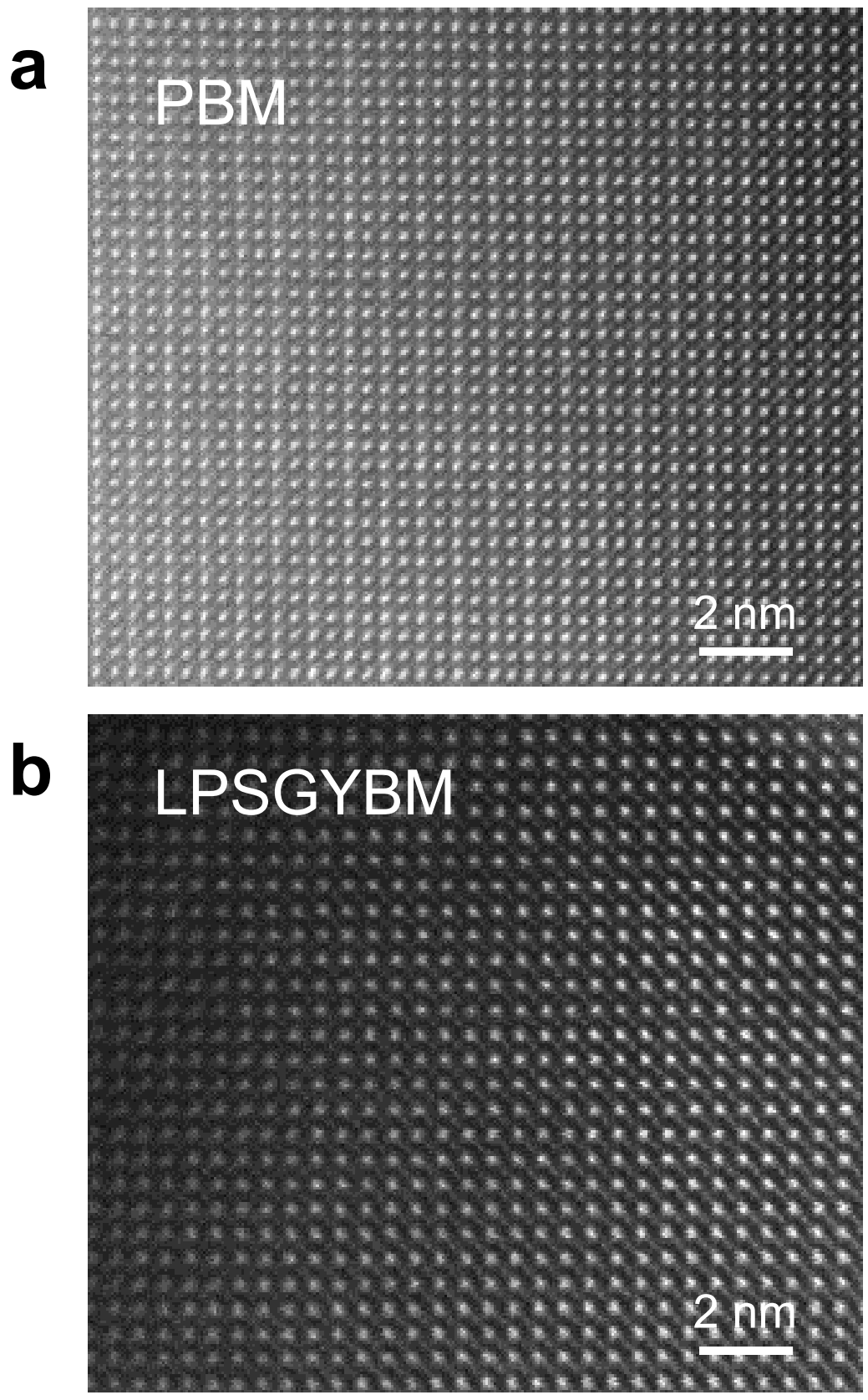

Fig. S14. HAADF-STEM images of (**a**) PBM and (**b**) LPSGYBM.

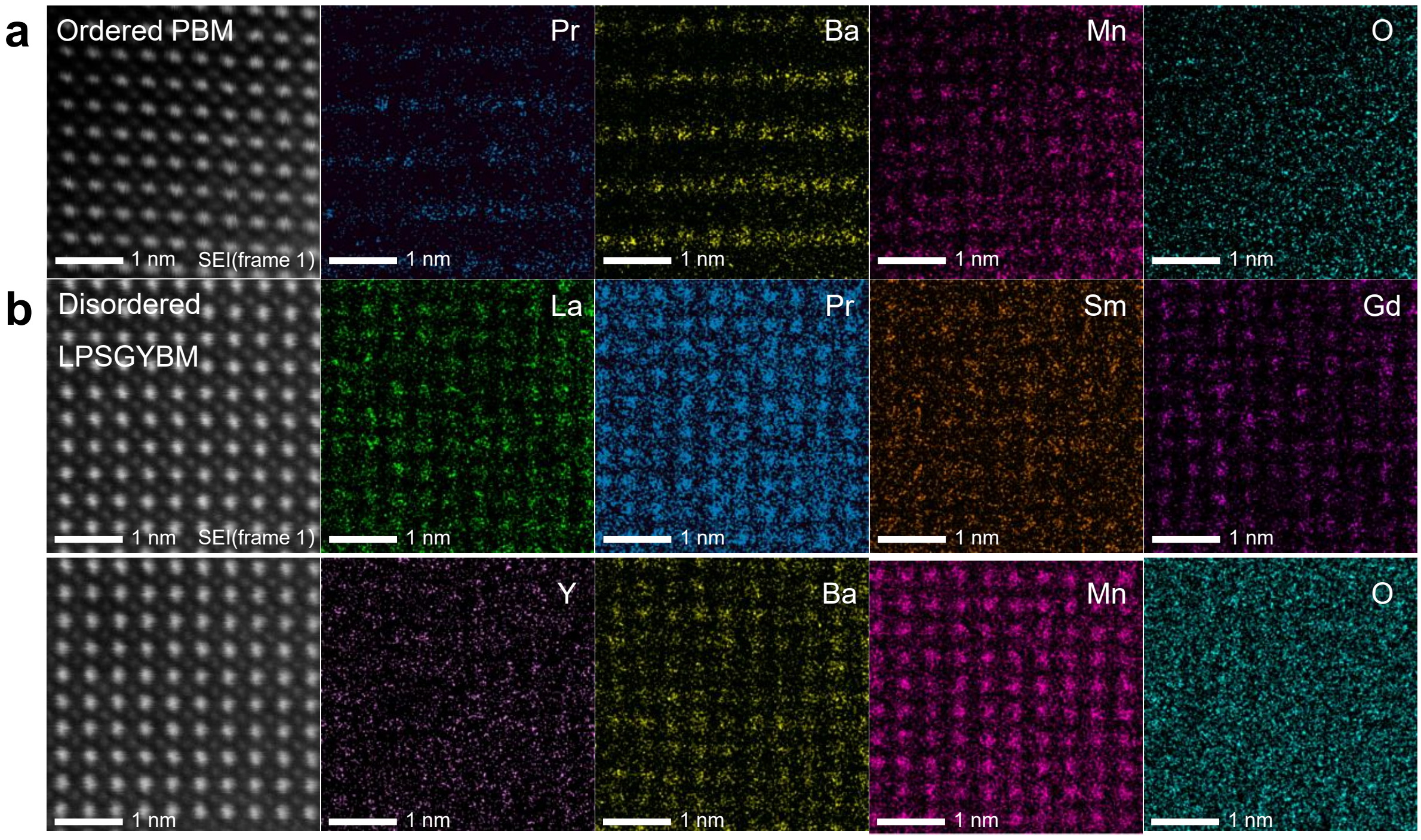

Fig. S15. HAADF-STEM elemental maps of (**a**) PBM and (**b**) LPSGYBM.

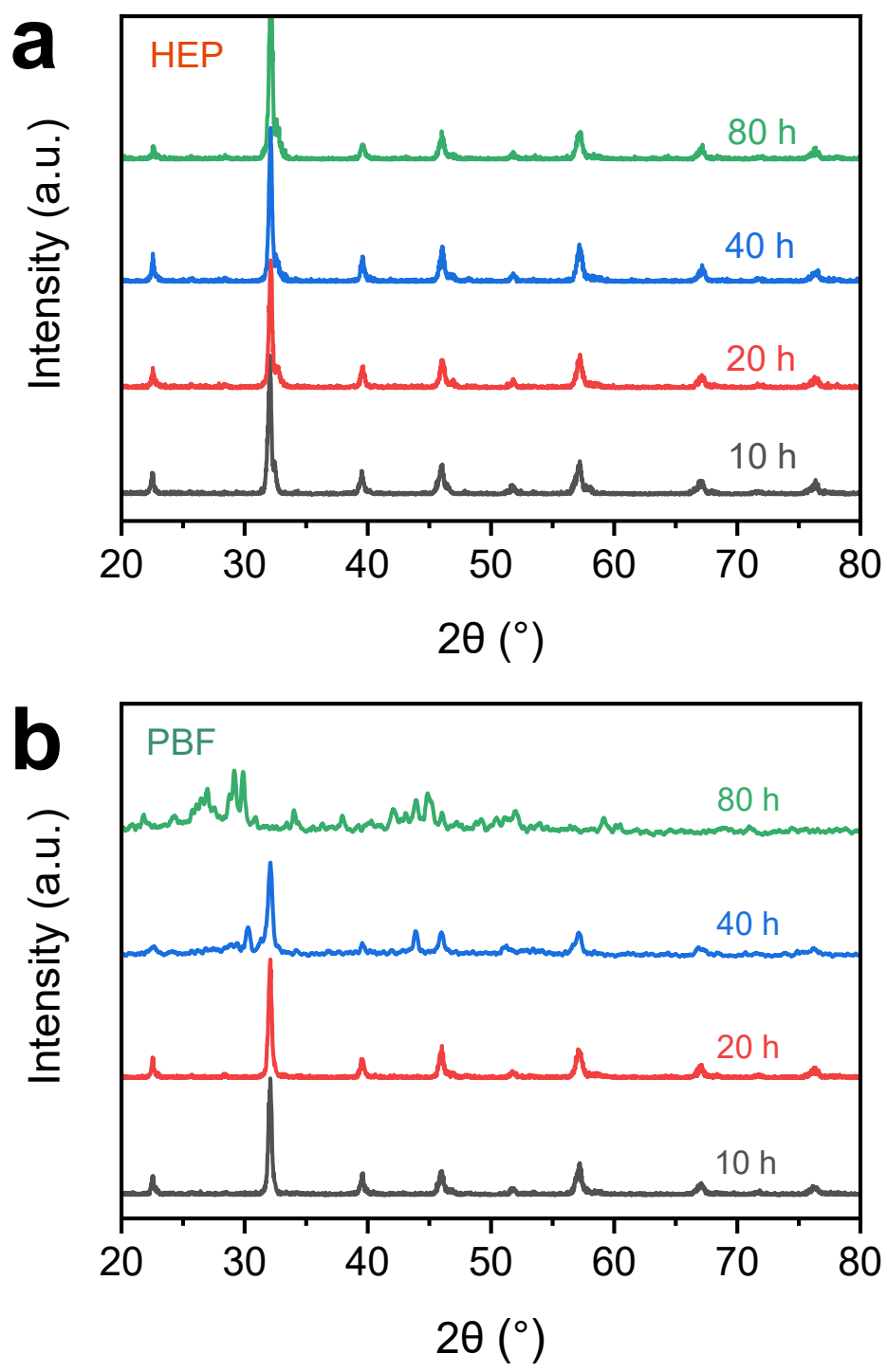

Fig. S16. XRD patterns of (**a**) HEP and (**b**) PBF reduced at 700 °C in 50 vol% CH_4/Ar for different time. Source data for **Fig. S16** are provided as a Source Data file.

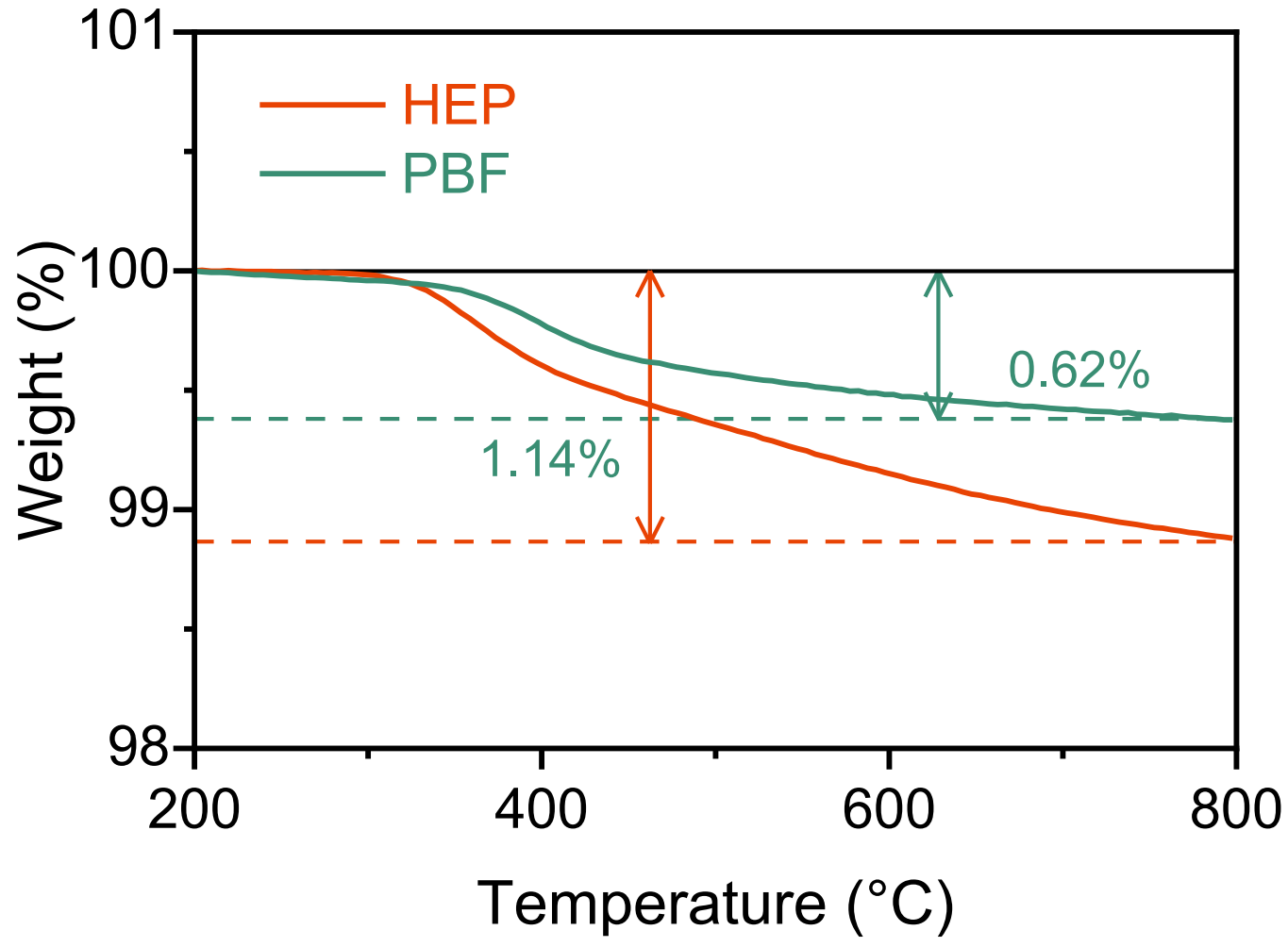

Fig. S17. TG patterns of HEP and PBF in the temperature range of 200−800 °C in air. Source data for **Fig. S17** are provided as a Source Data file.

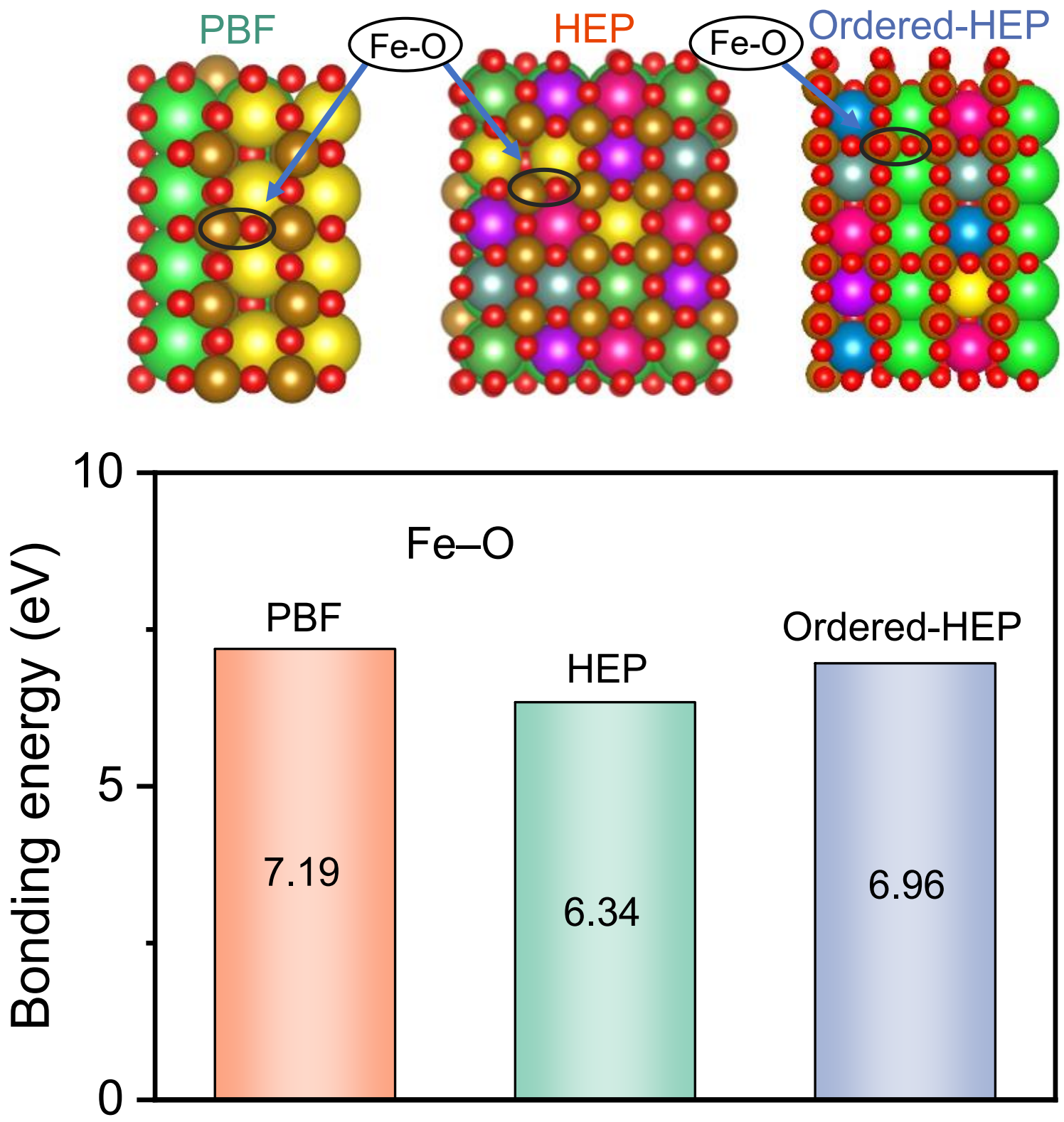

Fig. S18. Comparison of the Fe–O bond energies of different anode. In the structural model, red, brown, purple, yellow, green, gray, pink, and blue spheres represent oxygen, iron, gadolinium, praseodymium, barium, yttrium, samarium, and lanthanum atoms, respectively.

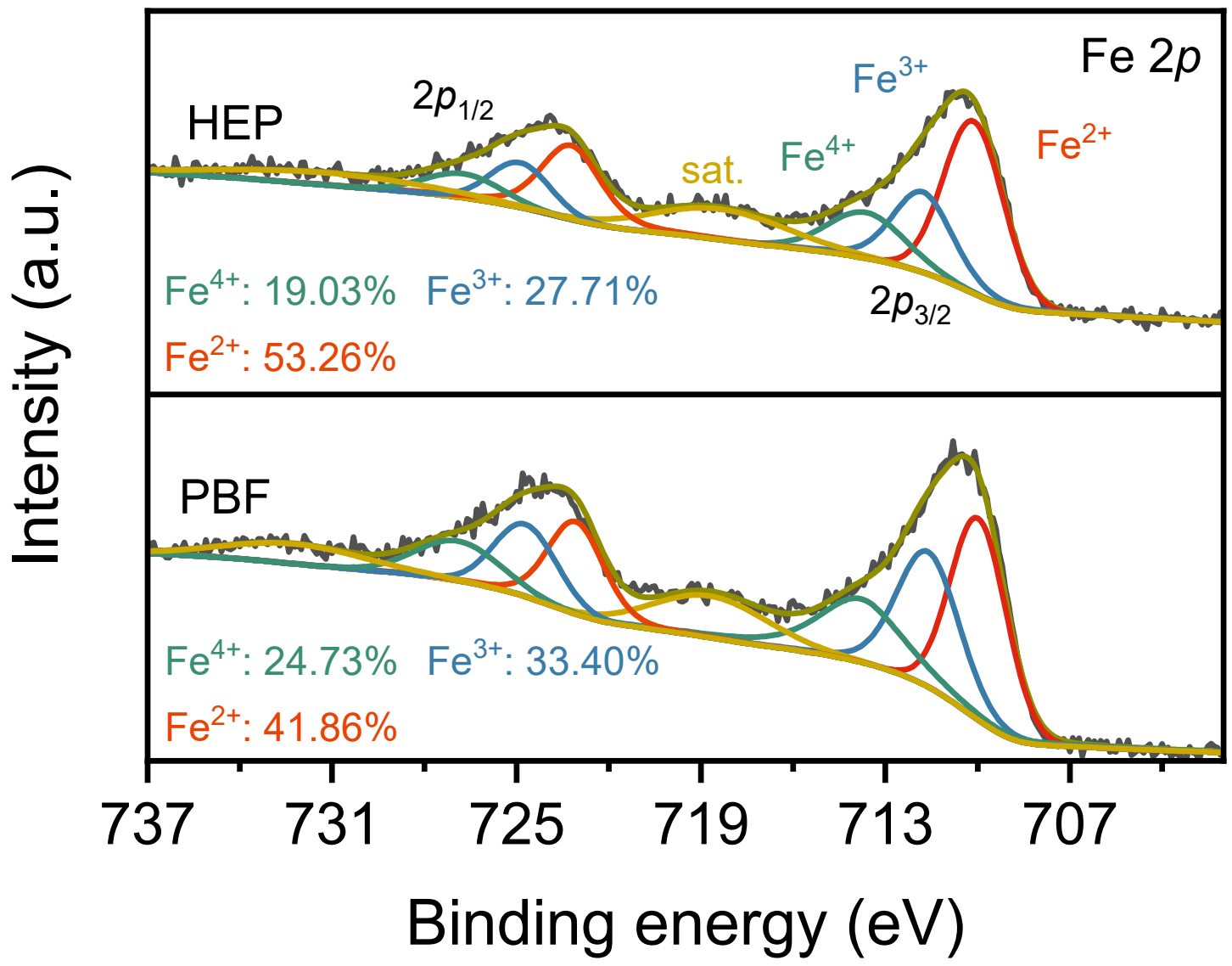

Fig. S19. Fe 2p XPS spectra of HEP and PBF. Source data for **Fig. S19** are provided as a Source Data file.

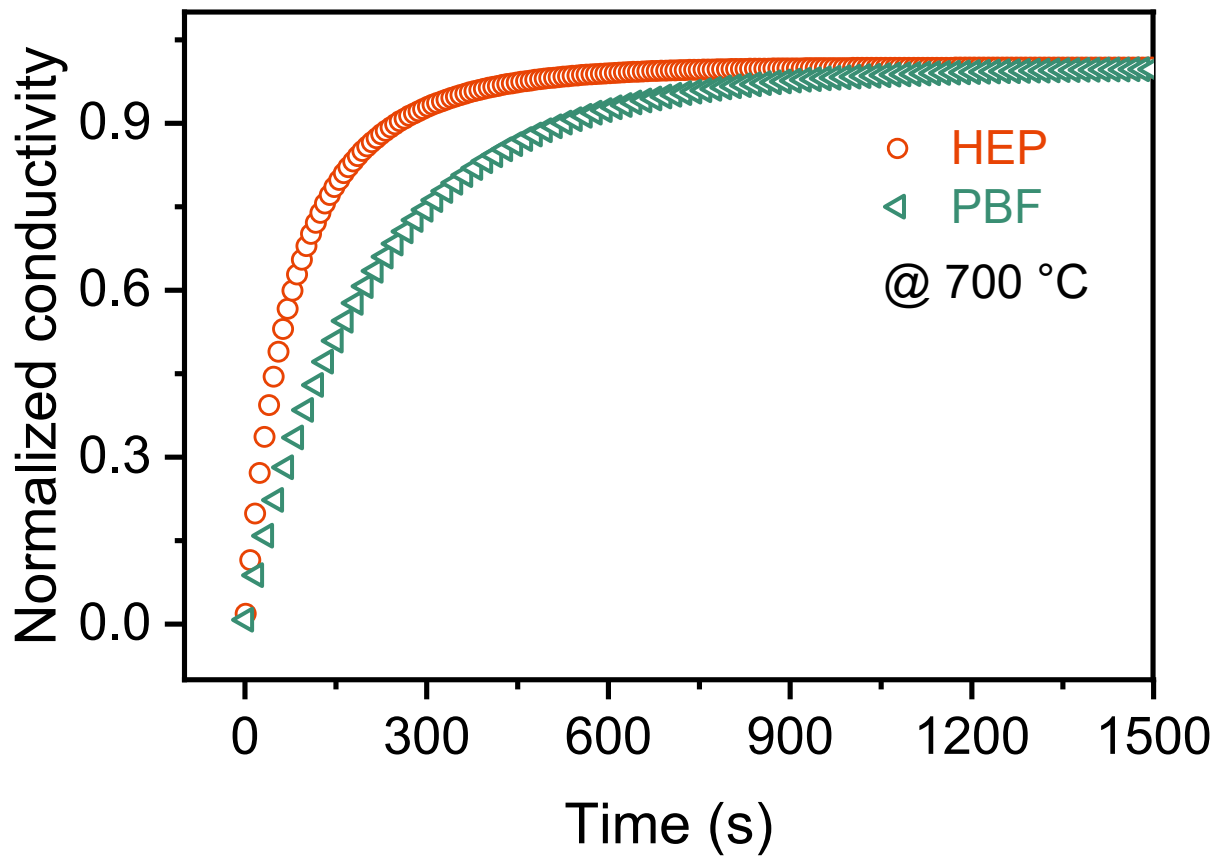

Fig. S20. Electrical conductivity relaxation curves of HEP and PBF samples at 700 ℃.

Source data for **Fig. S20** are provided as a Source Data file.

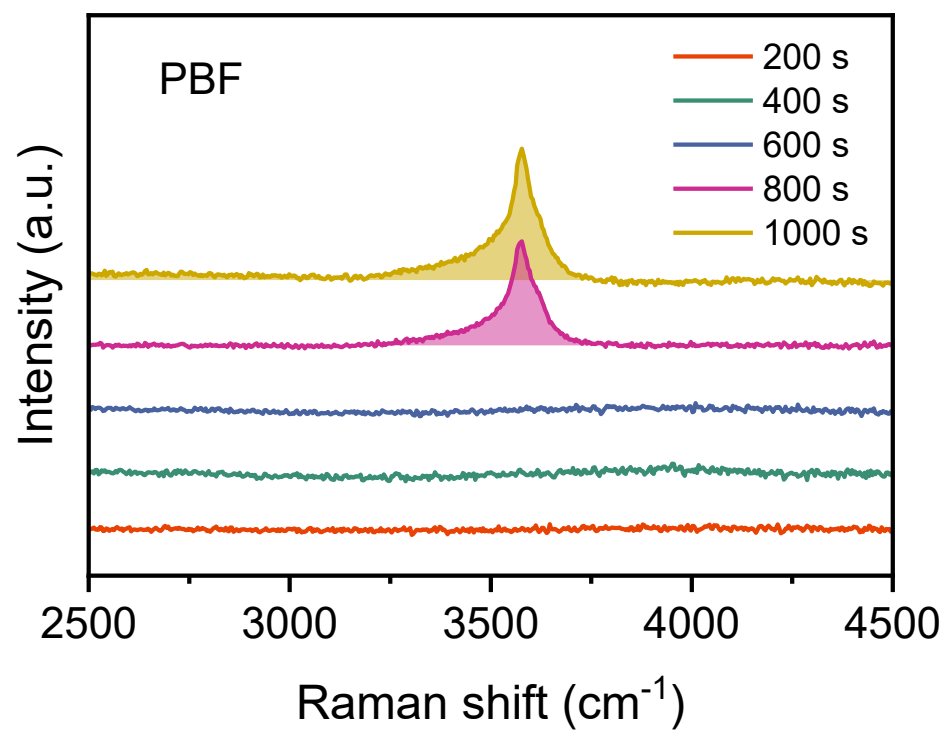

Fig. S21. Time-resolved Raman analysis of surface functional groups on PBF, during the exposure to wet N_2 (3% H_2O) at 500 °C (flow rate of N_2: 50 ml min^{-1}). Source data for **Fig. S21** are provided as a Source Data file.

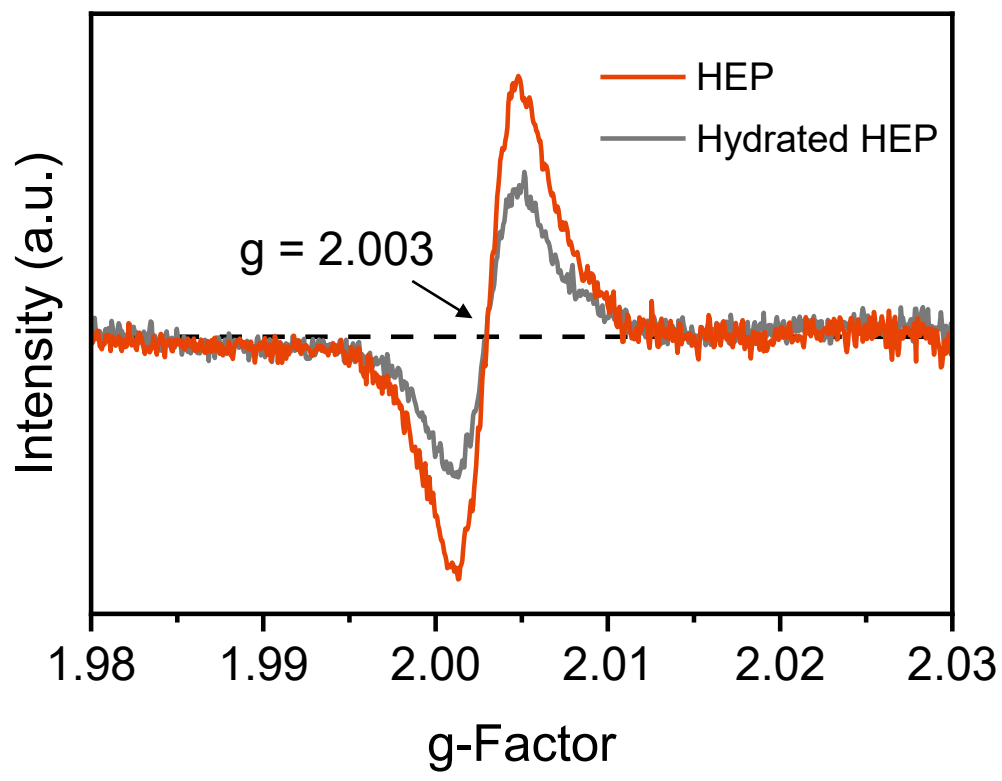

Fig. S22. EPR results of HEP and hydrated HEP. Source data for **Fig. S22** are provided as a Source Data file.

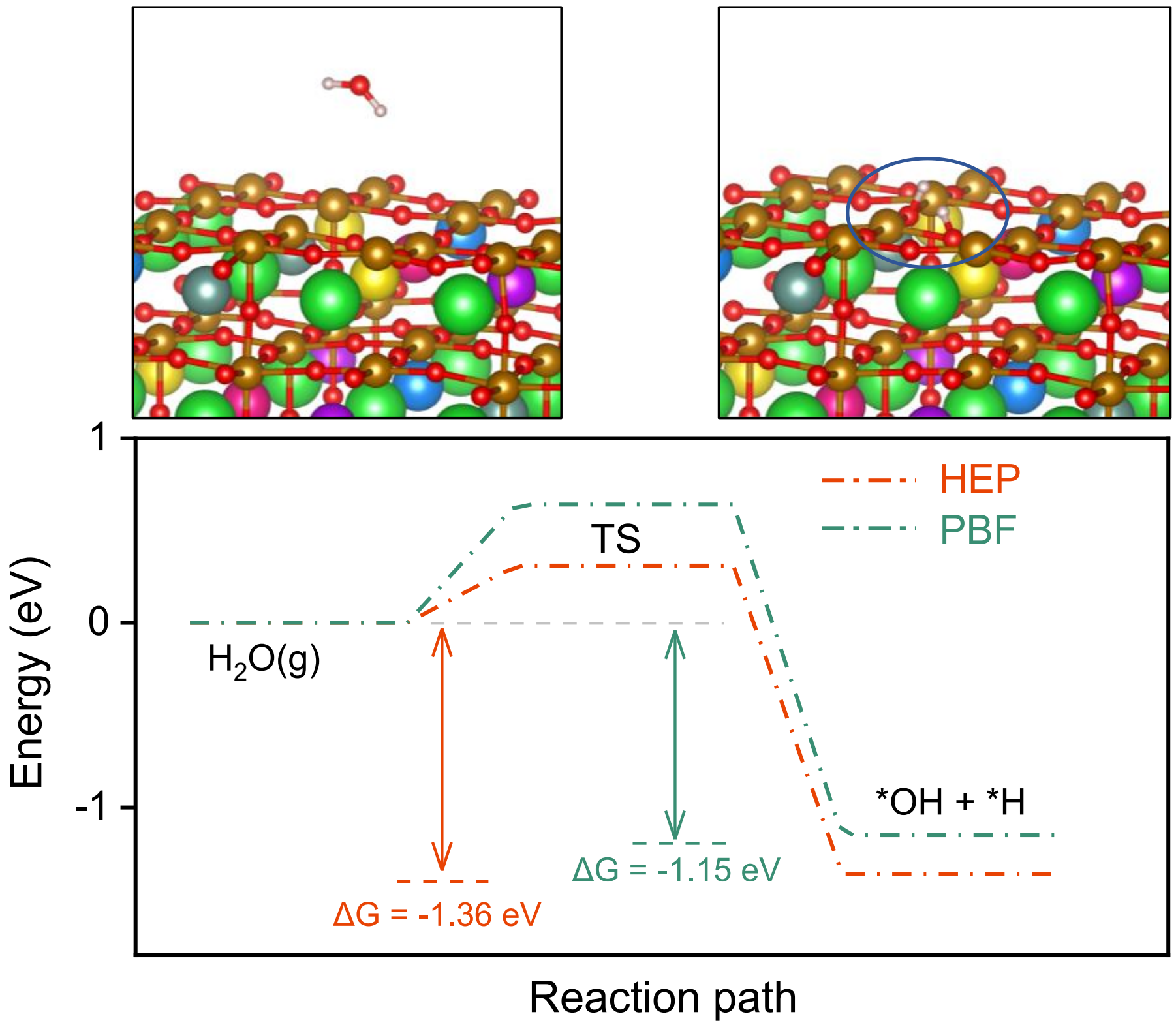

Fig. S23. Dissociative adsorption of H_2O on PBF and HEP surface. In the structural model, red, white, brown, purple, yellow, green, gray, pink, and blue spheres represent oxygen, hydrogen, iron, gadolinium, praseodymium, barium, yttrium, samarium, and lanthanum atoms, respectively.

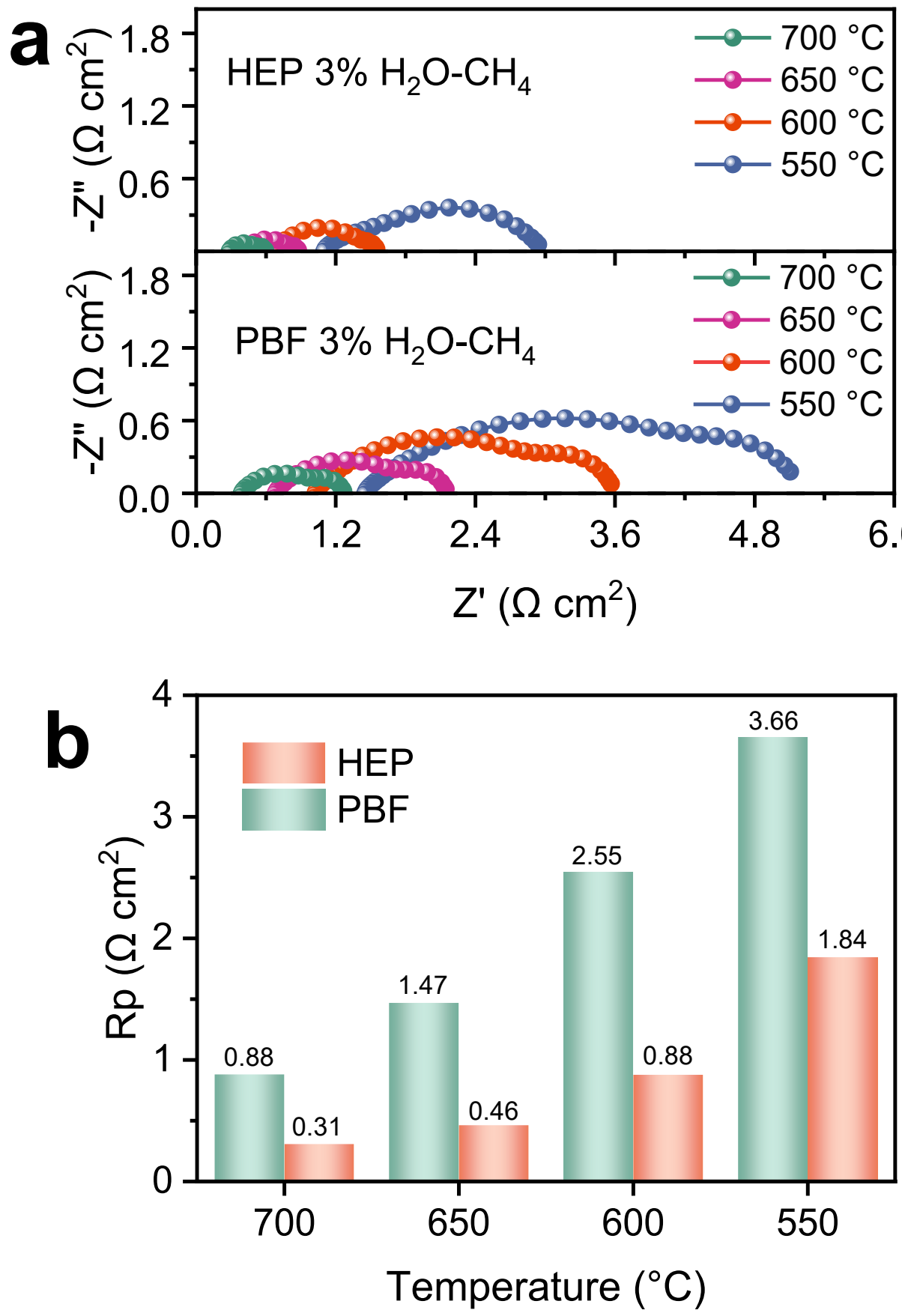

Fig. S24. a Electrochemical impedance of HEP and PBF anodes in 3% H_2O-CH_4. **b** Comparison of EIS at different temperatures. The flow rate of CH_4 is 20 ml min^{-1}. Source data for **Fig. S24** are provided as a Source Data file.

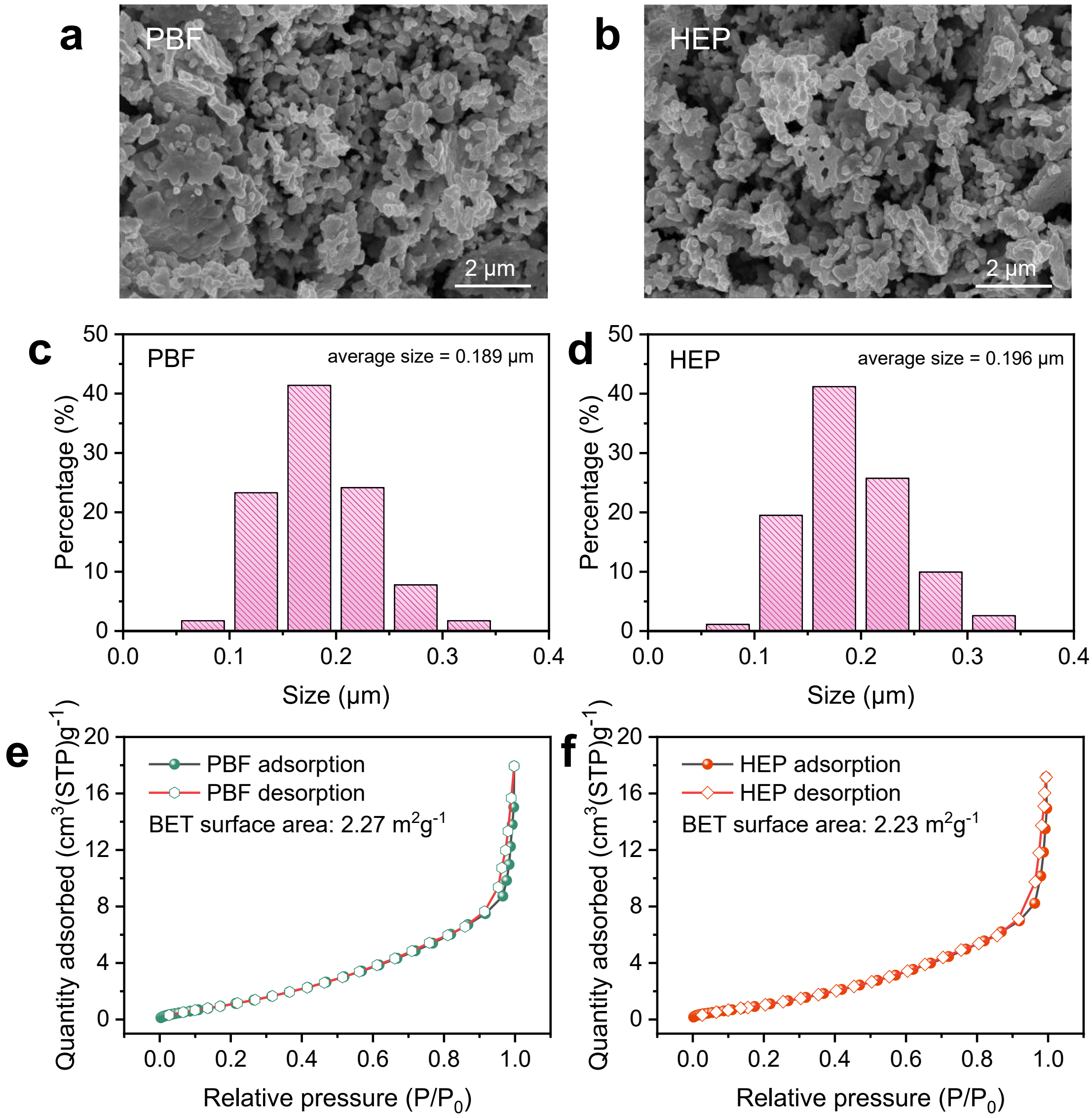

Fig. S25. SEM images of the as-prepared (**a**) PBF and (**b**) HEP. The corresponding size distribution of (**c**) PBF and (**d**) HEP. The nitrogen adsorption-desorption isotherm plots and Brunauer-Emmett-Teller (BET) surface area of (**e**) PBF and (**f**) HEP. Source data for **Fig. S25** are provided as a Source Data file.

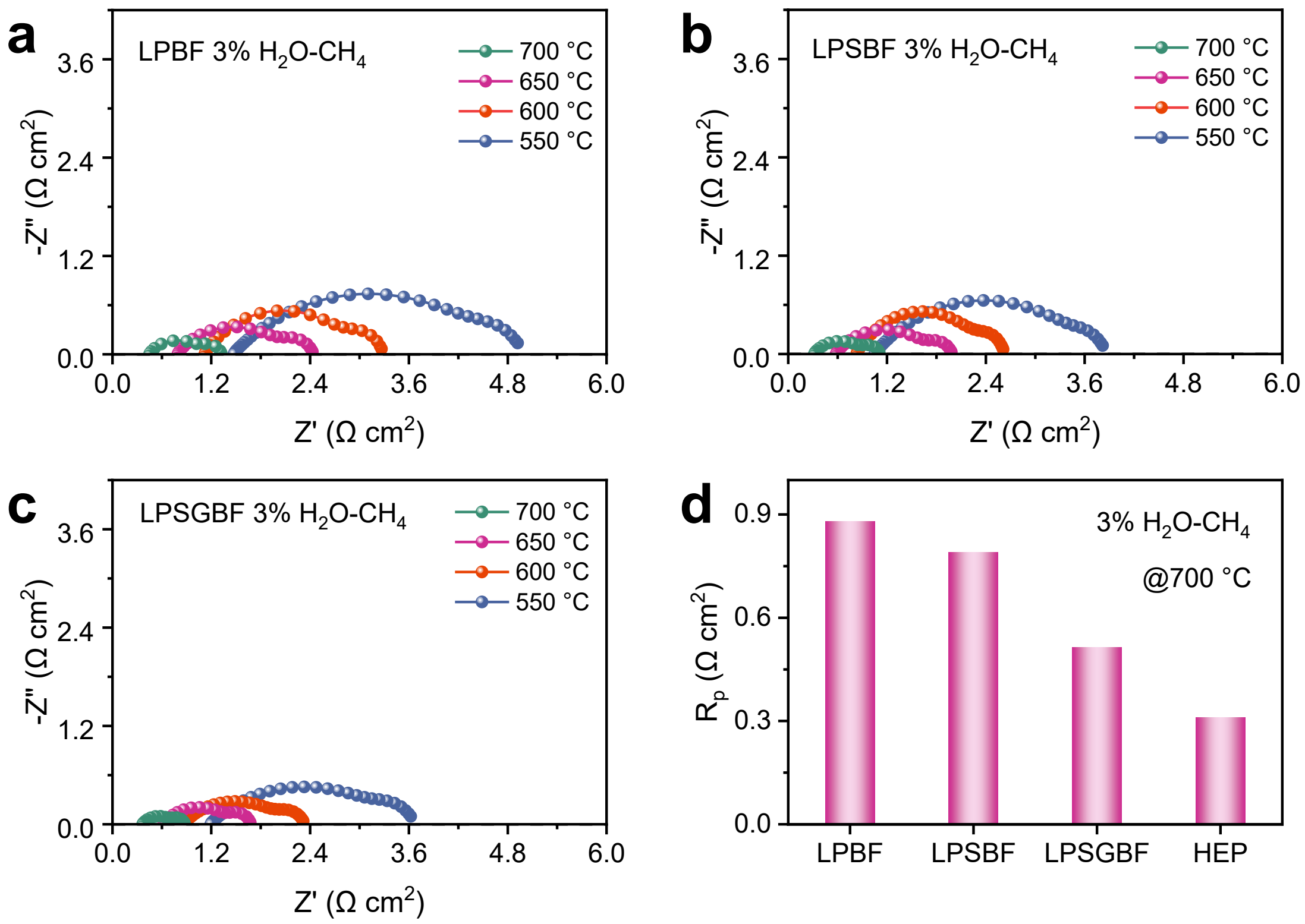

Fig. S26. EIS plots of anodes under 3% H_2O-CH_4 at 700 °C for (**a**) LPBF, (**b**) LPSBF and (**c**) LPSGBF. **d** The corresponding comparison of EIS at 700 °C. Source data for **Fig. S26** are provided as a Source Data file.

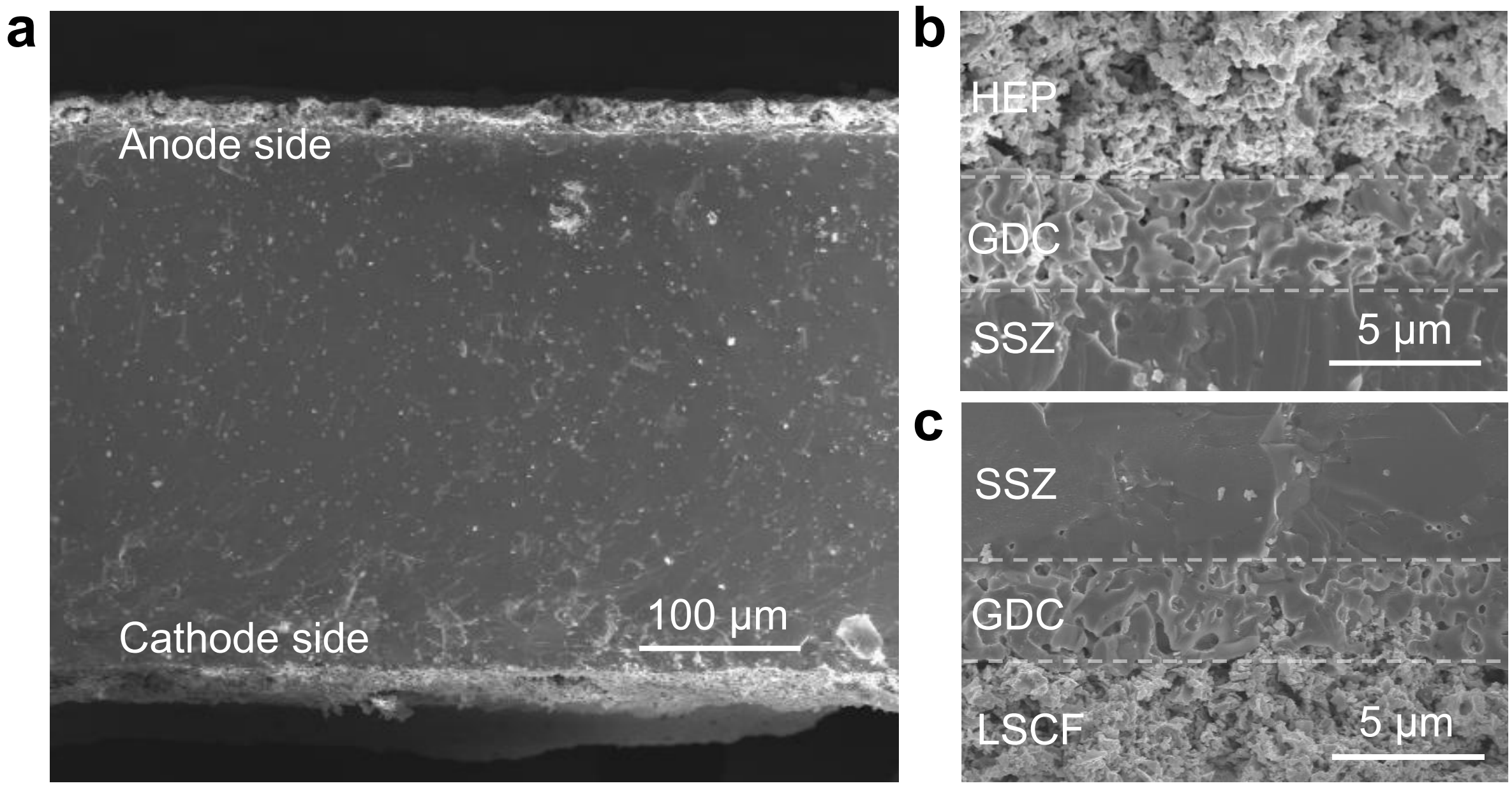

Fig. S27. a Cross-Wsectional view of the SSZ-based single cell with a configuration of HEP|GDC|SSZ|GDC|LSCF. **b** Magnified view of the anode side (HEP|GDC|SSZ). **c** Magnified view of the cathode side (SSZ|GDC|LSCF).

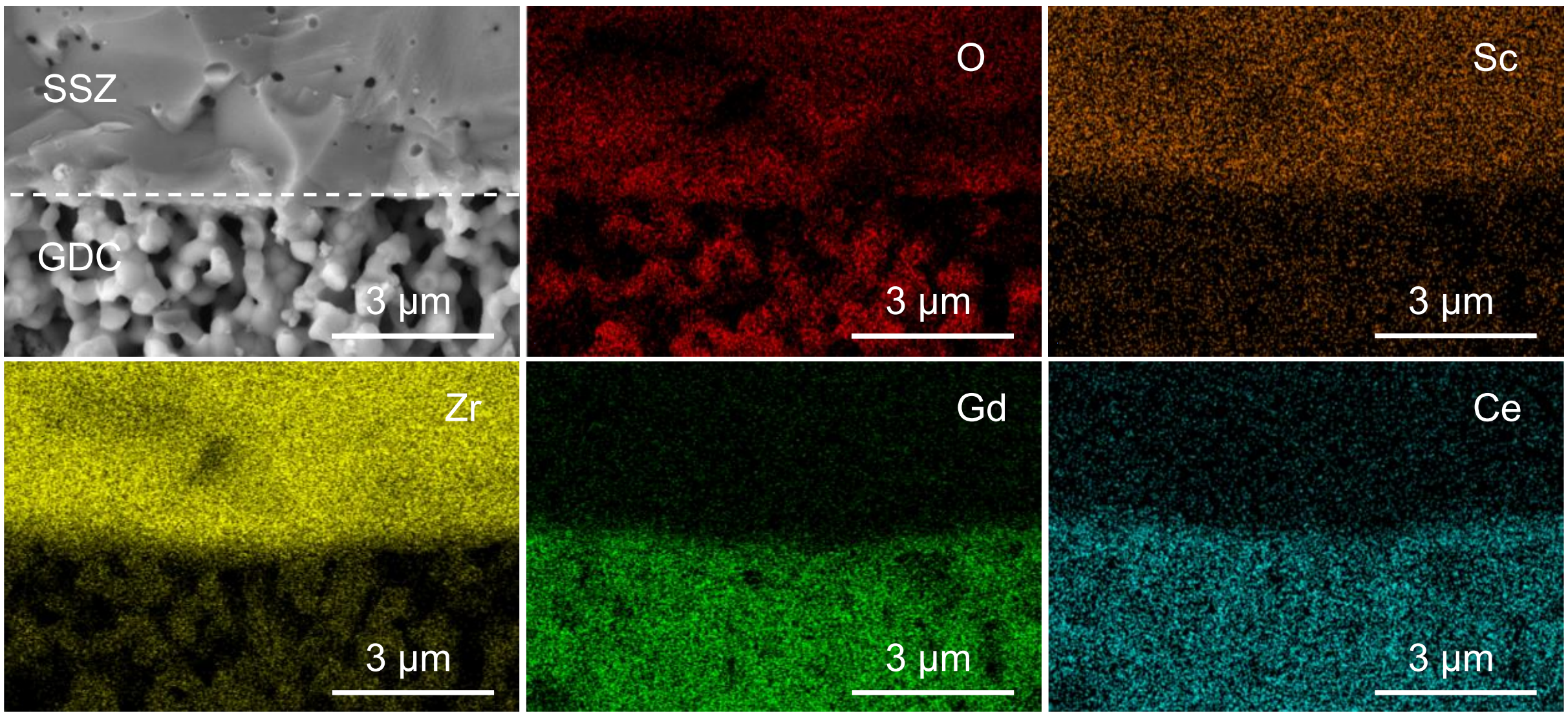

Fig. S28. EDS mapping images of SSZ|GDC interface. EDS results confirm the diffusion of Zr and Ce elements at the interface, which induces the formation of a (Zr, Ce)O_2 solid solution interlayer between SSZ and GDC. Notably, relevant literature[26] indicates that such an interlayer is not necessarily detrimental to cell performance; instead, a thin, continuous (Zr, Ce)O_2 solid solution interlayer can enhance electrolyte-electrode electrochemical activity, interfacial compatibility and adhesion, while potentially reducing polarization resistance compared to a sharp, reactive interface. In this study, the formed (Zr, Ce)O_2 interlayer is dense and well-bonded, contributing to the stable electrochemical performance of the cell during long-term testing.

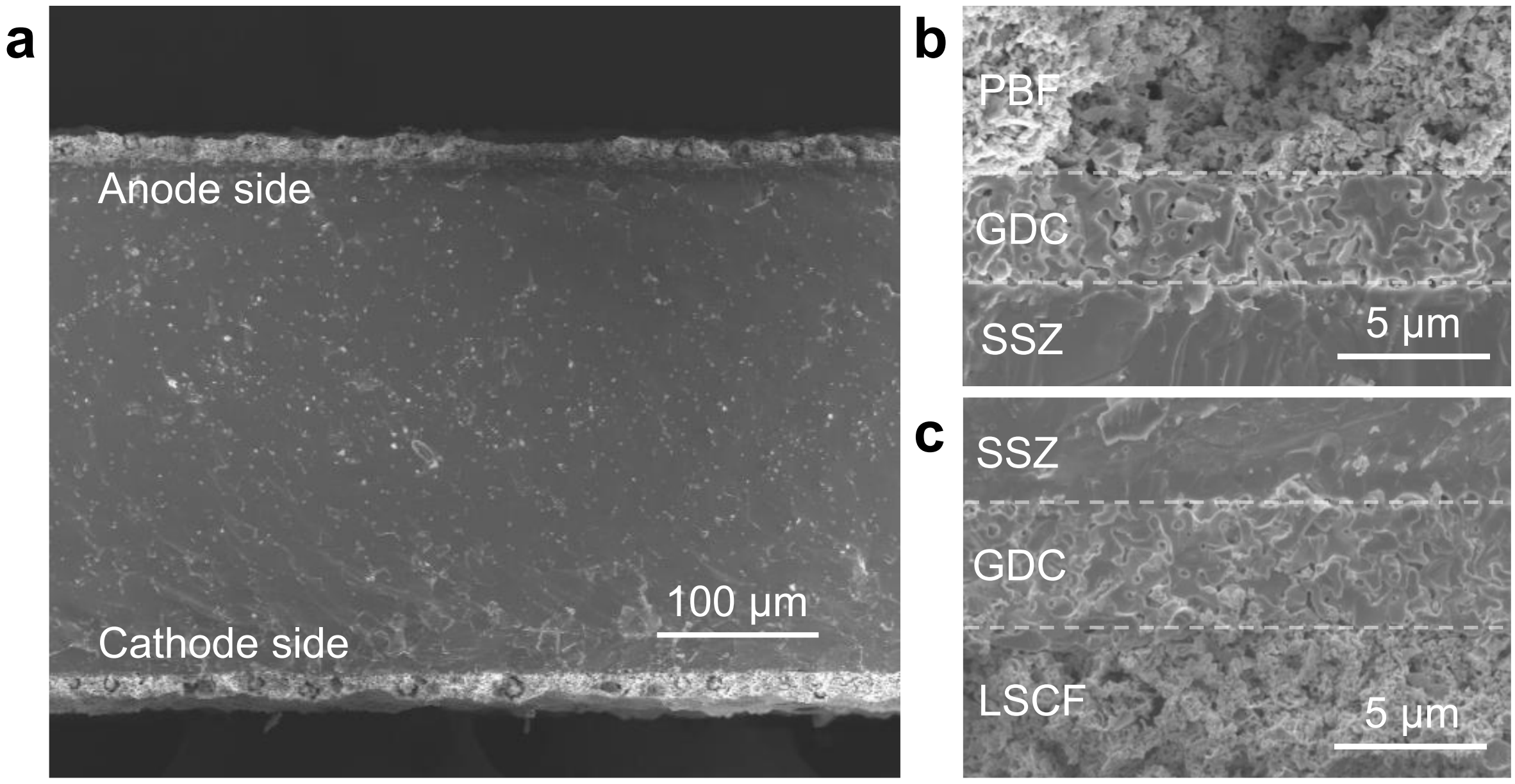

Fig. S29. a Cross-Wsectional view of the SSZ-based single cell with a configuration of PBF|GDC|SSZ|GDC|LSCF. **b** Magnified view of the anode side (PBF|GDC|SSZ). **c** Magnified view of the cathode side (SSZ|GDC|LSCF)

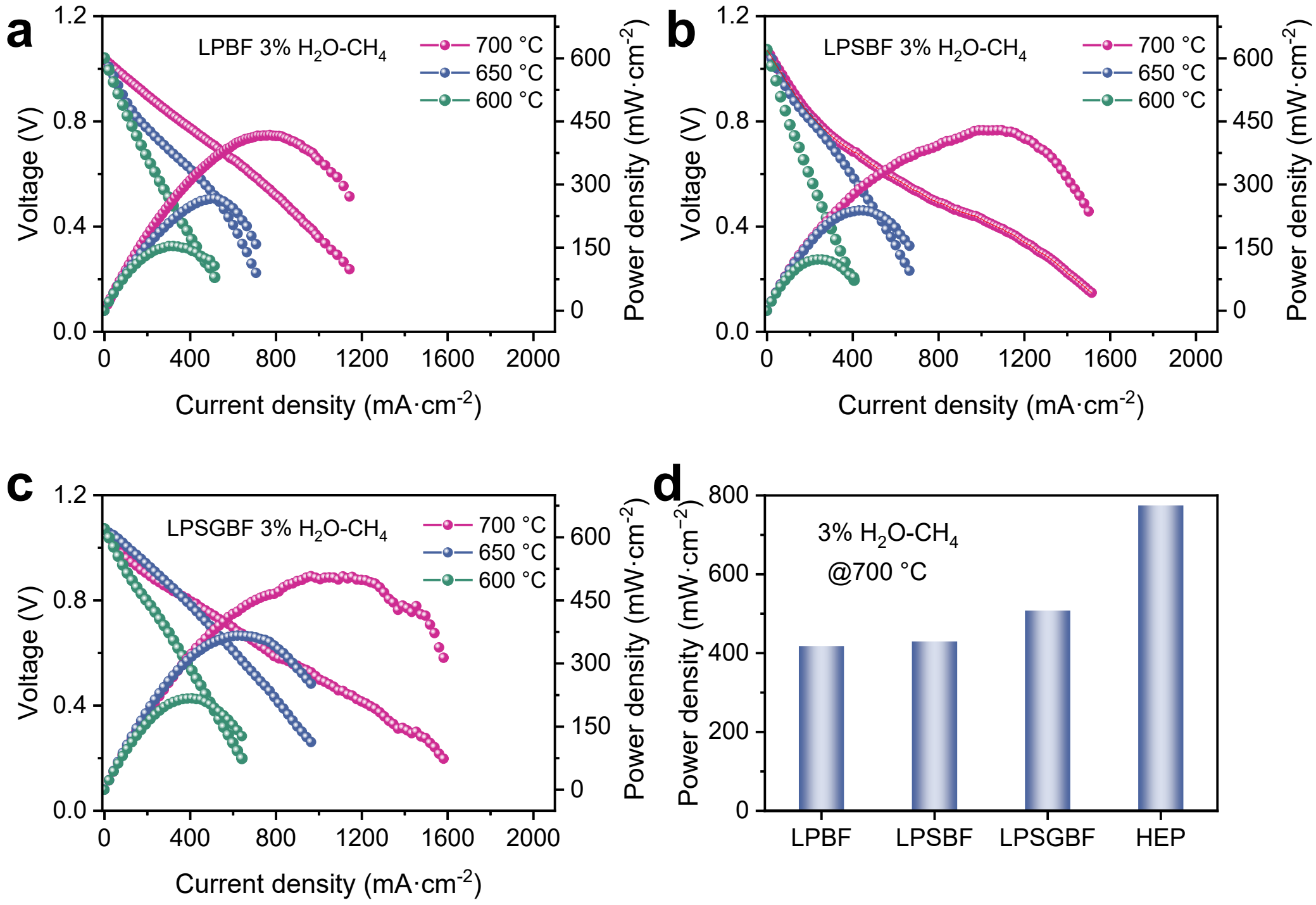

Fig. S30. I−V−P curves of single cells using (**a**) LPBF, (**b**) LPSBF and (**c**) LPSGBF as the anode in humidified CH_4 (3 vol% H_2O). The flow rate of CH_4 is 20 ml min^{-1}. **d** The corresponding comparison of electrochemical performance at 700 °C. Source data for **Fig. S30** are provided as a Source Data file.

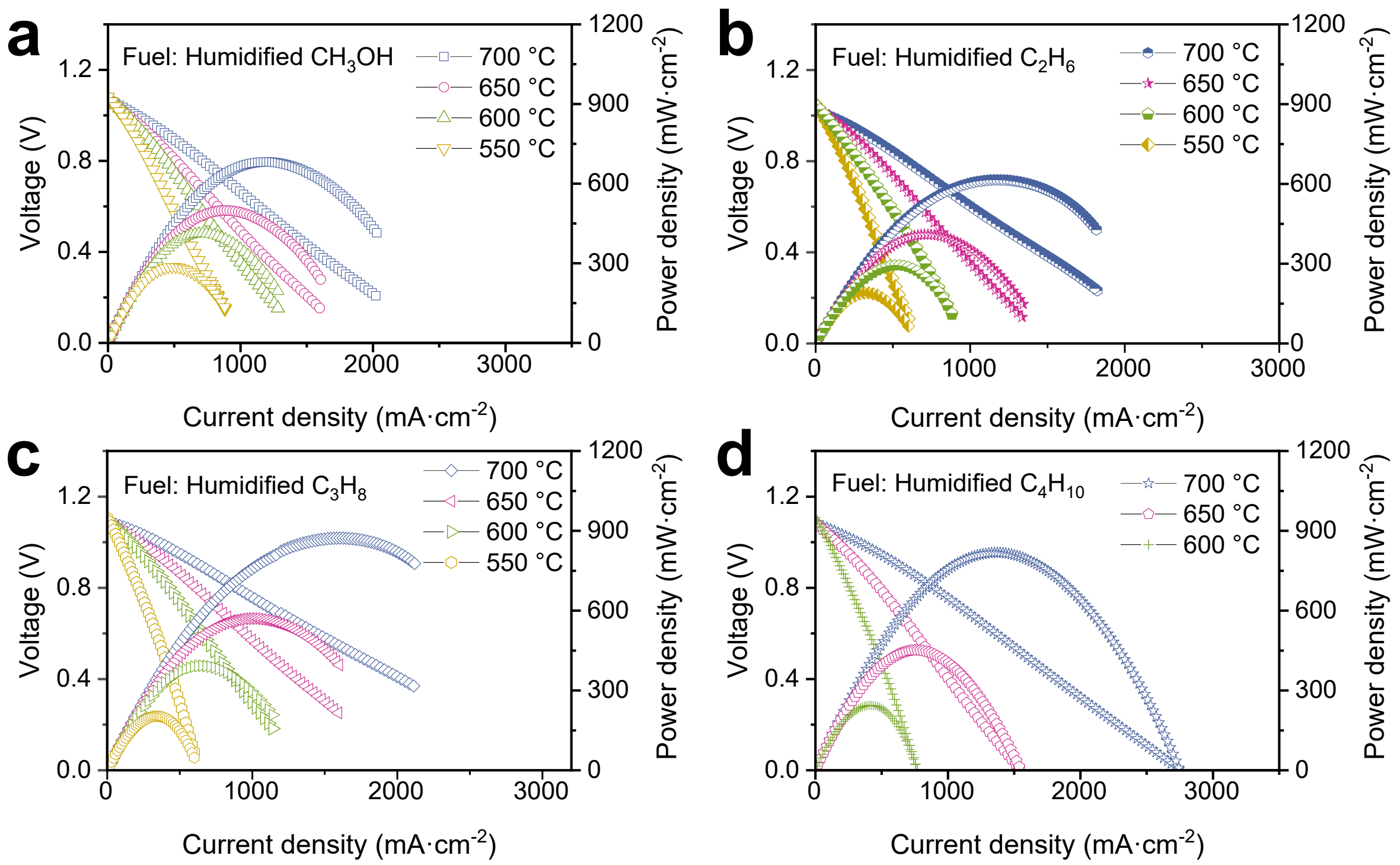

Fig. S31. I-V-P curves of HEP-based button cell with a configuration of HEP|GDC|SSZ|GDC|LSCF operated with various wet fuel, 50 ml min^{-1} Ar was used as the carrier gas. (**a**) 97 vol% CH_3OH + 3 vol% H_2O. The flow rate of CH_3OH was 20 ml min^{-1}. (**b**) 95 vol% C_2H_6 + 5 vol% H_2O. The flow rate of C_2H_6 was 14 ml min^{-1}. (**c**) 90 vol% C_3H_8 + 10 vol% H_2O. The flow rate of C_3H_8 was 10 ml min^{-1}. (**d**) 85 vol% n-C_4H_{10} + 15 vol% H_2O. The flow rate of C_4H_{10} was 5 ml min^{-1}. Source data for **Fig. S31** are provided as a Source Data file.

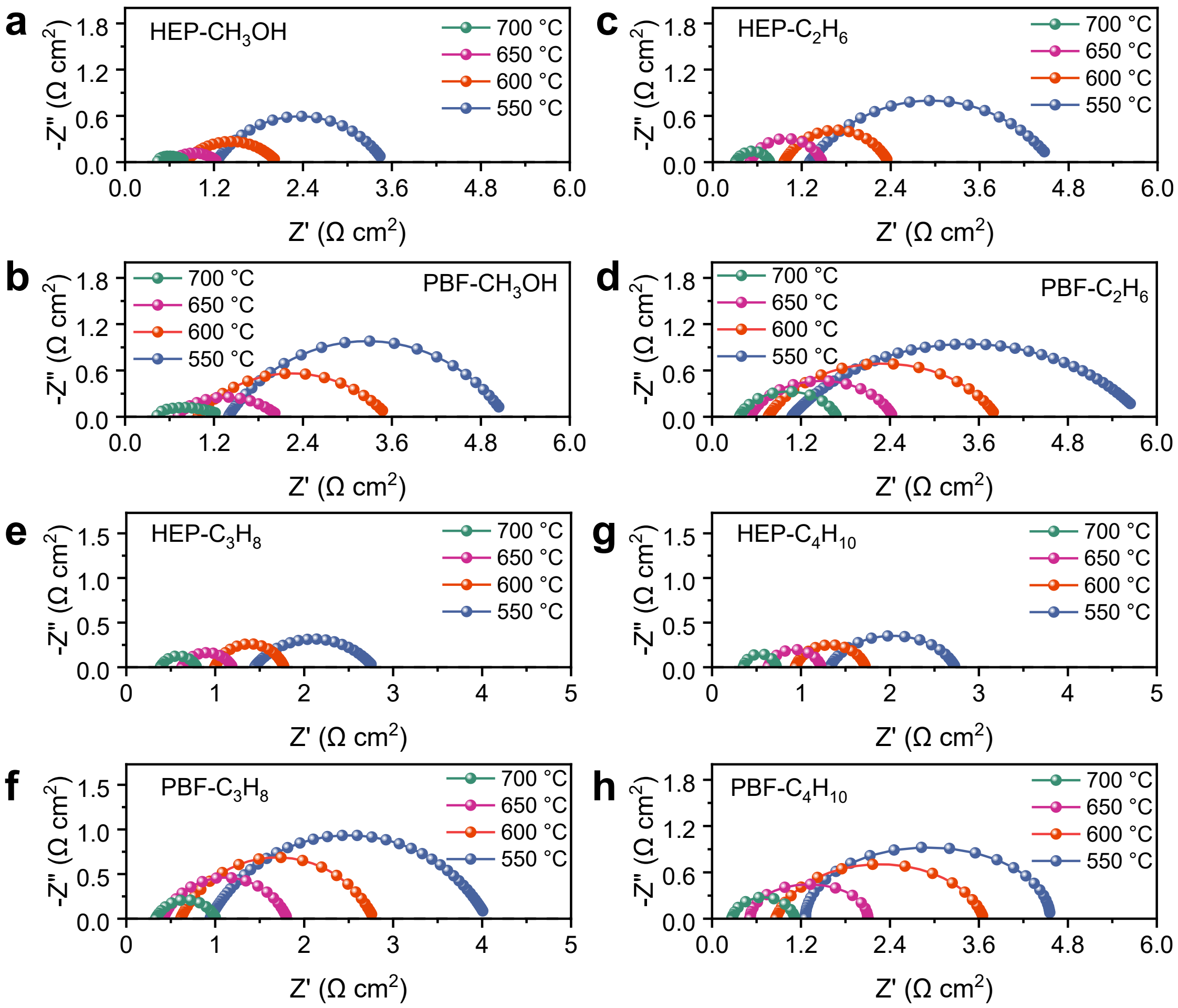

Fig. S32. EIS plots of HEP and PBF symmetrical cells under different atmosphere. (**a**) and (**b**) for 3% H_2O-CH_3OH. (**c**) and (**d**) for 5% H_2O-C_2H_6. (**e**) and (**f**) for 10% H_2O-C_3H_8. (**g**) and (**h**) for 15% H_2O/n-C_4H_{10}. Source data for **Fig. S32** are provided as a Source Data file.

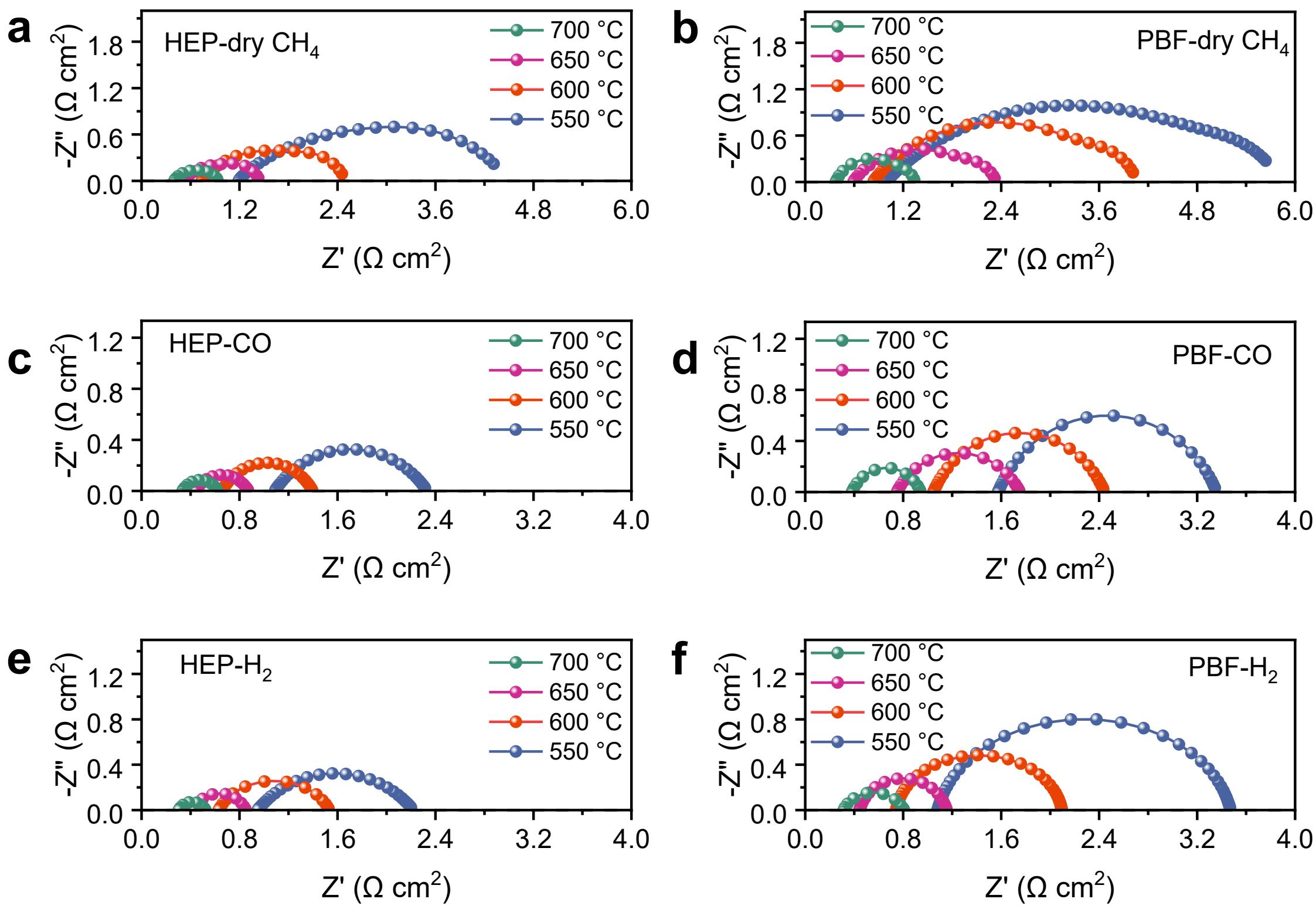

Fig. S33. EIS plots of HEP and PBF symmetrical cells under different atmosphere. (**a**) and (**b**) for dry CH_4. (**c**) and (**d**) for CO. (**e**) and (**f**) for H_2. Source data for **Fig. S33** are provided as a Source Data file.

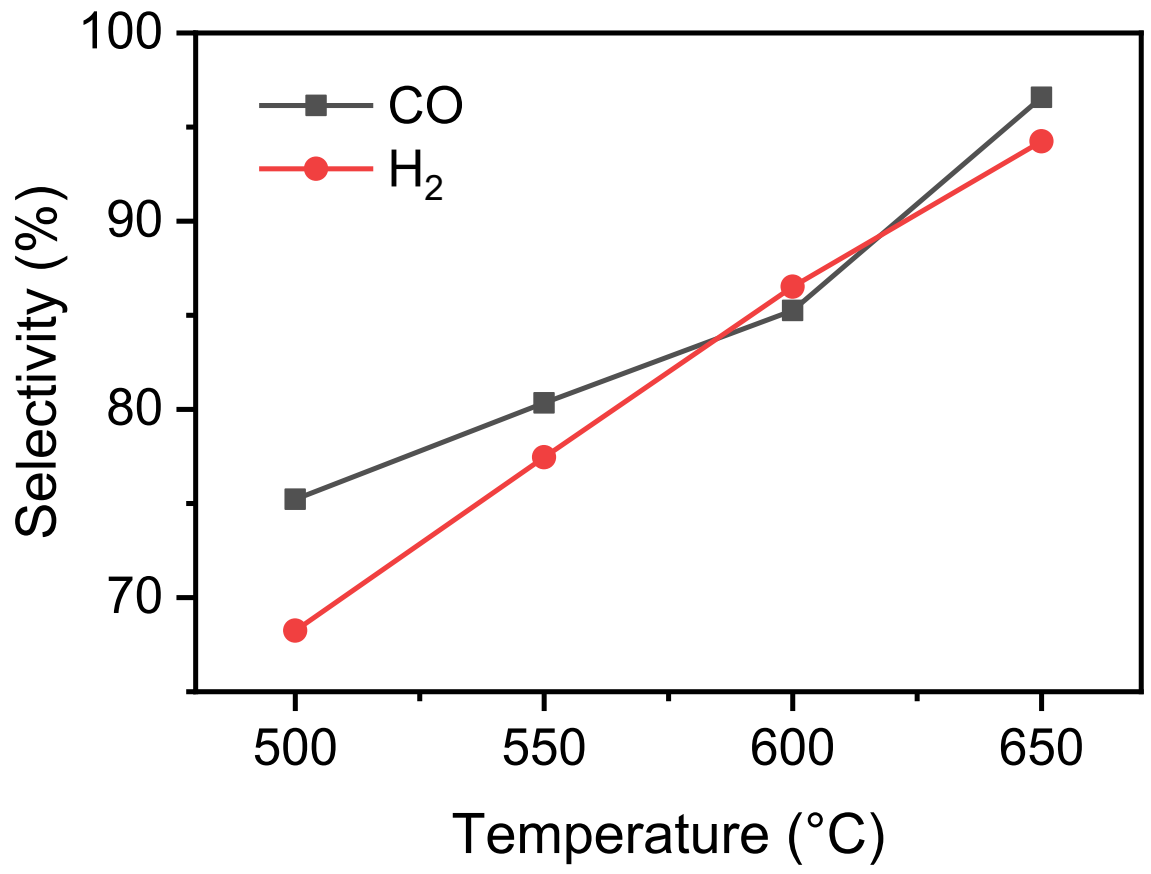

Fig. S34. Selectivity for H_2 and CO as a function of catalysis temperature for steam reforming on HEP. Source data for **Fig. S34** are provided as a Source Data file.

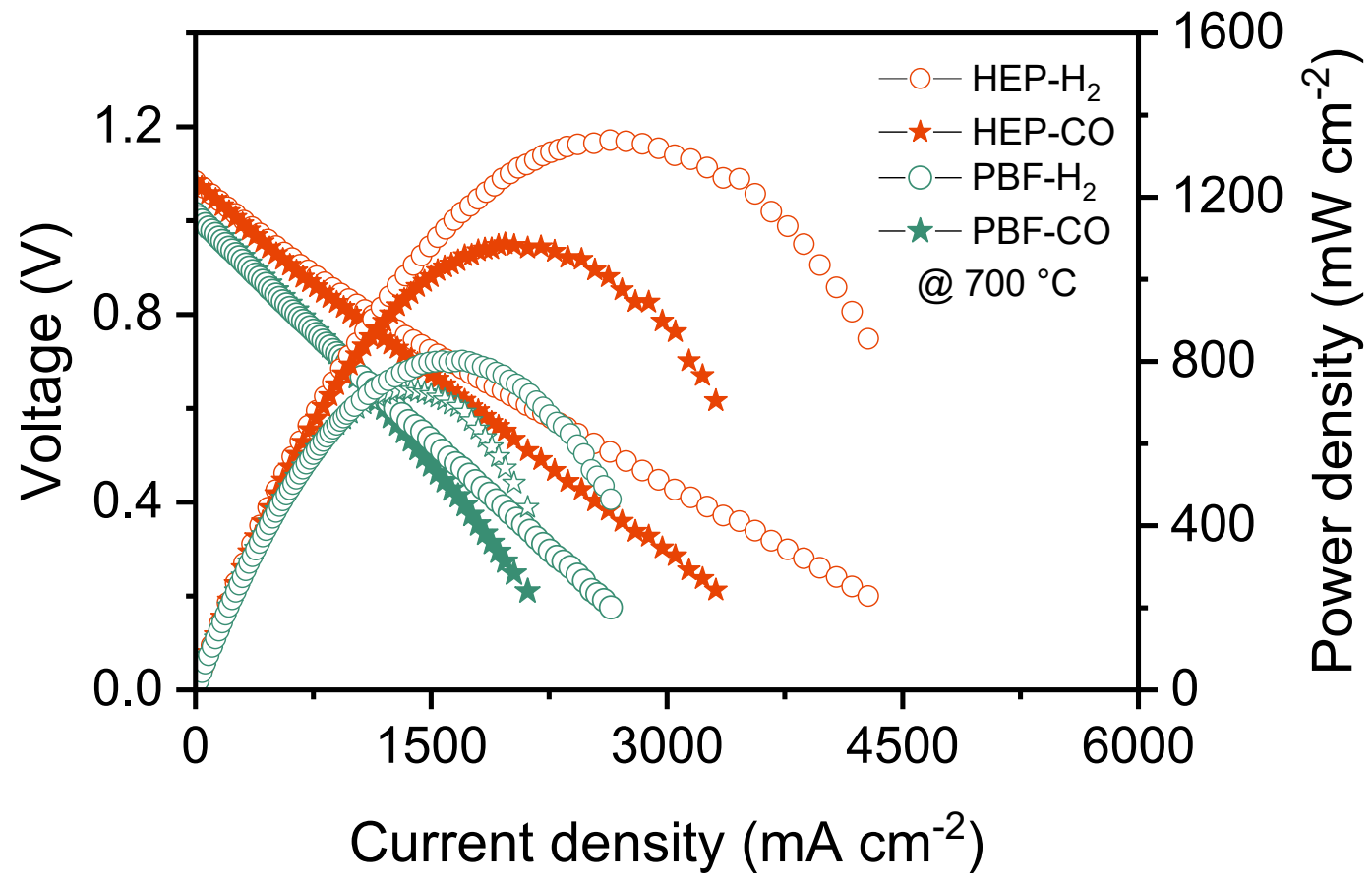

Fig. S35. I−V−P curves of single cells using HEP and PBF as the anode under H_2 and CO fuel at 700 °C. The flow rates of H_2 and CO are each 20 ml min^{-1}. Source data for **Fig. S35** are provided as a Source Data file.

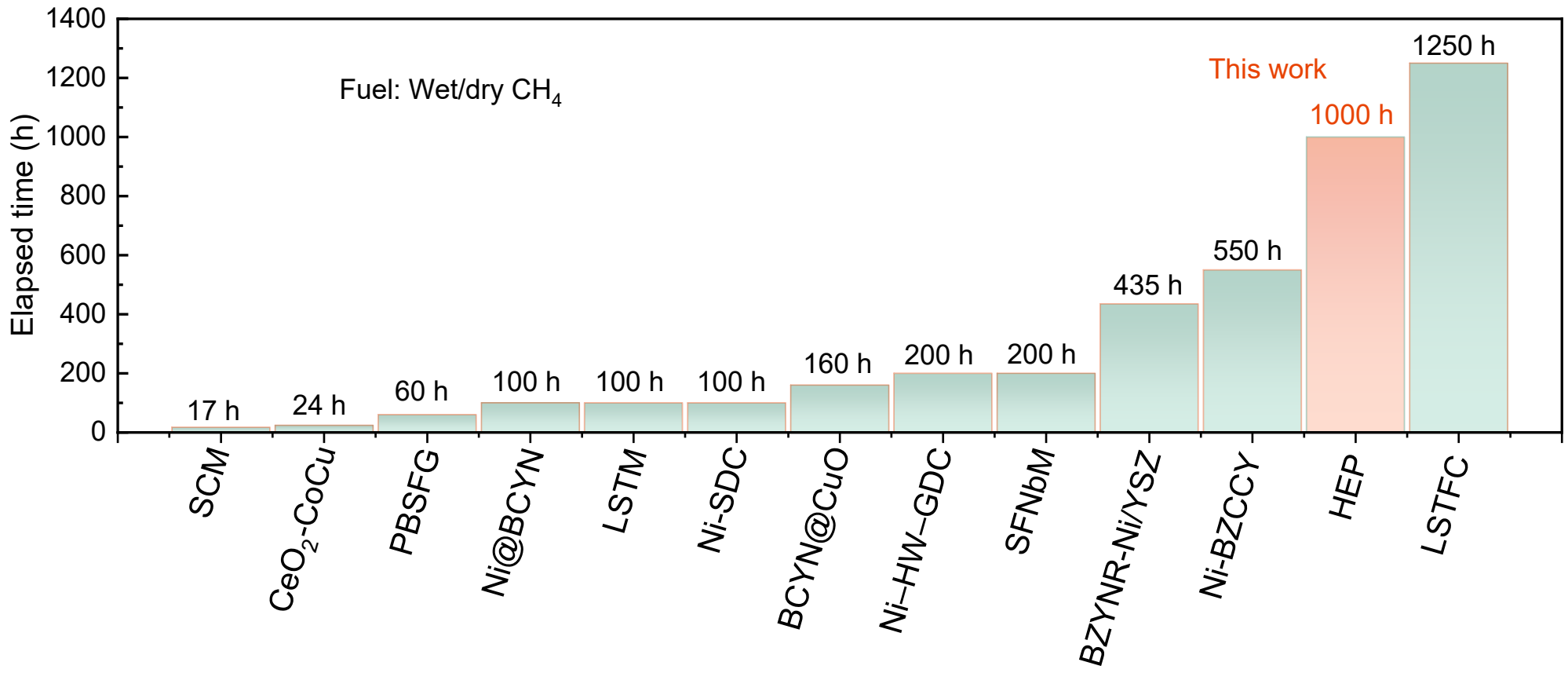

Fig. S36. Comparison of the stability of CH_4-SOFCs (this work) with the literature results.

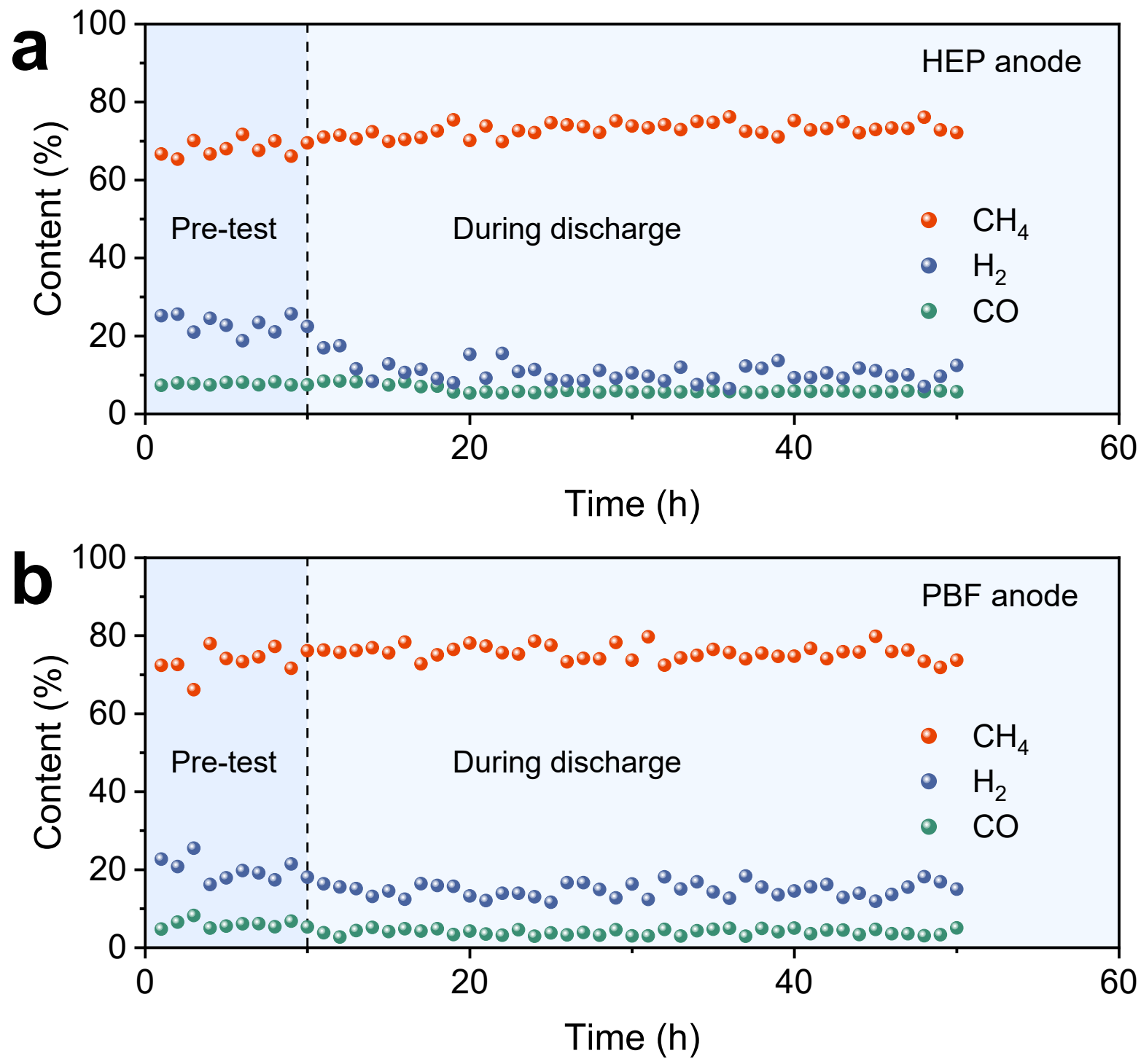

Fig. S37. Different gas component contents during stability testing process for (a) HEP and (b) PBF. Source data for **Fig. S37** are provided as a Source Data file.

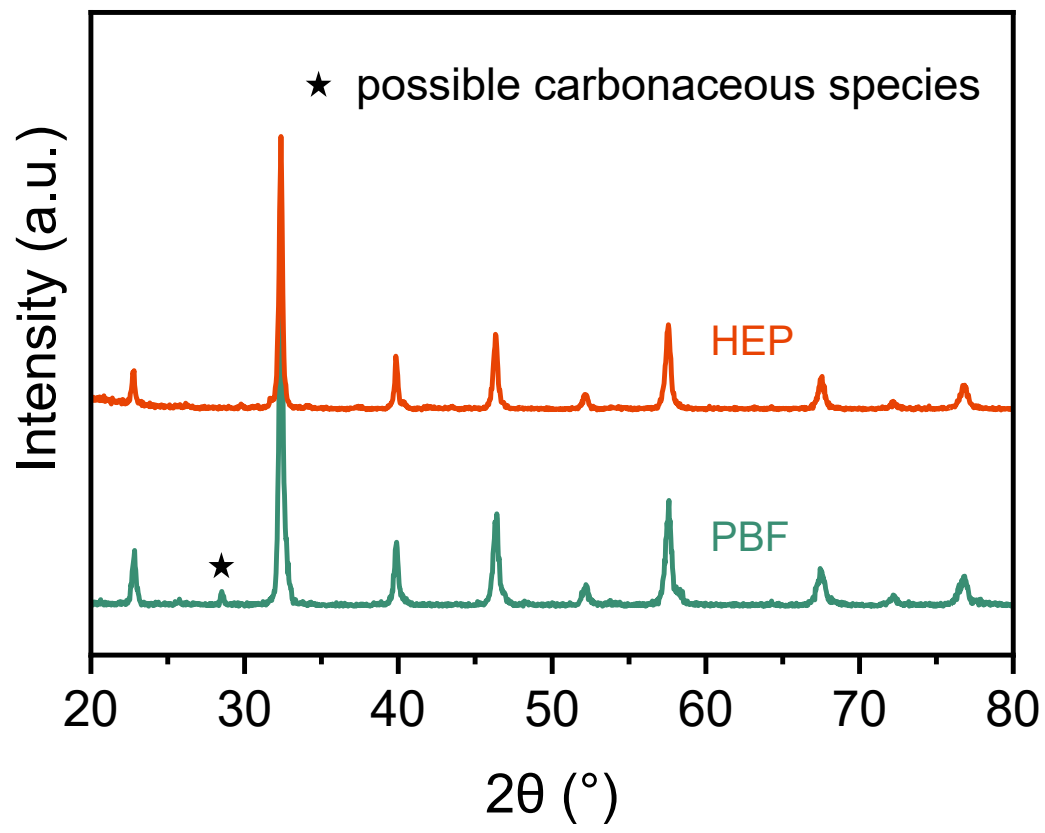

Fig. S38. XRD patterns of HEP sample after long-term stability test. Source data for **Fig. S38** are provided as a Source Data file.

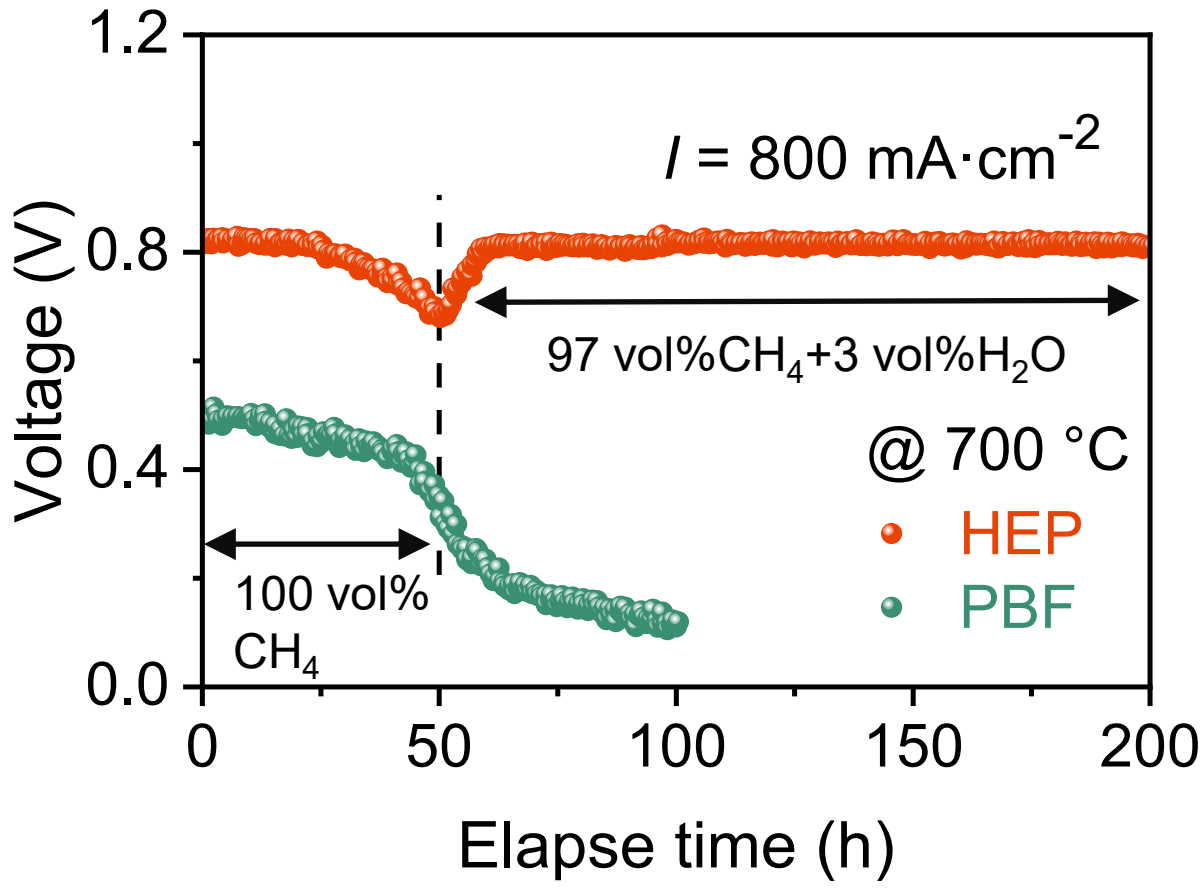

Fig. S39. Durability test of single cell with HEP (red) and PBF (green) as the anode, when fueled with dry methane or wet methane (with ~3% H_2O) under current of 800 mA cm^{-2} at 700 °C. The flow rate of CH_4 is 20 ml min^{-1}. Source data for **Fig. S39** are provided as a Source Data file.

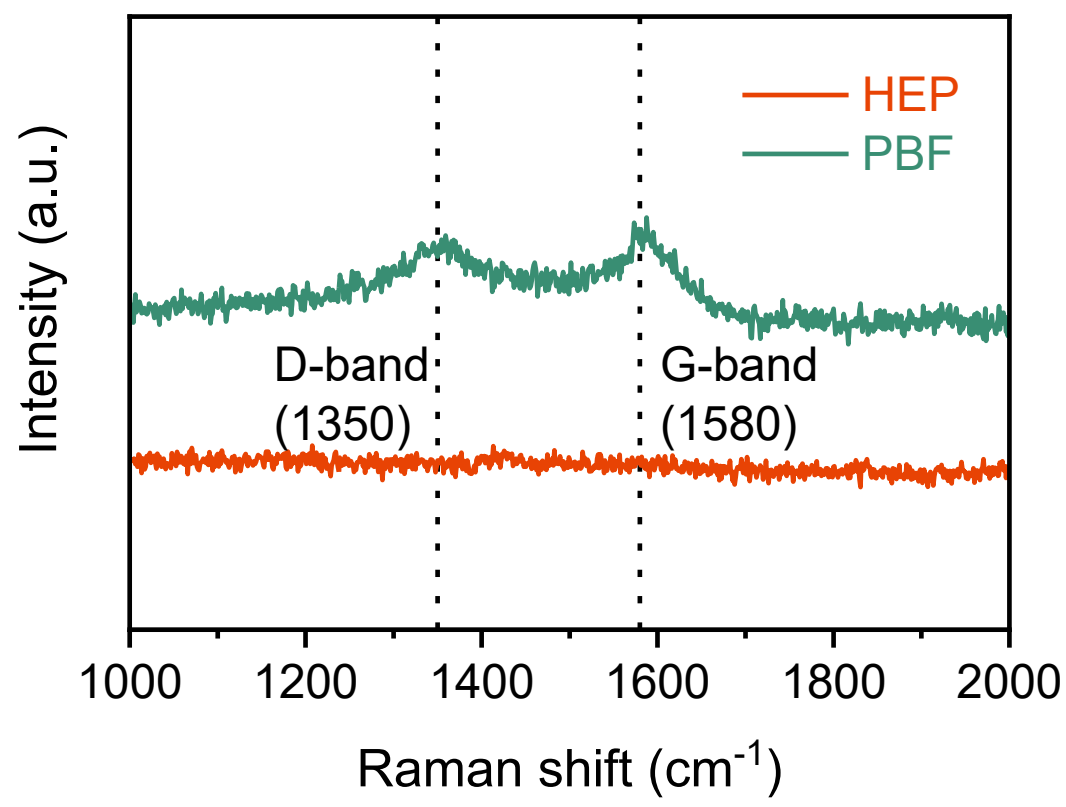

Fig. S40. Raman spectra of the HEP and PBF anode after the durability test for 200 h. Source data for **Fig. S40** are provided as a Source Data file.

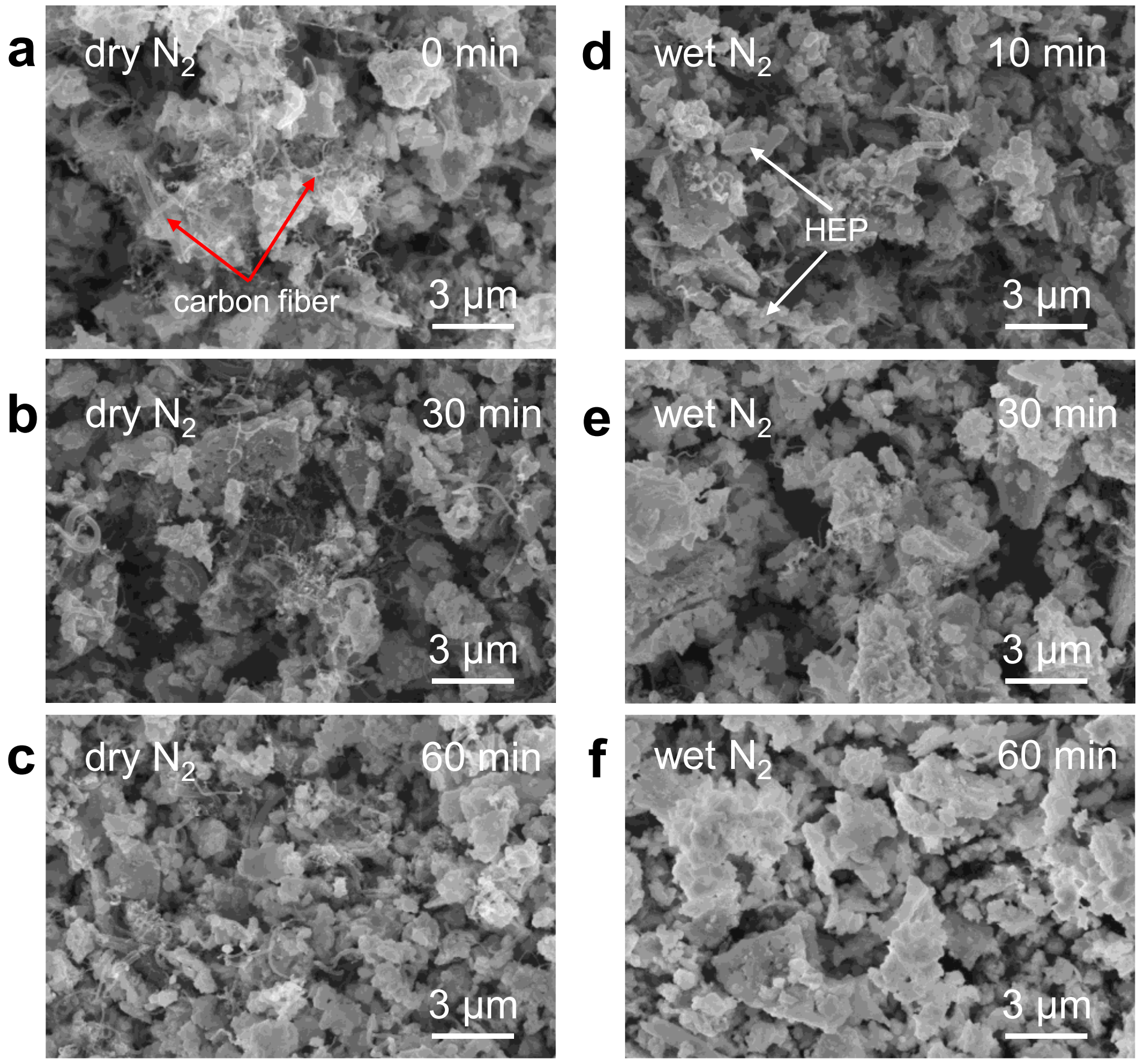

Fig. S41. SEM images of carbon-contaminated HEP anode during the Raman test under different N_2 atmospheres and durations. (**a-c**) Samples treated in dry N_2 for 0 min (**a**), 30 min (**a**) and 60 min (**c**). (**d-f**) Samples treated in wet N_2 for 10 min (**d**), 30 min (**e**), and 60 min (**f**).

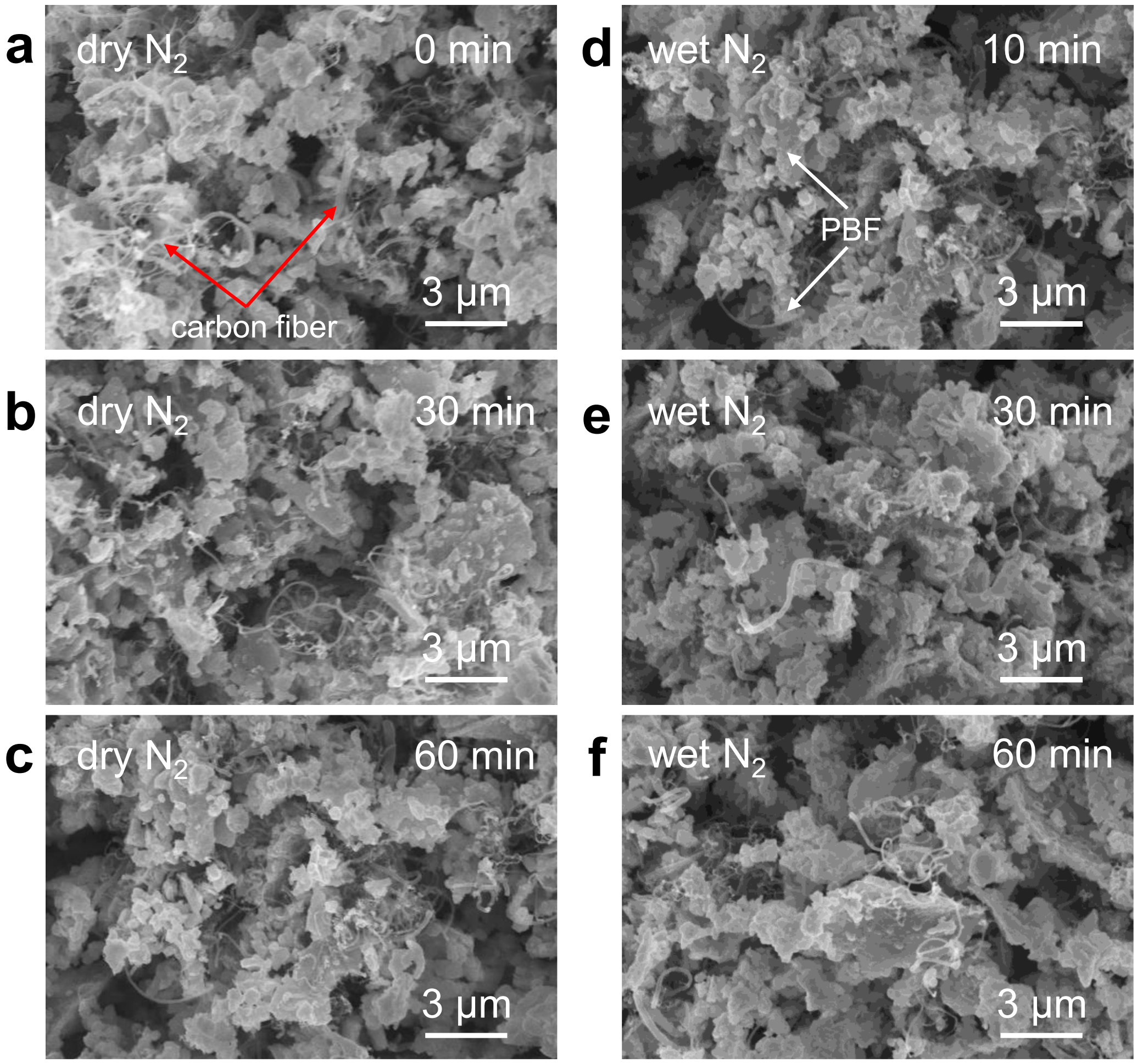

Fig. S42. SEM images of carbon-contaminated PBF anode during the Raman test under different N_2 atmospheres and durations. (**a**-**c**) Samples treated in dry N_2 for 0 min (**a**), 30 min (**a**) and 60 min (**c**). (**d**-**f**) Samples treated in wet N_2 for 10 min (**d**), 30 min (**e**), and 60 min (**f**).

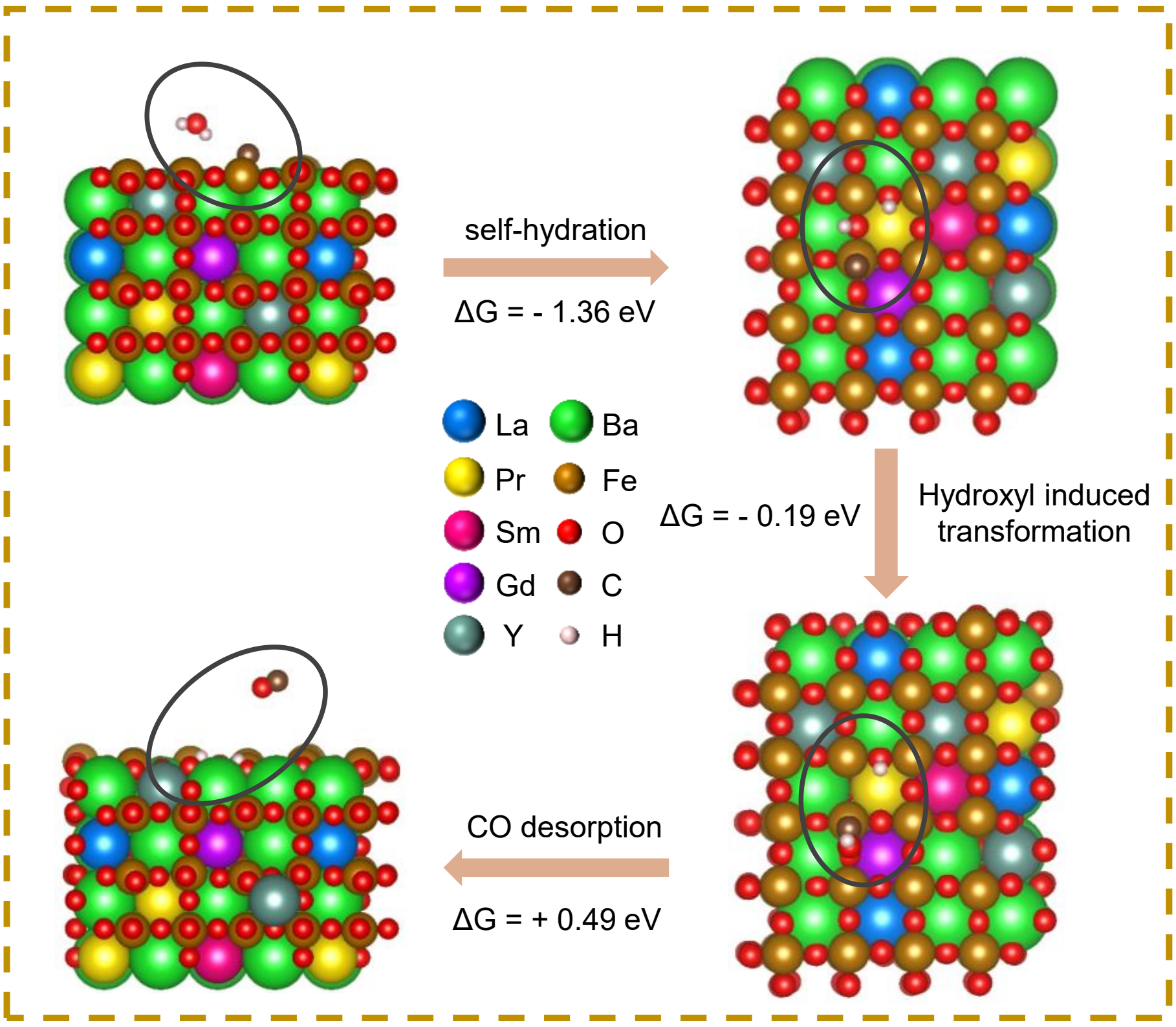

Fig. S43. Magnified view of the decarburization process on the HEP surface.

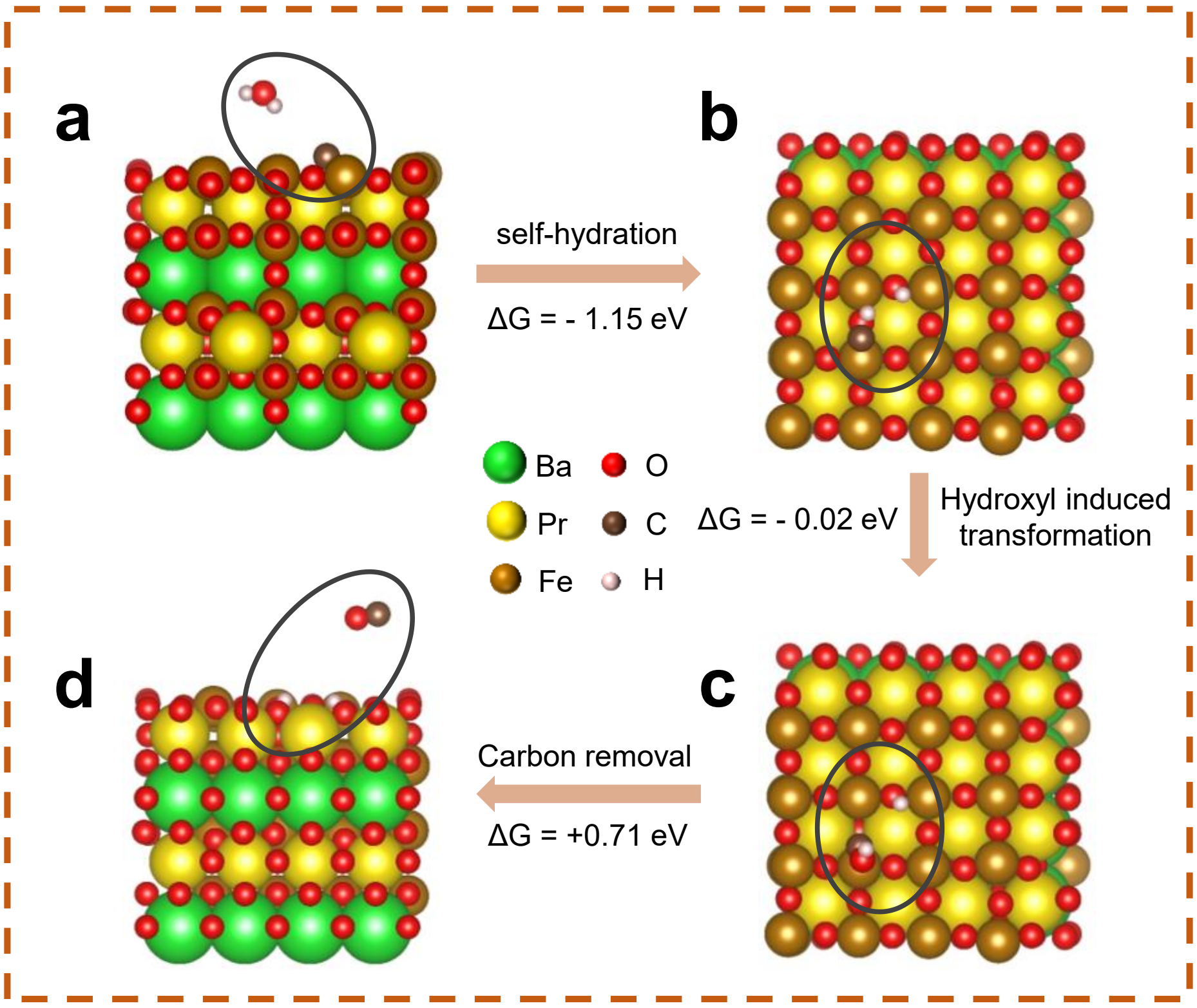

Fig. S44. Magnified view of the decarburization process on the PBF surface. **a** Adsorption of a water molecule on the catalyst surface. **b** Dissociation of the adsorbed water molecule to form surface hydroxyl groups (*OH) via self-hydration. **c** Reaction between the surface hydroxyl (*OH) and the deposited carbon to form the intermediate *COH. **d** Formation and desorption of carbon monoxide (CO) from the surface.

Table S1. Unit cell parameters of PBF after SXRPD refinement.

Space group	P4/mmm				
R_{wp} (%)	6.34				
R_p (%)	4.97				
χ^2	1.24				
a (Å)	b (Å)	c (Å)	alpha (°)	beta (°)	gamma (°)
3.93274	3.93274	7.91541	90	90	90
element	occupancy	x	y	z	uiso
Pr1	1	0	0	0.5	0.02425
O3	1	0.5	0.5	0.5	0.02363
Ba1	1	0	0	0	0.02252
O1	1	0.5	0.5	0	0.01937
Fe1	1	0.5	0.5	0.25	0.02019
O2	1	0.5	0	0.2993	0.02182

Table S2. Stoichiometry of the HEP compounds determined by inductively coupled plasma mass spectrometry.

	La	Pr	Sm	Gd	Y	Ba	Fe
HEP	0.205	0.203	0.195	0.192	0.205	1.006	2.000

Table S3. Unit cell parameters of HEP after SXRPD refinement.

Space group	$Pm\bar{3}m$				
R_{wp} (%)	7.45				
R_p (%)	5.32				
χ^2	1.31				
a (Å)	b (Å)	c (Å)	alpha (°)	beta (°)	gamma (°)
3.93332	3.93332	3.93332	90	90	90
element	occupancy	x	y	z	uiso
La	0.1	0	0	0	0.02127
Pr	0.1	0	0	0	0.02127
Sm	0.1	0	0	0	0.02127
Gd	0.1	0	0	0	0.02127
Y	0.1	0	0	0	0.02127
Ba	0.5	0	0	0	0.02127
Fe1	1	0.5	0.5	0.5	0.02671
O1	1	0.5	0.5	0	0.03641

Table S4. EXAFS fitting parameters at the Fe K-edge for PBF and HEP samples.

Sample	Path	Bond length (Å)	Coordination Number	σ^2 ($Å^2$)	E_0 shift (eV)	R-factor
PBF	Fe-O	2.04 ± 0.02	4.2 ± 0.9	0.007 ± 0.004	3.1 ± 0.7	0.009
HEP	Fe-O	2.06 ± 0.02	3.5 ± 0.8	0.010 ± 0.005	1.4 ± 2.1	0.014

Table S5. Performance and durability comparison of SOFC using methane as fuel in the range of 600-800 °C

Cell configuration	Electrolyte and thickness (μm)	Fuel mixture	Temperature (°C)	Peak power density (mW·cm^{-2})	Durability (h)	Ref.
$Ba(Ce_{0.9}Y_{0.1})_{0.8}Ni_{0.2}O_{3-\delta}$@CuO-GDC|GDC|LSCF	GDC (~20)	97% CH_4+3% H_2O	750	86	160	5
Ni@$Ba(Ce_{0.9}Y_{0.1})_{0.8}Ni_{0.2}O_{3-\delta}$-GDC|GDC|LSCF	GDC (—)	97% CH_4+3% H_2O	650<br>700<br>750	60<br>109<br>211	100<br>(750 °C)	6
CeO_2-Co-Cu|SSZ|LSM	SSZ (~150)	97% CH_4+3% H_2O	800	206.8	24	7
$Ba(Zr_{0.8}Y_{0.2})_{0.9}(Ni_{0.5}Ru_{0.5})_{0.1}O_{3-\delta}$-Ni-YSZ|YSZ|GDC|LSCF	YSZ (~15)	97% CH_4+3% H_2O	700	~620	430	8
Sr_2CoMoO_6|LDC|LSGM|SCF	LSGM (~300)	97% CH_4+3% H_2O	800	527	~17	9
Sr_2NiMoO_6|LDC|LSGM|SCF		97% CH_4+3% H_2O	800	~250	—	9
Ni–$H_xWO_{3-\delta}$–GDC |GDC|BSCF	GDC (—)	100% CH_4	700	~450	200	10
Ni–BZCCY|SDC|PBSCF	SDC (15)	97% CH_4+3% H_2O	500	370	550	11
$PrBa_{0.5}Sr_{0.5}Fe_{1.9}Ge_{0.1}O_{5+\delta}$ |GDC|SSZ|GDC|LSCF	SSZ (~100)	97% CH_4+3% H_2O	800	85	60	12
$(La_{0.2}Sr_{0.8})_{0.925}Ti_{0.55}Mn_{0.35}Ni_{0.1}O_{3-\delta}$ |LDC|LSGM|LSCF	LSGM (~350)	97% CH_4+3% H_2O	800	388	100	13
$Sr_{1.8}La_{0.2}FeMoO_{6-\delta}$ |GDC|YSZ|GDC|LSCF	YSZ (~300)	97% CH_4+3% H_2O	800	790	—	14
$La_{0.5}Sr_{0.5}Fe_{0.9}Mo_{0.1}O_{3-\delta}$|SDC |LSGM|$Ba_{0.6}Sr_{0.4}Co_{0.9}Nb_{0.1}O_{3-\delta}$	LSGM (~280)	97% CH_4+3% H_2O	750<br>800	~310<br>~520		15
CoFe-$PrBaMn_2O_{5+\delta}$|LDC| LSGM|NBSCF50-GDC	LSGM (~300)	97% CH_4+3% H_2O	750<br>800	~100<br>~280	—	16
$Sr_2MgMoO_{6-\delta}$|LDC|LSGM|$SrCo_{0.8}Fe_{0.2}O_{3-\delta}$	LSGM (~300)	100% CH_4	800	483	—	16
Ni-SDC|SDC|SSC	SDC (—)	100% CH_4	700	697	100	17
$Sr_2FeNb_{0.2}Mo_{0.8}O_{6-\delta}$|LSGM|$PrBaCo_2O_{5+\delta}$	LSGM (~150)	97% CH_4+3% H_2O	600<br>700<br>800	120<br>276<br>380	200<br>(700 °C)	18
$Sr_2Fe_{4/3}Mo_{2/3}O_{6-\delta}$|LSGM|LSCF	LSGM (~300)	97% CH_4+3% H_2O	800	130	—	19
FeCr/Ni-ScYSZ|ScYSZ|GDC|LSC	ScYSZ (~8)	2% CH_4+98% Air	600<br>650<br>700	~339<br>~523<br>~820	1200<br>(600 °C)	20
Ru-SFM/Ru-GDC|LSGM|PBSCF/GDC	LSGM (~230)	97% CH_4+3% H_2O	700<br>750	~206<br>~385	200<br>(800°C)	21

			800	~630		
FeCoNiCuAl-SDC/SDC/LSGM/LSCF	LSGM (~300)	100% CH_4	750<br>800	~201<br>~336	20 (750)	22
$La_{0.6}Sr_{0.4}Ti_{0.3}Fe_{0.5}Co_{0.2}O_{3-\delta}$|LSGM|LSCF-SDC	LSGM (~600)	40% CH_4 + 55% Ar + 5% N_2	800	—	1250	23
$Sr_{0.9}Ti_{0.3}Fe_{0.63}Ni_{0.07}O_{3-\delta}$|LDC|LSGM|LDC|LSCF-GDC	LSGM (~200)	97% CH_4+3% H_2O	800<br>750<br>700	655<br>465<br>251	80 (800 ℃)	24
$Ba_{0.6}La_{0.4}Fe_{0.8}Mo_{0.1}N_{i0.1}O_{3-\delta}$|LSGM|LSCF	LSGM (~300)	100% CH_4	800<br>750	203<br>76	325 (800 ℃)	25
HEP|GDC|SSZ|GDC|LSCF	SSZ (~300)	97% CH_4+3% H_2O	600<br>650<br>700	377.8<br>545.1<br>774.5	1000 (700 ℃)	This Work
PBF|GDC|SSZ|GDC|LSCF	SSZ (~300)	97% CH_4+3% H_2O	600<br>650<br>700	157.2<br>230.1<br>345.8	—	This Work

References

1. Sengodan, S. et al. Layered oxygen-deficient double perovskite as an effcient and stable anode for direct hydrocarbon solid oxide fuel cells. *Nat. Mater.* **14**, 205-209 (2015).
2. Chen, Dengjie, Wang, Fucun, Shi, Huangang, Ran, Ran&Shao, Zongping. Systematic evaluation of Co-free $LnBaFe_2O_{5+\delta}$ (Ln = Lanthanides or Y) oxides towards the application as cathodes for intermediate-temperature solid oxide fuel cells. *Electrochim. Acta* **78**, 466-474 (2012).
3. Xue, Liang-Mei et al. Fe-based double perovskite with Zn doping for enhanced electrochemical performance as intermediate-temperature solid oxide fuel cell cathode material. *RSC Adv.* **13**, 30606-30614 (2023).
4. Zhang, Yang, Zhao, Hailei, Zhang, Min, Yang, Tianrang&Du, Zhihong. Development of titanium-doped $SmBaMn_2O_{5+\delta}$ layered perovskite as a robust electrode for symmetrical solid oxide fuel cells. *Int. J. Hydrogen Energy* **48**, 28119-28130 (2023).
5. Liu, Y. et al. $Gd_{0.1}Ce_{0.9}O_{1.95}$ impregnated with $Ba(Ce_{0.9}Y_{0.1})_{0.8}Ni_{0.2}O_{3-\delta}$@CuO as SOFC anode with increased carbon-deposition resistant for direct hydrocarbon fuels. *Int. J. Hydrogen Energy* **62**, 652-658 (2024).
6. Liu, Y. et al. High-performance Ni in-situ exsolved $Ba(Ce_{0.9}Y_{0.1})_{0.8}Ni_{0.2}O_{3-\delta}/Gd_{0.1}Ce_{0.9}O_{1.95}$ composite anode for SOFC with long-term stability in methane fuel. *COMPOS PART B-ENG* **193**, 108033 (2020).
7. Sarruf, B., Hong, J., Steinberger-Wilckens, R.&de Miranda, P. Ceria-Co-Cu-based SOFC anode for direct utilisation of methane or ethanol as fuels. *Int. J. Hydrogen Energy* **45**, 5297-5308 (2020).
8. Zhang, W. et al. A Solid Oxide Fuel Cell Runs on Hydrocarbon Fuels with Exceptional Durability and Power Output. *Adv. Energy Mater.* **12**, 2202928 (2022).
9. Huang, Y. et al. Double-Perovskite Anode Materials Sr_2MMoO_6 (M = Co, Ni) for Solid Oxide Fuel Cells. *Chem. Mate.r* **21**, 2319-2326 (2009).
10. Yoon, D.&Manthiram, A. Hydrogen tungsten bronze as a decoking agent for long-life, natural gas-fueled solid oxide fuel cells. *Energy Environ. Sci.* **7**, 3069-3076 (2014).
11. Chen, Y. et al. A robust fuel cell operated on nearly dry methane at 500 °C enabled by synergistic thermal catalysis and electrocatalysis. *Nat. Energy* **3**, 1042-1050 (2018).
12. Lee, D. et al. Simultaneous A- and B- site substituted double perovskite ($AA'B_2O_{5+\delta}$) as a new high-performance and redox-stable anode material for solid oxide fuel cells. *J. Power Sources* **434**, 226743 (2019).
13. Yang, X. et al. Enhancing Stability and Catalytic Activity by In Situ Exsolution for High-Performance Direct Hydrocarbon Solid Oxide Fuel Cell Anodes. *Ind. Eng. Chem. Res.* **60**, 7826-7834 (2021).
14. Yang, X. et al. Electron doping of $Sr_2FeMoO_{6-\delta}$ as high performance anode materials for solid oxide fuel cells. *J. Mater. Chem. A* **7**, 733-743 (2019).
15. Liu, F. et al. High performance ferrite-based anode $La_{0.5}Sr_{0.5}Fe_{0.9}Mo_{0.1}O_{3-\delta}$ for intermediate-temperature solid oxide fuel cell. *Electrochim Acta* **255**, 118-126 (2017).
16. Huang, Y., Dass, R., Xing, Z. &Goodenough, J. Double perovskites as anode materials for solid-oxide fuel cells. *Science* **312**, 254-257 (2006).

17. Ideris, A., Croiset, E., Pritzker, M.&Amin, A. Direct-methane solid oxide fuel cell (SOFC) with Ni-SDC anode-supported cell. *Int. J. Hydrogen Energy* **42**, 23118-23129 (2017).
18. Ding, H., Tao, Z., Liu, S.&Yang, Y. A redox-stable direct-methane solid oxide fuel cell (SOFC) with $Sr_2FeNb_{0.2}Mo_{0.8}O_{6-\delta}$ double perovskite as anode material. *J. Power Sources* **327**, 573-579 (2016).
19. Xiao, G., Liu, Q., Dong, X., Huang, K.&Chen, F. $Sr_2Fe_{4/3}Mo_{2/3}O_6$ as anodes for solid oxide fuel cells. *J. Power Sources* **195**, 8071-8074 (2010).
20. Liu, F. et al. Nanocomposite Catalyst for High-Performance and Durable Intermediate-Temperature Methane-Fueled Metal-Supported Solid Oxide Fuel Cells. *ACS Appl Mater Interfaces* **14**, 53840-53849 (2022).
21 Hu, F. et al. Smart Dual-Exsolved Self-Assembled Anode Enables Efficient and Robust Methane-Fueled Solid Oxide Fuel Cells. *Advanced Science* **11**, 2306845 (2024).
22. Chen, Dezhi et al. High-Entropy Alloys FeCoNiCuX (X = Al, Mo)-$Ce_{0.8}Sm_{0.2}O_2$ as High-Performance Solid Oxide Fuel Cell Anodes. *ACS Appl Energy Mater* **6**, 1076-1084 (2023).
23. Guo, Y. et al. In situ exsolved CoFe alloy nanoparticles for stable anodic methane reforming in solid oxide electrolysis cells. *Joule* **8**, 2016-2032 (2024).
24. Jiang, Y. Y. et al. In situ exsolved NiFe nanoparticles in Ni-doped $Sr_{0.9}Ti_{0.3}Fe_{0.63}Ni_{0.07}O_{3-\delta}$ anode with a three-dimensionally ordered macroporous structure for solid oxide fuel cells fueled by alkanes. *Chem. Eng. J.* **491**, 151865 (2024).
25. Li, H. X. et al. A redox-reversible A/B-site co-doped $BaFeO_3$ electrode for direct hydrocarbon solid oxide fuel cells. *J. Mater. Chem. A* **12**, 14087-14098 (2024).
26. Yuldashev, R., Jung, H., Park, J. H., Lee, J. H.&Kim, M. C. High-Efficiency CO_2 Electrolysis Enabled by Interface-Engineered Composite Electrolytes in Ni-Based SOEC. *Adv. Sci.*, e18091 (2025).
