# Supplementary material for: Entropy-driven order-to-disorder transition in perovskite anodes for high-performance solid oxide fuel cells
Source: Nat Commun. 2026 Jun 19;17:7721. doi: 10.1038/s41467-026-74549-0 (PMC13434558; doi:10.1038/s41467-026-74549-0)
Supplement: Supplementary file 2 [file 41467_2026_74549_MOESM2_ESM.pdf]

Description of Additional Supplementary Files

Supplementary Data 1: is the structural model file for first-principles calculations of the HEP systems. This file contains the atomic coordinates of all atoms in the computational models, which can be used to reproduce the geometry used in this work.

Supplementary Data 2: are the structural model file for first-principles calculations of the PBF systems. This file contains the atomic coordinates of all atoms in the computational models, which can be used to reproduce the geometry used in this work.
