# Supplementary material for: Entropy-driven order-to-disorder transition in perovskite anodes for high-performance solid oxide fuel cells
Source: Nat Commun. 2026 Jun 19;17:7721. doi: 10.1038/s41467-026-74549-0 (PMC13434558; doi:10.1038/s41467-026-74549-0)
Supplement: Supplementary file 3 [file 41467_2026_74549_MOESM5_ESM.pdf]

nature portfolio

Peer Review File

Entropy-Driven Order-to-Disorder Transition in Perovskite Anodes for High-Performance Solid Oxide Fuel Cells

Corresponding Author: Professor Zhenhua Wang

This file contains all reviewer reports in order by version, followed by all author rebuttals in order by version.

Version 0:

Reviewer comments:

Reviewer #1

(Remarks to the Author)
High-entropy oxides have emerged as a hot topic in the field of energy conversion and storage. This article reports an entropy-driven order-to-disorder transition in a high-entropy perovskite oxide (HEP), which is a very intriguing observation. The authors demonstrate that disordered HEP exhibit enhanced hydration capacity, thereby promoting the hydrocarbon steam reforming and carbon elimination processes involved in solid oxide fuel cell (SOFC) anodes. The as-prepared SOFC using disordered HEP anodes achieve high power density and excellent stability. The manuscript demonstrates good quality, employing multiple methodologies for experimental validation, which enhances the credibility of the results. However, certain points need to be addressed before the consideration for acceptance.
1. The chemical equations presented in lines 66-72 should be revised to include the physical states (e.g., (g) for gas, (l) for liquid, (s) for solid) of all reactants and products. This is a standard practice that clarifies the reaction conditions.
2. While the synthesis was performed using a nominal equimolar stoichiometry of five rare-earth elements, the actual chemical composition of the resulting high-entropy phase (HEP) has not been characterized. It is recommended that the authors provide experimental evidence (ICP-MS) to confirm the final elemental distribution
3. It is noted that Figure S4 is not cited in the manuscript. Ensure that the numbering of the remaining supplementary figures and their in-text references are corrected.
4. The temperature range of the x-axis in Figure 3c is excessively wide. It’s recommended to adjust the axis range to better focus on the relevant data region.
5. The abbreviations “SSZ” and “LSCF” appear in line 270 without being defined. Upon their first appearance, the full names and corresponding chemical formulae should be provided.
6. The long-term stability evaluation currently presents voltage variation curves and SEM images. To provide a more comprehensive assessment of the structural stability, it is recommended to include additional post-test characterizations of the anode’s phase structure.
7. Figure 4d relies solely on Raman spectroscopy to monitor the carbon deposition, which is indirect. To more convincingly demonstrate the reduction of carbon deposits, direct observational evidence from SEM should be provided.
8. The introduction section adequately outlines the existing challenges of Ni-based anodes. However, the subsequent performance comparison lacks sufficient references to these Ni-based anodes. It would be beneficial to include more citations of relevant Ni-based anode studies to provide a more comprehensive and contextualized discussion.

Reviewer #2

(Remarks to the Author)
The manuscript entitled ‘Entropy-Driven Order-to-Disorder Transition in Perovskite Anodes for High-Performance Solid Oxide Fuel Cells’ presents an impressive and conceptually innovative study. The authors introduce a high-entropy strategy to break the layered A-site cation ordering, demonstrating that configurational entropy can effectively drive a transition from an ordered double perovskite to a fully disordered cubic perovskite which exhibits enhanced electrochemical activity. The manuscript is well written, the experiments are comprehensive and rigorous, and the conceptual insights are highly relevant to the SOFC community. Therefore, I would like to recommend it for publication after a minor revision.
1. Please keep the font consistent in the images, for example, EDS elements in Fig. 1c and 1d.
2. In Fig. 2b, please list the g-factor signal center to clarify the vacancy species.
3. Please add intensity scale of Wavelet transforms of the k3-weighted Fe K-edge EXAFS spectra of Fig. 2e PBF and Fig. 2f HEP.

4. Please standardize the notation in the manuscript and figures. For example, the minus sign appears on page 11: ‘The results show that the oxygen ion bulk diffusion coefficient (Dchem) increases from 6.51 × 10−6 cm2 s−1 for PBF to 3.51 × 10−5 cm2 s−1 for HEP (Fig S10), reflecting the inherently fast oxygen-ion diffusion nature in the disordered HEP.’ On pages 13: ‘774.53, 545.14, 377.77, and 136.19 mW cm-2’
5. The hydration behavior of HEP is clearly superior to PBF, as shown by TGA and in situ Raman. It would be helpful to add a short paragraph explicitly linking the higher oxygen-vacancy concentration, Fe electronic structure, and local coordination environment to the faster water absorption and dissociation kinetics.
6. Please standardize the coordinate format in the images, for example, the x and y coordinates in Fig. 4e are Reaction coordinates and Relative Energy.
7. The manuscript frequently refers to operation under “nearly dry methane” with 3 vol% H2O. How the significance of the 3% H2O condition and the robustness of the anode under very low steam contents?
8. Please explain the generality of the entropy-driven strategy and discuss how the proposed entropy-driven order-to-disorder transition might be extended to other perovskites.
9. Fig.3f, what’s the temperature? no description for fig.3g and h
10. in Fig.4a, it is interesting to observe the gas composition before and during the test is interesting, as it helps us understand whether the additional current load will consume only CO and H2 or methane, and how much.

Reviewer #3

(Remarks to the Author)
The manuscript addresses the development of a Ni-free oxide fuel electrode for solid oxide cells, a topic of clear relevance given the limitations of conventional NiO-8YSZ systems, particularly for hydrocarbon operation and long-term stability. The authors propose an entropy-driven design strategy based on a layered double perovskite, aiming to enhance oxygen vacancy concentration, ionic transport, surface hydration and, ultimately, coking resistance. While the reported electrochemical performance and durability under methane are promising, the work in its current state relies heavily on the current trend of high-entropy materials without providing sufficiently direct and conclusive evidence that entropy itself is the governing factor behind the observed behaviour. As a result, the manuscript does not yet convincingly demonstrate that the proposed material represents an exceptional or fundamentally distinct class of fuel electrodes, rather than a favourable compositional variant within an already explored design space.
In my opinion, for a work published in Nature Communications, conceptual novelty must be supported by direct experimental evidence that rigorously validates the proposed mechanisms, rather than relying on broadly defined concepts such as entropy stabilization. The main critical aspects that need to be addressed to support the central claims of the manuscript are outlined below.
1) Major Comment 1: Lack of electrochemical validation
It is surprising that a study focused on the synthesis and characterisation of a new fuel electrode material does not present any EIS data, neither in the main manuscript nor in the Supplementary Information. EIS is a fundamental requirement in the development and validation of new materials for solid oxide cells. The statement in the Supplementary Information that “symmetric cells were prepared for EIS measurements”, without presenting any corresponding results, is insufficient at this level. This is not merely a matter of missing data; rather, the mechanistic narrative of the manuscript is constructed in the absence of the standard electrochemical experiment required to validate or refute the proposed interpretations.
Side/related concerns:
I) Electrode sintering and attachment: The same sintering conditions are applied to both electrodes (1100 ºC 2h.) without demonstrating comparable attachment or interfacial quality. Without EIS or symmetric-cell data, differences in contact resistance cannot be excluded.
II) Microstructural and interfacial effects:
In the absence of impedance deconvolution, it is not possible to distinguish intrinsic fuel-electrode improvements from microstructural or interfacial contributions within the multilayer cell.
III) Reproducibility: The lack of EIS data prevents assessment of cell-to-cell variability and robustness of the reported performance.
IV) The reported electrode area is extremely small (Supplementary info "with an electrode area of 0.07 cm2"). If this is not a typographical error, such a limited active area may strongly amplify edge and contact effects. Without EIS, it is not possible to assess how this influences the reported performance and mechanistic interpretation. The authors should confirm the electrode area and clarify its impact.

Major Comment 2: Entropy is not isolated as a governing design parameter
The manuscript does not clearly justify the choice of this specific stoichiometric composition beyond invoking configurational entropy, nor does it discuss why alternative compositions or lower-complexity variants were not considered.
- What specific criteria, beyond configurational entropy, guided the selection of the chosen stoichiometric composition?
- Were alternative compositions with intermediate/lower configurational entropy considered or tested, and if so, how did their electrochemical behaviour compare?
- How do the authors exclude contributions from lattice strain, defect association, or differences in sintering and microstructure as the dominant factors governing the observed behaviour?

Major Comment 3: Lack of experimental validation of the proposed mechanism
The manuscript links ex situ evidence related to hydration, reforming activity and carbon removal to the electrochemical behaviour observed in current-voltage (iV) curves; however, such correlations are not sufficient to substantiate the proposed mechanism. In the absence of operando or operating-condition-resolved validation, the mechanistic interpretation remains largely correlative. Once again, the lack of EIS data and deconvoluted resistances prevents a clear assessment of whether the observed differences arise from intrinsic fuel-electrode behaviour or from other cell-related contributions.

Major Comment 4: Overinterpretation of oxygen vacancy concentration from ex situ spectroscopy
The manuscript places significant weight on oxygen vacancy concentration to support the proposed mechanistic framework. While the authors employ several characterisation techniques to assess defect chemistry, a central part of the argument relies on ex situ surface-sensitive measurements, particularly O 1s XPS deconvolution. It is well established that XPS cannot directly probe the absence of oxygen species, nor can it reliably discriminate between intrinsic lattice oxygen and surface adsorbates or hydroxylated (for example) species under ambient conditions. This has been extensively discussed in the literature and should be more carefully addressed here.
Idriss et al. Surface Science 712 (2021) 121894
Terry et al. Chemistry of Materials 2023, 35, 5468–5474

Comments:
• Higher-resolution micrographs should be provided for Fig. 4b and 4c, as the current images lack scale bars, making quantitative or comparative assessment difficult. In addition, the cross-sectional image in Fig. S14 does not show the complete cell configuration, limiting the evaluation of layer thicknesses and interfaces across the full device.
• Synchrotron measurement details: The wavelength used for the synchrotron XRD measurements (Fig. 1a) should be explicitly stated to ensure reproducibility and clarity
• Structural analysis: The authors perform Rietveld refinement using laboratory XRD data despite having access to synchrotron XRD measurements. A justification for this choice should be provided, or alternatively, Rietveld refinement of the synchrotron data should be included to take full advantage of the higher resolution.

Reviewer #4

(Remarks to the Author)
The manuscript presents a well-structured investigation into the development of a high-entropy perovskite anode with the composition $La_{0.2}Pr_{0.2}Sm_{0.2}Gd_{0.2}Y_{0.2}BaFe_2O_{5+\delta}$ (HEP) for hydrocarbon-fueled solid oxide fuel cells (SOFCs), demonstrating promising catalytic activity and durability. A major revision is recommended. Before considering its publication, the following points should be adequately addressed:
(1) Introduction
A more detailed discussion on high-entropy materials, particularly in the context of SOFC anode applications, is recommended. The rationale behind the selection of the five A-site elements (La, Pr, Sm, Gd, and Y) should be clarified. For instance, would alternative combinations of five elements yield comparable or superior performance? How would the properties be affected if only three or four elements were used at the A-site?
(2) Material stability
Regarding Figure S6, please provide an explanation for the disappearance of the diffraction peak at approximately 77° after 80 hours of reduction.
(3) Mechanism elucidation
Beyond the disordered structure, are there other contributing factors—such as surface chemistry, coordination environment, or local lattice distortions—that could account for the enhanced anode performance?
(4) Performance comparison
In Figure 3c, several high-performance results from the literature are omitted. To ensure a fair comparison, it is essential to include cells with similar configurations (e.g., the anode-supported cells normally show higher performance than the electrolyte-supported ones), electrolyte composition, and electrolyte thickness. A separate table summarizing these cell parameters is recommended.
(5) Electrochemical Impedance Spectroscopy (EIS)
EIS measurements under various fuel conditions and temperatures should be provided to facilitate a deeper understanding of the cell performance.
(6) Figure S17
Please specify the testing temperature used for the experiments in Figure S17.
(7) Experimental section
It is noted that the GDC barrier layer was co-sintered with the SSZ electrolyte at 1400 °C for 5 hours. However, GDC is known to react with SSZ at temperatures above 1250 °C. Additionally, the electrode area of 0.07 cm^2 is relatively small and may lead to overestimated performance. Fabrication and testing of cells with larger electrode areas (e.g., 0.5–1 cm^2) are advised.
(8) Language and Labeling
There are instances of mislabeling in the main text (Fig. S4 and S5) and inconsistent abbreviations in the supporting figures (e.g., “HEM” and “PBC”). These should be corrected for consistency and clarity.

Version 1:

Reviewer comments:

Reviewer #1

(Remarks to the Author)
It can be accepted in the current form.

Reviewer #2

(Remarks to the Author)
Authors have solved all questions I raised. I think it is ready for publication.

Reviewer #3

(Remarks to the Author)
The revised manuscript shows a clear and significant improvement compared to the original submission, and most of the concerns raised during the previous round of review have been carefully addressed. In particular, the addition of comprehensive impedance spectroscopy data, including DRT analysis under relevant operating conditions, effectively resolves the main issues raised in my previous report regarding electrochemical validation and mechanistic support. At the same time, the authors have responded constructively to the comments of the other reviewers, incorporating additional control experiments, clarifications, and post-test characterizations that improve the overall clarity and robustness of the work. Overall, the manuscript is now much clearer, more complete, and scientifically stronger.

Minor Comments:
1- The weak diffraction feature labeled as “carbon” in the XRD pattern (Fig. R2, Fig S39) would benefit from a more cautious assignment. While carbon deposition under methane operation is plausible, the identification based on a single low-intensity peak is not fully conclusive. It may be helpful to briefly clarify whether this feature is consistent with graphitic carbon (e.g., position and peak shape), or to acknowledge that other surface species (such as minor carbonate formation) cannot be entirely excluded from XRD alone. A more neutral labeling (e.g., “possible carbonaceous species”) or a short clarification in the text would improve the robustness of the interpretation without requiring additional experiments.
2- Fig R14 (Fig S24):a)temperature units are missing

3- Fig R15. A moderate variation in the high-frequency intercept (Rs) across different fuel atmospheres can be physically reasonable. However, since the electrolyte contribution should remain largely constant at fixed temperature, it would be helpful for the authors to briefly comment on the origin of the observed Rs variations when changing atmospheres.

4- In Fig. R15c, the x-axis title appears to contain a typo and should not read “Temperature”

Reviewer #4

(Remarks to the Author)
The revised manuscript is suggested to be published.

Open Access This Peer Review File is licensed under a Creative Commons Attribution 4.0 International License, which permits use, sharing, adaptation, distribution and reproduction in any medium or format, as long as you give appropriate credit to the original author(s) and the source, provide a link to the Creative Commons license, and indicate if changes were made.
In cases where reviewers are anonymous, credit should be given to 'Anonymous Referee' and the source.
The images or other third party material in this Peer Review File are included in the article's Creative Commons license, unless indicated otherwise in a credit line to the material. If material is not included in the article's Creative Commons license and your intended use is not permitted by statutory regulation or exceeds the permitted use, you will need to obtain permission directly from the copyright holder.
To view a copy of this license, visit https://creativecommons.org/licenses/by/4.0/

Response to Reviewers

Dear Reviewers:

We would like to express our sincere appreciation for the time and effort the reviewers dedicated to reviewing our manuscript (NCOMMS-25-87642) entitled "**Entropy-Driven Order-to-Disorder Transition in Perovskite Anodes for High-Performance Solid Oxide Fuel Cells**". We also highly appreciate the thoughtful comments and suggestions provided by the reviewers, which have substantially enhanced the quality and clarity of our manuscript.

In response, we have carefully revised the manuscript to address all the points raised. Below, we provide our point-by-point responses. To facilitate the review process, a table of contents has been included; reviewers may click the links to navigate directly to the corresponding comment.

Thank you once again to the referees for their valuable comments.

Table of contents

Reviewer #1 3

Comment 1-1: 4

Comment 1-2 5

Comment 1-3 6

Comment 1-4 7

Comment 1-5 8

Comment 1-6 9

Comment 1-7 11

Comment 1-8 14

Reviewer #2 17

Comment 2-1 18

Comment 2-2 19

Comment 2-3: 20

Comment 2-4 21

Comment 2-5 22

Comment 2-6 22

Comment 2-7 24
Comment 2-8 27
Comment 2-9 31
Comment 2-10 33
Reviewer #3 35
Major Comment 3-1 36
3-1-(I) 40
3-1-(II) 43
3-1-(III) 45
3-1-(IV) 47
Major Comment 3-2 49
3-2-(I) 51
3-2-(II) 53
3-2-(III) 60
Major Comment 3-3 65
Major Comment 3-4 68
Comments 3-5 71
3-5-(I) 71
3-5-(II) 74
3-5-(III) 75
Reviewer #4 77
Comment 4-1 78
Comment 4-2 85
Comment 4-3 87
Comment 4-4 90
Comment 4-5 93
Comment 4-6 95
Comment 4-7 96
Comment 4-8 99

Reviewer #1

High-entropy oxides have emerged as a hot topic in the field of energy conversion and storage. This article reports an entropy-driven order-to-disorder transition in a high-entropy perovskite oxide (HEP), which is a very intriguing observation. The authors demonstrate that disordered HEP exhibit enhanced hydration capacity, thereby promoting the hydrocarbon steam reforming and carbon elimination processes involved in solid oxide fuel cell (SOFC) anodes. The as-prepared SOFC using disordered HEP anodes achieve high power density and excellent stability. The manuscript demonstrates good quality, employing multiple methodologies for experimental validation, which enhances the credibility of the results. However, certain points need to be addressed before the consideration for acceptance.

Response to Reviewer #1:

We thank the reviewer for careful reading of our manuscript and the acknowledgement of our work. Following the suggestions from the reviewer, we have made detailed revisions to improve our manuscript. The modifications have been marked in yellow in the revised manuscript.

Comment 1-1:

The chemical equations presented in lines 66-72 should be revised to include the physical states (e.g., (g) for gas, (l) for liquid, (s) for solid) of all reactants and products. This is a standard practice that clarifies the reaction conditions.

Response to 1-1:

We appreciate the reviewer for this thoughtful suggestion.

We fully agree that including physical states is standard practice to clarify reaction conditions. Accordingly, we have duly revised the chemical equations in lines 66-72 by adding the appropriate state symbols (e.g., (g), (l), (s), (aq)) for all reactants and products.

The corresponding labels have been added in the revised manuscript as follows:

$$CH_4(g) + H_2O(g) \rightarrow CO(g) + H_2(g) \quad (1)$$

$$CO(g) + H_2O(g) \rightarrow CO_2(g) + H_2(g) \quad (2)$$

$$H_2O(g) + C(s) \rightarrow CO(g) + H_2(g) \quad (3)$$

$$CH_4(g) + 2O_2(g) \rightarrow CO_2(g) + 2H_2(g) \quad (4)$$

Water-free reactions

$$CH_4(g) \rightarrow C(s) + 2H_2(g) \quad (5)$$

$$2CO(g) \rightarrow C(s) + CO_2(g) \quad (6)$$

Comment 1-2

While the synthesis was performed using a nominal equimolar stoichiometry of five rare-earth elements, the actual chemical composition of the resulting high-entropy phase (HEP) has not been characterized. It is recommended that the authors provide experimental evidence (ICP-MS) to confirm the final elemental distribution.

Response to 1-2:

We thank the reviewer for raising this valid point.

Following the reviewer's recommendation, we have performed inductively coupled plasma mass spectrometry (ICP-MS) analysis on the as-synthesized HEP powder. ICP-MS analysis (**Table R1**) yielded the following elemental stoichiometries: La = 0.205, Pr = 0.203, Sm = 0.195, Gd = 0.192, Y = 0.205. These measured ratios are in good agreement with the nominal composition in the designed formula. The results confirm the successful incorporation of all five rare-earth elements with a near-equimolar distribution.

Table R1 (also **Table S2**). Stoichiometry of the HEP compounds determined by inductively coupled plasma mass spectrometry.

	La	Pr	Sm	Gd	Y	Ba	Fe
HEP	0.205	0.203	0.195	0.192	0.205	1.006	2.000

Related discussions (yellow-highlighted in Line 139-142) have been added in the revised manuscript as follows:

The stoichiometry of the resulting HEP was further determined by inductively coupled plasma mass spectrometry (ICP-MS), with the measured composition showing excellent agreement with the nominal stoichiometry (Table S2).

Comment 1-3

It is noted that Figure S4 is not cited in the manuscript. Ensure that the numbering of the remaining supplementary figures and their in-text references are corrected.

Response to 1-3:

We thank the reviewer for noting the inconsistency in the referencing of the supplementary figures.

Following this comment, we have carefully reviewed the entire manuscript and the Supplementary Information. The numbering of **Fig. S4**, **S5**, and **S6** has been standardized and synchronized. Consequently, all in-text citations have been updated accordingly. Furthermore, a citation and contextual description for **Fig. S4** have been inserted into the main text. This thorough check ensures the accuracy and clarity of the figure citations.

Related discussions have been added in the revised manuscript as follows:

Line 138-139: Transmission Electron Microscopy-EDS elemental mappings confirm that all constituent elements are homogeneously distributed throughout the material (Fig. S4).

Line 165-167: Based on these considerations, several representative structure models with varying Ba^{2+} substitution in the Pr-layer ($x = 0$, 0.2, 0.5, 0.7, 1) have been constructed (Fig. S5).

Line 178-179: Consequently, the configurational entropy of these structural models in Fig. S5 was also systematically calculated to systematically identify the optimal structural model.

Line 221-222: XRD analysis (Fig. S16) confirms that the HEP maintains its structural integrity throughout the entire testing period.

Comment 1-4

The temperature range of the x-axis in Figure 3c is excessively wide. It's recommended to adjust the axis range to better focus on the relevant data region.

Response to 1-4:

We appreciate the reviewer's recommendation.

The x-axis range in **Fig. 3c** has been revised accordingly in the updated manuscript. As shown in **Fig. R1**, the plot has been refined to highlight the pertinent temperature interval of 600-800 °C, thereby providing enhanced clarity for the primary data of interest.

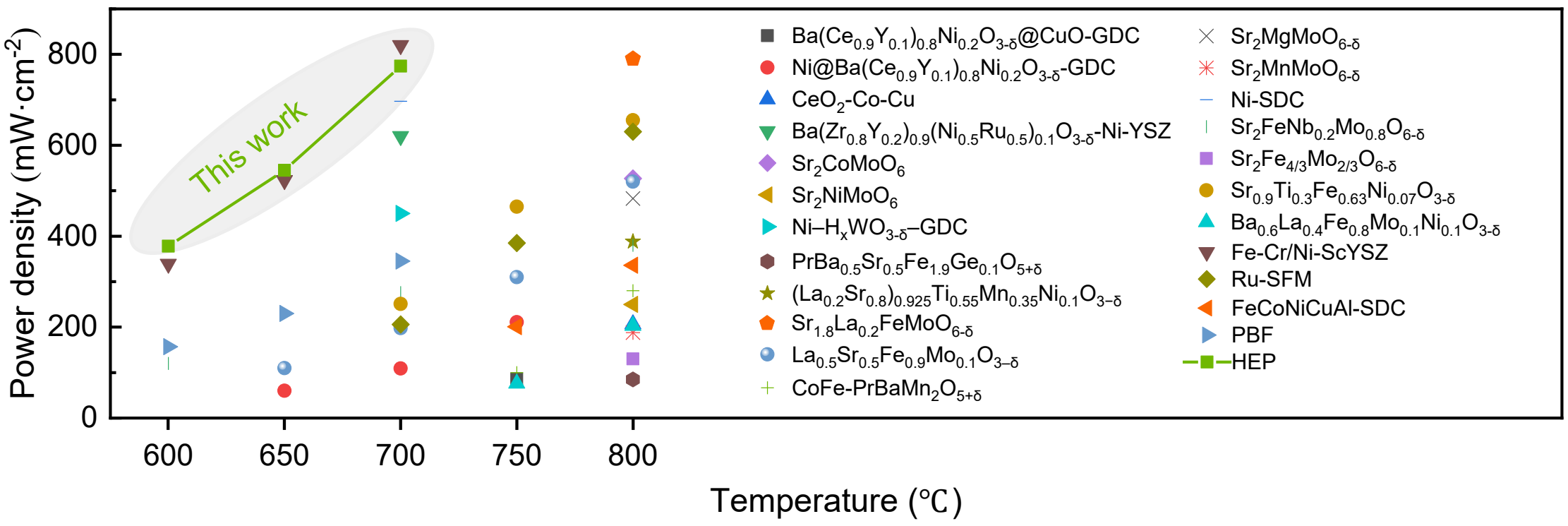

Fig. R1 (also **Fig. 3c)** Comparison of the PPDs of the SOFC technology with different anodes using methane as the fuel.

Comment 1-5

The abbreviations "SSZ" and "LSCF" appear in line 270 without being defined. Upon their first appearance, the full names and corresponding chemical formulae should be provided.

Response to 1-5:

We thank the reviewer for pointing out this omission.

We apologize for this oversight. The full names and chemical formulae for "SSZ" and "LSCF" have now been provided upon their first appearance in the manuscript. Specifically, they are defined as follows: SSZ ($Sc_{0.1}Zr_{0.9}O_{1.95}$) and LSCF ($La_{0.6}Sr_{0.4}Co_{0.2}Fe_{0.8}O_{3-\delta}$). All subsequent uses of these abbreviations are consistent with the definitions provided.

Related discussions have been added in the revised manuscript as follows:

Line 325-327: EIS measurements were carried out on SSZ ($Sc_{0.1}Zr_{0.9}O_{1.95}$) electrolyte-supported symmetrical cells under realistic operating conditions (3% H_2O-CH_4 atmosphere) at various temperatures.

Line 341-343: The output performance of the disordered HEP anode was evaluated in an electrolyte-supported SOFC with a dense SSZ electrolyte layer (~300 μm) and a porous LSCF ($La_{0.6}Sr_{0.4}Co_{0.2}Fe_{0.8}O_{3-\delta}$) cathode (Fig. S27).

Comment 1-6

The long-term stability evaluation currently presents voltage variation curves and SEM images. To provide a more comprehensive assessment of the structural stability, it is recommended to include additional post-test characterizations of the anode's phase structure.

Response to 1-6:

We sincerely thank the reviewer for this insightful suggestion.

We fully agree that evaluating the phase structure after long-term testing is crucial for a comprehensive assessment of structural stability. In response, we have conducted additional post-test characterizations on the tested anode. As shown in **Fig. R2**, X-ray diffraction (XRD) analyses confirm that the primary crystal structure remains intact, with no detectable phase decomposition or formation of new impurity phases after the stability test. In contrast, characteristic peaks indicative of carbon deposition is observed for the PBF anode. This new evidence directly substantiates our claims regarding the excellent structural stability and anti-coking performance of the HEP material, effectively complementing the existing electrochemical and morphological data.

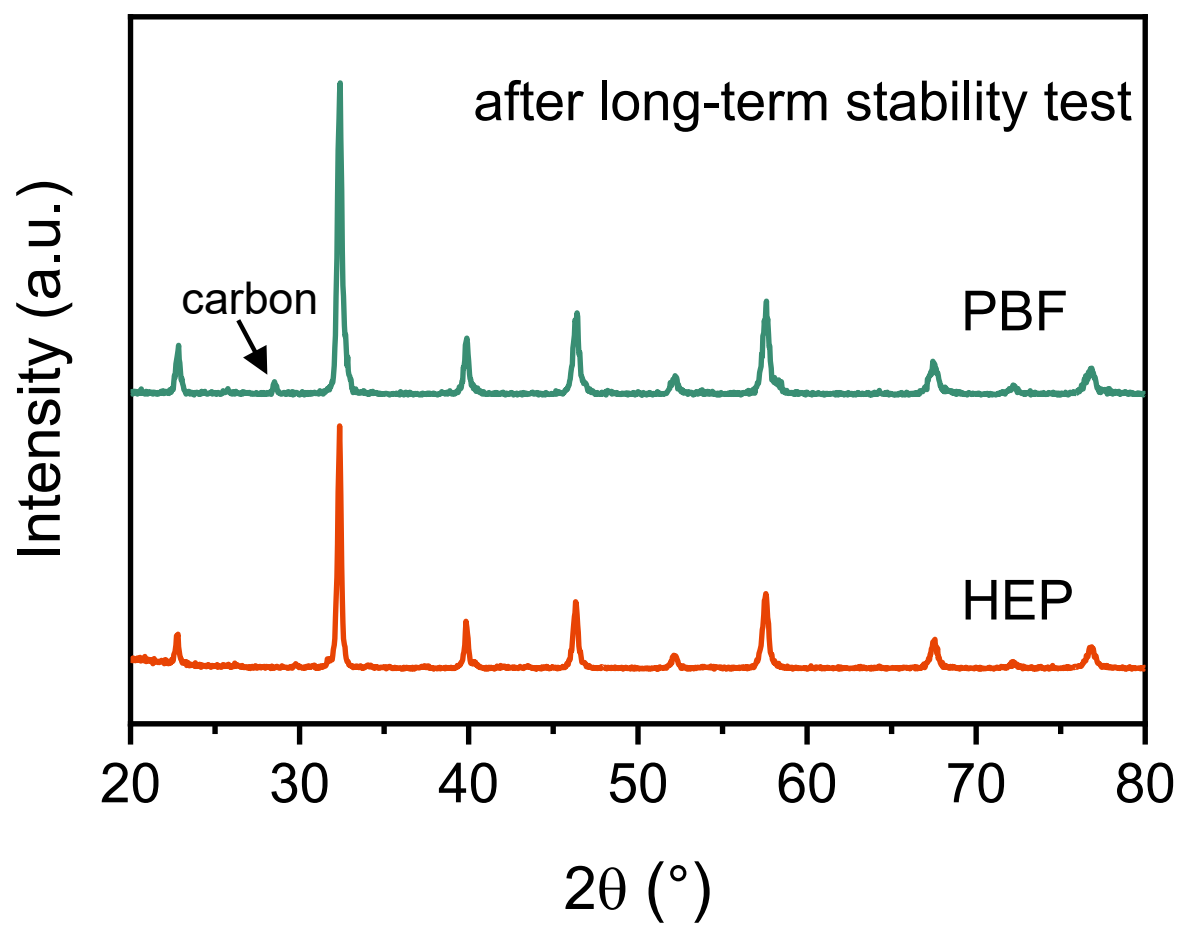

Fig. R2 (also **Fig. S39**) XRD patterns of HEP and PBF anode after long-term stability test.

Related discussions (yellow-highlighted in Line 437-439) have been added in the revised manuscript as follows:

Moreover, XRD analysis of the post-test HEP sample (Fig. S39) revealed a monophasic structure, indicating excellent structural stability of HEP under the testing conditions.

Comment 1-7

Figure 4d relies solely on Raman spectroscopy to monitor the carbon deposition, which is indirect. To more convincingly demonstrate the reduction of carbon deposits, direct observational evidence from SEM should be provided.

Response to 1-7:

We sincerely appreciate the reviewer for this insightful comment, which greatly strengthens the persuasiveness of our carbon deposition characterization.

In response to your suggestion, we have supplemented direct SEM morphological evidence recording the evolution of carbon deposition on HEP and PBF anodes during the Raman spectroscopy test process, and these new results are integrated into the corresponding Supplementary Information (**Fig. S42, 43**), complementing the indirect Raman spectral data with direct visual observations.

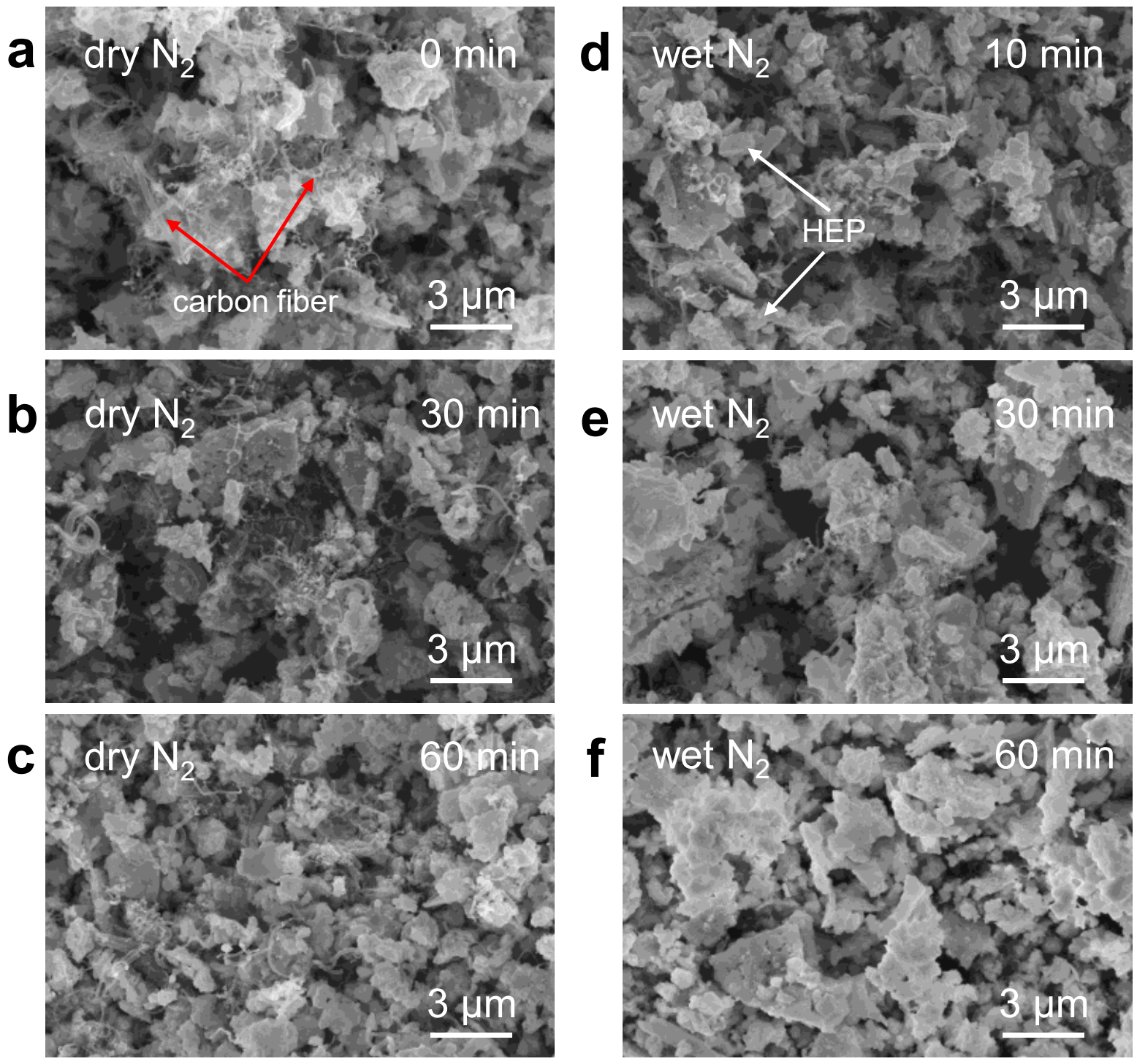

Fig. R3 (also **Fig. S42**) The SEM images of the carbon-contaminated HEP during the Raman test.

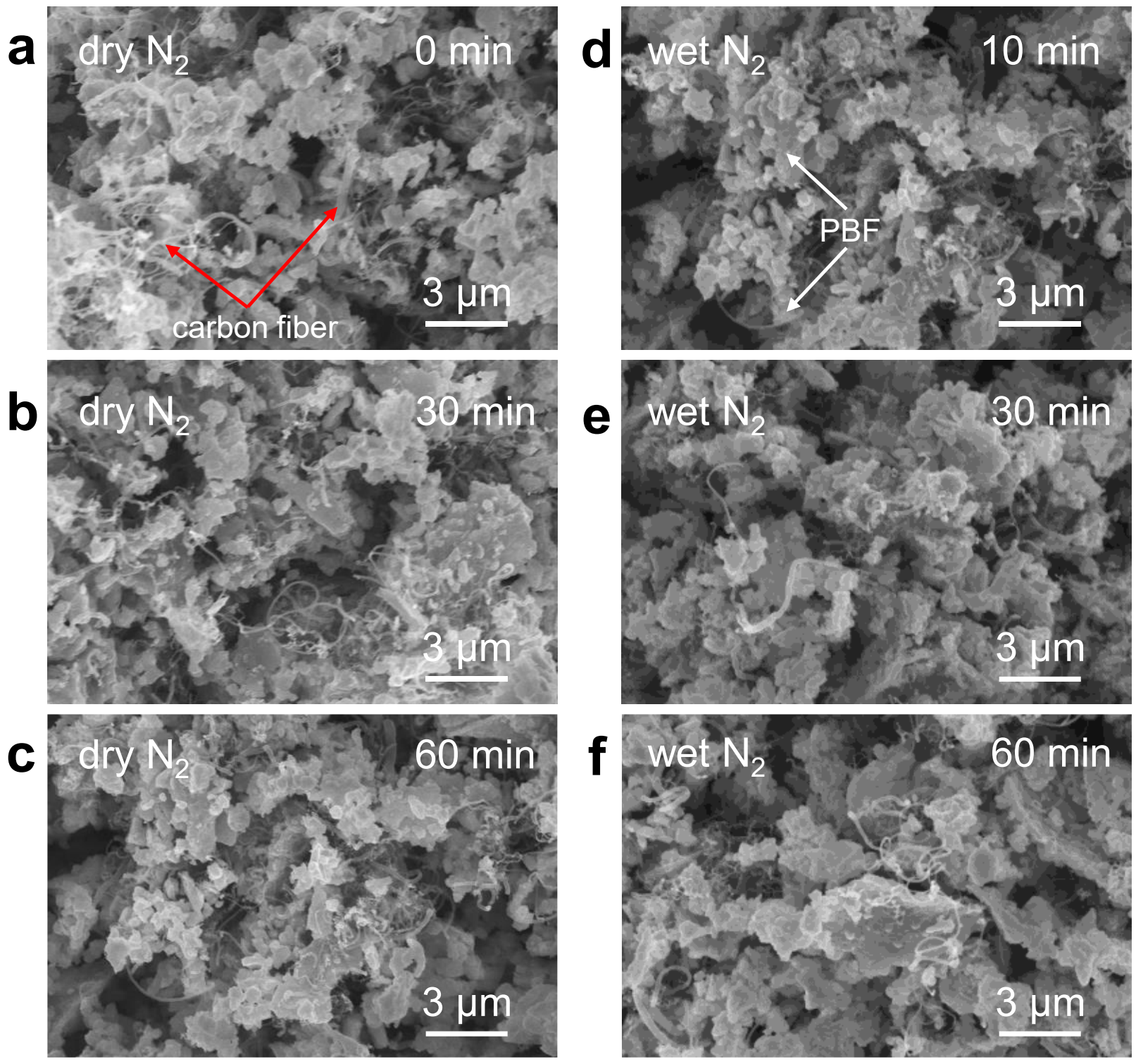

Fig. R4 (also **Fig. S43**) The SEM images of the carbon-contaminated PBF during the Raman test.

The supplementary time-resolved SEM characterizations clearly reveal the dynamic variation of surface carbon deposits on the two anodes under different atmospheres: under a dry N_2 atmosphere, the carbon deposits on both HEP and PBF surfaces show no observable reduction; after switching to a humid atmosphere (3 vol% H_2O), the carbon deposits on the HEP surface decrease gradually over time and are completely eliminated after 60 min, whereas the carbon deposits on the PBF surface persist and remain unremoved even after 60 min of testing.

These direct SEM morphological observations are highly consistent with our Raman spectroscopy results (the disappearance of carbon characteristic peaks for HEP and persistent carbon peaks for PBF after 60 min). The combination of direct SEM visual evidence and indirect Raman spectral data forms a complementary characterization system, which more convincingly and rigorously demonstrates the superior carbon elimination capability of the HEP anode. All supplementary time-resolved SEM images and detailed morphological analysis have been added to the revised manuscript (Line 468-473), with high-resolution SEM micrographs provided in the Supplementary Information for further detailed observation.

Related discussions (yellow-highlighted in Line 468-473) have been added in the revised manuscript as follows:

Fig. S42 and S43 provide the corresponding post-tested SEM images after exposure in dry and wet atmospheres for different time. After 60 minutes in wet N_2 atmosphere, carbon deposits on the HEP sample surface were completely removed, whereas carbon residues remained visible on the PBF sample surface throughout the entire test. The corresponding transformation process of carbon deposits on the anode surface is schematically illustrated in the middle of Fig. 4d.

Comment 1-8

The introduction section adequately outlines the existing challenges of Ni-based anodes. However, the subsequent performance comparison lacks sufficient references to these Ni-based anodes. It would be beneficial to include more citations of relevant Ni-based anode studies to provide a more comprehensive and contextualized discussion.

Response to 1-8:

Thank you for this valuable suggestion.

In accordance with your comment, we have now included performance data for two representative Ni-based anodes operating on methane fuel in our comparison. These additions are highlighted in yellow in the newly added **Table R2** and have also been incorporated into **Fig. R1 (Fig. 3c)** for visual comparison. As the updated results show, the newly developed HEP anode demonstrates superior power output and exceptional anti-coking performance relative to these referenced Ni-based systems, thereby providing a more comprehensive and contextualized discussion as you recommended.

Table R2 (also **Table S5**). Performance and durability comparison of SOFC using methane as fuel in the range of 600-800 °C

Cell configuration	Electrolyte and thickness (μm)	Fuel mixture	Temperature (°C)	Peak power density ($mW\cdot cm^{-2}$)	Durability (h)	Ref.
$Ba(Ce_{0.9}Y_{0.1})_{0.8}Ni_{0.2}O_{3-\delta}$@CuO-GDC|GDC|LSCF	GDC (~20)	97% CH_4+3% H_2O	750	86	160	9
Ni@$Ba(Ce_{0.9}Y_{0.1})_{0.8}Ni_{0.2}O_{3-\delta}$-GDC|GDC|LSCF	GDC (—)	97% CH_4+3% H_2O	650<br>700<br>750	60<br>109<br>211	100 (750 °C)	10
CeO_2-Co-Cu|SSZ|LSM	SSZ (~150)	97% CH_4+3% H_2O	800	206.8	24	11
$Ba(Zr_{0.8}Y_{0.2})_{0.9}(Ni_{0.5}Ru_{0.5})_{0.1}O_{3-\delta}$-Ni-YSZ|YSZ|GDC|LSCF	YSZ (~15)	97% CH_4+3% H_2O	700	~620	430	12
Sr_2CoMoO_6|LDC|LSGM|SCF	LSGM (~300)	97% CH_4+3% H_2O	800	527	~17	13
Sr_2NiMoO_6|LDC|LSGM|SCF		97% CH_4+3% H_2O	800	~250	—	13
Ni–$H_xWO_{3-\delta}$–GDC |GDC|BSCF	GDC (—)	100% CH_4	700	~450	200	14
Ni–BZCCY|SDC|PBSCF	SDC (15)	97% CH_4+3% H_2O	500	370	550	15

$PrBa_{0.5}Sr_{0.5}Fe_{1.9}Ge_{0.1}O_{5+\delta}$ |GDC|SSZ|GDC|LSCF	SSZ (~100)	97% CH_4+3% H_2O	800	85	60	16
$(La_{0.2}Sr_{0.8})_{0.925}Ti_{0.55}Mn_{0.35}Ni_{0.1}O_{3-\delta}$ |LDC|LSGM|LSCF	LSGM (~350)	97% CH_4+3% H_2O	800	388	100	17
$Sr_{1.8}La_{0.2}FeMoO_{6-\delta}$ |GDC|YSZ|GDC|LSCF	YSZ (~300)	97% CH_4+3% H_2O	800	790	—	18
$La_{0.5}Sr_{0.5}Fe_{0.9}Mo_{0.1}O_{3-\delta}$|SDC |LSGM|$Ba_{0.6}Sr_{0.4}Co_{0.9}Nb_{0.1}O_{3-\delta}$	LSGM (~280)	97% CH_4+3% H_2O	750<br>800	~310<br>~520		19
CoFe-$PrBaMn_2O_{5+\delta}$|LDC| LSGM|NBSCF50-GDC	LSGM (~300)	97% CH_4+3% H_2O	750<br>800	~100<br>~280	—	20
$Sr_2MgMoO_{6-\delta}$|LDC|LSGM|$SrCo_{0.8}Fe_{0.2}O_{3-\delta}$	LSGM (~300)	100% CH_4	800	483	—	20
Ni-SDC|SDC|SSC	SDC (—)	100% CH_4	700	697	100	21
$Sr_2FeNb_{0.2}Mo_{0.8}O_{6-\delta}$|LSGM|$PrBaCo_2O_{5+\delta}$	LSGM (~150)	97% CH_4+3% H_2O	600<br>700<br>800	120<br>276<br>380	200<br>(700 ℃)	22
$Sr_2Fe_{4/3}Mo_{2/3}O_{6-\delta}$|LSGM|LSCF	LSGM (~300)	97% CH_4+3% H_2O	800	130	—	23
FeCr/Ni-ScYSZ|ScYSZ|GDC|LSC	ScYSZ (~8)	2% CH_4+98% Air	600<br>650<br>700	~339<br>~523<br>~820	1200<br>(600 ℃)	24
Ru-SFM/Ru-GDC|LSGM|PBSCF/GDC	LSGM (~230)	97% CH_4+3% H_2O	700<br>750<br>800	~206<br>~385<br>~630	200<br>(800℃)	25
FeCoNiCuAl-SDC/SDC/LSGM/LSCF	LSGM (~300)	100% CH_4	750<br>800	~201<br>~336	20<br>(750)	26
$La_{0.6}Sr_{0.4}Ti_{0.3}Fe_{0.5}Co_{0.2}O_{3-\delta}$|LSGM|LSCF-SDC	LSGM (~600)	40% CH_4 + 55% Ar + 5% N_2	800	—	1250	27
$Sr_{0.9}Ti_{0.3}Fe_{0.63}Ni_{0.07}O_{3-\delta}$|LDC|LSGM|LDC|LSCF-GDC	LSGM (~200)	97% CH_4+3% H_2O	800<br>750<br>700	655<br>465<br>251	80<br>(800 ℃)	28
$Ba_{0.6}La_{0.4}Fe_{0.8}Mo_{0.1}N_{i0.1}O_{3-\delta}$|LSGM|LSCF	LSGM (~300)	100% CH_4	800<br>750	203<br>76	325<br>(800 ℃)	29
HEP|GDC|SSZ|GDC|LSCF	SSZ (~300)	97% CH_4+3% H_2O	600<br>650<br>700	377.8<br>545.1<br>774.5	1000<br>(700 ℃)	This Work
PBF|GDC|SSZ|GDC|LSCF	SSZ (~300)	97% CH_4+3% H_2O	600<br>650<br>700	157.2<br>230.1<br>345.8	—	This Work

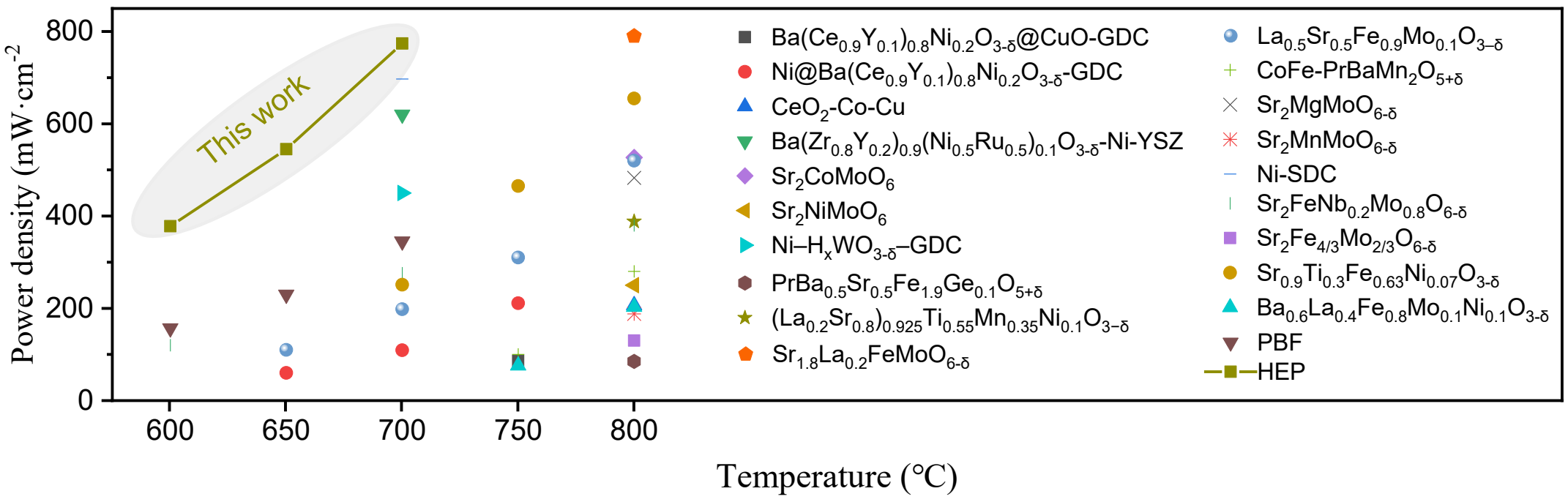

Fig. R1 (also **Fig. 3c**) Comparison of the PPDs of the SOFC technology with different anodes using methane as the fuel.

Reviewer #2

The manuscript entitled 'Entropy-Driven Order-to-Disorder Transition in Perovskite Anodes for High-Performance Solid Oxide Fuel Cells' presents an impressive and conceptually innovative study. The authors introduce a high-entropy strategy to break the layered A-site cation ordering, demonstrating that configurational entropy can effectively drive a transition from an ordered double perovskite to a fully disordered cubic perovskite which exhibits enhanced electrochemical activity. The manuscript is well written, the experiments are comprehensive and rigorous, and the conceptual insights are highly relevant to the SOFC community. Therefore, I would like to recommend it for publication after a minor revision.

Response to Reviewer #2:

We express our sincere gratitude to the reviewer for providing us with detailed and insightful comments. These comments have greatly contributed to enhancing the quality of this work. We have made every effort to address these comments in the revised version of our manuscript and the modifications have been marked in green in the revised manuscript. Please find our responses to the comments point by point as follows.

Comment 2-1

Please keep the font consistent in the images, for example, EDS elements in Fig. 1c and 1d.

Response to 2-1:

We thank the reviewer for highlighting this inconsistency in the figure formatting.

We have now revised **Fig. 1c** and **1d** to ensure that the font styles (including those for the EDS element labels) are consistent as shown in **Fig. R5** (also **Fig. 1c, d**). Furthermore, we have checked and standardized the font usage across all figures in the manuscript to maintain a uniform presentation style.

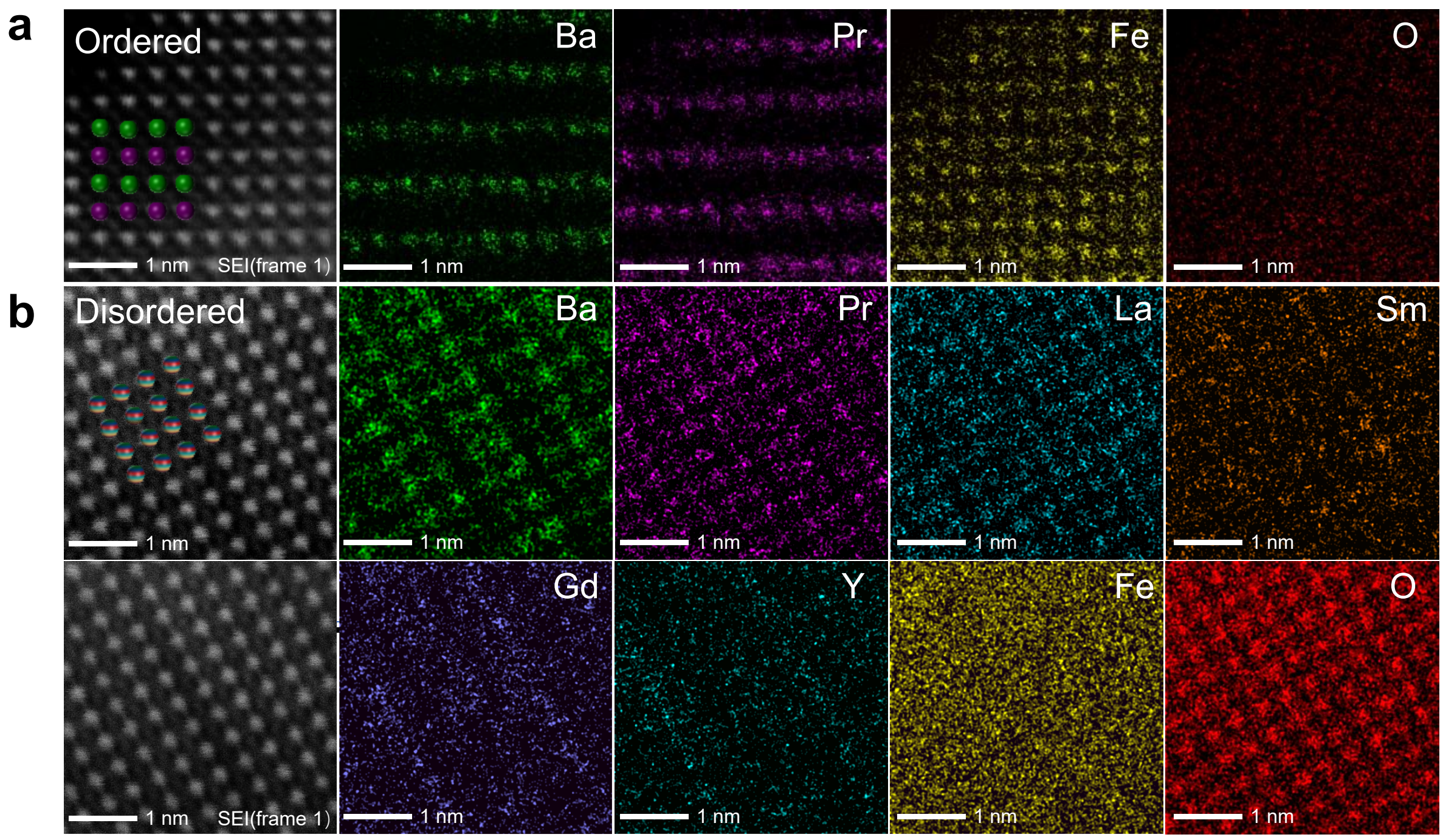

Fig. R5 (also **Fig. 1c, d**) HAADF-STEM elemental maps of (**a**) PBF and (**b**) HEP.

Comment 2-2

In Fig. 2b, please list the g-factor signal center to clarify the vacancy species.

Response to 2-2:

Thank you for this constructive comment.

In response, we have now clearly labeled the g-factor centers of the prominent signals in the revised **Fig. R6** (also **Fig. 2b**). The observed signals at g = 2.003 are characteristic of oxygen vacancies. Providing these quantitative g-factors offers more definitive evidence for the nature of the vacancy species generated, strengthening our structural analysis.

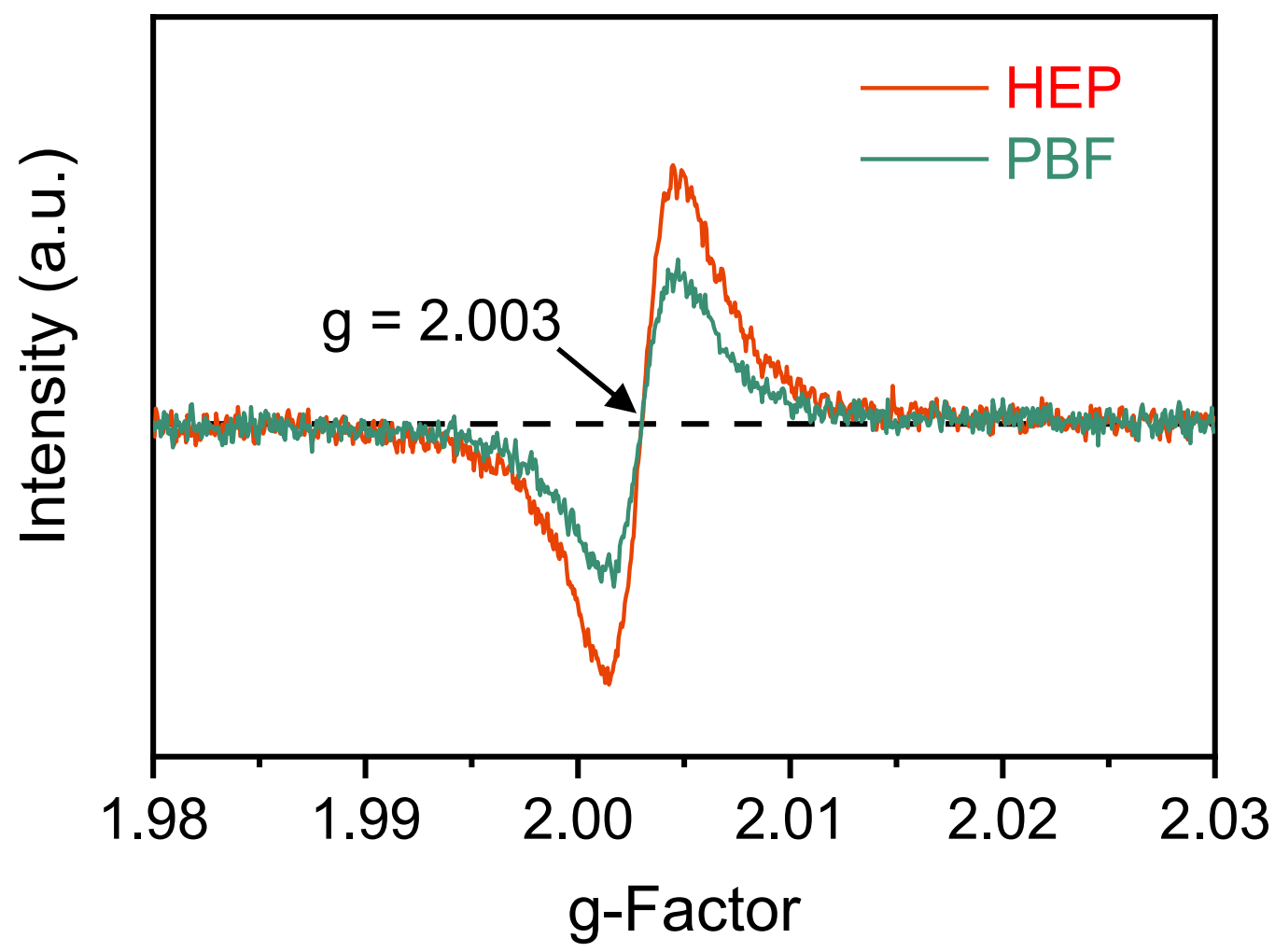

Fig. R6 (also **Fig. 2b**) EPR plots of PBF and HEP.

Comment 2-3:

Please add intensity scale of Wavelet transforms of the k3-weighted Fe K-edge EXAFS spectra of Fig. 2e PBF and Fig. 2f HEP.

Response to 2-3:

We thank the reviewer for this suggestion to improve the data presentation.

In the revised manuscript, we have added a clearly labeled, color-coded intensity scale bar adjacent to the Wavelet transform plots in **Fig. R7** (**Fig. 2e, f**). This addition provides the necessary quantitative information regarding the magnitude of the EXAFS signals in the wavelet space, allowing for a more accurate visual and quantitative comparison between the two samples.

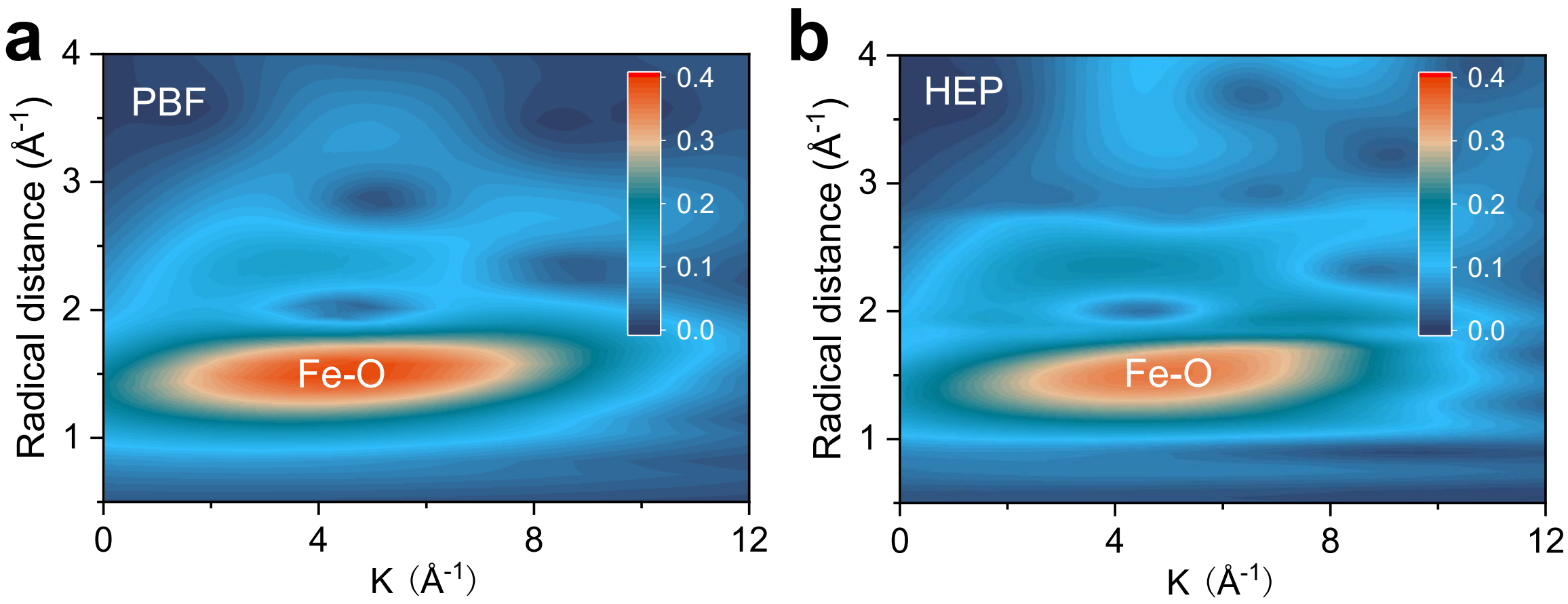

Fig. R7 (also **Fig. 2e, f**) Wavelet transforms of the k^3-weighted Fe K-edge EXAFS spectra of (**a**) PBF and (**b**) HEP.

Comment 2-4

Please standardize the notation in the manuscript and figures. For example, the minus sign appears on page 11: 'The results show that the oxygen ion bulk diffusion coefficient (D_{chem}) increases from 6.51 × 10^{-6} cm^2 s^{-1} for PBF to 3.51 × 10^{-5} cm^2 s^{-1} for HEP (Fig. S10), reflecting the inherently fast oxygen-ion diffusion nature in the disordered HEP.' On pages 13: '774.53, 545.14, 377.77, and 136.19 mW cm^{-2}'.

Response to 2-4:

We sincerely thank the reviewer for their meticulous attention to the manuscript's formatting details.

We acknowledge the inconsistency in notation, particularly regarding the presentation of minus signs in superscripts (e.g., × 10^{-6}) versus inline hyphens (e.g., cm^{-2}). We have now standardized the notation throughout the text, figures, and captions. Specifically, all mathematical expressions and units (such as diffusion coefficients and power densities) now consistently use superscript minus signs (e.g., cm^2 s^{-1}, mW cm^{-2}), while en dashes/hyphens are reserved for text ranges and compound adjectives. This revision ensures professional and consistent typography across the entire manuscript.

The corresponding labels have been added in the revised manuscript (green-highlighted in Line 346-348) as follows:

The cell demonstrates remarkable power output across the tested temperature range, achieving peak power densities of 774.53, 545.14, 377.77, and 136.19 mW·cm^{-2} at 700, 650, 600, and 550 °C, respectively.

Comment 2-5

The hydration behavior of HEP is clearly superior to PBF, as shown by TGA and in situ Raman. It would be helpful to add a short paragraph explicitly linking the higher oxygen-vacancy concentration, Fe electronic structure, and local coordination environment to the faster water absorption and dissociation kinetics.

Response to 2-5:

We thank the reviewer for this constructive suggestion to strengthen the mechanistic discussion.

We fully agree that an explicit connection between the atomic-scale properties and the macroscopic hydration kinetics would enhance the manuscript. Accordingly, we have added a new paragraph in the revised manuscript (line 313-321) that directly links the higher oxygen-vacancy concentration, altered Fe electronic structure and coordination environment to the superior water absorption and dissociation kinetics of HEP. This addition provides a clearer and more integrated narrative on the structure-activity relationship.

Related discussions (green-highlighted in Line 313-321) have been added in the revised manuscript as follows:

Based on the above analysis, the enhanced hydration capability of HEP can be attributed to the following two factors. First, the order-to-disorder transition promotes the generation of a higher concentration of oxygen vacancies, which provide more active adsorption sites for water molecules. Second, the order-to-disorder transition modifies coordination environment of Fe and induces an upshift of the Fe d-band center toward the Fermi level, rendering Fe sites more electron-deficient and thus strengthening their interaction with the electron-donating oxygen atoms in water. Accordingly, the synergistic effect of increased oxygen vacancy concentration, favorable local coordination, and optimized electronic structure collectively contributes to the superior hydration reactivity of disordered HEP relative to ordered PBF.

Comment 2-6

Please standardize the coordinate format in the images, for example, the x and y coordinates in Fig. 4e are Reaction coordinates and Relative Energy.

Response to 2-6:

We thank the reviewer for pointing out the inconsistency in the axis label formatting.

We have standardized the coordinate labels in **Fig. R8** (also **Fig. 4e**) (and across all figures) to ensure a uniform presentation style. Specifically, the font style, size, and alignment of the x-axis ("Reaction coordinate") and y-axis ("Relative energy") labels have been made consistent.

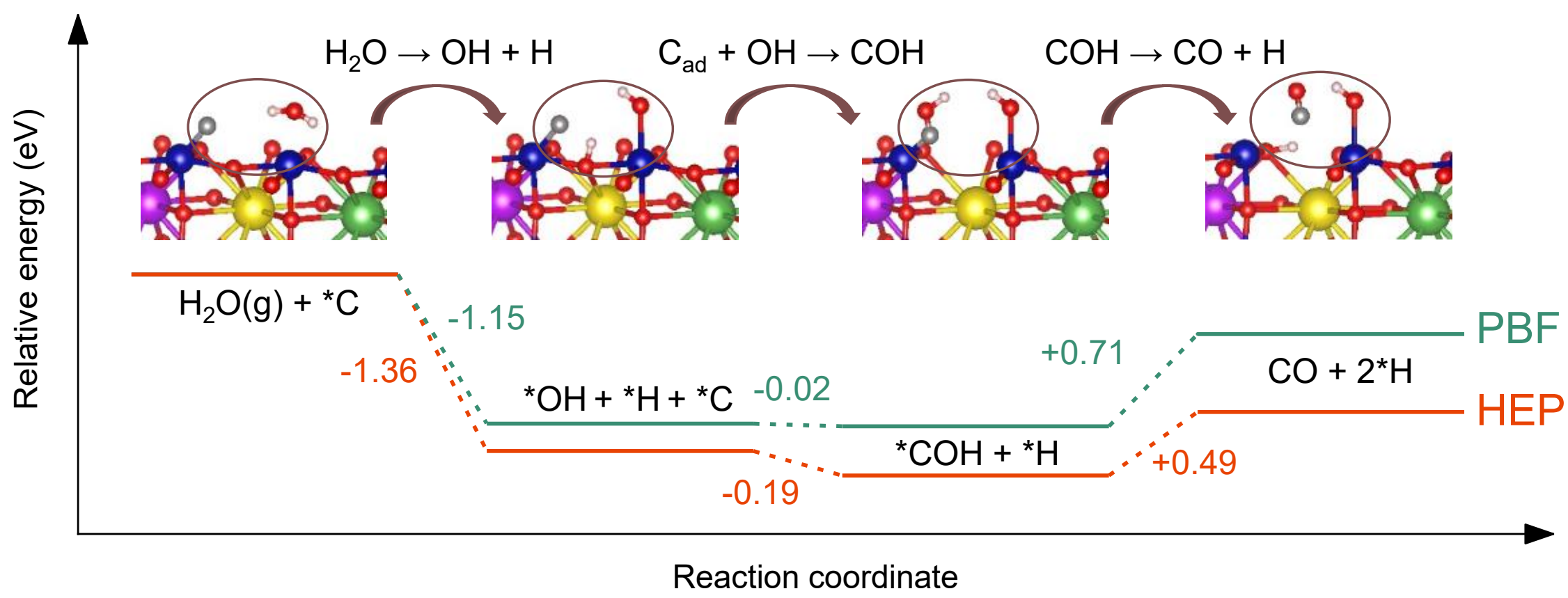

Fig. R8 (also **Fig. 4e**) Water-assisted removal of carbon from the PBF and HEP interface (carbon atom is labeled by grey color). The energy of water adsorption with formation of intermediate surface species (*OH and *COH) and final CO desorption are presented.

Comment 2-7

The manuscript frequently refers to operation under "nearly dry methane" with 3 vol% H_2O. How the significance of the 3% H_2O condition and the robustness of the anode under very low steam contents?

Response to 2-7:

Thank you for your meticulous and professional revision suggestions. We fully agree that justifying the selected condition and demonstrating stability under such is crucial for evaluating practical applicability. To avoid any ambiguity or misunderstanding, all descriptions involving "near-dry methane" in the manuscript have been revised to "wet methane (3 vol% H_2O)". Our choice of 3 vol% H_2O is based on the following considerations:

1. Easily achievable moisture content condition:

From a practical experimental standpoint, this concentration is readily and reproducibly achieved by bubbling the methane fuel stream through a water saturator maintained at room temperature (approximately 25 °C). Given that the saturated vapor pressure of water at this temperature is about 3.17 kPa, the effluent gas achieves a near-equilibrium water vapor content of ~3 vol% at atmospheric pressure. This method provides a simple, stable, and cost-effective means of humidifying the fuel without requiring complex, precision-controlled vapor delivery systems. It significantly simplifies the experimental setup, lowers operational costs, and improves the reproducibility of the fuel atmosphere. This represents an important factor for fundamental laboratory studies that seek to establish a consistent baseline for performance comparison.

2. A Prevalent Benchmark for Assessing Anti-Coking Performance:

The use of methane fuel containing approximately 3 vol% H_2O is widely adopted and reported in numerous key studies focusing on direct hydrocarbon oxidation anodes [e.g., Zhang et al. *Adv. Energy Mater.* 2022, 12, 2202928; Lv et al. *Renew. Energy* 150 (2020) 334e34; Chen et al. *Nat. Energy* **3**, 1042-1050 (2018)]. This condition is recognized as a critical threshold that effectively creates a "near-dry" environment, rigorously testing an anode's intrinsic ability to resist carbon deposition with minimal steam assistance. By employing this commonly used

condition, we ensure that the superior electrochemical stability and carbon tolerance of our HEP anode, as demonstrated in Fig.4a and S18, can be directly and fairly evaluated against the performance metrics of other state-of-the-art materials reported in the literature.

Regarding the anode's robustness under very low steam content:

The long-term stability test presented in **Fig. R9a** was conducted precisely under this challenging 3% H_2O-CH_4 atmosphere for over 1000 hours. The stable voltage output and the post-test characterizations (**Fig. R9b, c, d**) confirming the absence of carbon deposits provide direct experimental evidence for the exceptional structural and operational robustness of the HEP anode under low steam content. This demonstrates not just initial activity, but sustained stability under the harsh conditions relevant to practical application.

In summary, the 3% H_2O condition serves as a meaningful, stringent, and mechanistic benchmark that critically underscores the technological advantage and fundamental insights provided by our HEP material.

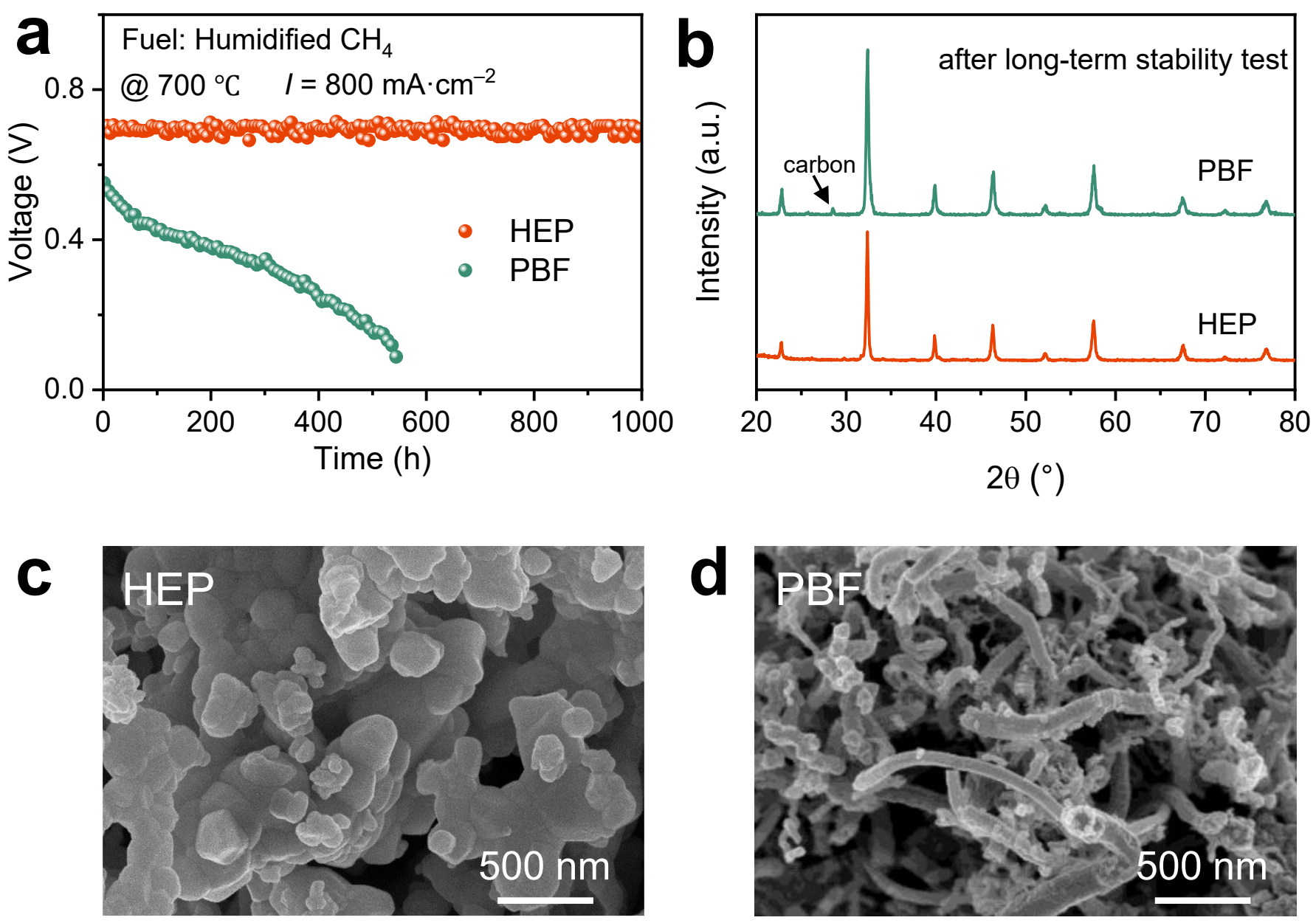

Fig. R9 (also **Fig. 4a, b, c** and **Supplementary Fig. 39**) **a** Long-term stability at the constant current density 800 mA cm^{-2} at 700 °C. **b** XRD patterns of HEP and PBF anodes after long-term stability test. Cross-sectional SEM images of **c**) HEP and **d**) PBF anode after 1000 h durability testing under 800 mA cm^{-2} at 700 °C.

Related discussions have been added in the revised manuscript as follows:

Line 327-329: This fuel composition was chosen mainly because it is readily accessible and easy to control, as the saturated vapor pressure of water at room temperature is close to 3%.

Line 437-439: Moreover, XRD analysis of the post-test HEP sample (Fig. S39) revealed a monophasic structure, indicating excellent structural stability of HEP under the testing conditions.

Comment 2-8

Please explain the generality of the entropy-driven strategy and discuss how the proposed entropy-driven order-to-disorder transition might be extended to other perovskites.

Response to 2-8:

We sincerely thank the reviewer for this insightful and constructive comment.

To demonstrate its generality in layered double perovskite oxide beyond the reported Fe-based system, we have successfully extended this strategy to other members of the layered double perovskite family, specifically the Co-based $PrBaCo_2O_{5+\delta}$ (PBC) and Mn-based $PrBaMn_2O_{5+\delta}$ (PBM) materials. We synthesized their high-entropy derivatives, $La_{0.2}Pr_{0.2}Sm_{0.2}Gd_{0.2}Y_{0.2}BaCo_2O_{5+\delta}$ (LPSGYBC) and $La_{0.2}Pr_{0.2}Sm_{0.2}Gd_{0.2}Y_{0.2}BaMn_2O_{5+\delta}$ (LPSGYBM). Comprehensive structural characterizations via XRD, HAADF-STEM imaging, and elemental mapping (**Fig. R10** and **R11**) confirm that while the parent PBC and PBM exhibit the characteristic A-site cation-ordered layered structure, their high-entropy counterparts (LPSGYBC and LPSGYBM) display a homogeneous, disordered A-site cation distribution. This confirms the successful induction of an entropy-driven order-to-disorder transition in these distinct perovskite systems.

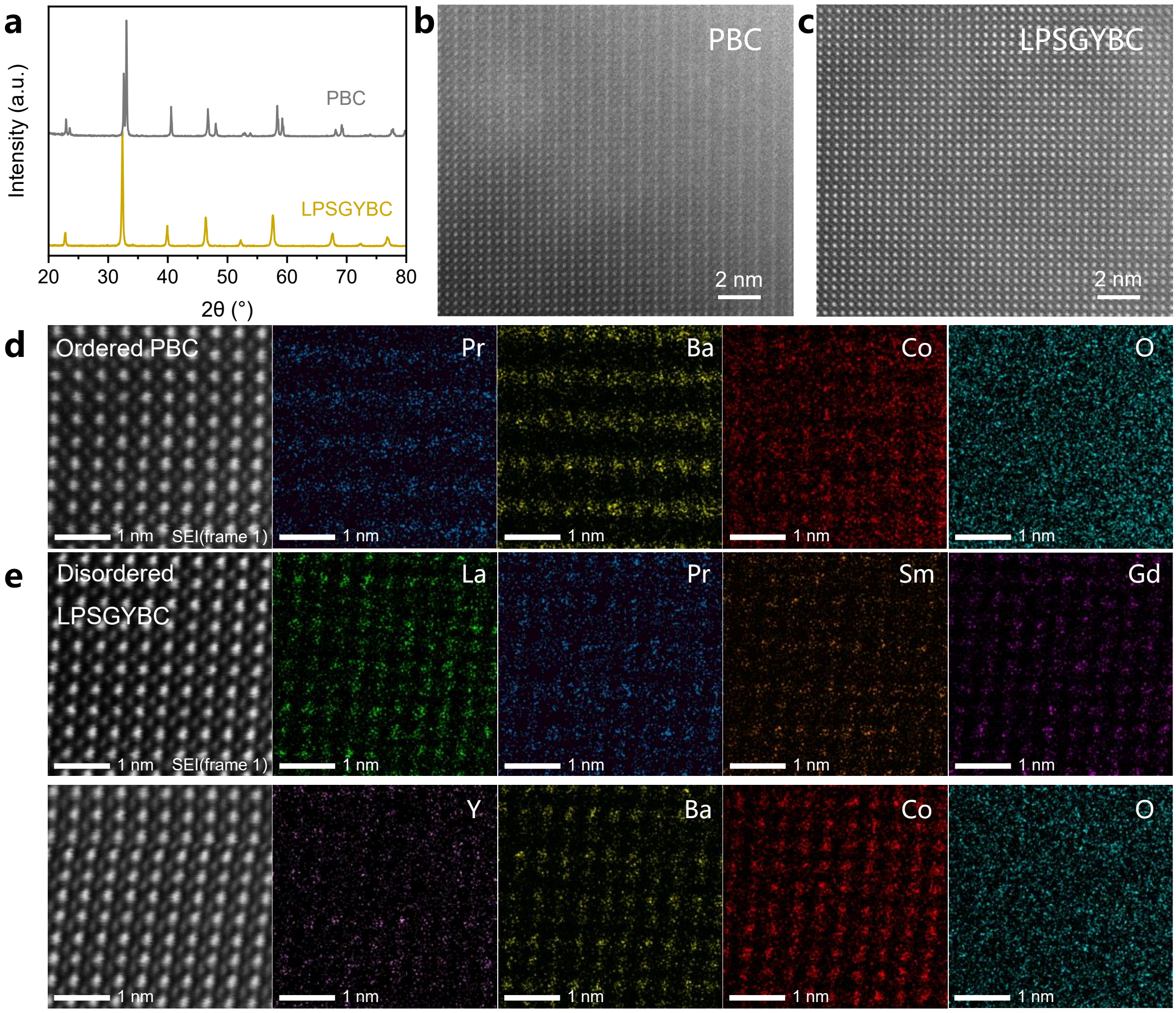

Fig. R10 (also **Fig. S10,11, 12**). **a** XRD patterns at RT of PBC and LPSGYBC. HAADF-STEM images of (**b**) PBC and (**c**) LPSGYBC. HAADF-STEM elemental maps of (**d**) PBC and (**e**) LPSGYBC

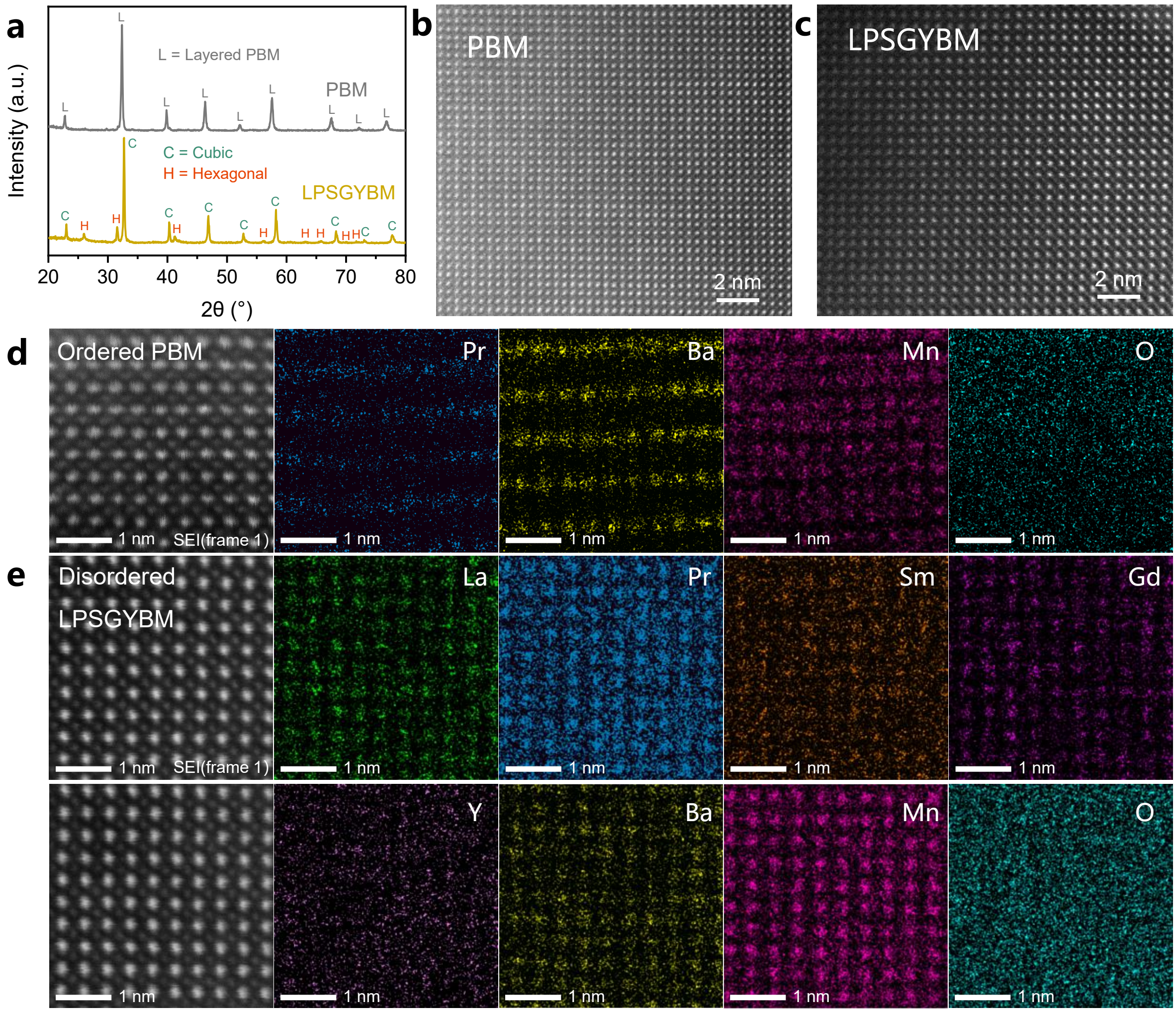

Fig. R11 (also **Fig. S13, 14, 15**) **a** XRD patterns at RT of PBM and PSGYBM after reduction in H_2. HAADF-STEM images of (**b**) PBM and (**c**) LPSGYBM. HAADF-STEM elemental maps of (**d**) PBM and (**e**) LPSGYBM

Related discussions have been added in the revised manuscript as follows:

Line 205-217: In addition, to verify the generality of the entropy-driven strategy, we extended the high-entropy design to A-site layered perovskite materials $PrBaCo_2O_{5+\delta}$ (PBC) and $PrBaMn_2O_{5+\delta}$ (PBM), synthesizing the high-entropy compositions $La_{0.2}Pr_{0.2}Sm_{0.2}Gd_{0.2}Y_{0.2}BaCo_2O_{5+\delta}$ (LPSGYBC) and $La_{0.2}Pr_{0.2}Sm_{0.2}Gd_{0.2}Y_{0.2}BaMn_2O_{5+\delta}$ (LPSGYBM). As shown in the XRD patterns (Fig. S10), the characteristic split peaks of layered PBC merged and vanished in LPSGYBC, resulting in a single perovskite phase. Similarly, a structural transition from the layered phase of PBM to a disordered mixed phase

(cubic/hexagonal) was observed in LPSGYBM (Fig. S13).[23] Furthermore, HAADF-STEM images (Fig. S11 and S14) revealed alternating bright and dark contrasts indicative of a layered structure in PBC and PBM, which disappeared in their high-entropy counterparts. EDS elemental mapping (Fig. S12 and S15) further confirmed the cationic disorder in LPSGYBC and LPSGYBM. These results collectively demonstrate that the entropy-driven strategy is also applicable to other layered perovskite systems.

Comment 2-9

Fig.3f, what's the temperature? no description for fig.3g and h.

Response to 2-9:

Thank you for pointing out these omissions.

We have now added the corresponding descriptions in the revised manuscript. We apologize for any confusion caused by these incomplete descriptions and have carefully checked all figure captions to ensure consistency and clarity throughout the manuscript.

The temperature for the methane conversion rates measurement in **Fig. 3f** is 600 °C and has been clearly indicated in the figure (**Fig. R12**) and the corresponding figure caption (Line 363-364) in the revised manuscript. **Fig. 3g** and **3h** represent the schematic illustrations of the steam reforming mechanism on the HEP and PBF surfaces, respectively, as described in the results section (line 412-417).

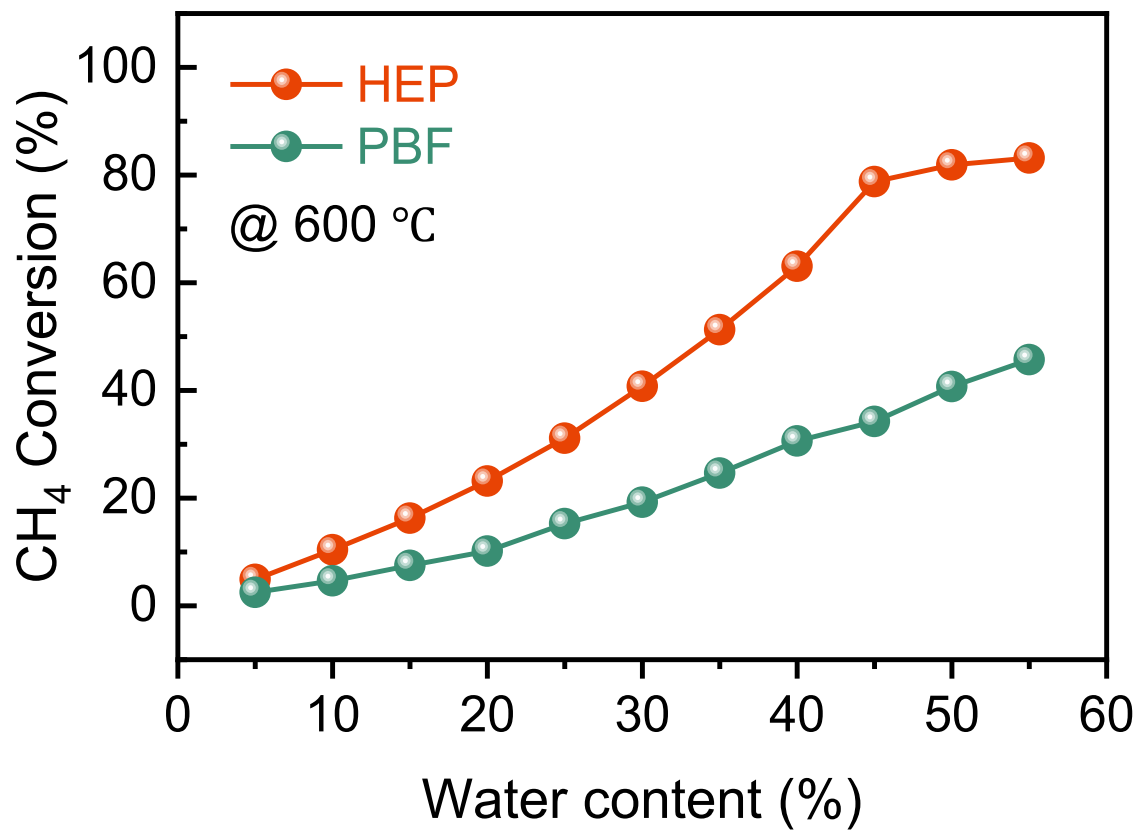

Fig. R12 (also **Fig. 3f**) Comparison of methane conversion rates under different water contents at 600 °C.

The specific revisions have been added in the revised manuscript as follows:

Line 363-364: **f** Comparison of methane conversion rates under different water contents at 600 °C.

Line 388-391: As demonstrated in Fig. 3f, when the temperature is held constant at 600°C, both PBF and HEP exhibit an increase in methane conversion with rising water vapor pressure, indicating that hydration plays a promoting role in methane steam reforming.

Line 412-417: Based on the above analysis, the mechanism for the enhanced performance is

illustrated in Fig. 3g and h. Compared to PBF, the HEP features stronger hydration, which facilitates the chemical reforming of methane to generate more CO and H_2. Additionally, the higher bulk oxygen ion diffusion coefficient D_{chem} in HEP enables fast reaction of oxygen ions with CO and H_2. Consequently, HEP exhibits a much higher peak power density.

Comment 2-10

In Fig.4a, it is interesting to observe the gas composition before and during the test is interesting, as it helps us understand whether the additional current load will consume only CO and H_2 or methane, and how much.

Response to 2-10:

We thank the reviewer for this insightful comment regarding gas consumption under current load. We agree that analyzing the gas composition is crucial for understanding the electrochemical oxidation process at the anode. In direct response to this suggestion, we have conducted a dedicated short-term stability test under current load and performed online gas chromatography (GC) analysis of the effluent gas.

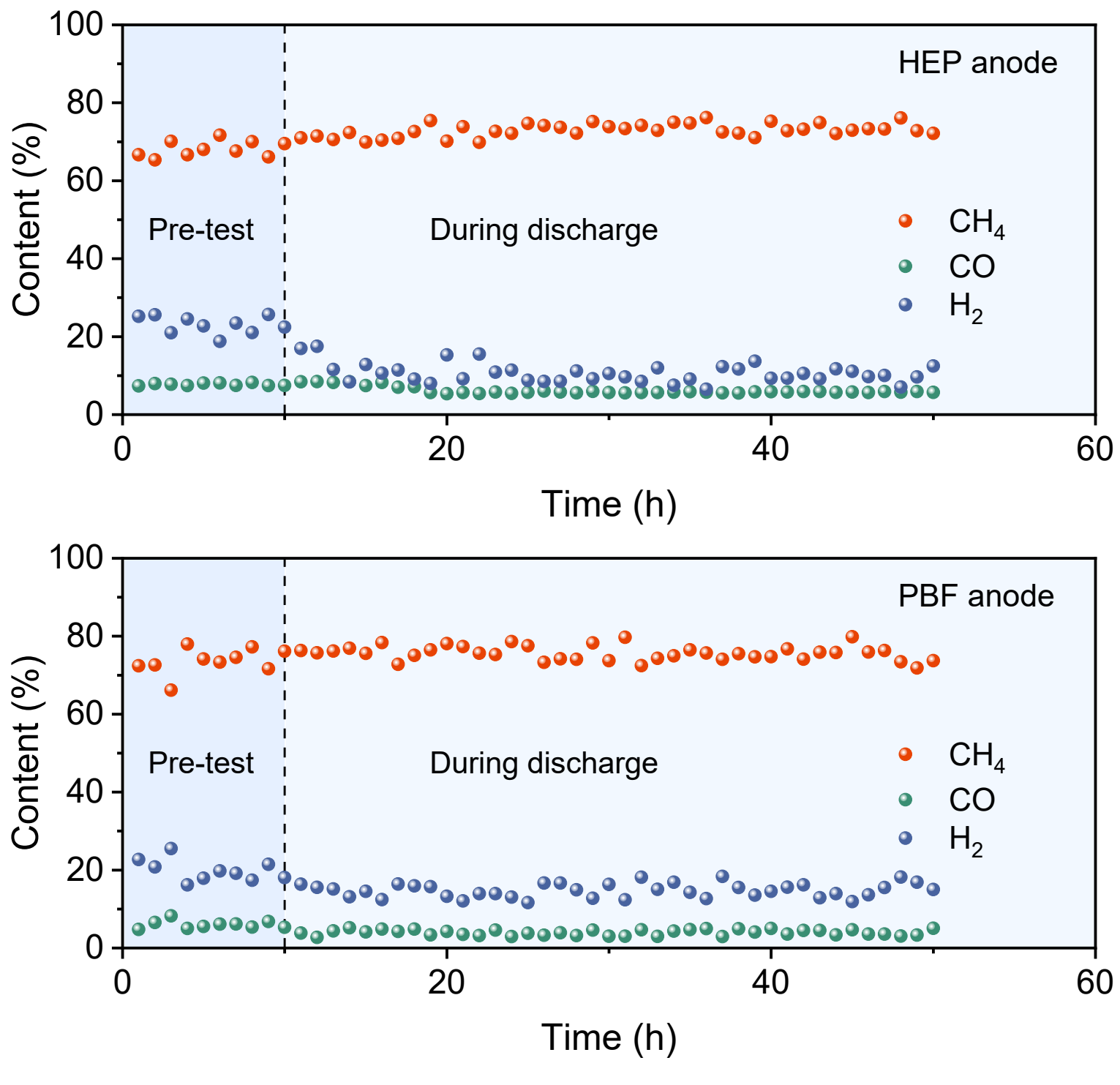

Fig. R13 (also **Fig. S38**) Different gas component contents during stability testing process.

As shown in **Fig. R13**, the exhaust flow rates of the three main gas components (CH_4, CO, H_2) were recorded. Prior to current loading, the effluent composition reached a near-steady state for both HEP and PBF cells, governed by the catalytic reforming and cracking of methane. Upon applying the current load, a pronounced and immediate decrease in the flow rates of CO

and H_2 was observed, indicating their electrochemical consumption at the anode. In contrast, the CH_4 flow rate remained largely unchanged, and even exhibited a slight relative increase due to the significant depletion of CO and H_2, demonstrating that methane was not directly electrochemically oxidized to a substantial extent.

This result provides direct experimental evidence that the additional current load during the operation primarily consumes the reformed syngas products (H_2 and CO), rather than methane itself. This aligns with the prevailing reaction pathway where methane is first catalytically reformed on the anode surface, with the resulting H_2 and CO then serving as the primary fuels for electrochemical oxidation.

Related discussions (green-highlighted in Line 426-432) have been added in the revised manuscript as follows:

To further elucidate the reaction mechanism, we analyzed the exhaust gas composition before and after applying a constant current load during the stability test (Fig. S38). Specifically, the methane concentration remained constant upon current loading, while H_2 and CO were rapidly consumed, confirming that methane is primarily converted via steam reforming rather than direct electrochemical oxidation. Notably, the HEP-based cell demonstrated a faster consumption rate of H_2 and CO compared to the PBF-based cell, providing additional evidence for the enhanced electrochemical activity and stability of the HEP anode.

Reviewer #3

The manuscript addresses the development of a Ni-free oxide fuel electrode for solid oxide cells, a topic of clear relevance given the limitations of conventional NiO-8YSZ systems, particularly for hydrocarbon operation and long-term stability. The authors propose an entropy-driven design strategy based on a layered double perovskite, aiming to enhance oxygen vacancy concentration, ionic transport, surface hydration and, ultimately, coking resistance. While the reported electrochemical performance and durability under methane are promising, the work in its current state relies heavily on the current trend of high-entropy materials without providing sufficiently direct and conclusive evidence that entropy itself is the governing factor behind the observed behaviour. As a result, the manuscript does not yet convincingly demonstrate that the proposed material represents an exceptional or fundamentally distinct class of fuel electrodes, rather than a favourable compositional variant within an already explored design space.

In my opinion, for a work published in Nature Communications, conceptual novelty must be supported by direct experimental evidence that rigorously validates the proposed mechanisms, rather than relying on broadly defined concepts such as entropy stabilization. The main critical aspects that need to be addressed to support the central claims of the manuscript are outlined below.

Response to Reviewer #3:

We are deeply grateful to Reviewer #3 for this incisive and constructive overall assessment, which compellingly highlights the need for greater mechanistic rigor in supporting our central claim. We fully agree that for a work in Nature Communications, compelling novelty must be rooted in direct evidence, not just association. The reviewer's critique that we must distinguish a "fundamentally distinct class" from a "favourable compositional variant" is precisely the key point we have now addressed with substantial new experiments and analysis. According to your valuable suggestions, we have carefully and thoroughly revised and improved the manuscript. We hope that these adjustments will further enhance the clarity and scientific rigor of our paper.

Major Comment 3-1

Lack of electrochemical validation: It is surprising that a study focused on the synthesis and characterisation of a new fuel electrode material does not present any EIS data, neither in the main manuscript nor in the Supplementary Information. EIS is a fundamental requirement in the development and validation of new materials for solid oxide cells. The statement in the Supplementary Information that "symmetric cells were prepared for EIS measurements", without presenting any corresponding results, is insufficient at this level. This is not merely a matter of missing data; rather, the mechanistic narrative of the manuscript is constructed in the absence of the standard electrochemical experiment required to validate or refute the proposed interpretations.

Response to 3-1:

We thank the reviewer for this suggestion. As advised, we have added detailed electrochemical impedance spectroscopy (EIS) analysis of the HEP electrode to the revised manuscript and Supplementary Information.

The EIS measurements were performed under operating conditions (3% H_2O-CH_4 atmosphere) at various temperatures using SSZ electrolyte-supported symmetrical cells, as shown in **Fig. R14**. Generally, the difference between the intercepts of the low-frequency arc and the high-frequency arc with the real axis was used as the polarization resistance (R_p) and was taken as a leading parameter to assess the electrochemical performance of the electrodes; a lower R_p corresponds to higher catalytic activity. As evident from **Fig. R14a, b**, the R_p of HEP is consistently and significantly lower than that of PBF across all measured temperatures, indicating that the HEP anode facilitates the relevant surface electrochemical processes more effectively.

To clarify the source of the reduced R_p values, the EIS data were analyzed based on the distribution of relaxation times (DRT). Each DRT curve exhibits three distinct peaks in the low frequency, intermediate frequency, and high-frequency ranges, corresponding to gas diffusion processes, ion surface exchange, and interface charge transfer processes, respectively [Liu et al. *J. Am. Chem. Soc.* 146, 4704-4715 (2024)]. Obviously, the differences in DRT areas are

primarily observed in the intermediate frequency region as shown in **Fig. R14c, d**, suggesting that the ion surface exchange processes in the HEP anode are significantly enhanced. This is primarily attributed to the disordered HEP structure, which increases the oxygen vacancy concentration and thereby promotes the surface exchange process.

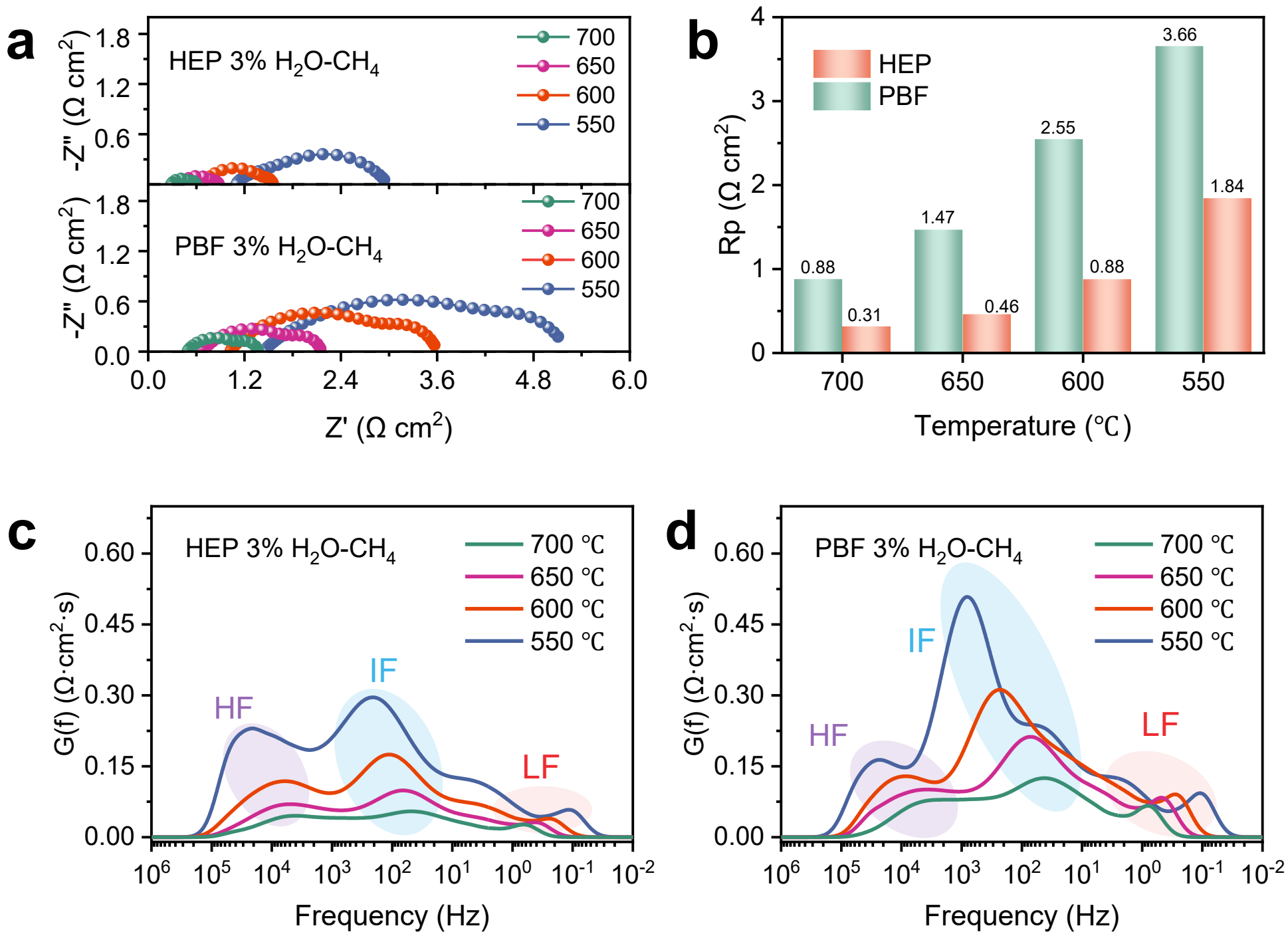

Fig. R14 (also **Fig. S24**) **a** EIS plots of HEP and PBF anodes in 3% H_2O-CH_4. **b** Comparison of EIS using different anodes. DRT fitting results for **c**) HEP and **d**) PBF.

To further verify the enhancement of surface hydration capacity derived from the entropy-driven A-site disordered structure of HEP, we compared the electrochemical impedance of HEP and PBF at 700 °C under CO, H_2, pure CH_4, and 3% H_2O-CH_4 atmospheres, with the detailed results presented in **Fig. R15**. The impedance response of the two anodes showed distinct differences after the introduction of water vapor. In pure methane atmosphere, both HEP and PBF exhibited relatively high impedance values; upon adding 3% H_2O to the methane fuel, the impedance of HEP decreased drastically and its value closely approached the low impedance levels measured under CO and H_2 atmospheres. In sharp contrast, PBF only showed a slight reduction in impedance after the introduction of water vapor, with no obvious tendency to

approach the impedance values under CO and H_2. This result clearly demonstrates that the excellent hydration capacity of HEP is the core reason for its significantly lower impedance in 3% H_2O-CH_4 atmosphere, and further confirms that the entropy-driven A-site disordered structure is the fundamental factor optimizing the key electrochemical properties of the anode such as hydration capacity and methane reforming activity.

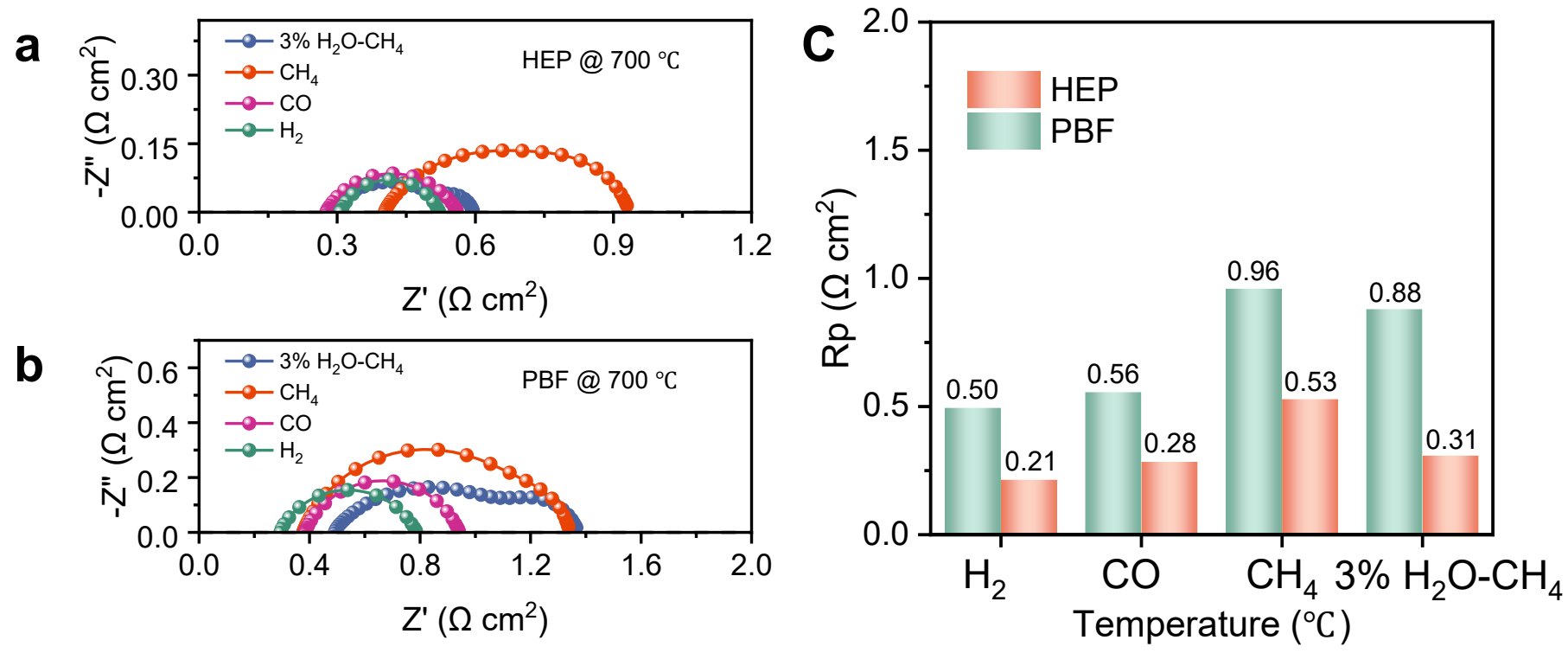

Fig. R15 EIS plots of (**a**) HEP and (**b**) PBF anodes under different atmosphere at 700 °C. **c** Comparison of EIS under different atmosphere at 700 °C for HEP and PBF.

Related discussions (blue-highlighted in Line 323-340) have been added in the revised manuscript as follows:

Line 323-340: To elucidate the influence of structural disorder on the electrochemical performance of anode materials, electrochemical impedance spectroscopy (EIS) was first employed to characterize HEP and PBF. EIS measurements were carried out on SSZ electrolyte-supported symmetrical cells under realistic operating conditions (3% H_2O-CH_4 atmosphere) at various temperatures. This fuel composition was chosen mainly because it is readily accessible and easy to control, as the saturated vapor pressure of water at room temperature is close to 3%. As presented in Fig. S24a, the HEP anode shows consistently and remarkably lower polarization resistance (R_p) than the PBF anode over the entire temperature range, demonstrating the outstanding potential of disordered HEP as a highly active anode material. A detailed performance comparison is provided in Fig. S24b. Notably, for the two anodes prepared under

similar conditions with comparable specific surface areas (Fig. S25), the observed differences in electrochemical properties can be attributed to the intrinsic nature of the samples themselves.

To further understand the effect of the order-to-disorder transition on electrochemical performance, we also compared the EIS spectra of perovskites with different entropy values (LPBF, LPSBF, and LPSGBF). As shown in Fig. S26, the results reveal that disordered HEP exhibit the lowest Rp values. This indicates that anodes with a disordered structure possess superior electrochemical properties.

Side/related concerns:

3-1-(I)

Electrode sintering and attachment: The same sintering conditions are applied to both electrodes (1100 ºC 2 h.) without demonstrating comparable attachment or interfacial quality. Without EIS or symmetric-cell data, differences in contact resistance cannot be excluded.

Response to 3-1-(I):

We thank the reviewer for raising this valid concern regarding electrode sintering and interfacial quality. We agree that applying the same sintering conditions (1100 °C for 2 h) does not, by itself, guarantee comparable electrode attachment or exclude differences in contact resistance. To address this point directly, we have now supplemented the manuscript with the following key data:

1. Electrochemical Impedance Spectroscopy (EIS)

As fully presented in **Fig. R14 and R15**, we provide the EIS plots and corresponding distribution of relaxation times (DRT) analysis for both the HEP and PBF electrodes under symmetric cell configuration in different atmospheres. The obtained polarization resistances serve as a direct, quantitative measure of the total electrode performance, which intrinsically includes the contributions from both the electrochemical reaction kinetics and the interfacial contact quality. The clear and consistent semicircles in the spectra, along with the low series resistances, indicate that both electrodes achieved good adhesion and comparable, high-quality interfaces with the electrolyte under the optimized sintering conditions.

2. Microstructural Evidence

We have added cross-sectional SEM images of the full single cells (**Fig. R16 and R17**), which clearly show the dense, continuous, and well-bonded interfaces for both the cathode and the HEP anode after co-sintering. No delamination or significant structural defects are observed at the electrode-electrolyte interfaces.

Collectively, these new results directly demonstrate that the applied sintering protocol is effective for both materials, resulting in comparable and high-quality electrode attachment. This allows us to confidently attribute the differences in the measured polarization resistances

primarily to the intrinsic electrochemical activity of the electrode materials, rather than to variations in interfacial contact resistance.

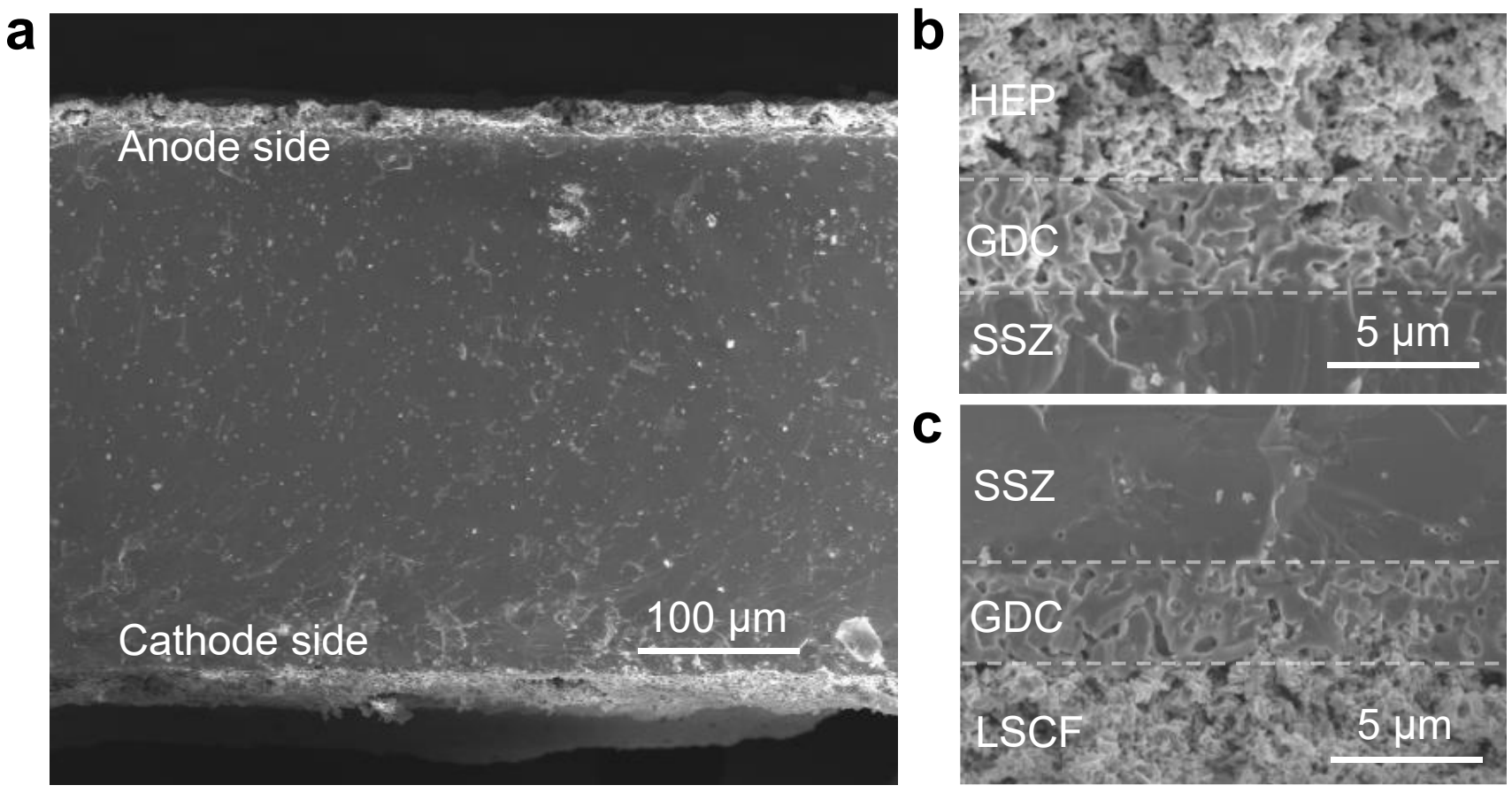

Fig. R16 (also **Fig. S27**). **a** Cross-Wsectional view of the SSZ-based single cell with a configuration of HEP|GDC|SSZ|GDC|LSCF. **b** Magnified view of the anode side (HEP|GDC|SSZ). **c** Magnified view of the cathode side (SSZ|GDC|LSCF).

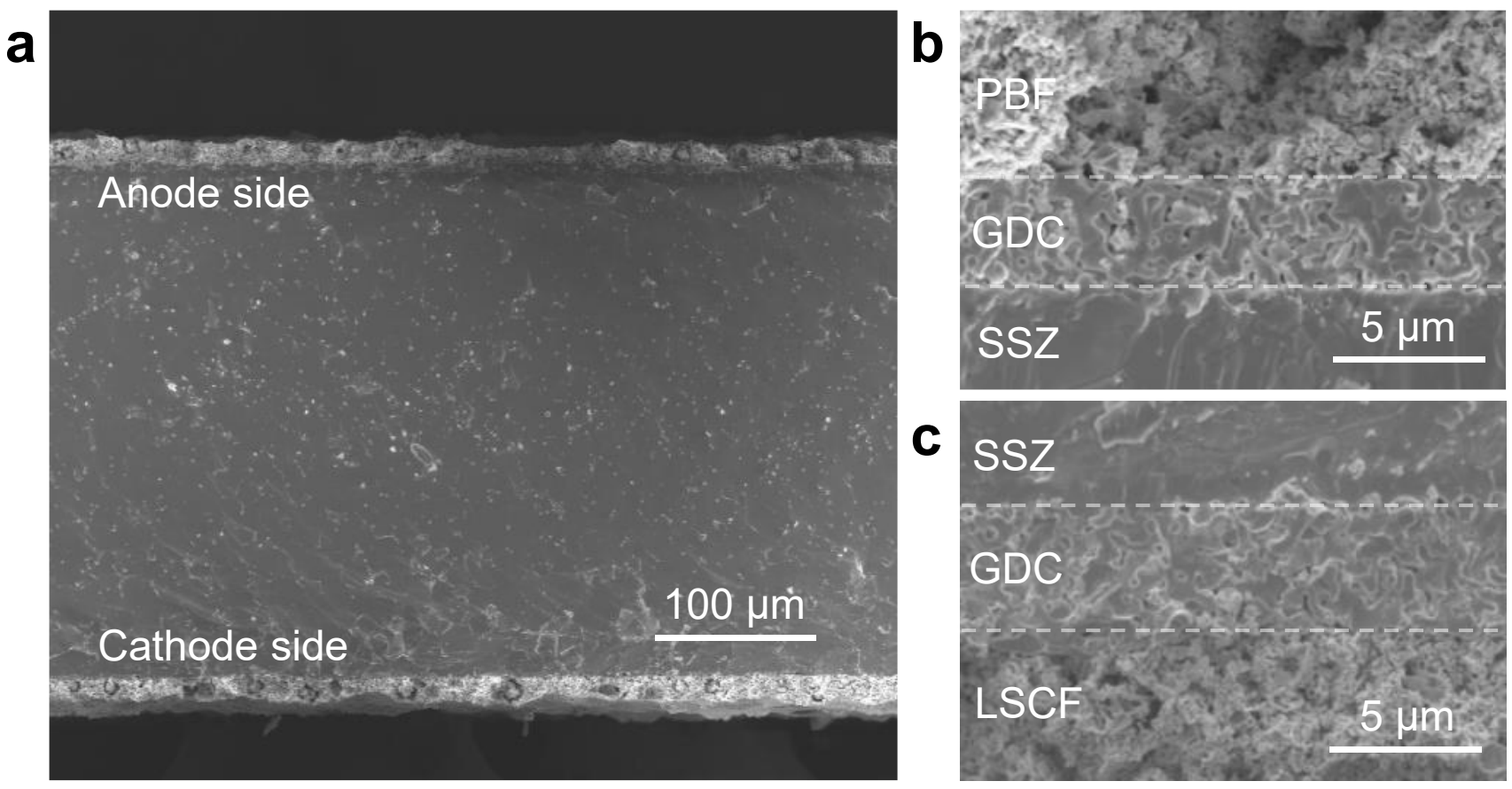

Fig. R17 (also **Fig. S29**). **a** Cross-Wsectional view of the SSZ-based single cell with a configuration of PBF|GDC|SSZ|GDC|LSCF. **b** Magnified view of the anode side (PBF|GDC|SSZ). **c** Magnified view of the cathode side (SSZ|GDC|LSCF).

Related discussions (blue-highlighted in Line 341-343 and 348-349) have been added in the revised manuscript as follows:

Line 341-343: The output performance of the disordered HEP anode was evaluated in an electrolyte-supported SOFC with a dense SSZ electrolyte layer (~300 μm) and a porous LSCF

($La_{0.6}Sr_{0.4}Co_{0.2}Fe_{0.8}O_{3-\delta}$) cathode (Fig. S27).

Line 349-350: In contrast, the power density of the ordered PBF-based anode under identical cell configuration (Fig. S29)

3-1-(II)

Microstructural and interfacial effects: In the absence of impedance deconvolution, it is not possible to distinguish intrinsic fuel-electrode improvements from microstructural or interfacial contributions within the multilayer cell.

Response to 3-1-(II):

We sincerely appreciate the reviewer for raising this critical point.

We fully agree that deconvoluting the impedance spectrum is essential to distinguish intrinsic electrode activity from other contributions. In direct response, we have conducted a detailed Distribution of Relaxation Times (DRT) analysis and incorporated a comprehensive discussion of the results in the revised manuscript. As shown in **Fig. R18** (previously mentioned in **Fig. R14**), the DRT plots for both HEP and PBF electrodes, measured in 3% H_2O-CH_4 between 550-700 °C, reveal three distinct peaks. These are conventionally assigned to: (i) the gas diffusion process (low-frequency, LF, <10 Hz), (ii) processes involving ion migration and surface exchange (intermediate-frequency, IF, 10-2000 Hz), and (iii) the charge transfer process (high-frequency, HF, >2000 Hz) [Niu et al., Adv. Energy Mater. 12, 2103783 (2022); He et al., Adv. Mater. 35, e2209469 (2023)]. Critically, the IF region contributes dominantly to the total polarization resistance, indicating that the electrode performance is primarily governed by kinetics related to surface exchange (e.g., adsorption/dissociation of CH_4 and H_2O) and bulk ion transport. The significantly smaller peak area in the IF region for the HEP anode, compared to PBF, provides direct evidence for its superior surface catalytic activity and oxygen-ion bulk diffusion capability. This deconvolution effectively isolates and highlights the intrinsic electrochemical improvements of the HEP material, substantiating that its exceptional performance stems from enhanced reaction kinetics rather than microstructural or interfacial advantages.

We believe this added analysis directly addresses your concern and strengthens the mechanistic narrative of our work. Thank you again for prompting this valuable clarification.

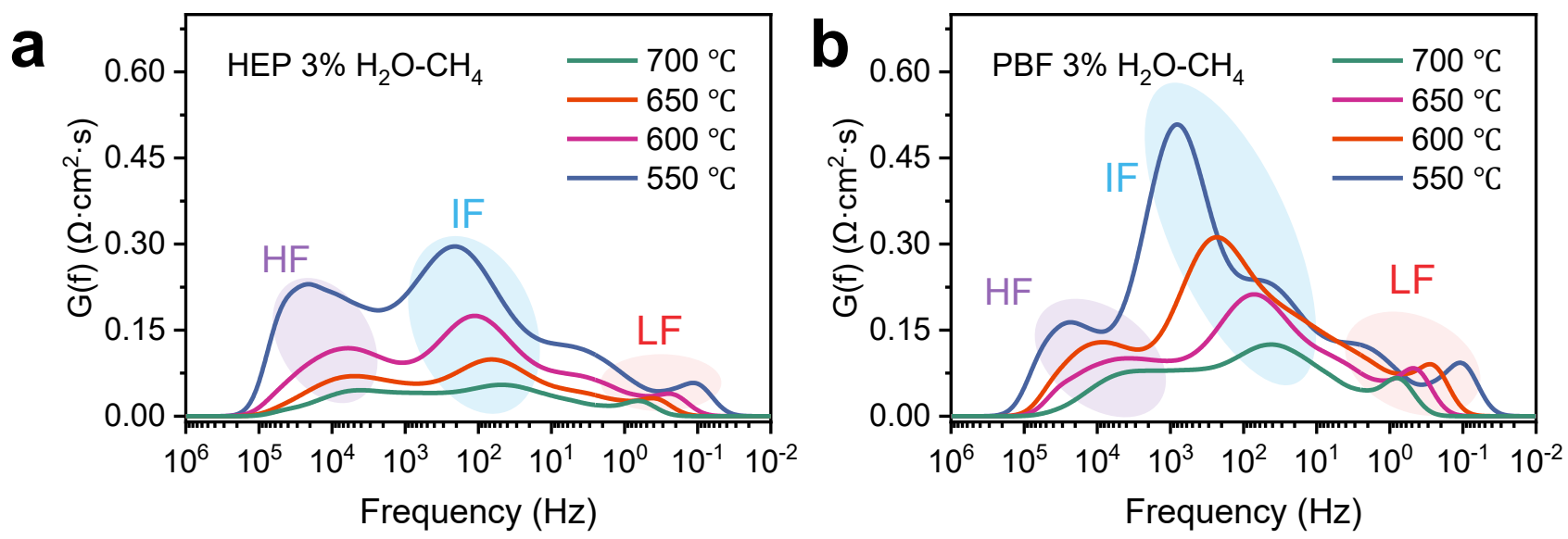

Fig. R18 DRT fitting results for (**a**) HEP and (**b**) PBF anodes.

3-1-(III)

Reproducibility: The lack of EIS data prevents assessment of cell-to-cell variability and robustness of the reported performance.

Response to 3-1-(III):

We sincerely thank the reviewer for raising this critical point regarding reproducibility. We fully agree that evaluating cell-to-cell variability is essential for validating the robustness of any new electrode material. We recognize that our initial omission of EIS data was a significant oversight, and we are grateful for the opportunity to address it.

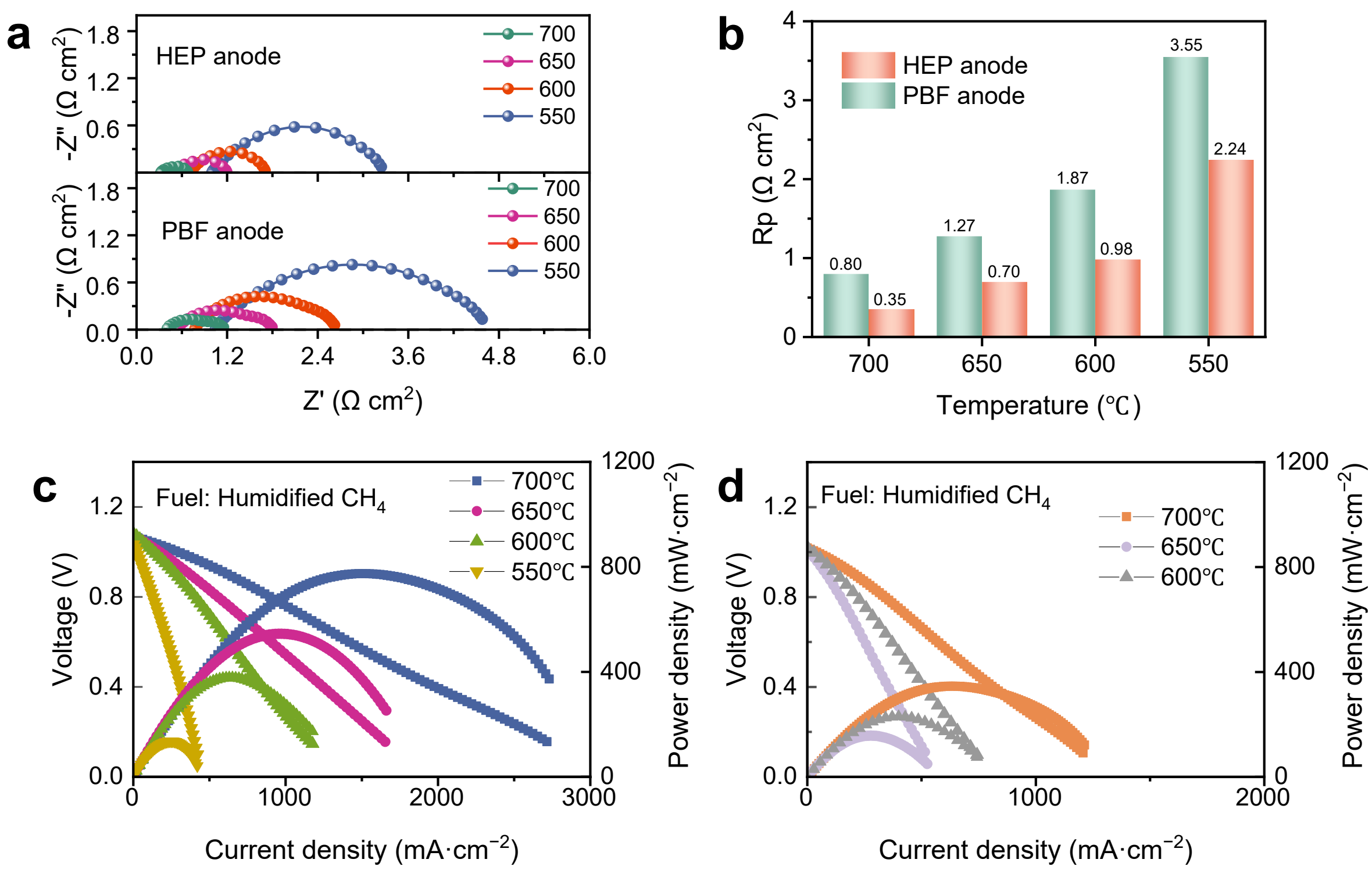

Fig. R19 (also **Fig. 3a, d** and **Fig. S28, 30**) **a** EIS plots of HEP and PBF single cells in 3% H_2O-CH_4. **b** Comparison of EIS using different anodes. I−V−P curves of single cells using (**c**) HEP and (**d**) PBF as the anodes in humidified CH_4 (3 vol% H_2O).

In direct response, we have now supplemented the manuscript with comprehensive full cell EIS data. As shown in the **Fig. R19a**, the R_p of the HEP anode is consistently and markedly lower than that of the PBF anode under all tested conditions. This improvement is quantified in **Fig. R19b**, which indicates that the R_p for HEP is reduced by more than half compared to PBF, a trend that aligns well with the enhancements observed in the direct electrochemical

performance tests (**Fig. R19c, d**).

Together, this evidence supports our conclusion that the superior performance of HEP is a reproducible property of the material itself, rather than an artifact of an exceptionally well-fabricated single cell. The consistent data also reflect the reliability of our optimized fabrication protocol. We hope these comprehensive additions have directly addressed the reviewer's concern by providing the necessary electrochemical validation to support the reproducibility of our findings. Once again, we thank the reviewer for this valuable feedback, which has helped us strengthen the manuscript.

Related discussions (blue-highlighted in Line 398-403) have been added in the revised manuscript as follows:

To substantiate this, we performed EIS and distribution of relaxation times (DRT) analysis on the full cells under operating conditions, with the results presented in Fig. S35. The impedance analysis demonstrates that the R_p of the cell with HEP as the anode is significantly lower than that of the cell using pristine PBF under identical conditions, further verifying the remarkable electrochemical superiority of the HEP anode.

3-1-(IV)

The reported electrode area is extremely small (Supplementary info "with an electrode area of 0.07 cm^2"). If this is not a typographical error, such a limited active area may strongly amplify edge and contact effects. Without EIS, it is not possible to assess how this influences the reported performance and mechanistic interpretation. The authors should confirm the electrode area and clarify its impact.

We sincerely thank the reviewer for highlighting this critical point regarding the electrode area and its potential impact on performance interpretation. We apologize for the typographical error in the Supplementary Information, which has now been corrected.

The actual active electrode area for all electrochemical testing was 0.7 cm^2, not 0.07 cm^2. We regret this oversight and have corrected the value throughout the revised manuscript (Methods section and relevant figure captions). To provide unambiguous evidence, we have included a photograph of a typical fabricated single cell in the **Fig. R20**, where the electrode area is clearly delineated and labeled as 0.7 cm^2.

As extensively detailed in our response to the major concern regarding EIS, we have now supplemented the manuscript with complete electrochemical impedance spectroscopy data (**Fig. R14**). The obtained spectra exhibit well-defined, reproducible features with low series resistance. This confirms that the cell with the 0.7 cm^2 electrode exhibited good interfacial contact, allowing the measured polarization resistance to robustly reflect the intrinsic electrochemical activity of the electrode materials.

In summary, the combination of the corrected area, direct visual evidence, and the newly provided EIS analysis ensures that the reported performance metrics and our subsequent mechanistic interpretations are based on reliable and representative electrochemical data. We are grateful to the reviewer for prompting these important clarifications, which have strengthened the manuscript.

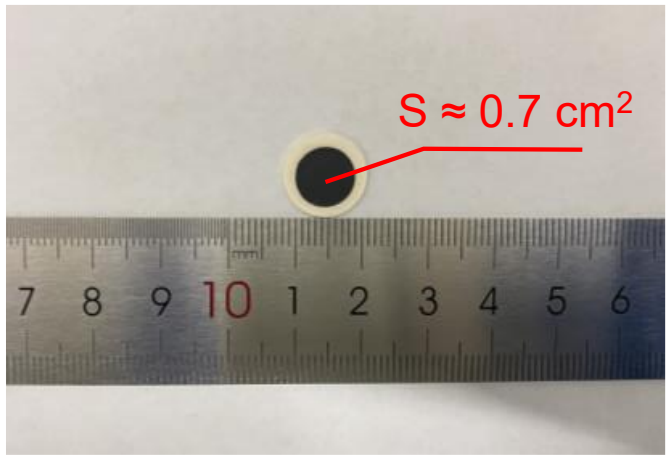

Fig. R20 Photograph of the fabricated single cell.

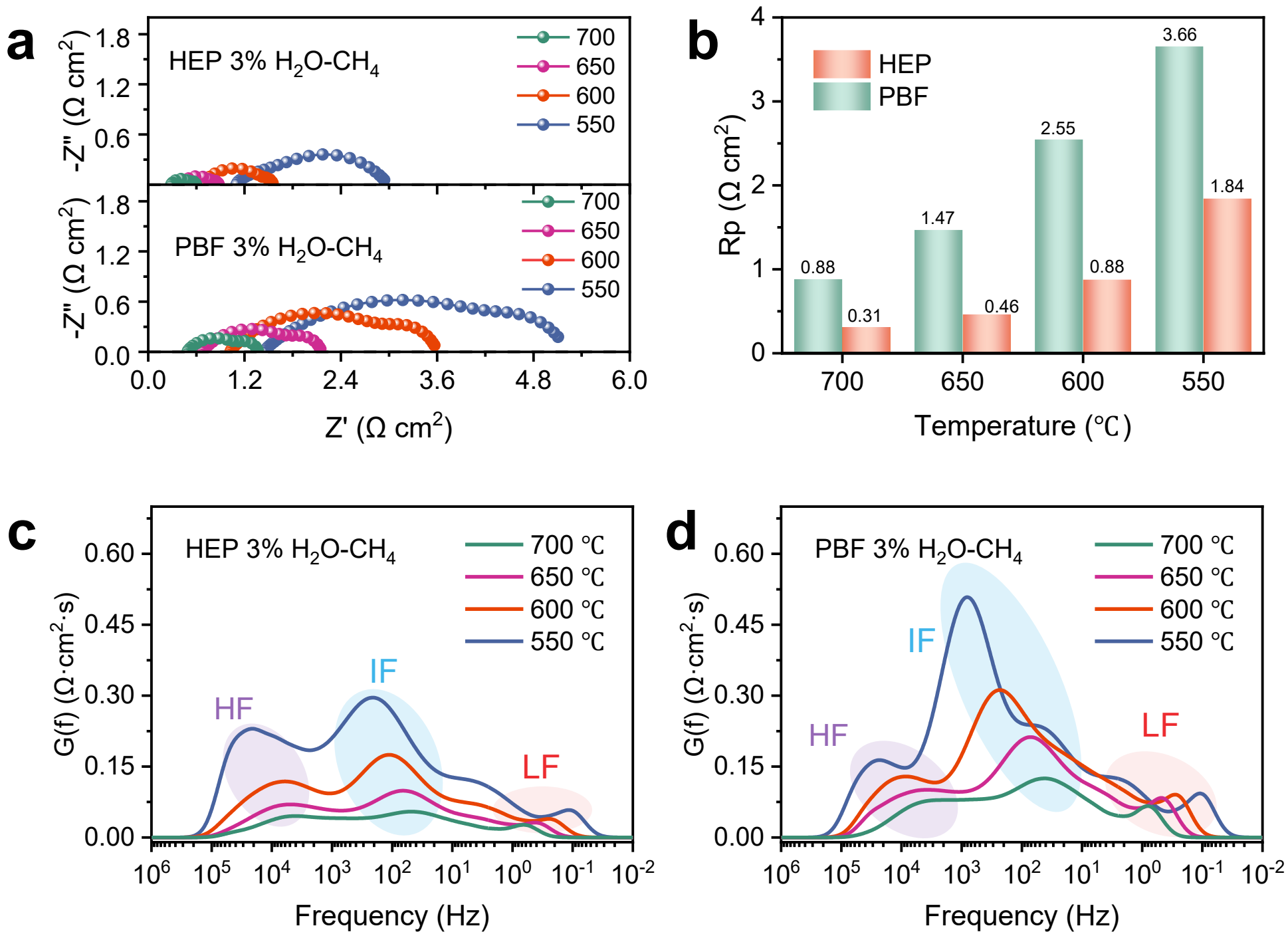

Fig. R14 (also **Fig. S24**) **a** EIS plots of HEP and PBF anodes in 3% H_2O-CH_4. **b** Comparison of EIS using different anodes. DRT fitting results for **c**) HEP and **d**) PBF.

Related discussions have been added in the revised Supplementary information as follows:

HEP and $La_{0.6}Sr_{0.4}Co_{0.2}Fe_{0.8}O_3$ (LSCF) were used as the anode and cathode of the cell, respectively, and were coated on both sides of the GDC by screen printing, with an electrode area of 0.7 cm^2.

Major Comment 3-2

Entropy is not isolated as a governing design parameter: The manuscript does not clearly justify the choice of this specific stoichiometric composition beyond invoking configurational entropy, nor does it discuss why alternative compositions or lower-complexity variants were not considered.

(I) What specific criteria, beyond configurational entropy, guided the selection of the chosen stoichiometric composition?

(II) Were alternative compositions with intermediate/lower configurational entropy considered or tested, and if so, how did their electrochemical behaviour compare?

(III) How do the authors exclude contributions from lattice strain, defect association, or differences in sintering and microstructure as the dominant factors governing the observed behaviour?

Response to 3-2:

We sincerely appreciate the reviewer for this incisive and insightful comment. Your valuable opinion not only points out the essential consideration in material design but also helps us more clearly elaborate on the core research focus and design logic of this work. We fully agree with your viewpoint that configurational entropy is not an isolated governing design parameter for the development of SOFC anode materials.

In the design of high-performance SOFC anode materials, it is undeniable that the comprehensive performance is regulated by a synergy of multiple key parameters including chemical composition, crystal structure, microstructural characteristics, and surface chemical properties. Configurational entropy is only one of the important structural design parameters in this system. In this work, we do not intend to ignore the role of other design parameters, but rather choose to focus on a specific core scientific issue for in-depth and systematic investigation: we take the layered double perovskite oxide $La_{0.2}Pr_{0.2}Sm_{0.2}Gd_{0.2}Y_{0.2}BaFe_2O_{5+\delta}$ (HEP) as the research object, and systematically demonstrate the regulatory law of configurational entropy on the Ln/Ba cation ordering of this material system, and further elucidate the intrinsic mechanism by which the entropy-induced order-disorder transition of the

layered double perovskite optimizes the core electrochemical performance of the SOFC anode (e.g., oxygen vacancy formation and migration, surface hydration capacity, hydrocarbon reforming kinetics, and anti-coking performance).

This focused research design is intended to establish a clear structure-property relationship for entropy-engineered layered perovskite SOFC anodes, and to provide a targeted design strategy for the development of high-performance hydrocarbon-fueled SOFC anode materials—rather than attempting to isolate configurational entropy as the sole design parameter for anode performance optimization. We believe that this in-depth exploration of the correlation between configurational entropy and the structural and electrochemical properties of layered perovskites can also lay a solid foundation for the subsequent synergistic optimization of SOFC anode materials by combining configurational entropy with other design parameters.

Again, we would like to express our sincere gratitude to the reviewer for this instructive comment, which has helped us further clarify the research positioning of this work.

3-2-(I)

What specific criteria, beyond configurational entropy, guided the selection of the chosen stoichiometric composition?

Response to 3-2-(I):

We sincerely appreciate the reviewer for this meticulous question, which helps us further clarify the comprehensive considerations for the selection of the stoichiometric composition of our layered double perovskite oxide $La_{0.2}Pr_{0.2}Sm_{0.2}Gd_{0.2}Y_{0.2}BaFe_2O_{5+\delta}$. We would like to elaborate on the specific criteria and practical considerations beyond configurational entropy as follows:

First, the selection of the rare-earth elements is based on the mature experimental research foundation of layered double perovskite oxides. In previous studies, rare-earth elements including La, Pr, Sm, Gd, and Y have been successfully incorporated into layered double perovskite oxide systems, as exemplified by $LaBaFe_2O_{5+\delta}$, $SmBaFe_2O_{5+\delta}$ and $GdBaFe_2O_{5+\delta}$. These results demonstrate that such rare-earth species can be stably hosted in the crystal lattice of these oxides, without inducing significant structural distortion or phase decomposition [D. Chen et al., *Electrochim. Acta* **78**, 466–474 (2012); Sengodan et al., *Nat. Mater.* **14**, 205–209 (2015); Xue et al., *RSC Adv.* **13**, 30606-30614 (2023); Zhang. *Int. J Hydrogen Energy* **48**, 28119-28130 (2023)]. This provides a reliable experimental basis for our selection of rare-earth elements.

Second, it is important to note that the core research objective of this work is to systematically demonstrate the regulatory law of configurational entropy on the Ln/Ba cation ordering of this layered double perovskite oxide. Configurational entropy itself has no inevitable correlation with the specific types of rare-earth elements selected. We are frank to state that the primary practical considerations for choosing La, Pr, Sm, Gd and Y as the A-site rare-earth elements in this study are that these elements are relatively low in price, non-radioactive, and easily accessible in laboratory research. Such a selection also ensures the repeatability and practical application potential of our experimental research.

In addition, the regulation of configurational entropy in our research system is mainly

achieved by controlling the number of rare-earth element types introduced at the A-site. Our research results have also confirmed that the configurational entropy of the oxide will be further increased with the transition of the material from an A-site Ln/Ba ordered structure to a disordered structure. We adopted the equimolar ratio for the five rare-earth elements simply because this ratio can maximize the configurational entropy of the material, which is highly consistent with our core research goal of exploring the regulatory effect of configurational entropy on the structural and electrochemical properties of the oxide.

All the above considerations are closely combined with the research focus of this work, and the selected stoichiometric composition is also to better serve the in-depth exploration of the correlation between configurational entropy and Ln/Ba ordering in layered double perovskite oxides.

Related discussions have been added in the revised manuscript (blue-highlighted in Line 107) and Supplementary information as follows:

Line 107: (see Supplementary Note 1 for material design rationale).

Supplementary Note 1

Design Rationale of the High-Entropy Perovskite Anode Material

La, Pr, Sm, Gd, Y are selected as equimolar A-site rare-earth elements for constructing $La_{0.2}Pr_{0.2}Sm_{0.2}Gd_{0.2}Y_{0.2}BaFe_2O_{5+\delta}$ (HEP) based on three key considerations:

These elements have been successfully incorporated into layered double perovskite lattices in previous studies, verifying their stable existence without severe lattice distortion or phase decomposition, and providing a reliable experimental foundation for synthesis.[5-8]

As non-radioactive rare-earth elements with low cost and easy accessibility, their selection ensures experimental repeatability and potential for subsequent practical application.

The core goal of this work is to elucidate configurational entropy regulation on Ln/Ba ordering and electrochemical properties. Equimolar doping of five elements maximizes A-site configurational entropy, which is the key to complete disruption of the original ordered layered structure and formation of a disordered high-entropy perovskite, directly serving the study of entropy-driven structural modulation mechanisms.

3-2-(II)

Were alternative compositions with intermediate/lower configurational entropy considered or tested, and if so, how did their electrochemical behaviour compare?

Response to 3-2-(II):

We sincerely appreciate the reviewer for this insightful question, which allows us to further elaborate on our systematic investigations into low/medium-entropy alternative compositions and the core correlation between configurational entropy, A-site cation order degree, and electrochemical performance. This is a key research focus that underpins the central argument of our paper.

In response, we have synthesized a series of A-site binary, ternary and quaternary rare-earth low/medium-entropy perovskite variants, including $La_{0.5}Pr_{0.5}BaFe_2O_{5+\delta}$ (LPBF, S_{config} = 0.69R), $La_{0.33}Pr_{0.33}Sm_{0.33}BaFe_2O_{5+\delta}$, (LPSBF, S_{config} = 1.10R) and $La_{0.25}Pr_{0.25}Sm_{0.25}Gd_{0.25}BaFe_2O_{5+\delta}$, (LPSGBF, S_{config} = 1.39R). Comprehensive thermodynamic, structural, and electrochemical characterizations were conducted on all variants. The detailed results, which repeatedly validate that configurational entropy is the core driving factor regulating the A-site cation order degree of layered double perovskite oxides, are elaborated as follows:

1. Thermodynamic analysis of configurational entropy reveals the entropy threshold for A-site order-disorder transition

We extended the thermodynamic analysis of HEP to these low/medium-entropy variants, with the configurational entropy of the material system quantified by a modified formula:

$$S_{config} = -2R[(1-x)\ln(1-x) + x\ln x] + R\ln N$$

(where x is the Ba incorporation ratio in the Pr layer, N is the number of A-site rare-earth elements, and R is the universal gas constant). As presented in **Fig. R21**, the maximum configurational entropy of the material system decreases progressively with the reduction in the number of A-site rare-earth elements (N), while the corresponding system energy rises significantly. For the binary LPBF and ternary LPSBF variants, the configurational entropy generated by fewer A-site rare-earth elements is insufficient to provide the thermodynamic

driving force required to disrupt the inherently stable A-site cation ordered structure of layered double perovskites. This constitutes the fundamental thermodynamic origin of their preserved layered structural characteristics. Only when the number of A-site rare-earth elements reaches $N = 4$ and $N = 5$, and the configurational entropy crosses the critical threshold, can a complete A-site cation order-to-disorder transition be thermodynamically achieved in the layered double perovskite system. This result directly demonstrates that configurational entropy is the key thermodynamic factor determining the A-site cation order degree of the material.

2. Structural characterizations confirm the entropy-dependent evolution of A-site cation ordering

XRD characterizations (**Fig. R22a-c**) verify that all these low/medium-entropy variants form a pure single-phase perovskite structure, eliminating phase interference and ensuring valid, phase-consistent structural and electrochemical comparisons with HEP. HAADF-STEM (**Fig. R22d-f**) and EDS elemental mapping (**Fig. R23-25**) analyses further reveal a clear entropy-dependent structural evolution law of A-site cation ordering in the layered double perovskite system, where the order degree of A-site cations is directly regulated by the magnitude of configurational entropy. Specifically, LPBF and LPSBF, with lower configurational entropy, retain the characteristic alternating bright-dark lattice fringes of the parent layered PBF oxide, and Pr and Ba exhibit a distinct ordered layered distribution in the lattice. These features represent the typical structural hallmarks of the original A-site cation ordered structure, confirming that low configurational entropy is unable to disrupt A-site ordering. For LPSGBF (the quaternary variant with the highest configurational entropy among the low/medium-entropy samples), the increase in configurational entropy drives the gradual fading of these ordered lattice features, and Pr/Ba cations begin to display a homogeneous mixed distribution in the lattice. This observation clearly indicates the incipient disruption of A-site cation ordering with the elevation of configurational entropy, further validating that configurational entropy is the core structural driving factor regulating the A-site order degree of layered double perovskite oxides.

3. Electrochemical tests demonstrate that configurational entropy modulates

electrochemical performance by regulating A-site order degree

We performed systematic electrochemical tests on all these low/medium-entropy variants under identical SOFC operating conditions as HEP, and the results reveal a direct positive correlation between configurational entropy and electrochemical performance. This correlation is essentially mediated by the configurational entropy's regulation of A-site cation order degree. As shown in **Fig. R26**, LPBF and LPSBF exhibit markedly higher polarization resistance and a significantly lower peak power density than HEP. This inferior electrochemical performance originates from their retained A-site cation ordered structure (caused by insufficient configurational entropy), which inherently limits oxygen vacancy formation in the bulk phase, restricts long-range oxygen ion diffusion, and impairs the surface hydration capacity of the anode—all of which are core physicochemical properties governing the catalytic and electrochemical activity of SOFC anodes. Even LPSGBF, which exhibits partial A-site disordering driven by a relatively higher configurational entropy compared with LPBF and LPSBF, still shows inferior electrochemical performance to HEP (with higher polarization resistance and lower peak power density). This is because the configurational entropy of the quaternary LPSGBF has not reached the optimal level of the pentanary HEP, leading to an incomplete A-site order-to-disorder transition; the residual A-site ordering still imposes inherent limitations on the key electrochemical properties of the anode. These electrochemical results fully confirm that the magnitude of configurational entropy governs the A-site cation order degree of layered double perovskite oxides, and this order degree in turn determines the ultimate electrochemical performance of the SOFC anode. Such a structure-property relationship centered on configurational entropy constitutes the core conclusion of this work.

These comparative studies on low/medium-entropy variants not only validate that configurational entropy serves as the dominant governing parameter for regulating A-site cation ordering in PBF-based perovskites, but also confirm that only the high-entropy composition (HEP, N=5, equimolar) enables a complete order-to-disorder transition. Such a transition is essential for optimizing oxygen vacancy chemistry, oxygen ion transport, and hydration capacity, which ultimately endows HEP with outstanding electrochemical activity and anti-

coking stability.

The electrochemical performance of the lower-complexity variants is inherently limited by their incomplete A-site disordering, directly justifying our design of the five-element equimolar high-entropy composition for the SOFC anode.

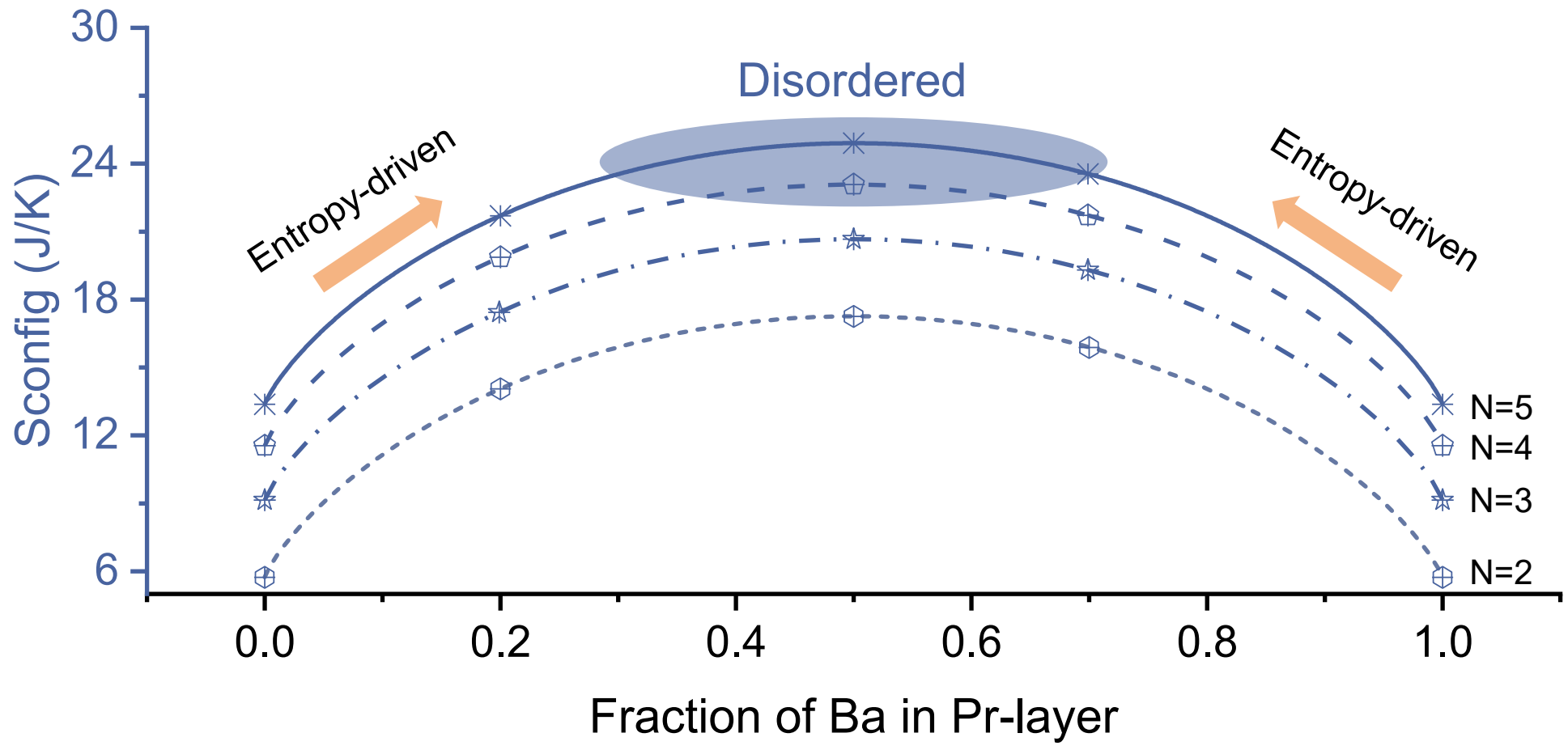

Fig. R21 Entropy of different perovskites and system energy values as a function of fraction of Ba in Pr-layer.

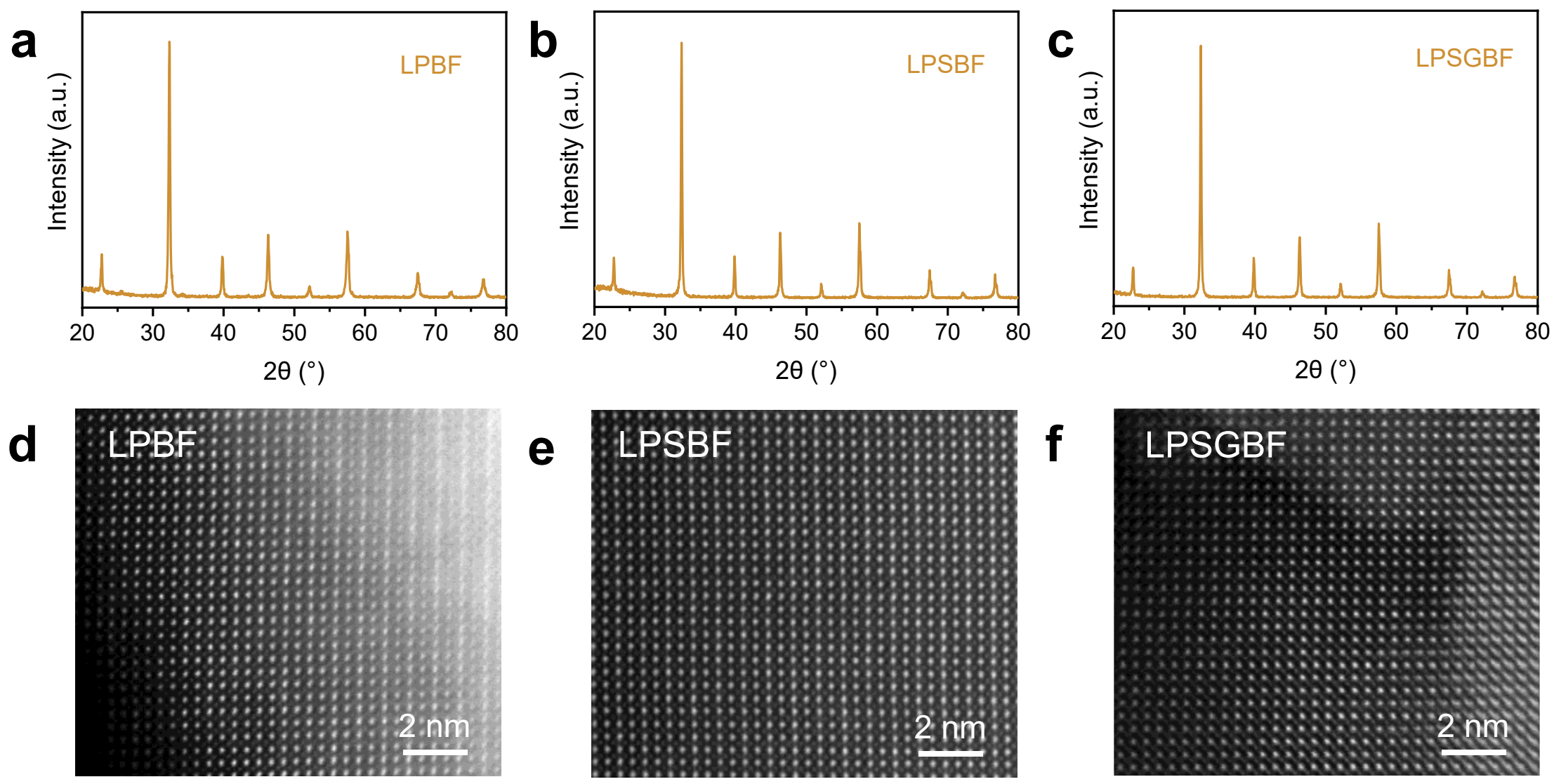

Fig. R22 (also **Fig. S12)** XRD patterns at RT of (**a**) LPBF, (**b**) LPSBF and (**c**) LPSGBF. HAADF-STEM images of (**d**) LPBF, (**e**) LPSBF and (**f**) LPSGBF.

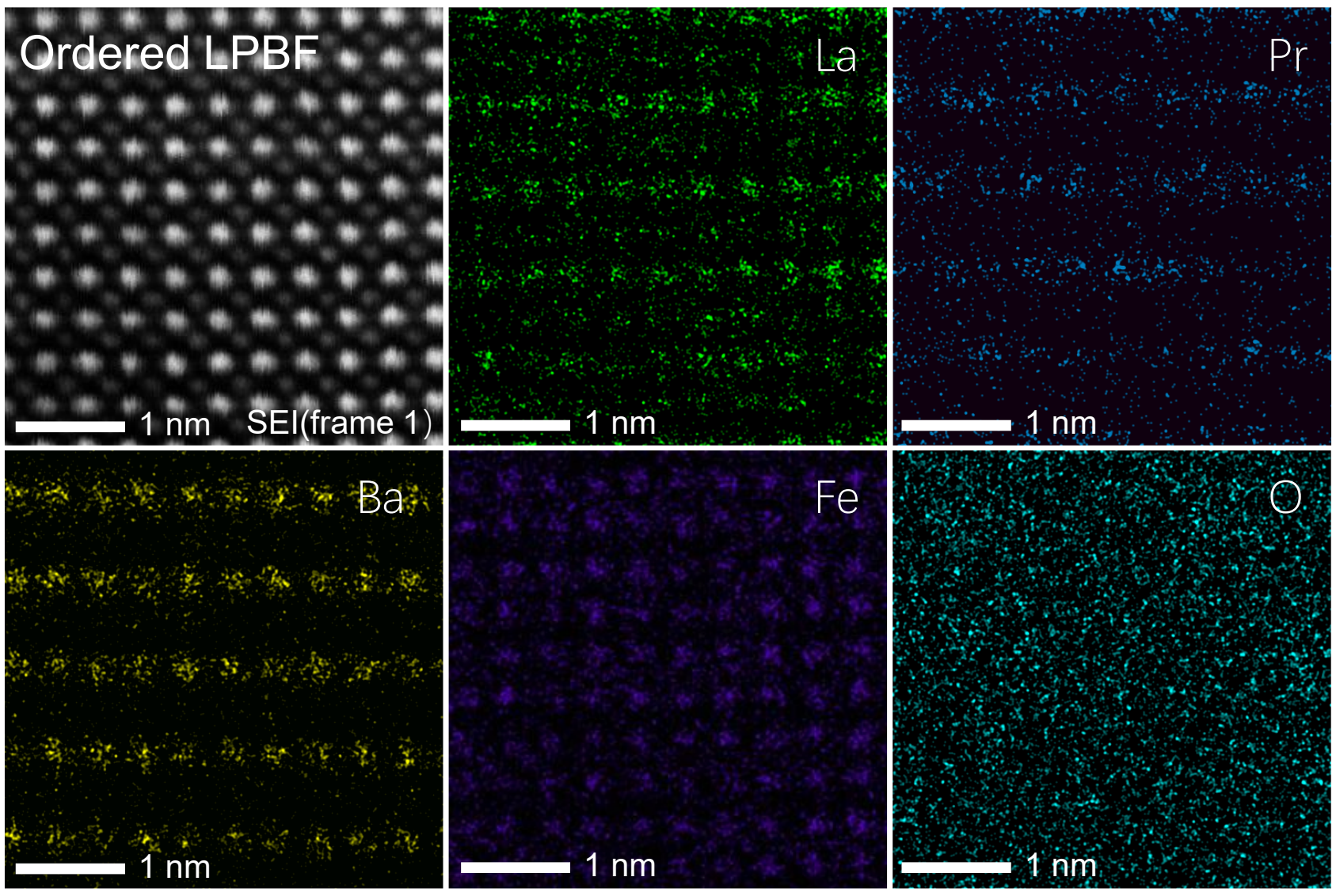

Fig. R23 (also **Fig. S13)** HAADF-STEM elemental maps of LPBF.

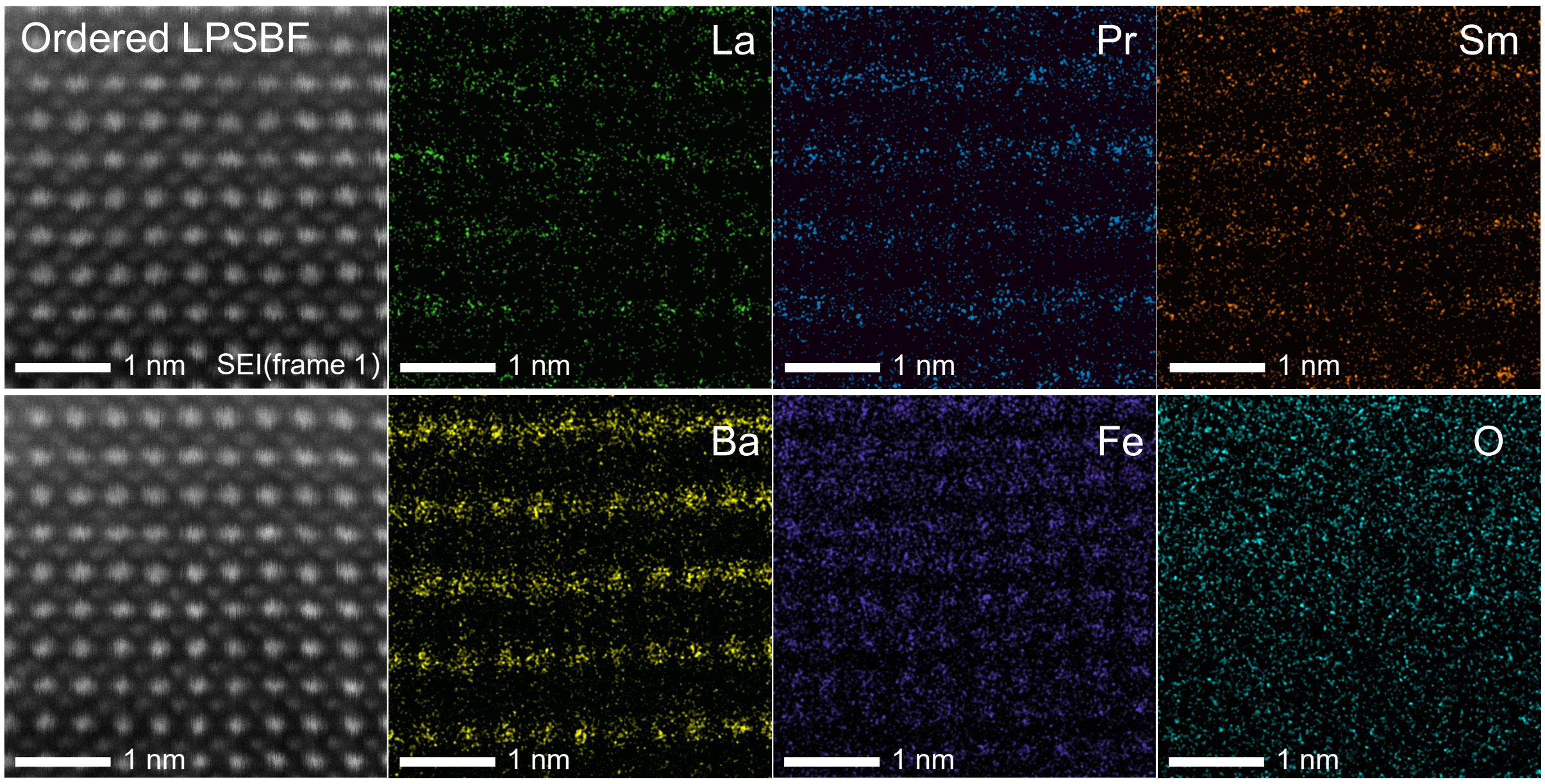

Fig. R24 (also **Fig. S14)** HAADF-STEM elemental maps of LPSBF.

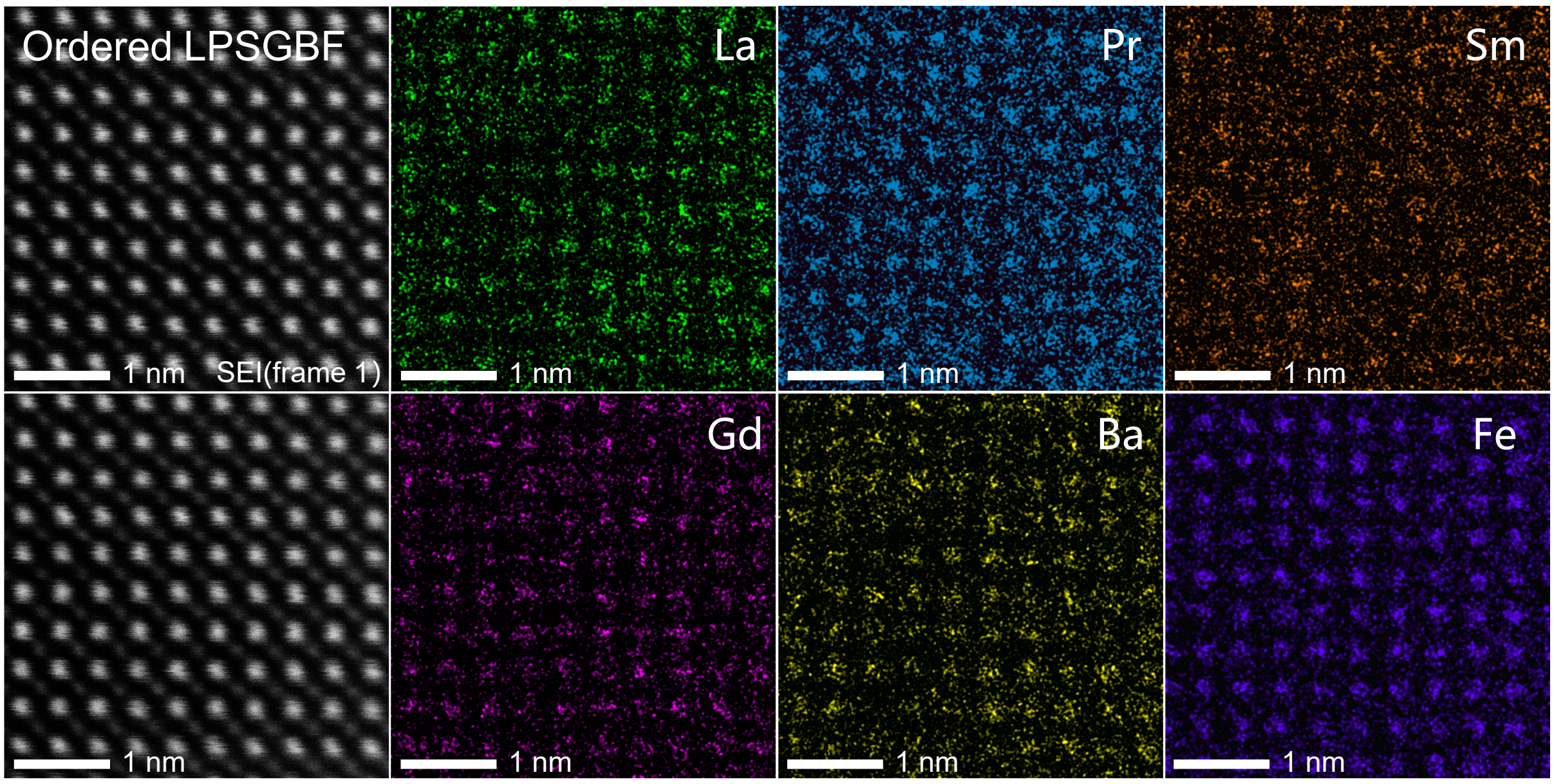

Fig. R25 (also **Fig. S15**) HAADF-STEM elemental maps of LPSGBF.

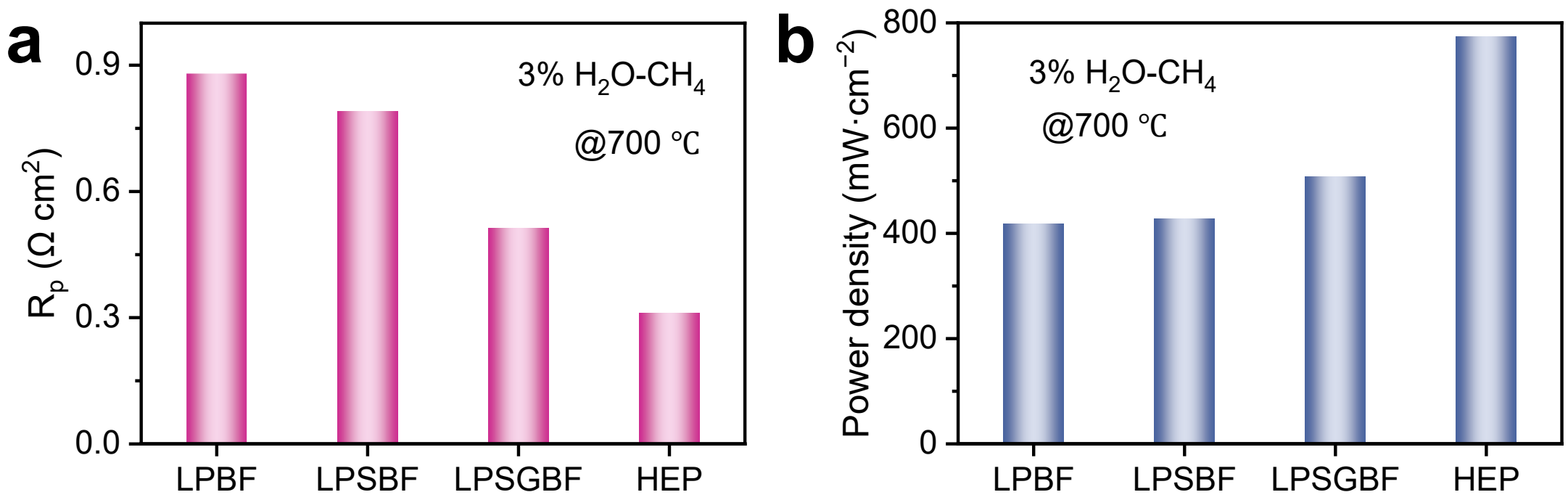

Fig. R26 a EIS plots of anodes under 3% H_2O-CH_4 at 700 °C. **b** I−V−P curves of single cells using different anodes under 3% H_2O-CH_4 at 700 °C.

Related discussions have been added in the revised manuscript as follows:

Line 193-204: To further understand the relationship between configurational entropy and order-to-disorder transitions, the number of A-site elements was reduced to lower the configurational entropy of the perovskite. A series of A-site binary, ternary, and quaternary rare-earth low/medium-entropy perovskite derivatives were synthesized and investigated, including $La_{0.5}Pr_{0.5}BaFe_2O_{5+\delta}$ (LPBF, S_{config} = 0.69R), $La_{0.33}Pr_{0.33}Sm_{0.33}BaFe_2O_{5+\delta}$ (LPSBF, S_{config} =

1.10R), $La_{0.25}Pr_{0.25}Sm_{0.25}Gd_{0.25}BaFe_2O_{5+\delta}$ (LPSGBF, S_{config} = 1.39R). XRD characterizations (Fig. S6a–c) confirm that all these samples adopt a pure single-phase perovskite structure. HAADF-STEM (Fig. S6d–f) and EDS elemental mappings (Fig. S7–9) reveal a clear entropy-dependent structural evolution. Binary LPBF and ternary LPSBF with lower entropy retain the typical layered ordering of the parent PBF, whereas the high-entropy quaternary LPSGBF exhibits a disordered structure. These results confirm that configurational entropy acts as the key driving force regulating the A-site ordering in layered double perovskites.

Line 336-340: To further understand the effect of the order-to-disorder transition on electrochemical performance, we also compared the EIS spectra of perovskites with different entropy values (LPBF, LPSBF, and LPSGBF). As shown in Fig. S26, the results reveal that disordered HEP exhibit the lowest Rp values. This indicates that anodes with a disordered structure possess superior electrochemical properties.

Line 353-356: Compared to the anodes with different S_{config} (LPBF, LPSBF, and LPSGBF) and the most recent advancements in the field (Fig. 3c, S30, and Table S5), our disordered HEP anode exhibits significantly superior power density performance when benchmarked against both single-phase and ordered double perovskite oxide systems.

3-2-(III)

How do the authors exclude contributions from lattice strain, defect association, or differences in sintering and microstructure as the dominant factors governing the observed behaviour?

Response to 3-2-(III):

We sincerely appreciate the reviewer for this critical and insightful question, which drives us to rigorously exclude the potential dominant contributions of lattice strain, defect association, sintering conditions and microstructure to the observed performance differences between HEP and PBF via targeted theoretical calculations and supplementary experimental characterizations. The detailed evidence and analysis for each factor are presented as follows:

1. Exclusion of lattice strain as a dominant influencing factor

Lattice strain is a common structural feature in high-entropy oxide systems. To rule out its role as a dominant factor for the enhanced oxygen vacancy-related properties and electrochemical performance of HEP, we constructed a theoretical model of Ordered-HEP, whose A-site cations adopt the same ordered layered arrangement as PBF, for comparative DFT calculations of Fe–O bond energy with disordered HEP and PBF (**Fig. R27**). The calculated Fe–O bond energy values are 6.96 eV for computationally designed ordered-HEP, 6.34 eV for experimentally disordered HEP, and 7.19 eV for PBF. It is noteworthy that lattice strain is also present in the ordered-HEP theoretical model, yet its Fe–O bond energy is significantly higher than that of disordered HEP. A higher Fe–O bond energy indicates a stronger metal-oxygen interaction in the lattice and a higher energy barrier for oxygen vacancy formation. This theoretical calculation result directly demonstrates that the optimized oxygen vacancy chemistry of HEP is not derived from lattice strain, but from the entropy-driven A-site cation order-to-disorder transition of the layered double perovskite structure.

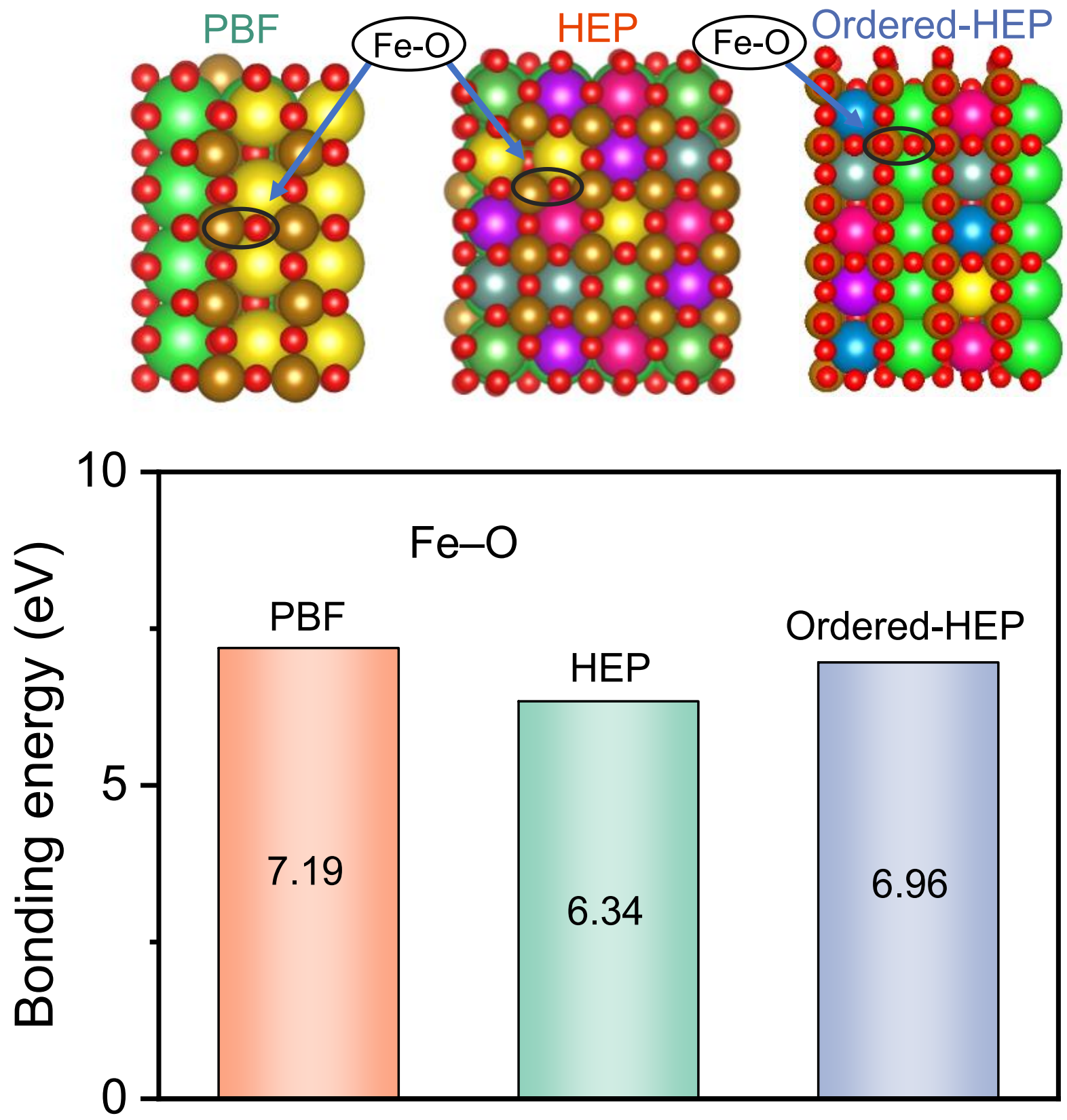

Fig. R27 (also **Fig. S18**) Comparison of the Fe–O bond energies of different anode.

2. Exclusion of defect association as a dominant influencing factor

We clearly confirm that oxygen vacancy defects are a key factor regulating the electrochemical performance of SOFC anodes, and this is also the case in our research. The superior electrochemical performance of HEP is indeed closely related to the characteristics of oxygen vacancy defects in its lattice, yet this positive effect is derived from the increased concentration of oxygen vacancies induced by the entropy-driven A-site cation disordered structure rather than defect association. We have obtained multiple lines of experimental evidence to verify the absence of dominant defect association in HEP and clarify the intrinsic link between cation disorder, oxygen vacancy concentration and performance. First, all selected A-site rare-earth cations stably maintain a +3 valence state in the lattice, and Ba^{2+} remains in a fixed +2 valence state during both material synthesis and electrochemical testing. This stable valence distribution avoids charge imbalance in the perovskite lattice, which is the main cause of defect clustering and association. Second, electron paramagnetic resonance measurements

show that HEP only exhibits a single, sharp characteristic signal at g = 2.003, which is assigned to isolated oxygen vacancies in the lattice, and no additional miscellaneous peaks corresponding to defect associations or complex defect clusters are detected. Third, the H_2-TPR profile of HEP presents a single continuous reduction peak instead of discrete stepwise peaks, which further verifies that oxygen vacancies in the HEP lattice are uniformly distributed in an isolated form, and there is no inhomogeneous reducibility caused by defect association. Collectively, these experimental results confirm that there is no significant defect association in the HEP lattice. The positive contribution of oxygen vacancy defects to the electrochemical performance of HEP is essentially the result of the increased oxygen vacancy concentration induced by the A-site cation disordered structure. This also further confirms that the entropy-driven cation disorder is the core factor regulating the oxygen vacancy characteristics of HEP and thus enhancing its electrochemical performance, while defect association does not act as a dominant factor governing the observed superior performance of HEP.

3. Excluding the contribution of sintering conditions

To eliminate the influence of sintering and preparation conditions on the structural and electrochemical properties of HEP and PBF, we adopted an identical synthetic and sintering protocol for both materials throughout the entire preparation process. This consistent protocol includes the same nitrate-based precursor ratio, identical ball-milling conditions involving milling time, rotation speed and milling medium, the same calcination and sintering parameters covering temperature, holding time, heating and cooling rate and air atmosphere, as well as consistent post-treatment processes for the two materials. The use of completely uniform preparation and sintering conditions ensures that no artificial performance differences are introduced by external preparation parameters. The observed structural and electrochemical disparities between HEP and PBF are thus solely derived from their intrinsic compositional and structural differences that are the ordered and disordered A-site cation arrangements.

4. Excluding the contribution of microstructure

We have supplemented a series of microstructure characterizations (**Fig. R28**) for as-synthesized HEP and PBF powders to rule out microstructure as a dominant factor, including

scanning electron microscopy (SEM), particle size distribution statistics, and Brunauer-Emmett-Teller (BET) specific surface area measurements. The results show that HEP and PBF exhibit highly similar powder morphologies with no obvious differences in particle shape; their average particle sizes are nearly identical (PBF: 0.189 μm, HEP: 0.196 μm); and their BET specific surface areas are also at the same level (PBF: 2.27 m^2/g, HEP: 2.23 m^2/g). The high similarity in these key microstructural parameters excludes the dominant contributions of particle size, specific surface area and powder morphology to the observed electrochemical performance differences between HEP and PBF.

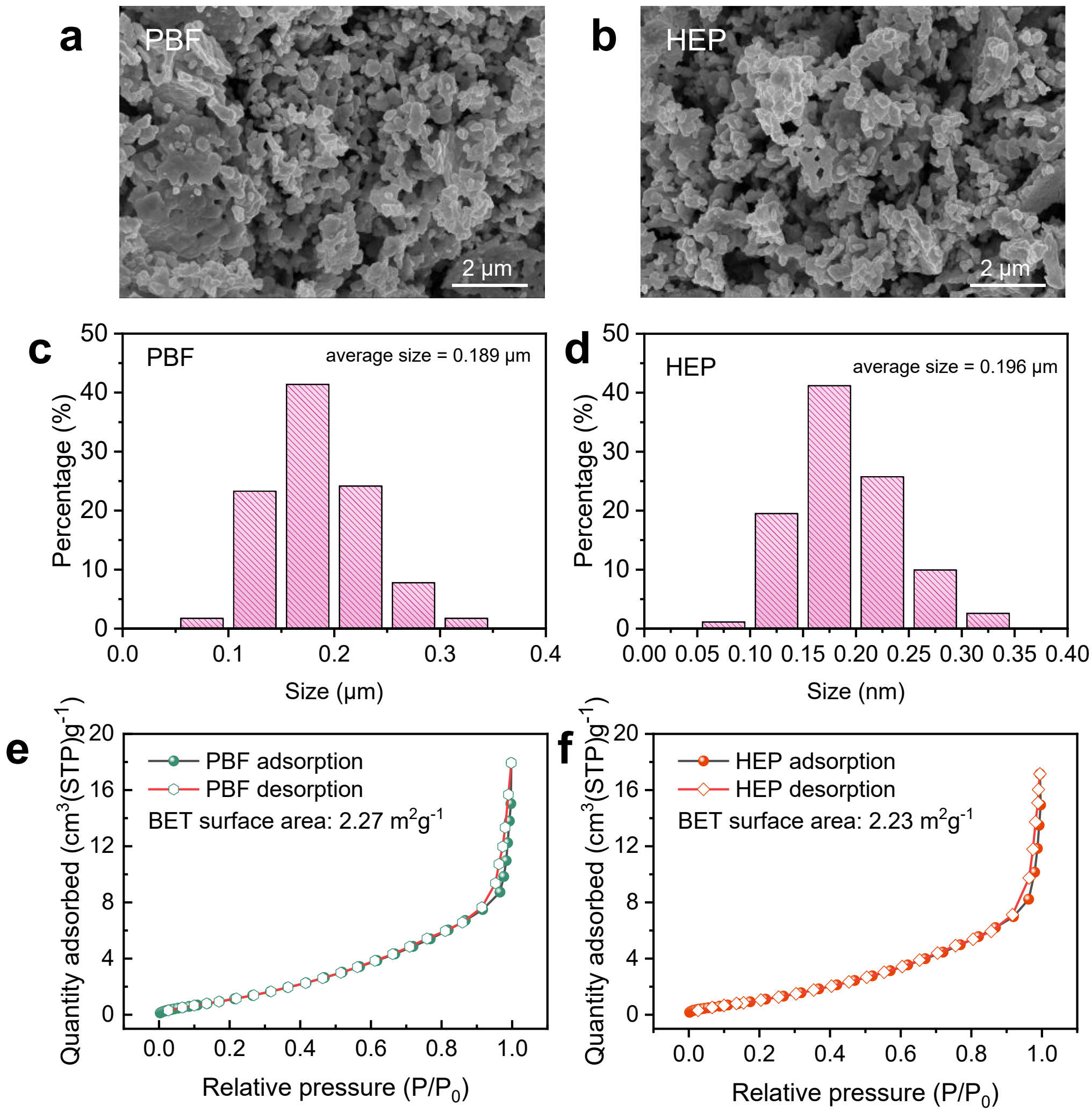

Fig. R28 (also **Fig. S25**) SEM images of the as-prepared (**a**) PBF and (**b**) HEP. The corresponding size distribution of (**c**) PBF and (**d**) HEP. The nitrogen adsorption-desorption isotherm plots and Brunauer-Emmett-Teller (BET) surface area of (**e**) PBF and (**f**) HEP.

In summary, the above targeted theoretical calculations and comprehensive experimental characterizations have rigorously and systematically excluded lattice strain, defect association, sintering conditions and microstructure as the dominant factors governing the observed behaviour. We confirm that the enhanced electrochemical activity, anti-coking performance and long-term stability of HEP are essentially derived from the entropy-driven A-site cation order-to-disorder transition, which optimizes the oxygen vacancy chemistry, oxygen ion transport and surface hydration capacity of the perovskite anode.

Related discussions have been added in the revised manuscript as follows:

Line 245-251: To further verify the influence of cation ordering on the Fe–O bond energy, we constructed an ordered high-entropy perovskite model (computationally designed ordered HEP) with the same unit-cell parameters as the experimentally disordered HEP, as illustrated in Fig. S18. The calculated Fe–O bond energy for computationally designed ordered-HEP is 6.96 eV, which is significantly higher than that of experimentally disordered HEP (6.34 eV). This directly demonstrates that the order-to-disorder transition can promote the formation of oxygen vacancies.

Line 332-335: Notably, for the two anodes prepared under similar conditions with comparable specific surface areas (Fig. S25), the observed differences in electrochemical properties can be attributed to the intrinsic nature of the samples themselves.

Major Comment 3-3

Lack of experimental validation of the proposed mechanism: The manuscript links ex situ evidence related to hydration, reforming activity and carbon removal to the electrochemical behaviour observed in current-voltage (iV) curves; however, such correlations are not sufficient to substantiate the proposed mechanism. In the absence of operando or operating-condition-resolved validation, the mechanistic interpretation remains largely correlative. Once again, the lack of EIS data and deconvoluted resistances prevents a clear assessment of whether the observed differences arise from intrinsic fuel-electrode behaviour or from other cell-related contributions.

Response to 3-3:

We sincerely appreciate the reviewer for this rigorous and insightful comment, which accurately points out the deficiencies in our mechanistic experimental validation. We attach great importance to this suggestion and have supplementarily conducted in situ electrochemical impedance spectroscopy (EIS) measurements of single cells under dry and humid methane atmospheres, as well as distribution of relaxation time (DRT) deconvolution analysis for the EIS data. All supplementary results have been integrated into the revised manuscript and Supplementary Information, aiming to consolidate the proposed mechanism and clarify the source of the observed performance differences.

As shown in **Fig. R29a, b**, the polarization resistance of the HEP-based single cell decreases by 43.9% upon the introduction of water vapor into methane at 700 °C. In contrast, the polarization resistance of the PBF-based single cell shows only a slight reduction of 24.7% with water vapor addition. These in situ electrochemical results directly verify the stronger hydration capacity of HEP and the significant acceleration of its anode reaction kinetics after water vapor introduction, while also intuitively reflecting the intrinsic performance difference between the two anodes

To further explore the anode reaction processes, we performed distribution of relaxation times (DRT) analysis on the electrochemical impedance spectroscopy (EIS) spectra obtained at 700 °C, with the corresponding results presented in **Fig. R29c, d**. The DRT profiles can be

divided into high frequency (HF), medium frequency (MF), and low frequency (LF) relaxation regions. After the introduction of water vapor, the MF peak of HEP decreases significantly, indicating a remarkable acceleration of its surface exchange kinetics, whereas the medium-frequency peak of PBF only shows a slight reduction, suggesting a limited improvement in its surface exchange processes. The LF region is associated with gas diffusion processes, and HEP exhibits a smaller LF peak than PBF, which can be attributed to the enhanced methane conversion efficiency of HEP that generates more H_2 and CO to facilitate gas diffusion. The HF region is related to charge transfer processes, and both anodes display low intensities of high-frequency peaks, demonstrating that charge transfer is not the rate-limiting step for the CH_4-fueled SOFC investigated in this study.

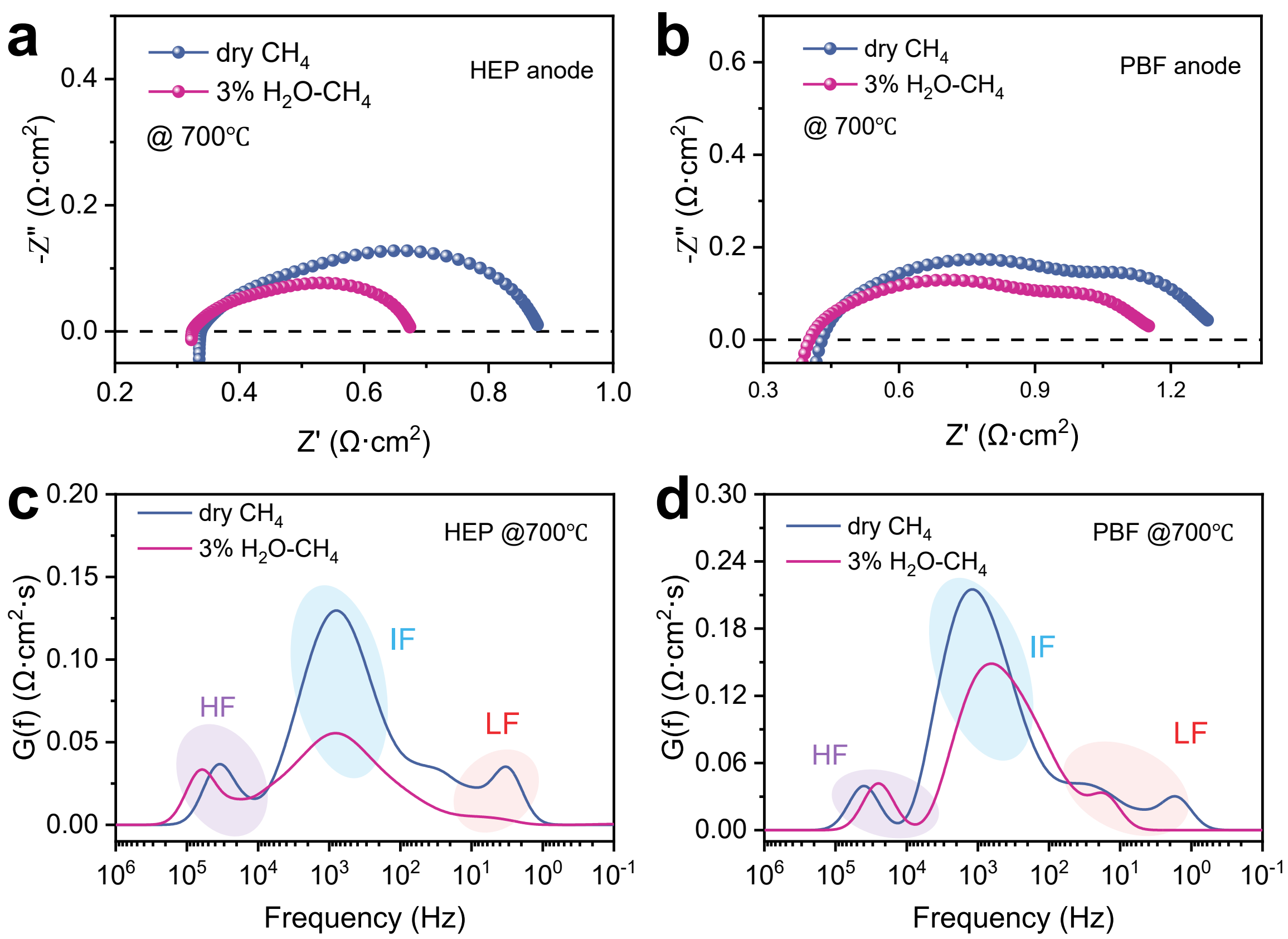

Fig. R29 Electrochemical impedance of (**a**) HEP-based and (**b**) PBF-based single cells in dry or wet CH_4. DRT spectra of (**c**) HEP-based and (**d**) PBF-based cells at 700 °C in 3% H_2O-CH_4.

These supplementary in situ EIS data and DRT analysis establish a direct correlation between our ex situ characterization results and the in situ electrochemical performance of

single cells, and clearly confirm that the observed performance differences between HEP and PBF originate from their intrinsic fuel-electrode behaviour rather than other cell-related contributions. This effectively consolidates the proposed structure-performance mechanism of the material. We sincerely thank the reviewer again for this valuable suggestion, which has significantly improved the scientific rigor of our research. All the supplementary data and detailed analysis have been presented in the revised manuscript, and we kindly request your further review.

Related discussions have been added in the revised manuscript as follows:

Line 398-409: To substantiate this, we performed EIS and distribution of relaxation times (DRT) analysis on the full cells under operating conditions, with the results presented in Fig. S35. The impedance analysis demonstrates that the R_p of the cell with HEP as the anode is significantly lower than that of the cell using pristine PBF under identical conditions, further verifying the remarkable electrochemical superiority of the HEP anode. The intrinsic origin of the reduced R_p for HEP was further elucidated by DRT analysis of the EIS data. As shown in Fig. S35b, each DRT profile displays three well-resolved peaks associated with gas diffusion, ion surface exchange, and interfacial charge transfer in the low-frequency (LF), intermediate-frequency (IF), and high-frequency (HF) regions, respectively. The most prominent difference in the DRT peak areas between HEP and PBF appears in the intermediate-frequency range, verifying that ion surface exchange processes are greatly enhanced in the HEP anode, consistent with its accelerated oxygen ion migration.

Major Comment 3-4

Overinterpretation of oxygen vacancy concentration from ex situ spectroscopy: The manuscript places significant weight on oxygen vacancy concentration to support the proposed mechanistic framework. While the authors employ several characterisation techniques to assess defect chemistry, a central part of the argument relies on ex situ surface-sensitive measurements, particularly O 1s XPS deconvolution. It is well established that XPS cannot directly probe the absence of oxygen species, nor can it reliably discriminate between intrinsic lattice oxygen and surface adsorbates or hydroxylated (for example) species under ambient conditions. This has been extensively discussed in the literature and should be more carefully addressed here.

Idriss et al. Surface Science 712 (2021) 121894

Terry et al. Chemistry of Materials 2023, 35, 5468−5474

Response to 3-4:

We sincerely appreciate the reviewer for this professional and rigorous comment, as well as the valuable reference literatures provided. We have carefully and thoroughly studied the two papers by Idriss et al. and Terry et al., and have a profound understanding of the inherent technical limitations of XPS in oxygen vacancy characterization. It is indeed true that XPS cannot directly detect the absence of oxygen species, nor can it reliably distinguish between intrinsic lattice oxygen and surface adsorbates or hydroxylated species under ambient conditions. To ensure the scientific rigor of our research conclusions and avoid inappropriate interpretation of oxygen vacancy concentration, we have removed all the oxygen vacancy-related characterizations and analyses based on O 1s XPS deconvolution from the revised manuscript.

To more reliably and rigorously verify the oxygen vacancy concentration and the related defect chemistry characteristics of the materials, we have adopted H_2-TPR as an alternative characterization method and completed the relevant tests (**Fig. R30**). H_2-TPR tests were performed on HEP and PBF under identical conditions with a 5 vol% H_2/Ar atmosphere, a heating rate of 10 °C min^{-1} and a temperature range of 30–900 °C. The reduction peaks in the test correspond to the stepwise $Fe^{4+}/Fe^{3+} \rightarrow Fe^{2+}$ reduction process, accompanied by the release

of lattice oxygen, which is an event directly linked to oxygen vacancy concentration and metal-oxygen bond strength. Key test results show the main reduction peak of HEP is at 379.4 °C, a significant 32.8 °C low-temperature shift relative to PBF at 412.2 °C. This obvious low-temperature shift directly demonstrates the weakened Fe–O bond in HEP arising from entropy-driven A-site cation disordering, which is conducive to the formation of a higher concentration of oxygen vacancies in the material lattice.

We would like to express our sincere gratitude to the reviewer again for this instructive comment and the important reference literatures. Your suggestion has helped us identify and correct the deficiencies in our characterization methods, and significantly improved the scientificity and rigor of the defect chemistry characterization in this study. All the original H_2-TPR test data and detailed analysis have been fully supplemented and elaborated in the revised manuscript and Supplementary Information, and we kindly request your further review and guidance.

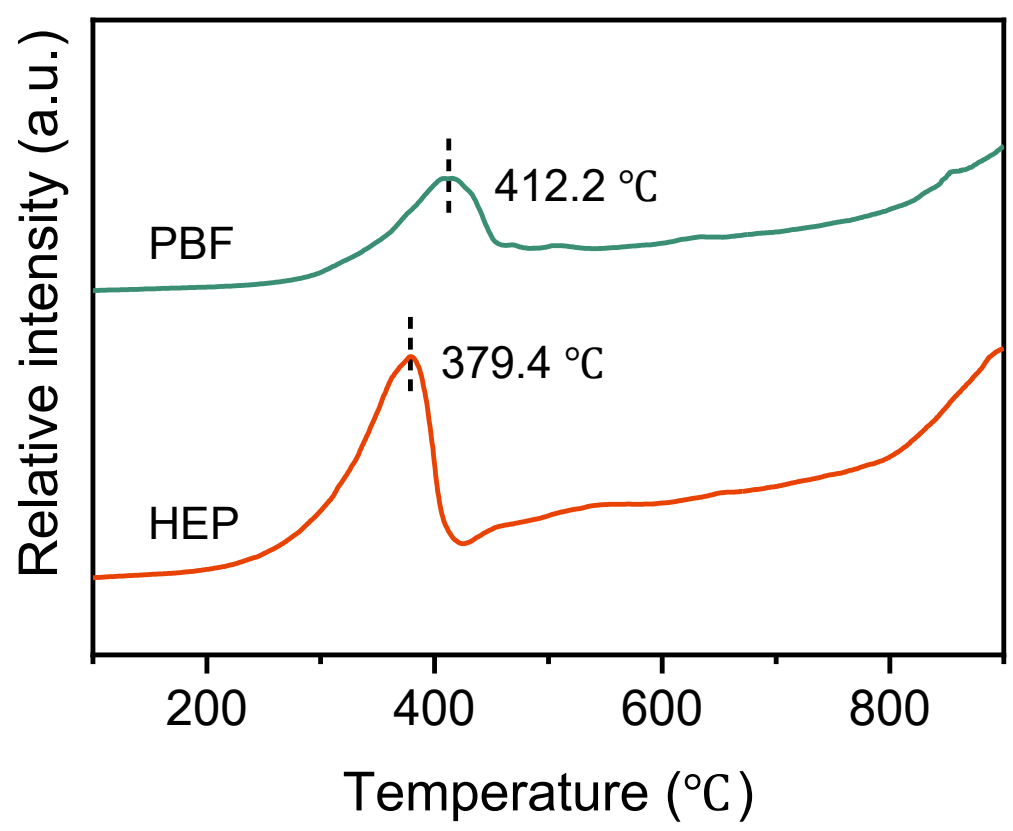

Fig. R30 (also **Fig. 2a**) H_2-TPR spectra of PBF and HEP.

Related discussions (blue-highlighted in Line 228-235) have been added in the revised manuscript as follows:

Line 228-235: To elucidate the influence of ionic disorder on oxygen vacancy concentration in HEP, H_2 temperature-programmed reduction (H_2-TPR) analysis was performed. As presented in Fig. 2a, the H_2-TPR spectra of HEP exhibit a high-temperature H_2

consumption peak, which corresponds to the stepwise reduction of Fe^{4+}/Fe^{3+} to Fe^{2+}.[40] Notably, compared with pristine PBF, the H_2 consumption peak of HEP shifts significantly toward lower temperatures, indicating that the lattice oxygen in HEP is more easily consumed. This observation further confirms that the disordered structure weakens the Fe–O bonds, thereby facilitating the generation of oxygen vacancies in the HEP lattice.

Comments 3-5

3-5-(I) Higher-resolution micrographs should be provided for Fig. 4b and 4c, as the current images lack scale bars, making quantitative or comparative assessment difficult. In addition, the cross-sectional image in Fig. S14 does not show the complete cell configuration, limiting the evaluation of layer thicknesses and interfaces across the full device.

3-5-(II) Synchrotron measurement details: The wavelength used for the synchrotron XRD measurements (Fig. 1a) should be explicitly stated to ensure reproducibility and clarity

3-5-(III) Structural analysis: The authors perform Rietveld refinement using laboratory XRD data despite having access to synchrotron XRD measurements. A justification for this choice should be provided, or alternatively, Rietveld refinement of the synchrotron data should be included to take full advantage of the higher resolution.

Response to 3-5:

We thank the reviewer for these important concerns.

3-5-(I)

Higher-resolution micrographs should be provided for Fig. 4b and 4c, as the current images lack scale bars, making quantitative or comparative assessment difficult. In addition, the cross-sectional image in Fig. S14 does not show the complete cell configuration, limiting the evaluation of layer thicknesses and interfaces across the full device.

Response to 3-5-(I):

We sincerely appreciate the reviewer for this meticulous and constructive comment, which helps improve the clarity and completeness of our microstructural characterization data. We have fully addressed the concerns regarding the micrographs as follows:

1. Updated high-resolution micrographs for Fig. 4b and 4c with scale bars

We have replaced the original micrographs in **Fig. 4b** (HEP anode) and **4c** (PBF anode) with higher-resolution cross-sectional SEM images (as shown in **Fig. R31**) to enhance the visibility of surface morphology, pore structure, and carbon deposition details. Critically, we have added clear scale bars (500 nm) to both images, enabling quantitative comparison of surface features (e.g., carbon fiber thickness, pore size) between HEP and PBF after the 1000-

hour durability test.

The updated images clearly demonstrate that the HEP anode maintains a smooth and intact surface with no observable carbon deposition. By contrast, the PBF anode shows extensive fibrous carbon growth covering the surface and blocking the pores. These observations are consistent with our previous conclusions and are now further supported by quantifiable microstructural evidence. The revised figures have been incorporated into the main manuscript (**Fig. 4b, c**).

2. Supplementary complete full-cell cross-sectional image for Fig. S14

We have updated **Fig. S14** to show the entire SOFC configuration: anode|GDC|SSZ|GDC|LSCF, with explicit layer labels and quantitative thicknesses (anode: ~20 μm; GDC: ~5 μm; SSZ: ~300 μm; LSCF: ~20 μm). High-magnification interface insets (**Fig. R16 and 17**) confirm no delamination or cracks, validating full-device structural integrity.

All images are standardized for contrast/brightness, with revised captions detailing scale bars and layer parameters. We thank the reviewer for their scrutiny, which strengthens the rigor of our experimental presentation.

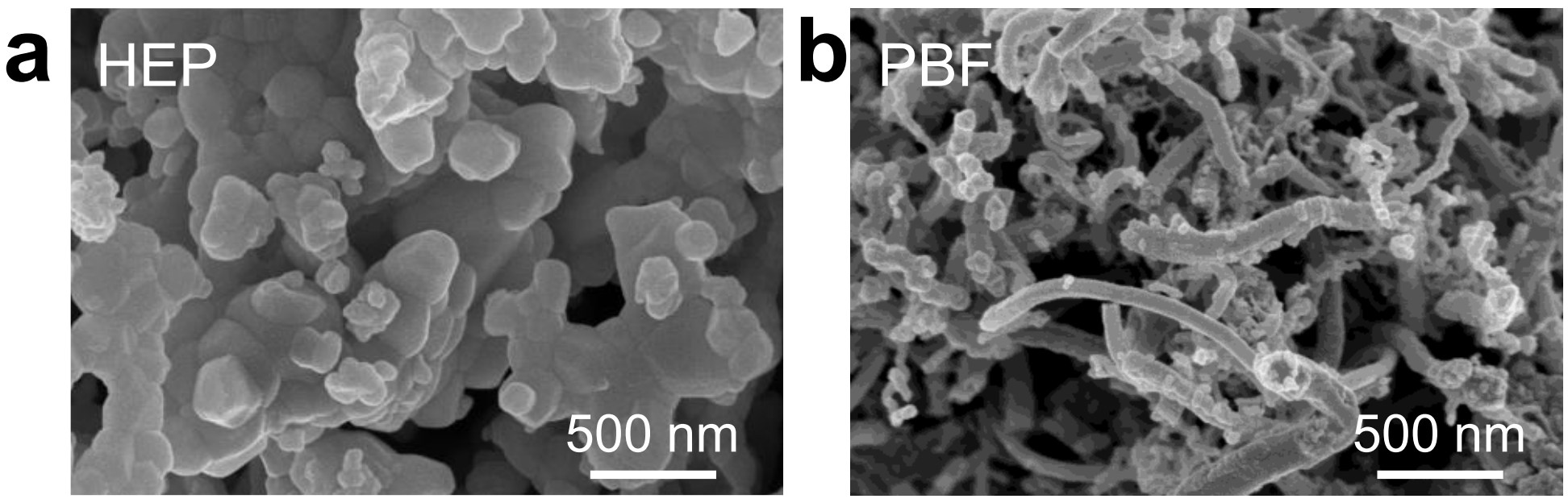

Fig. R31 (also **Fig. 4b, c**) Cross-sectional SEM images of (**a**) HEP and (**b**) PBF anode after 1000 h durability testing under 800 mA cm^{-2} at 700 °C.

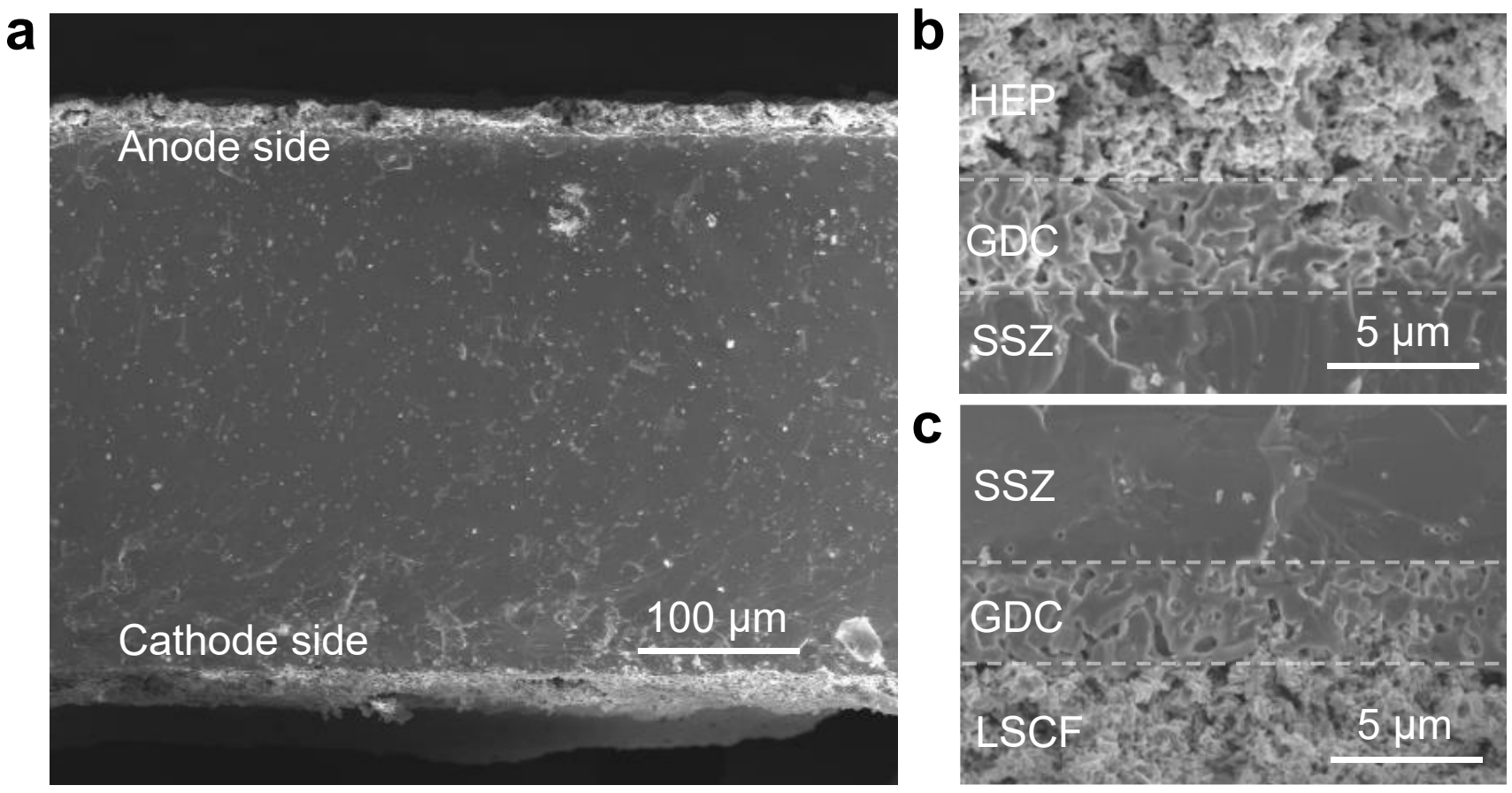

Fig. R16 (also **Fig. S27**). **a** Cross-Wsectional view of the SSZ-based single cell with a configuration of HEP|GDC|SSZ|GDC|LSCF. **b** Magnified view of the anode side (HEP|GDC|SSZ). **c** Magnified view of the cathode side (SSZ|GDC|LSCF).

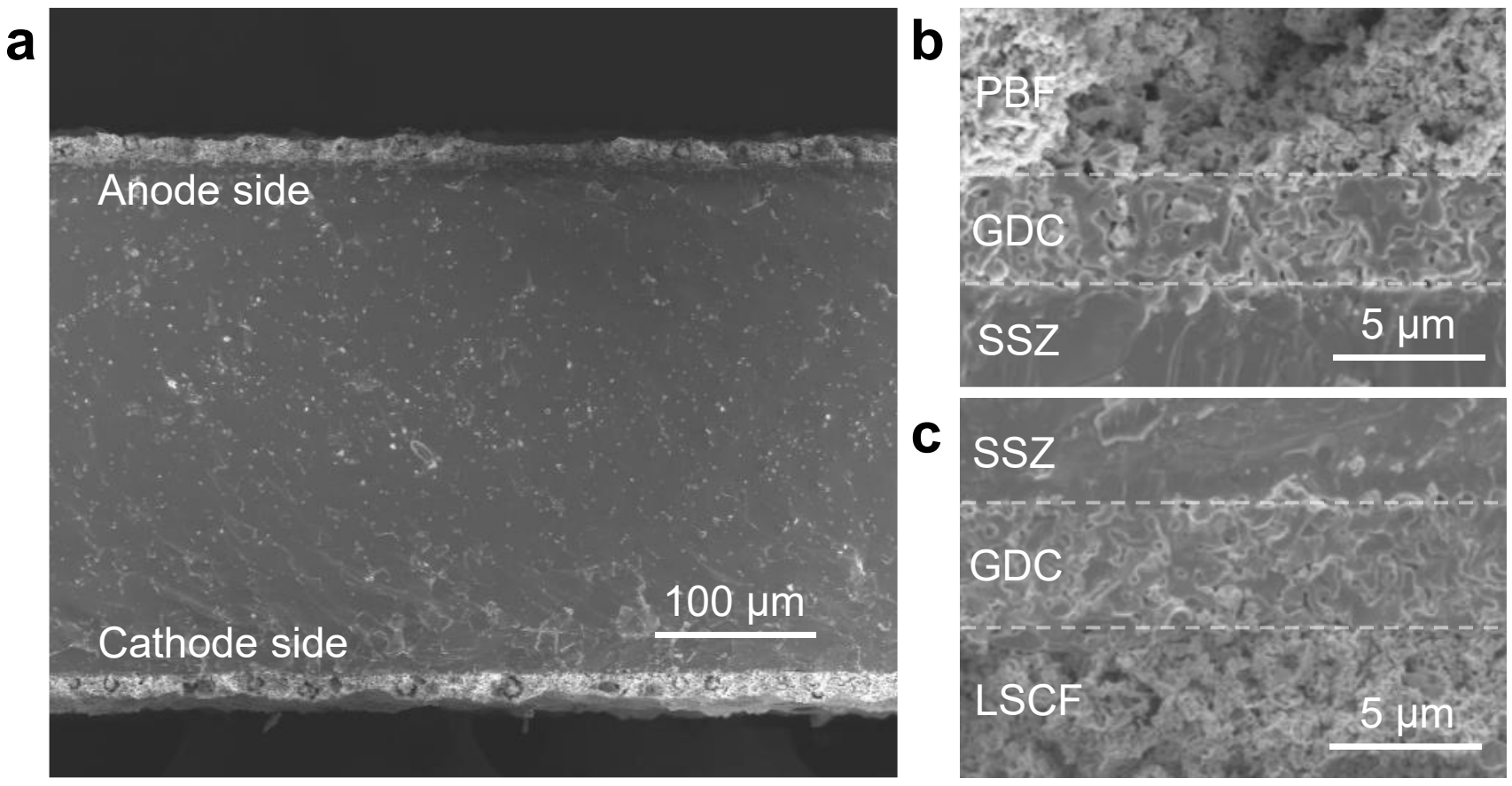

Fig. R17 (also **Fig. S29**). **a** Cross-Wsectional view of the SSZ-based single cell with a configuration of PBF|GDC|SSZ|GDC|LSCF. **b** Magnified view of the anode side (PBF|GDC|SSZ). **c** Magnified view of the cathode side (SSZ|GDC|LSCF).

3-5-(II)

Synchrotron measurement details: The wavelength used for the synchrotron XRD measurements (Fig. 1a) should be explicitly stated to ensure reproducibility and clarity

Response to 3-5-(II):

We sincerely appreciate the reviewer for this thoughtful comment, which enhances the reproducibility and clarity of our experimental procedures. The wavelength employed for the synchrotron X-ray powder diffraction (SXRPD) measurements (**Fig. 1a**) is 0.6888 Å, and this key detail has been explicitly supplemented in the "Experimental Methods" section of the revised Supplementary Information to ensure full transparency and reproducibility of the work.

We thank the reviewer again for their scrutiny, which helps refine the completeness of our experimental reporting.

Related discussions have been added in the revised Supplementary information as follows:

Synchrotron X-ray powder diffraction (SXRPD) measurements, performed with a wavelength of 0.6888 Å, were carried out at the BL02U beamline of the Shanghai Synchrotron Radiation Facility (SSRF).

3-5-(III)

Structural analysis: The authors perform Rietveld refinement using laboratory XRD data despite having access to synchrotron XRD measurements. A justification for this choice should be provided, or alternatively, Rietveld refinement of the synchrotron data should be included to take full advantage of the higher resolution.

Response to 3-5-(III):

We sincerely appreciate the reviewer for this professional and insightful comment, which helps us further improve the rigor and completeness of the structural analysis in our study. Your suggestion to take full advantage of the higher resolution of synchrotron XRD data is highly valuable and has been carefully addressed in our revisions.

The core purpose of our XRD characterization is to accurately determine whether the synthesized perovskite materials form a layered structure, and the key criterion for judging a layered perovskite structure is the presence of split peaks in the XRD pattern [Yu et al. *J. Am. Chem. Soc.*, **30**, 27000–27010 (2025)]. In laboratory XRD measurements, we used dichromatic light from a Cu Kα source (wavelengths: $K\alpha_1$ = 1.54056 Å, $K\alpha_2$ = 1.54439 Å), which has a relatively long wavelength. This longer wavelength leads to a wider full width at half maximum of the diffraction peaks, making the split peaks of the layered structure prone to merging. In addition, the dichromatic radiation can lead to peak splitting, where a single diffraction peak is artificially split into two peaks in the XRD pattern. Consequently, the characteristic split peaks cannot be clearly distinguished, which hinders the accurate identification of the layered structure. In contrast, the synchrotron XRD measurements we performed used a wavelength of 0.6888 Å, which effectively avoids the problem of split peak merging caused by long wavelengths. This shorter wavelength ensures higher diffraction resolution, allowing us to clearly observe the split peaks characteristic of the layered perovskite structure and accurately confirm the structural characteristics of the materials. Therefore, we chose synchrotron XRD data for the structural identification of layered perovskites.

In response to your suggestion, we have supplemented the Rietveld refinement results of the SXRPD data in the revised manuscript (**Fig. R32**). The unit cell parameters of PBF refined

by SXRPD are a = b = 3.9327 Å and c = 7.9154 Å, while those of HEP are a = b = c = 3.9333 Å. These refinement results are similar to those obtained from laboratory XRD, and as requested, the corresponding refined results have been updated in the revised manuscript.

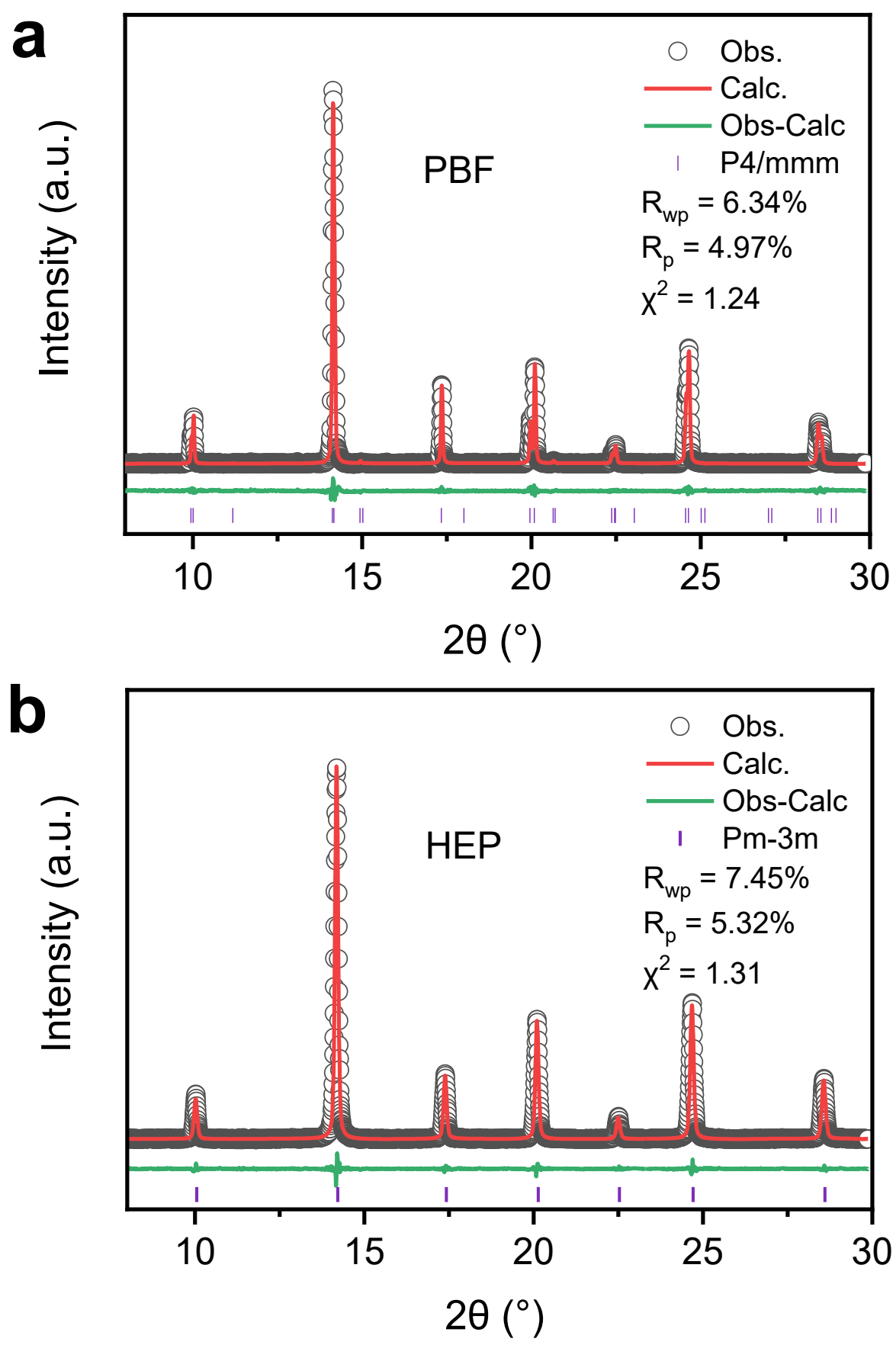

Fig. R32 (also **Fig. S2**) Rietveld refinement of (**a**) PBF and (**b**) HEP SXRPD data.

We would like to express our sincere gratitude to the reviewer again for this instructive comment. Your suggestion has helped us fully exploit the value of synchrotron XRD data and significantly enhance the scientific rigor of the structural analysis in our research.

Related discussions have been added in the revised Supplementary information as follows:

Line 128-130: Rietveld refinement of the SXRPD data (Fig. S2a and Table S1), performed in the space group P4/mmm, yielded lattice parameters of a = b = 3.9327 Å and c = 7.9154 Å.

Line 145: as further confirmed by XRD and the SXRPD refinement results.

Reviewer #4

The manuscript presents a well-structured investigation into the development of a high-entropy perovskite anode with the composition $La_{0.2}Pr_{0.2}Sm_{0.2}Gd_{0.2}Y_{0.2}BaFe_2O_{5+\delta}$ (HEP) for hydrocarbon-fueled solid oxide fuel cells (SOFCs), demonstrating promising catalytic activity and durability. A major revision is recommended. Before considering its publication, the following points should be adequately addressed:

Response:

We sincerely appreciate the reviewer for the positive evaluation of our work and the constructive comments, as well as the recommendation of a major revision. Your insightful comments have precisely identified the aspects of our manuscript that require further improvement and optimization, which are of great guiding significance for us to enhance the scientific rigor, data completeness and argument persuasiveness of the paper.

We attach great importance to all the comments raised by you, and have carefully studied and fully addressed each point through supplementary key experiments, perfecting mechanistic validation, optimizing characterization data presentation, and standardizing experimental detail reporting. Corresponding revisions have been made throughout the manuscript, and all newly added experimental data, characterization results and analysis discussions are detailed in the revised manuscript (with track changes for easy checking) and the attached supplementary information.

In this response, we will elaborate on the specific revision measures, supplementary research content and relevant experimental results for each comment one by one. We sincerely hope the revised manuscript and detailed response can meet your requirements, and we kindly request your further review and evaluation.

Again, we would like to express our sincere gratitude for your valuable time, professional guidance and constructive suggestions on our work.

Comment 4-1

(1) Introduction

A more detailed discussion on high-entropy materials, particularly in the context of SOFC anode applications, is recommended. The rationale behind the selection of the five A-site elements (La, Pr, Sm, Gd, and Y) should be clarified. For instance, would alternative combinations of five elements yield comparable or superior performance? How would the properties be affected if only three or four elements were used at the A-site?

Response to 4-1:

We sincerely appreciate the reviewer for this insightful and constructive comment, which has guided us to further enrich the content of the introduction and clarify the material design rationale of our work. We have fully addressed the concerns raised by supplementing relevant discussions and experimental data in the revised manuscript, and the details are elaborated as follows.

In response to the suggestion of a more detailed discussion on high-entropy materials for SOFC anode applications, we have added targeted content in the Introduction section of the revised manuscript, highlighting that high-entropy engineering has become a powerful strategy for regulating the atomic structure and electrochemical properties of functional oxides, and its unique configurational entropy-driven structural modulation effect can effectively regulate cation ordering and defect chemistry in perovskite materials, which thus lays a theoretical foundation for the design of high-entropy perovskite SOFC anodes. Meanwhile, to clarify the rationale for selecting La, Pr, Sm, Gd and Y as the five A-site rare-earth elements, we have added a dedicated Supplementary Note 1 in the revised Supplementary Information, which elaborates the selection considerations from three key aspects: sufficient literature support for their stable existence in layered double perovskite lattices, practical feasibility of being non-radioactive, low-cost and easily accessible, and perfect alignment with our research objective of maximizing A-site configurational entropy to disrupt the original ordered layered structure.

To address your question regarding how properties are affected when only three or four rare-earth elements are used at the A-site, we have systematically synthesized and characterized

a series of A-site binary, ternary and quaternary rare-earth low/medium-entropy perovskite variants, including $La_{0.5}Pr_{0.5}BaFe_2O_{5+\delta}$ (LPBF, S_{config} = 0.69*R*), $La_{0.33}Pr_{0.33}Sm_{0.33}BaFe_2O_{5+\delta}$, (LPSBF, S_{config} = 1.10*R*) and $La_{0.25}Pr_{0.25}Sm_{0.25}Gd_{0.25}BaFe_2O_{5+\delta}$, (LPSGBF, S_{config} = 1.39*R*).

Structural and thermodynamic analyses demonstrate that configurational entropy increases with the number of A-site elements (**Fig. R21-25**). Three-element and four-element compositions do not generate sufficient configurational entropy to fully break the inherent A-site cation ordering. Only the five-element equimolar composition reaches the entropy threshold required for a complete order-to-disorder transition.

Electrochemical tests further show that reducing the number of A-site elements leads to higher polarization resistance and lower peak power density compared with HEP (**Fig. R26**). Even the quaternary composition, with partial disordering, cannot match the performance of the fully disordered five-element HEP. The lower performance of three-element and four-element variants arises from their limited oxygen vacancy formation, slower ion transport, and weaker surface hydration, all caused by incomplete disordering.

These results clearly confirm that reducing the number of A-site elements below five weakens the structural disordering and thus degrades the key electrochemical properties of the anode. This directly supports our choice of a five-element equimolar high-entropy composition to achieve optimal anode performance.

We would like to express our sincere gratitude again for the reviewer's valuable suggestion, which has significantly improved the completeness of the introduction and the clarity of the material design rationale in our work. All the supplemented discussions, experimental data and supplementary notes have been clearly presented in the revised manuscript and Supplementary Information, and we kindly request your further review.

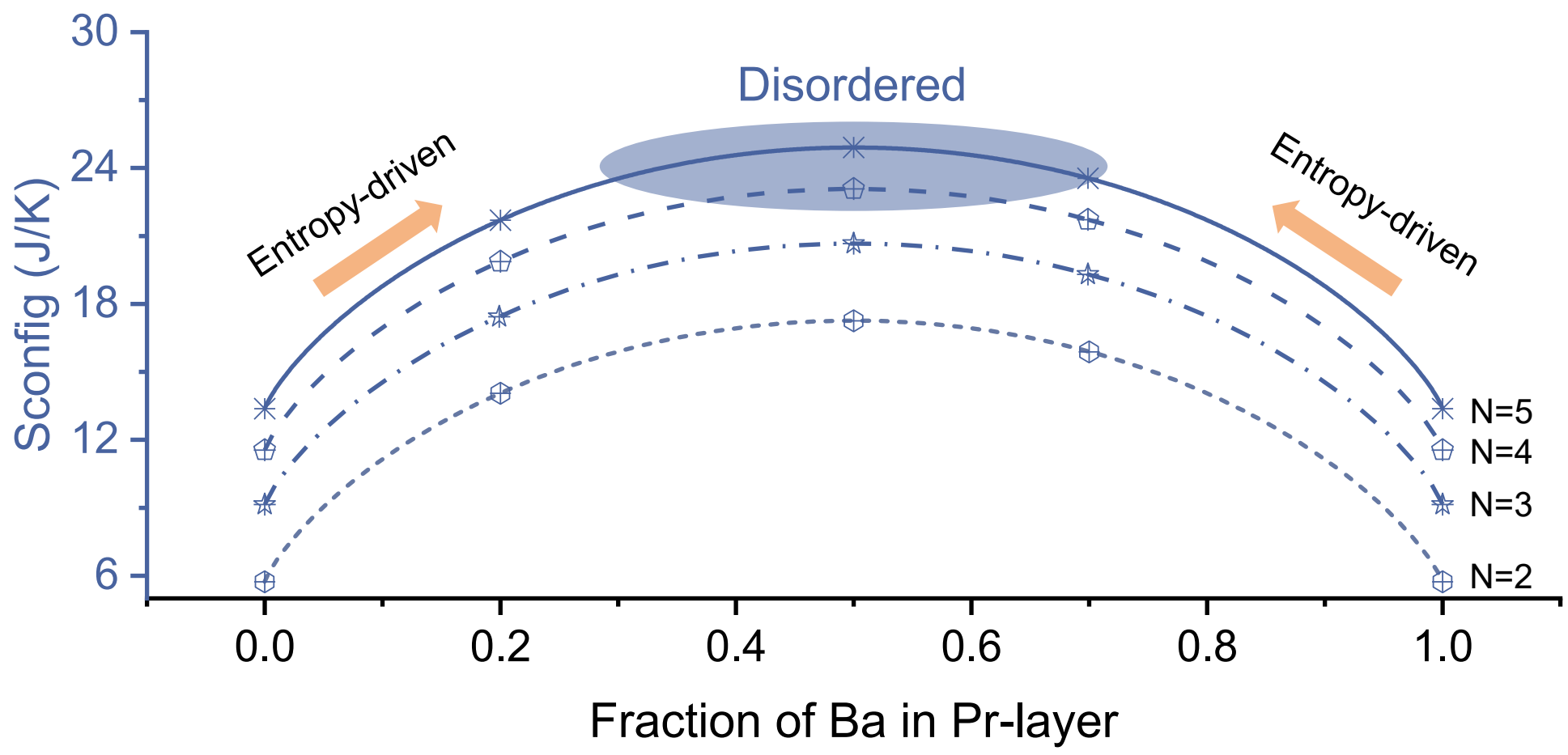

Fig. R21 Entropy of different perovskites and system energy values as a function of fraction of Ba in Pr-layer.

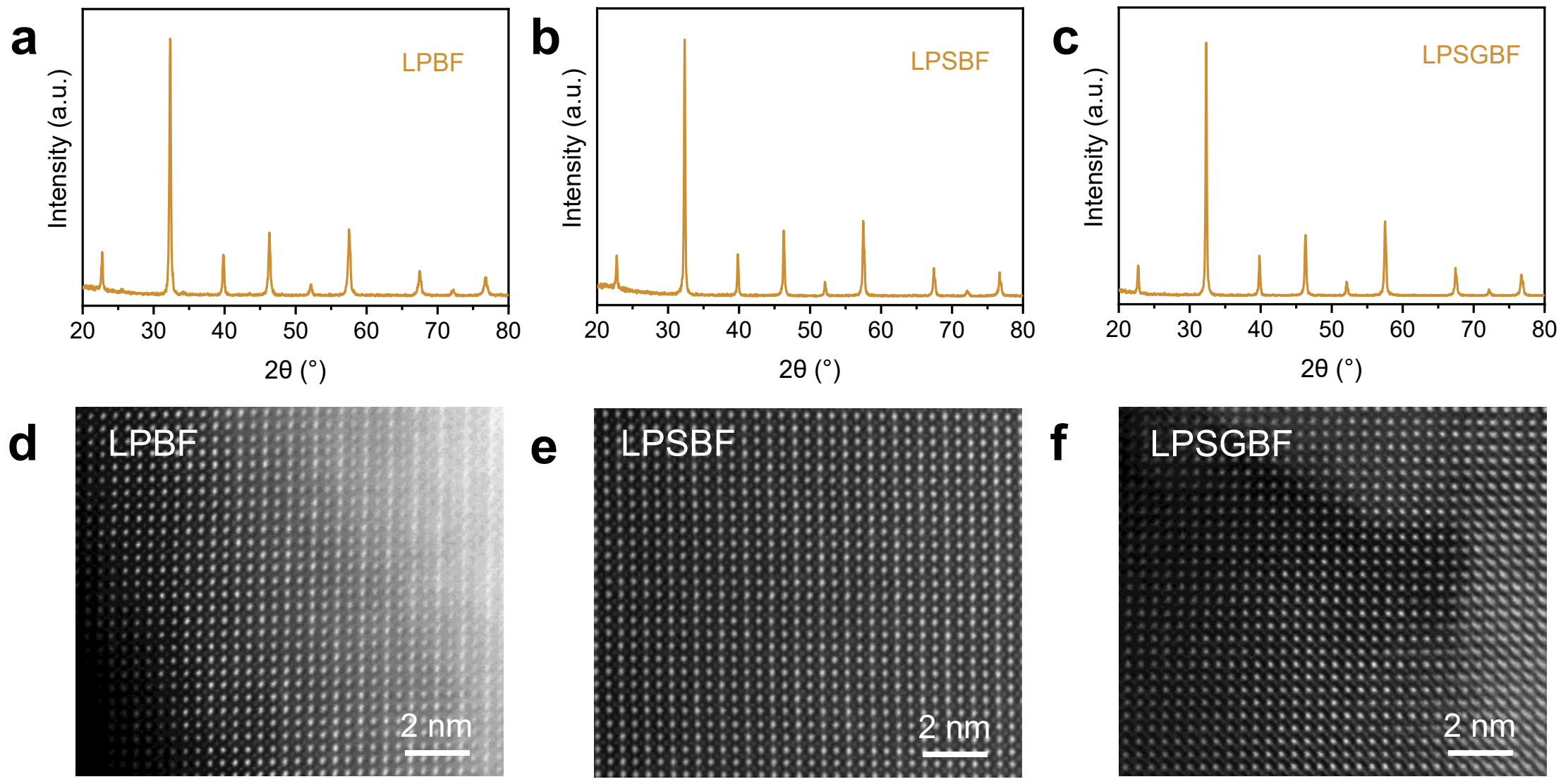

Fig. R22 (also **Fig. S12**) XRD patterns at RT of (**a**) LPBF, (**b**) LPSBF and (**c**) LPSGBF. HAADF-STEM images of (**d**) LPBF, (**e**) LPSBF and (**f**) LPSGBF.

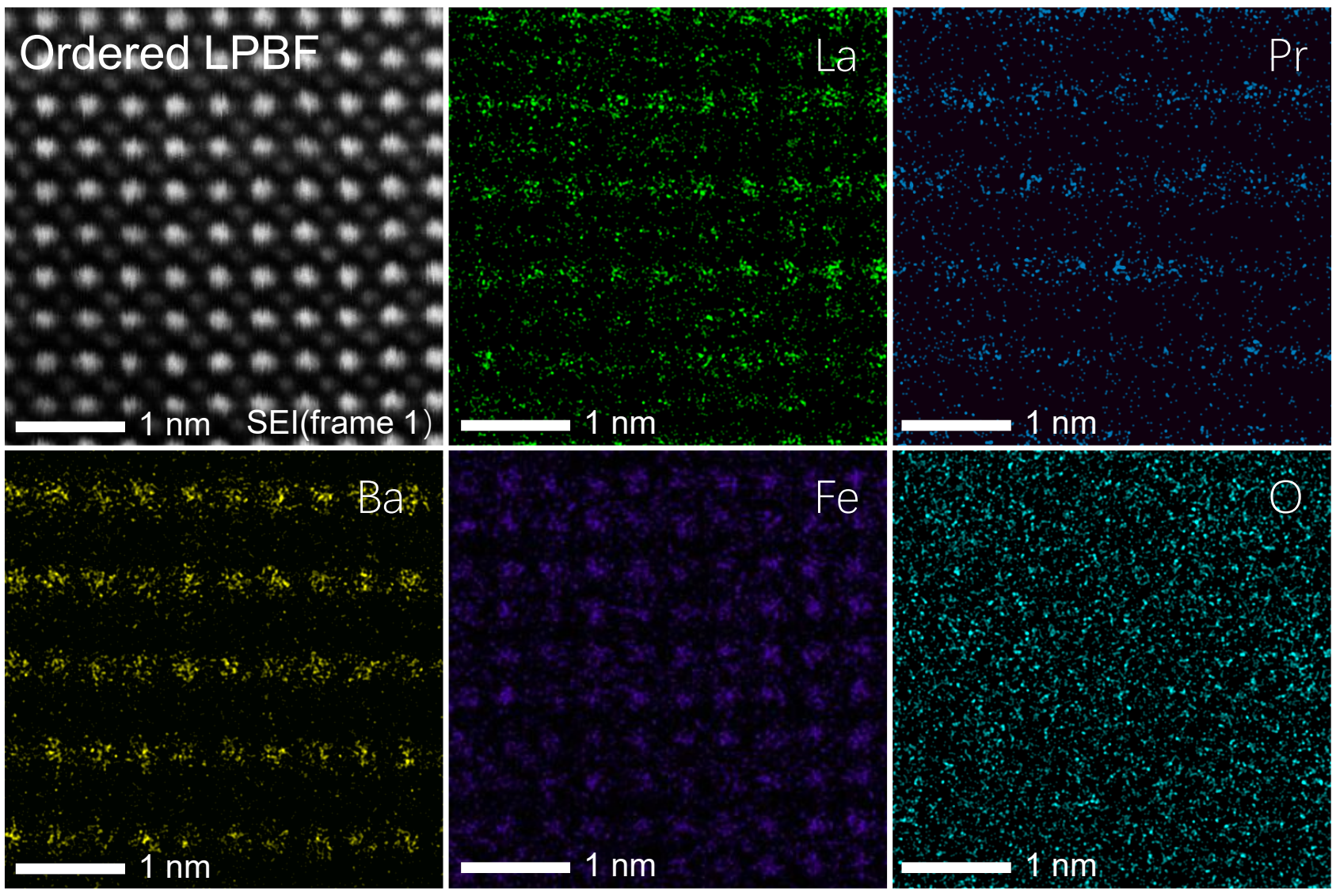

Fig. R23 (also **Fig. S13**) HAADF-STEM elemental maps of LPBF.

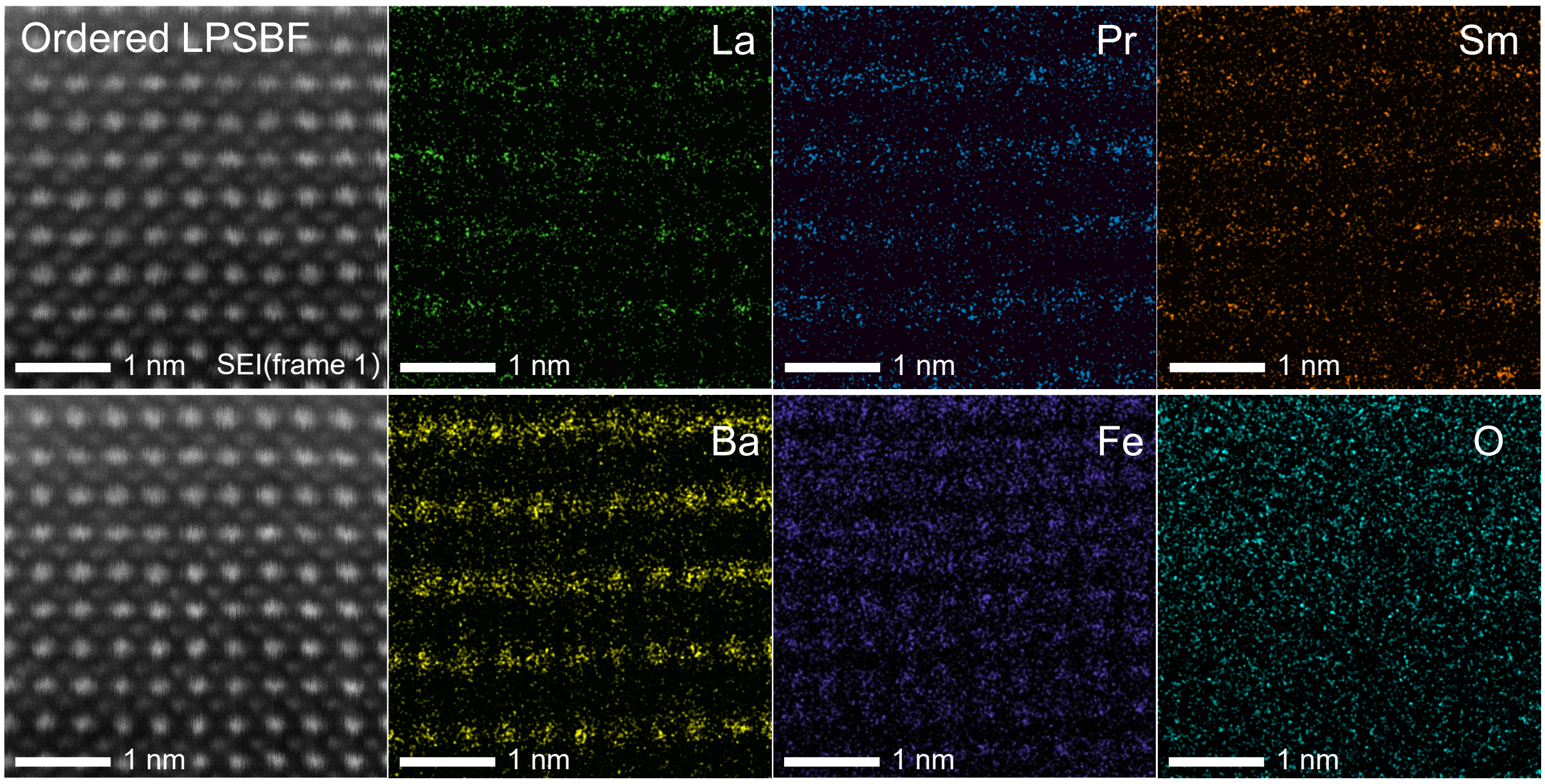

Fig. R24 (also **Fig. S14**) HAADF-STEM elemental maps of LPSBF.

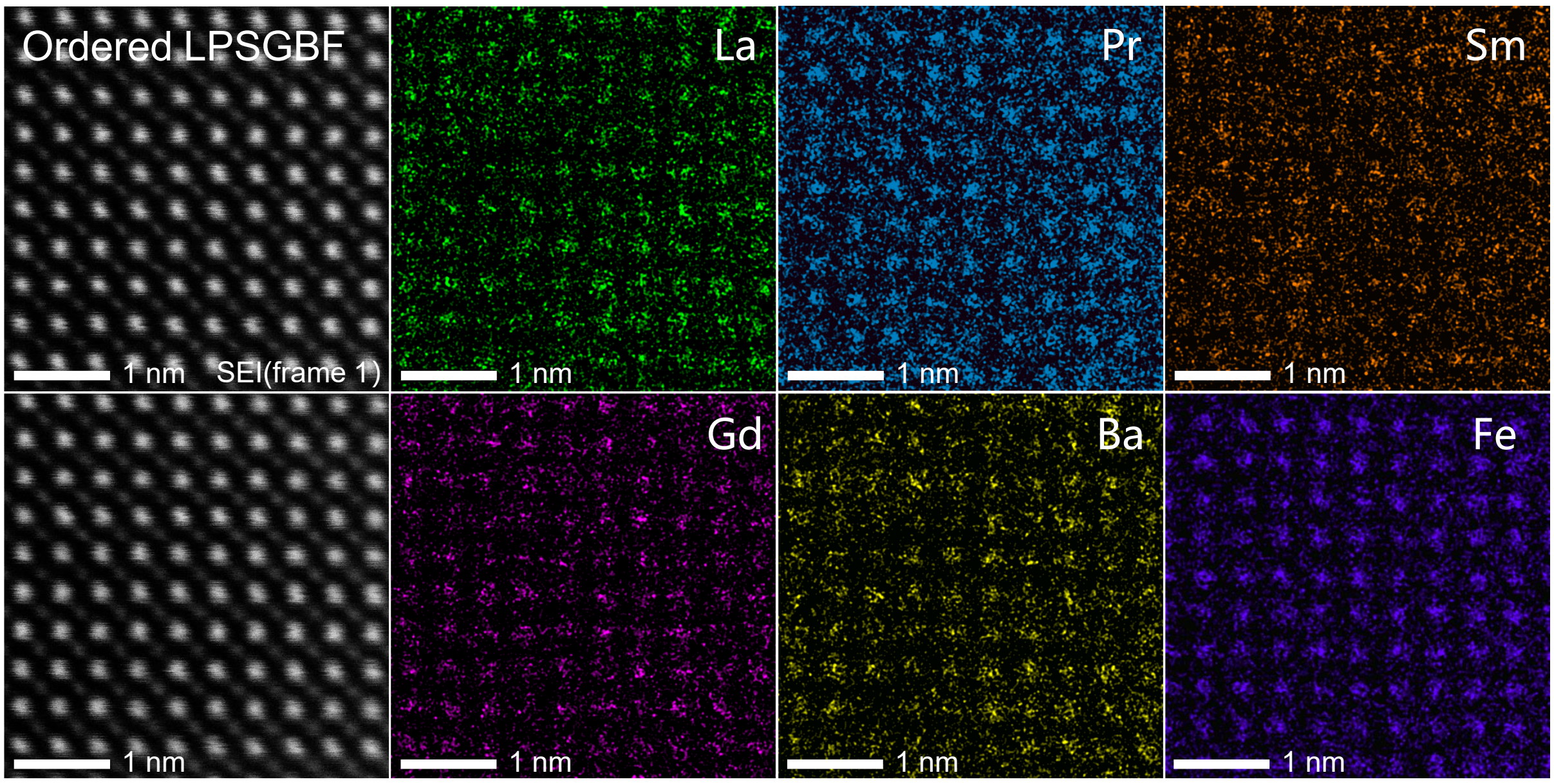

Fig. R25 (also **Fig. S15**) HAADF-STEM elemental maps of LPSGBF.

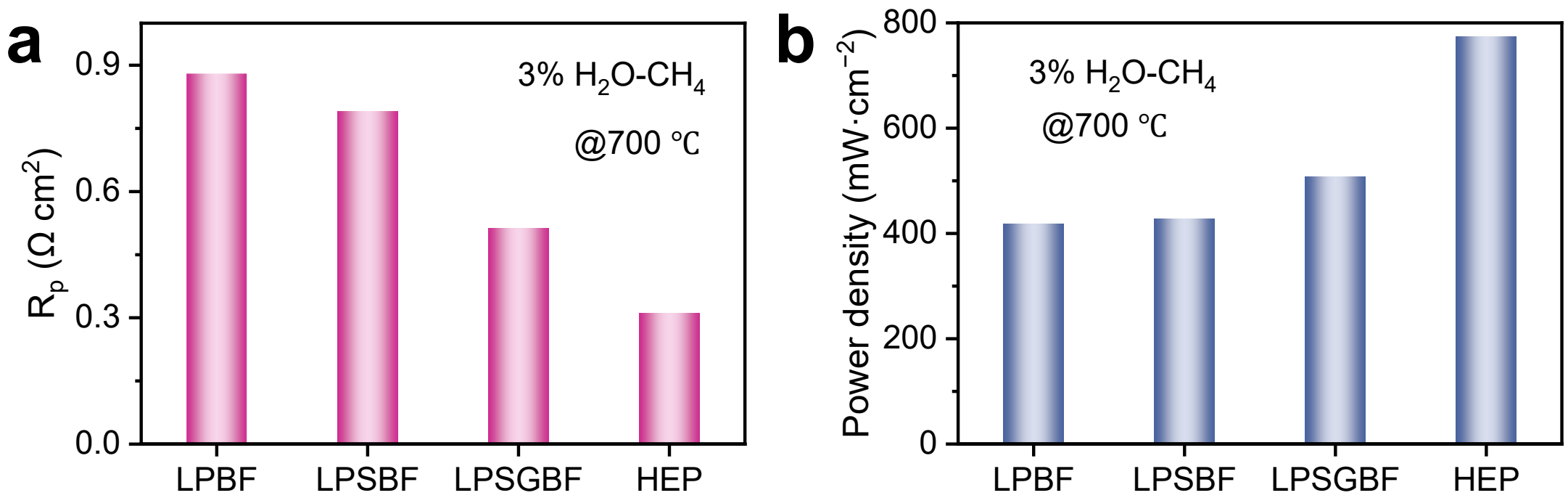

Fig. R26 a EIS plots of anodes under 3% H_2O-CH_4 at 700 °C. **b** I−V−P curves of single cells using different anodes under 3% H_2O-CH_4 at 700 °C.

Related discussions have been added in the revised manuscript and Supplementary information as follows:

Line 98-101: Recently, high-entropy engineering has emerged as a powerful strategy for tailoring the atomic-scale structure and electrochemical properties of functional oxides, with its unique configurational entropy-driven structural modulation effect enabling the rational regulation of cation ordering and defect chemistry in perovskites.[33,34]

Line 107: (The design rationale of this material is provided in Supplementary Note 1)

Supplementary Note 1

Design Rationale of the High-Entropy Perovskite Anode Material

La, Pr, Sm, Gd, Y are selected as equimolar A-site rare-earth elements for constructing $La_{0.2}Pr_{0.2}Sm_{0.2}Gd_{0.2}Y_{0.2}BaFe_2O_{5+\delta}$ (HEP) based on three key considerations:

These elements have been successfully incorporated into layered double perovskite lattices in previous studies, verifying their stable existence without severe lattice distortion or phase decomposition, and providing a reliable experimental foundation for synthesis.[5-8]

Practical feasibility. As non-radioactive rare-earth elements with low cost and easy accessibility, their selection ensures experimental repeatability and potential for subsequent practical application.

Alignment with research objectives. The core goal of this work is to elucidate configurational entropy regulation on Ln/Ba ordering and electrochemical properties. Equimolar doping of five elements maximizes A-site configurational entropy, which is the key to complete disruption of the original ordered layered structure and formation of a disordered high-entropy perovskite, directly serving the study of entropy-driven structural modulation mechanisms.

Line 193-204: To further understand the relationship between configurational entropy and order-to-disorder transitions, the number of A-site elements was reduced to lower the configurational entropy of the perovskite. A series of A-site binary, ternary, and quaternary rare-earth low/medium-entropy perovskite derivatives were synthesized and investigated, including $La_{0.5}Pr_{0.5}BaFe_2O_{5+\delta}$ (LPBF, S_{config} = 0.69R), $La_{0.33}Pr_{0.33}Sm_{0.33}BaFe_2O_{5+\delta}$ (LPSBF, S_{config} = 1.10R), $La_{0.25}Pr_{0.25}Sm_{0.25}Gd_{0.25}BaFe_2O_{5+\delta}$ (LPSGBF, S_{config} = 1.39R). XRD characterizations (Fig. S6a–c) confirm that all these samples adopt a pure single-phase perovskite structure. HAADF-STEM (Fig. S6d–f) and EDS elemental mappings (Fig. S7–9) reveal a clear entropy-dependent structural evolution. Binary LPBF and ternary LPSBF with lower entropy retain the

typical layered ordering of the parent PBF, whereas the high-entropy quaternary LPSGBF exhibits a disordered structure. These results confirm that configurational entropy acts as the key driving force regulating the A-site ordering in layered double perovskites.

Line 336-340: To further understand the effect of the order-to-disorder transition on electrochemical performance, we also compared the EIS spectra of perovskites with different entropy values (LPBF, LPSBF, and LPSGBF). As shown in Fig. S26, the results reveal that disordered HEP exhibit the lowest R_p values. This indicates that anodes with a disordered structure possess superior electrochemical properties.

Line 353-356: Compared to the anodes with different S_{config} (LPBF, LPSBF, and LPSGBF) and the most recent advancements in the field (Fig. 3c, S30, and Table S5), our disordered HEP anode exhibits significantly superior power density performance when benchmarked against both single-phase and ordered double perovskite oxide systems.

Comment 4-2

(2) Material stability

Regarding Figure S6, please provide an explanation for the disappearance of the diffraction peak at approximately 77° after 80 hours of reduction.

Response to 4-2:

We sincerely appreciate the reviewer for their meticulous observation regarding the diffraction peak at approximately 77° in **Figure S6**, and we would like to apologize sincerely for any confusion caused by the initial presentation of this data. We fully acknowledge that the apparent disappearance of this diffraction peak after 80 h of reduction treatment in the original figure was unclear and insufficiently rigorous, which was our oversight in the initial data collection and presentation.

Upon careful re-examination of our experimental methodology and data, we identified that the ambiguity originated from suboptimal parameters in the initial XRD measurement: the fast scan rate of 20 °/min, combined with the inherently weak peak intensity after prolonged reduction, obscured the diffraction peak within background noise and created a false impression of its disappearance. To ensure data accuracy and rigor, we repeated the XRD measurement on the 80-hour reduced sample with a significantly slower scan rate of 3 °/min to enhance resolution, and the new pattern clearly confirms the presence of the peak at approximately 77°, verifying the stability of the main phase after long-term reduction. The updated XRD pattern has replaced the original Figure S6 in the revised Supplementary Information (now **Fig. R33**) to eliminate any further confusion.

We would like to express our deepest gratitude again for the reviewer's rigorous scrutiny and valuable feedback. Your careful observation has helped us identify and correct the deficiency in our initial data, significantly improving the scientific rigor and reliability of our dataset. We will be more meticulous in data collection and presentation in future research, and we sincerely appreciate the reviewer's guidance in enhancing the quality of our work.

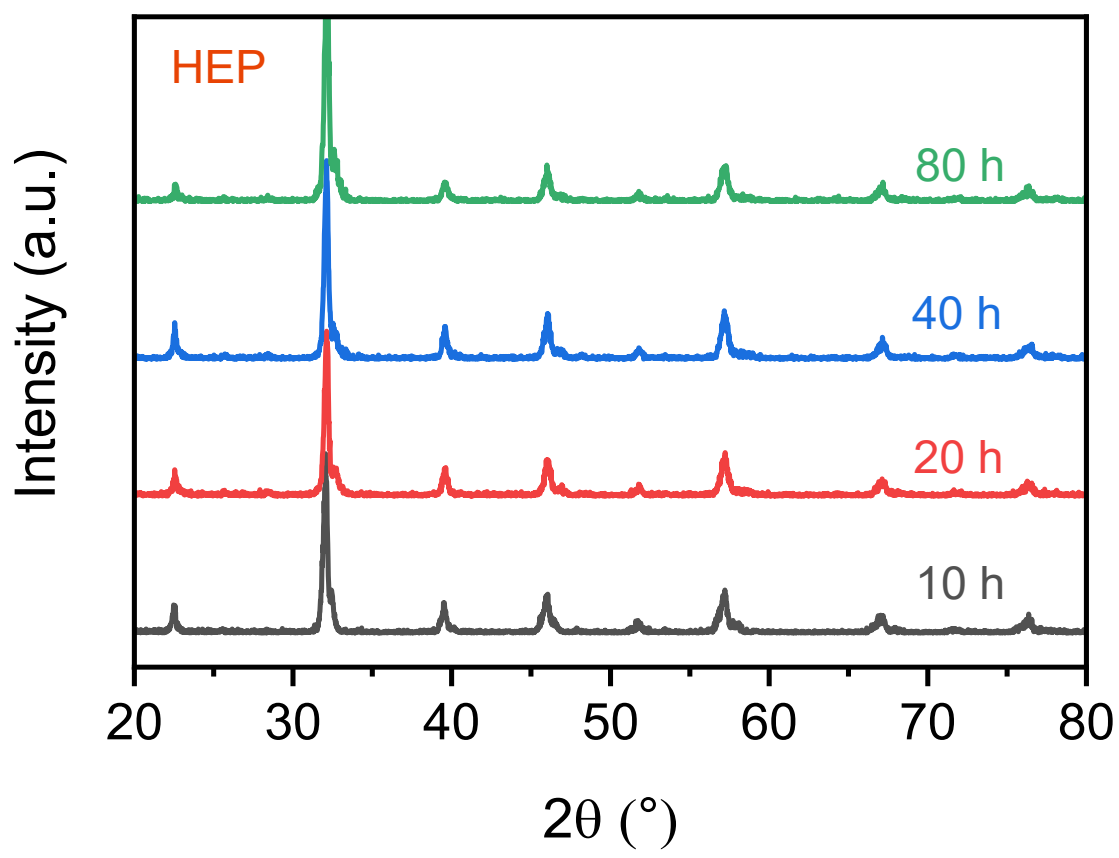

Fig. R33 (also **Fig. S16**) XRD patterns of HEP reduced at 700 °C in 50 vol% CH_4/Ar for different time.

Comment 4-3

(3) Mechanism elucidation

Beyond the disordered structure, are there other contributing factors—such as surface chemistry, coordination environment, or local lattice distortions—that could account for the enhanced anode performance?

Response to 4-3:

We sincerely appreciate the reviewer for this thoughtful and comprehensive comment, which helps us establish a more complete and multi-dimensional mechanism for the excellent performance of HEP anode. In addition to entropy-driven cation disorder, we have systematically verified the contributions from surface chemistry, coordination environment and local lattice distortion. All these factors are intrinsically induced by the disordered structure and work together to boost the anode performance.

1. Surface chemistry

Surface chemistry directly affects the surface catalytic activity of the anode. HEP shows more favorable surface chemistry characteristics including higher surface-active oxygen vacancy concentration, stronger water adsorption and dissociation ability, and higher methane reforming activity. These surface properties effectively promote fuel activation and surface oxidation reactions, and also improve the anti-coking ability of the anode. Such advantageous surface chemistry is derived from the entropy-induced cation disorder that regulates surface defect concentration and surface activity.

2. Coordination environment

For the coordination environment, we used synchrotron radiation X-ray absorption fine structure to analyze the local coordination of Fe. The results prove that the average coordination number of Fe in HEP is lower than that in PBF. The reduced coordination number of Fe directly confirms the higher oxygen vacancy concentration in HEP, which effectively promotes oxygen ion migration and surface exchange kinetics. This change in coordination environment is a direct structural result of A-site cation disorder.

3. Local lattice distortions

Lattice strain is a common structural feature in high-entropy oxide systems. To rule out its role as a dominant factor for the enhanced oxygen vacancy-related properties and electrochemical performance of HEP, we constructed a theoretical model of ordered-HEP, whose A-site cations adopt the same ordered layered arrangement as PBF, for comparative DFT calculations of Fe–O bond energy with disordered HEP and PBF (**Fig. R26**). The calculated Fe–O bond energy values are 6.96 eV for computationally designed ordered-HEP, 6.34 eV for experimentally disordered HEP, and 7.19 eV for PBF. It is noteworthy that lattice strain is also present in the ordered-HEP theoretical model, yet its Fe–O bond energy is significantly higher than that of disordered HEP. A higher Fe–O bond energy indicates a stronger metal-oxygen interaction in the lattice and a higher energy barrier for oxygen vacancy formation. This theoretical calculation result directly demonstrates that the optimized oxygen vacancy chemistry of HEP is not derived from lattice strain, but from the entropy-driven A-site cation order-to-disorder transition of the layered double perovskite structure.

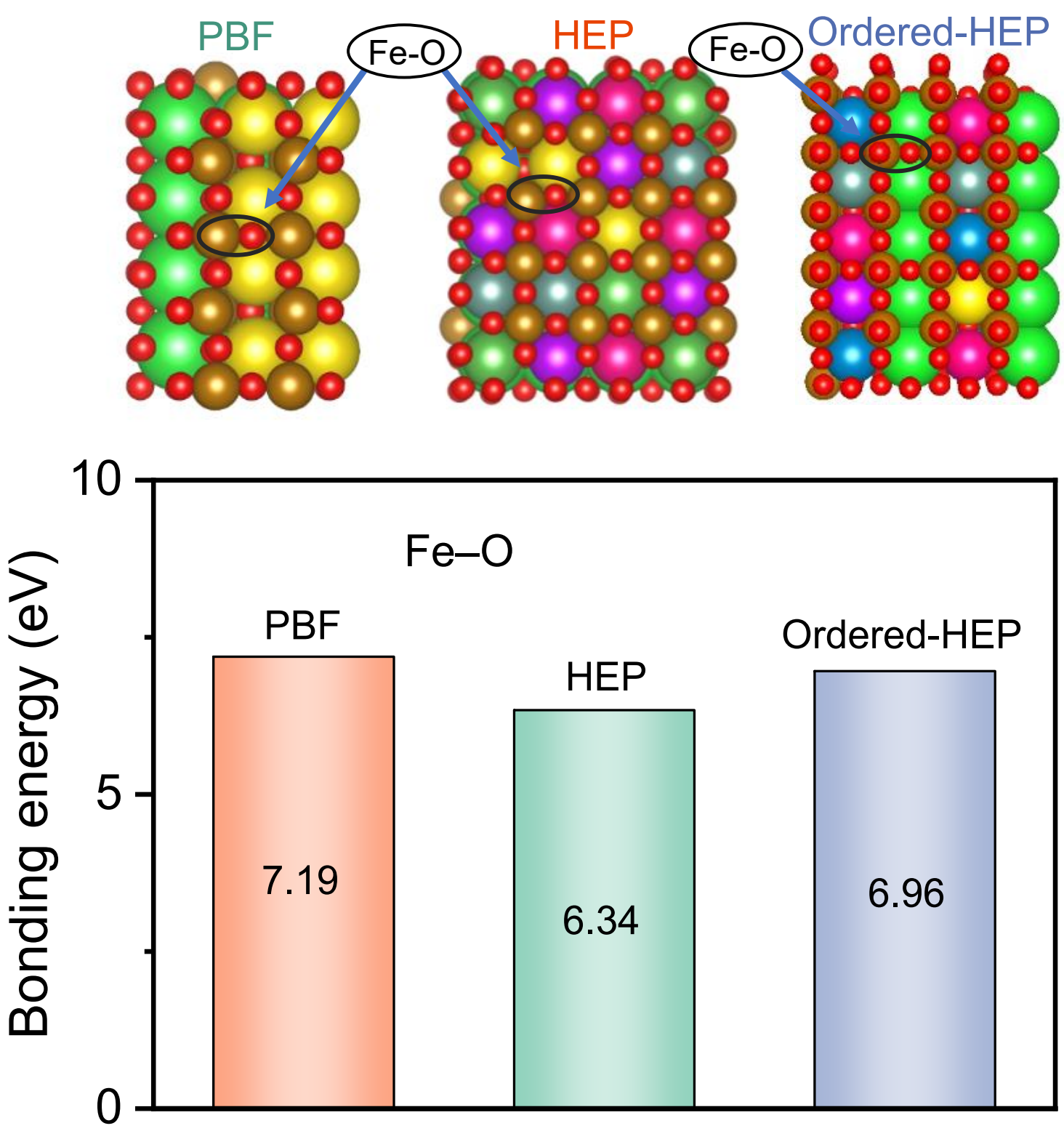

Fig. R27 (also **Fig. S18**) Comparison of the Fe–O bond energies of different anode.

Related discussions have been added in the revised manuscript as follows:

Line 245-251: To further verify the influence of cation ordering on the Fe–O bond energy, we constructed an ordered high-entropy perovskite model (computationally designed ordered HEP) with the same unit-cell parameters as the experimentally disordered HEP, as illustrated in Fig. S18. The calculated Fe–O bond energy for computationally designed ordered-HEP is 6.96 eV, which is significantly higher than that of experimentally disordered HEP (6.34 eV). This directly demonstrates that the order-to-disorder transition can promote the formation of oxygen vacancies.

Comment 4-4

(4) Performance comparison

In Figure 3c, several high-performance results from the literature are omitted. To ensure a fair comparison, it is essential to include cells with similar configurations (e.g., the anode-supported cells normally show higher performance than the electrolyte-supported ones), electrolyte composition, and electrolyte thickness. A separate table summarizing these cell parameters is recommended.

Response to 4-4:

We sincerely appreciate the reviewer for this meticulous and valuable comment, which helps us improve the fairness and comprehensiveness of the performance comparison in **Fig. 3c**. Your suggestion on standardizing the comparison criteria and supplementing relevant parameters is of great guiding significance for enhancing the scientific rigor of our study.

In the initial version of **Fig. 3c**, the performance comparison of anode materials is strictly limited to the temperature range of 600–800 °C. For literature reports with performance data beyond this temperature range, we omitted them intentionally to ensure a fair comparison, as significant temperature differences can lead to substantial discrepancies in cell performance and invalidate the comparability. To further improve the comprehensiveness and fairness of the comparison, we have supplemented three high-performance anode materials reported in recent literature. Specifically, we included the Fe-Cr metal-supported (highlighted in purple) anode reported by Liu et al., which achieves a power density of 820 $mW \cdot cm^{-2}$ at 700 °C with methane as fuel, using Ni-ScYSZ as the catalytic layer [Liu et al. *ACS Appl. Mater. Interfaces*, **14**, 53840−53849 (2022)]. In line with your recommendation, we have also prepared a separate table summarizing the key parameters of all compared cells, including cell configuration, electrolyte composition, electrolyte thickness, operating temperature, and fuel type, to facilitate a more intuitive and fair comparison.

We would like to express our sincere gratitude again for the reviewer's insightful feedback. Your scrutiny has helped us optimize the performance comparison part of our study, making the data more convincing and the discussion more rigorous. All the supplemented literature data,

the updated **Fig. 3c** (**Fig. R1**), and the separate parameter summary table (**Table R2**) have been integrated into the revised manuscript, and we kindly request your further review.

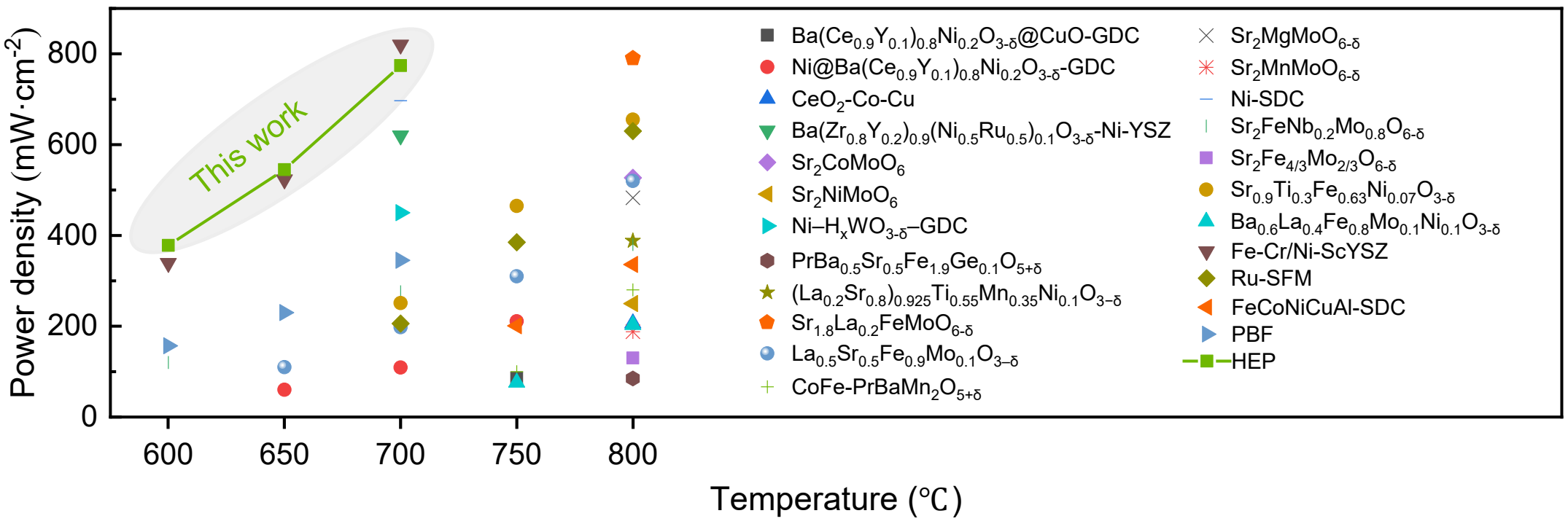

Fig. R1 (also **Fig. 3c**) Comparison of the PPDs of the SOFC technology with different anodes using methane as the fuel.

Table R2 (also **Table S5**). Performance and durability comparison of SOFC using methane as fuel in the range of 600-800 °C

Cell configuration	Electrolyte and thickness (μm)	Fuel mixture	Temperature (°C)	Peak power density (mW·cm^{-2})	Durability (h)	Ref.
$Ba(Ce_{0.9}Y_{0.1})_{0.8}Ni_{0.2}O_{3-\delta}$@CuO-GDC|GDC|LSCF	GDC (~20)	97% CH_4+3% H_2O	750	86	160	[9]
Ni@$Ba(Ce_{0.9}Y_{0.1})_{0.8}Ni_{0.2}O_{3-\delta}$-GDC|GDC|LSCF	GDC (—)	97% CH_4+3% H_2O	650<br>700<br>750	60<br>109<br>211	100 (750 °C)	[10]
CeO_2-Co-Cu|SSZ|LSM	SSZ (~150)	97% CH_4+3% H_2O	800	206.8	24	[11]
$Ba(Zr_{0.8}Y_{0.2})_{0.9}(Ni_{0.5}Ru_{0.5})_{0.1}O_{3-\delta}$-Ni-YSZ|YSZ|GDC|LSCF	YSZ (~15)	97% CH_4+3% H_2O	700	~620	430	[12]
Sr_2CoMoO_6|LDC|LSGM|SCF	LSGM (~300)	97% CH_4+3% H_2O	800	527	~17	[13]
Sr_2NiMoO_6|LDC|LSGM|SCF		97% CH_4+3% H_2O	800	~250	—	[13]
Ni–$H_xWO_{3-\delta}$–GDC |GDC|BSCF	GDC (—)	100% CH_4	700	~450	200	[14]
Ni–BZCCY|SDC|PBSCF	SDC (15)	97% CH_4+3% H_2O	500	370	550	[15]
$PrBa_{0.5}Sr_{0.5}Fe_{1.9}Ge_{0.1}O_{5+\delta}$ |GDC|SSZ|GDC|LSCF	SSZ (~100)	97% CH_4+3% H_2O	800	85	60	[16]
$(La_{0.2}Sr_{0.8})_{0.925}Ti_{0.55}Mn_{0.35}Ni_{0.1}O_{3-\delta}$ |LDC|LSGM|LSCF	LSGM (~350)	97% CH_4+3% H_2O	800	388	100	[17]
$Sr_{1.8}La_{0.2}FeMoO_{6-\delta}$ |GDC|YSZ|GDC|LSCF	YSZ (~300)	97% CH_4+3% H_2O	800	790	—	[18]

$La_{0.5}Sr_{0.5}Fe_{0.9}Mo_{0.1}O_{3-\delta}$|SDC<br>|LSGM|$Ba_{0.6}Sr_{0.4}Co_{0.9}Nb_{0.1}O_{3-\delta}$	LSGM (~280)	97% CH_4+3% H_2O	750<br>800	~310<br>~520		19
CoFe-$PrBaMn_2O_{5+\delta}$|LDC| LSGM|NBSCF50-GDC	LSGM (~300)	97% CH_4+3% H_2O	750<br>800	~100<br>~280	—	20
$Sr_2MgMoO_{6-\delta}$|LDC|LSGM|$SrCo_{0.8}Fe_{0.2}O_{3-\delta}$	LSGM (~300)	100% CH_4	800	483	—	20
Ni-SDC|SDC|SSC	SDC (—)	100% CH_4	700	697	100	21
$Sr_2FeNb_{0.2}Mo_{0.8}O_{6-\delta}$|LSGM|$PrBaCo_2O_{5+\delta}$	LSGM (~150)	97% CH_4+3% H_2O	600<br>700<br>800	120<br>276<br>380	200<br>(700 °C)	22
$Sr_2Fe_{4/3}Mo_{2/3}O_{6-\delta}$|LSGM|LSCF	LSGM (~300)	97% CH_4+3% H_2O	800	130	—	23
FeCr/Ni-ScYSZ|ScYSZ|GDC|LSC	ScYSZ (~8)	2% CH_4+98% Air	600<br>650<br>700	~339<br>~523<br>~820	1200<br>(600 °C)	24
Ru-SFM/Ru-GDC|LSGM|PBSCF/GDC	LSGM (~230)	97% CH_4+3% H_2O	700<br>750<br>800	~206<br>~385<br>~630	200<br>(800°C)	25
FeCoNiCuAl-SDC/SDC/LSGM/LSCF	LSGM (~300)	100% CH_4	750<br>800	~201<br>~336	20<br>(750)	26
$La_{0.6}Sr_{0.4}Ti_{0.3}Fe_{0.5}Co_{0.2}O_{3-\delta}$|LSGM|LSCF-SDC	LSGM (~600)	40% CH_4 + 55% Ar<br>+ 5% N_2	800	—	1250	27
$Sr_{0.9}Ti_{0.3}Fe_{0.63}Ni_{0.07}O_{3-\delta}$|LDC|LSGM|LDC|LSCF-GDC	LSGM (~200)	97% CH_4+3% H_2O	800<br>750<br>700	655<br>465<br>251	80<br>(800 °C)	28
$Ba_{0.6}La_{0.4}Fe_{0.8}Mo_{0.1}Ni_{0.1}O_{3-\delta}$|LSGM|LSCF	LSGM (~300)	100% CH_4	800<br>750	203<br>76	325<br>(800 °C)	29
HEP|GDC|SSZ|GDC|LSCF	SSZ (~300)	97% CH_4+3% H_2O	600<br>650<br>700	377.8<br>545.1<br>774.5	1000<br>(700 °C)	This Work
PBF|GDC|SSZ|GDC|LSCF	SSZ (~300)	97% CH_4+3% H_2O	600<br>650<br>700	157.2<br>230.1<br>345.8	—	This Work

Comment 4-5

(5) Electrochemical Impedance Spectroscopy (EIS)

EIS measurements under various fuel conditions and temperatures should be provided to facilitate a deeper understanding of the cell performance.

Response to 4-5:

We sincerely appreciate the reviewer for this constructive comment, which helps to deepen the understanding of the electrochemical performance of HEP and PBF anodes under practical SOFC operating conditions. In response to your suggestion, we have systematically supplemented comprehensive EIS measurements of HEP and PBF anodes across a full temperature range (550–700 °C) and various fuel atmospheres, and the corresponding data have been integrated into the revised manuscript and Supplementary Information for detailed presentation.

Specifically, we have tested the EIS performance of the two anodes under different atmospheres, including dry CH_4, 3% H_2O-CH_4, CH_3OH, C_2H_6, C_3H_8, n-C_4H_{10}, CO and H_2. The full EIS plots are provided in **Fig. R34** (also **Fig. S32 and S33**). These supplementary data directly and quantitatively reflect the electrochemical response of HEP and PBF anodes to different hydrocarbon fuels and temperatures, clearly demonstrating that HEP exhibits a consistently lower R_p than PBF under all tested conditions. The result further validates the superior intrinsic electrochemical activity and fuel adaptability of the HEP anode.

All supplementary EIS characterization data and detailed quantitative analysis have been elaborated in the revised manuscript (Line 374-377), provided in the Supplementary Information to ensure data transparency and reproducibility.

We thank the reviewer again for this valuable suggestion, which has significantly improved the completeness of our electrochemical performance characterization and provided more direct experimental evidence for the in-depth analysis of the anode's structure-performance relationship.

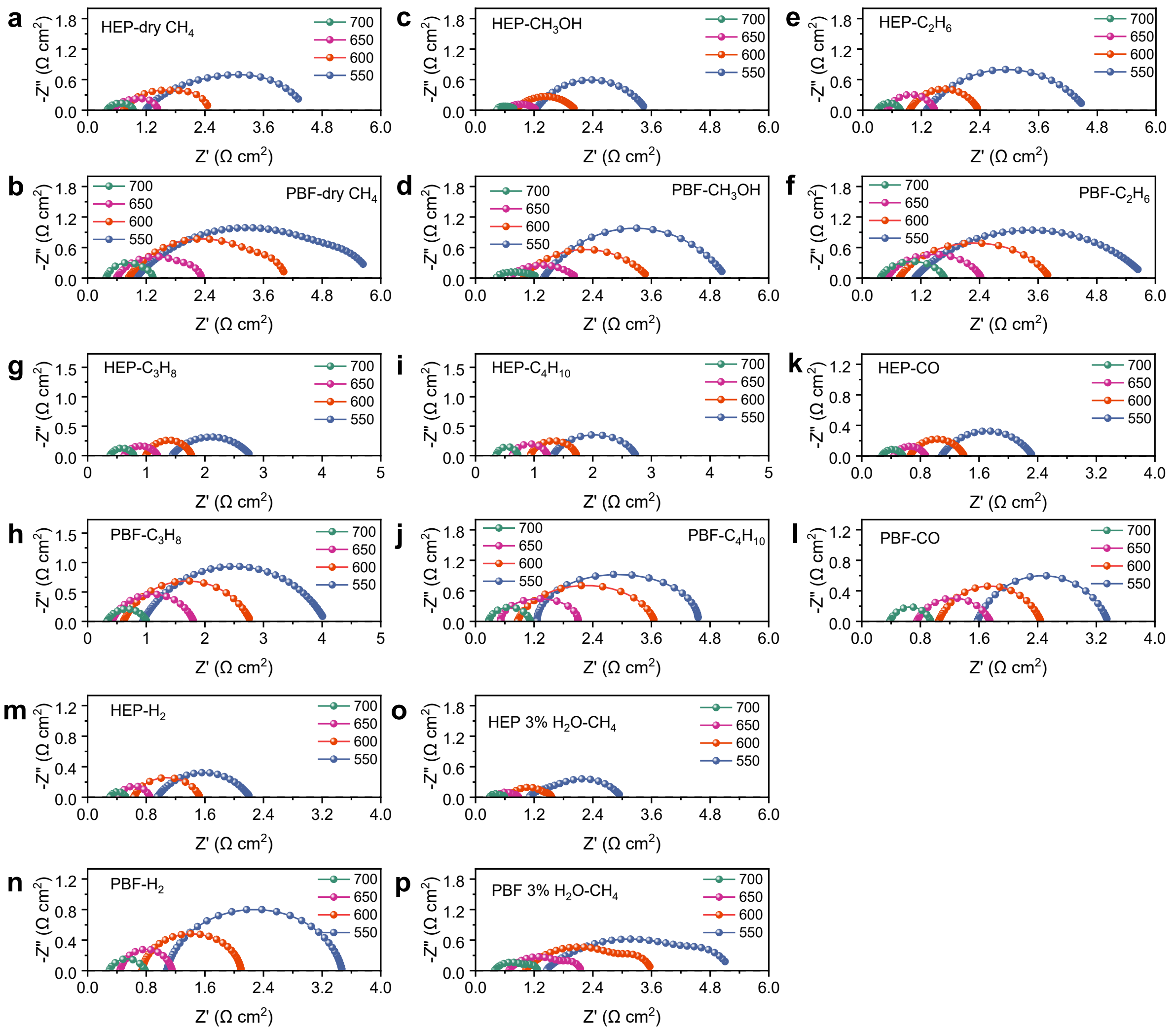

Fig. R34 (also **Fig. S32 and S33**) EIS plots of HEP and PBF under different atmosphere. (**a**) and (**b**) for dry CH_4. (**c**) and (**d**) for 3% H_2O-CH_3OH. (**e**) and (**f**) for 5% H_2O-C_2H_6. (**g**) and (**h**) for 10% H_2O-C_3H_8. (**i**) and (**j**) for 15% H_2O/n-C_4H_{10}. (**k**) and (**l**) for CO. (**m**) and (**n**) for H_2. (**o**) and (**p**) for 3% H_2O-CH_4.

Related discussions have been added in the revised manuscript as follows:

Line 374-377: Fig. S32 and S33 presents the EIS spectra of symmetrical cells collected under various fuel atmospheres. The results demonstrate that the HEP anode maintains a consistently lower R_p than the PBF anode under all tested conditions, further validating its superior intrinsic electrochemical activity and excellent fuel adaptability.

Comment 4-6

(6) Figure S17

Please specify the testing temperature used for the experiments in Figure S17.

Response to 4-6:

We thank the reviewer for raising this question.

The electrochemical performance measurements presented in **Fig. S17** (**Fig. R35**) were conducted at 700 °C. This temperature was selected to maintain consistency with the testing conditions used for evaluating performance with other fuels, thereby ensuring a fair and direct comparison. We have now explicitly noted this testing temperature in both the revised figure and its corresponding caption. We appreciate the reviewer's attention to this important detail, which has helped improve the clarity of our data presentation.

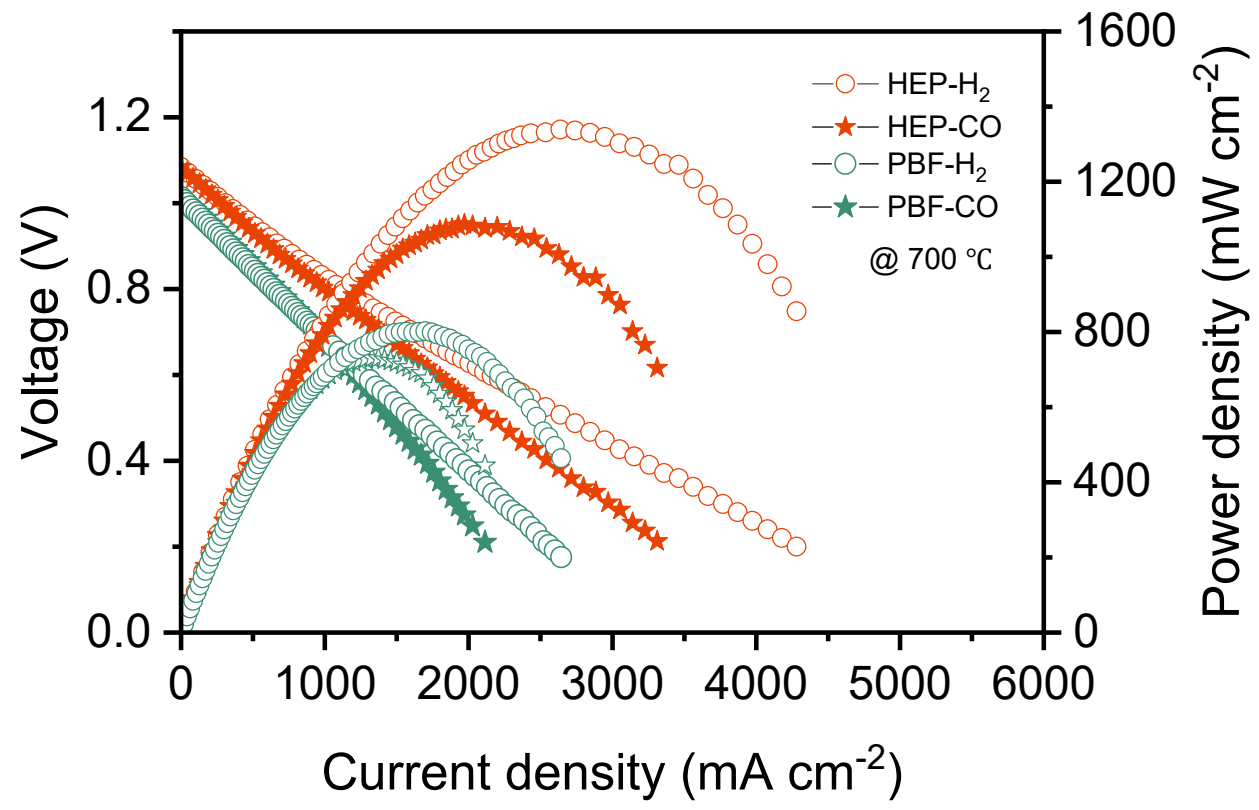

Fig. R35 (also **Fig. S36**) I–V–P curves of single cells using HEP and PBF as the anode under H_2 and CO fuel at 700 °C.

Comment 4-7

(7) Experimental section

It is noted that the GDC barrier layer was co-sintered with the SSZ electrolyte at 1400 °C for 5 hours. However, GDC is known to react with SSZ at temperatures above 1250 °C. Additionally, the electrode area of 0.07 cm^2 is relatively small and may lead to overestimated performance. Fabrication and testing of cells with larger electrode areas (e.g., 0.5–1 cm^2) are advised.

Response to 4-7:

We thank the reviewer for raising this valid concern regarding the potential reaction between the GDC barrier layer and the SSZ electrolyte at the co-sintering temperature of 1400 °C. We acknowledge that inter-diffusion can occur at this temperature. Our experimental investigations align with the reviewer's point. Energy-dispersive X-ray spectroscopy (EDS) line scan analysis across the SSZ/GDC interface (now provided in the new **Fig. R36**) confirms the mutual inter-diffusion of Zr and Ce elements, leading to the formation of a $(Zr, Ce)O_2$ solid solution interlayer at the interface.

Critically, literature suggests that this interlayer is not necessarily detrimental. Studies indicate that a thin, continuous $(Zr, Ce)O_2$ solid solution can enhance the electrochemical activity and compatibility of the interface, improving interfacial adhesion and potentially reducing polarization resistance compared to a sharp, reactive interface (e.g., *Adv. Sci. 2025, e18091*). In our study, the formed interlayer appears dense and well-bonded, contributing to the stable cell performance observed during long-term testing. We have included a brief discussion on this point in the revised supplementary information (Page 36) to provide a more complete interpretation of our fabrication process.

We sincerely apologize for the typographical error in the manuscript regarding the electrode area. The reviewer is correct to highlight the importance of this parameter. The actual active electrode area used for all electrochemical testing was 0.7 cm^2, not 0.07 cm^2 as mistakenly written. This has been corrected throughout the revised manuscript (Methods section and relevant figure captions).

To substantiate this correction, we have included a photograph of a typical fabricated single cell with the electrode area clearly delineated and labeled (**Fig. R20**). We agree that testing with larger areas (e.g., 0.5-1 cm^2) is valuable for future scale-up studies. For the present fundamental study focused on material properties and mechanism exploration, the 0.7 cm^2 area provides reliable and reproducible data for comparative performance analysis.

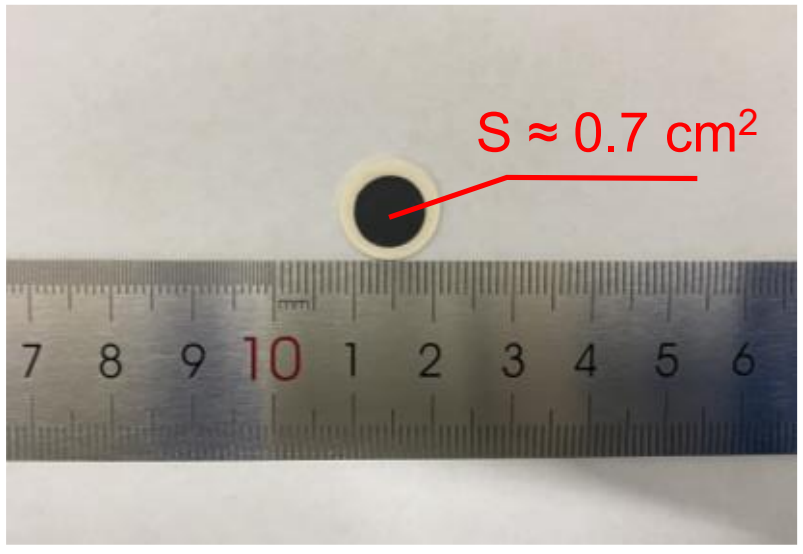

Fig. R20 The photograph of single cell.

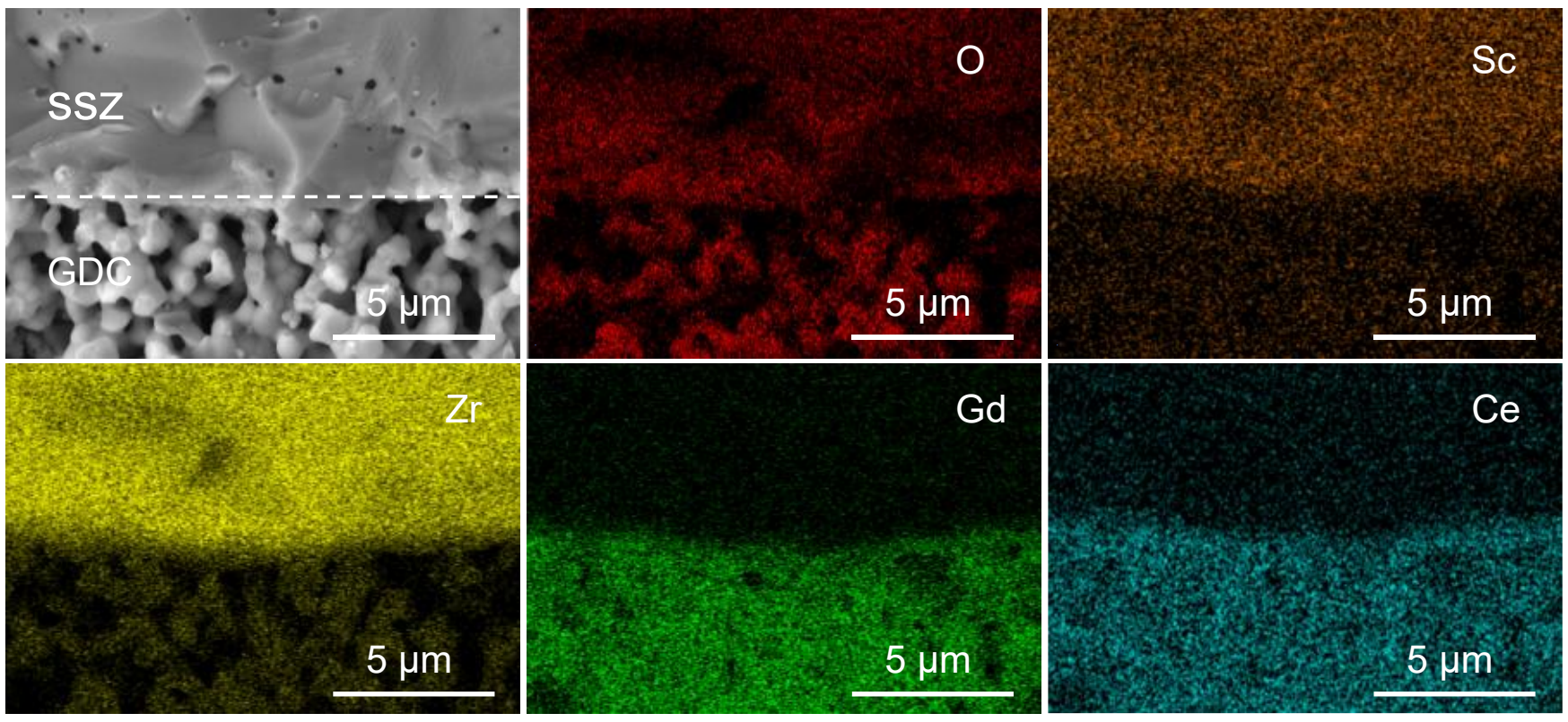

Fig. R36 (also **Fig. S28**) EDS mapping images of SSZ|GDC interface.

Related discussions have been added in the revised manuscript as follows:

Line 343-344: A thin GDC interlayer was applied between the cathode/anode and the electrolyte to prevent phase reactions (Fig. S28).

Supplementary information page 36: EDS results confirm the diffusion of Zr and Ce elements at the interface, which induces the formation of a (Zr, Ce)O_2 solid solution interlayer between SSZ and GDC. Notably, relevant literature[27] indicates that such an interlayer is not necessarily detrimental to cell performance; instead, a thin, continuous (Zr, Ce)O_2 solid solution interlayer can enhance electrolyte-electrode electrochemical activity, interfacial compatibility

and adhesion, while potentially reducing polarization resistance compared to a sharp, reactive interface. In this study, the formed (Zr, Ce)O_2 interlayer is dense and well-bonded, contributing to the stable electrochemical performance of the cell during long-term testing.

Comment 4-8

(8) Language and Labeling

There are instances of mislabeling in the main text (Fig. S4 and S5) and inconsistent abbreviations in the supporting figures (e.g., “HEM” and “PBC”). These should be corrected for consistency and clarity.

Response to 4-8:

We thank the reviewer for their meticulous attention to the details of labeling and nomenclature. We sincerely apologize for the inconsistencies, which have now been thoroughly corrected in the revised manuscript and Supplementary Information to ensure professionalism and clarity. Specifically, we have taken the following actions:

All in-text citations to supplementary figures (including **Fig. S4** and **S5**, as shown in **Fig. R37** and **R38**) have been checked and are now accurate. All abbreviations in the supporting figures and the main text have been unified. For clarity and consistency with the main text: “HEP” has been standardized to “HEP” (High-Entropy Perovskite). “PBC” has been standardized to “PBF” ($PrBaFe_2O_{6-\delta}$). We have performed a complete check of all figures, captions, and the main text to ensure consistency in terminology, abbreviations, and references throughout the entire manuscript.

We believe these corrections have significantly improved the consistency and readability of our work. We are grateful to the reviewer for helping us enhance the quality of our presentation.

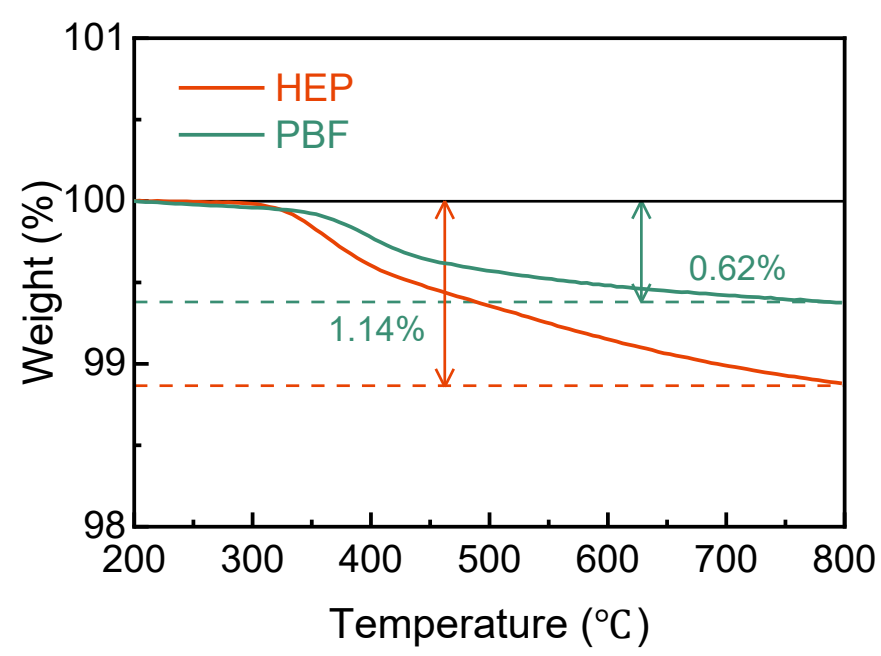

Fig. R37 (also **Fig. S17**) TG patterns of HEP and PBF in the temperature range of 200−800 °C in air.

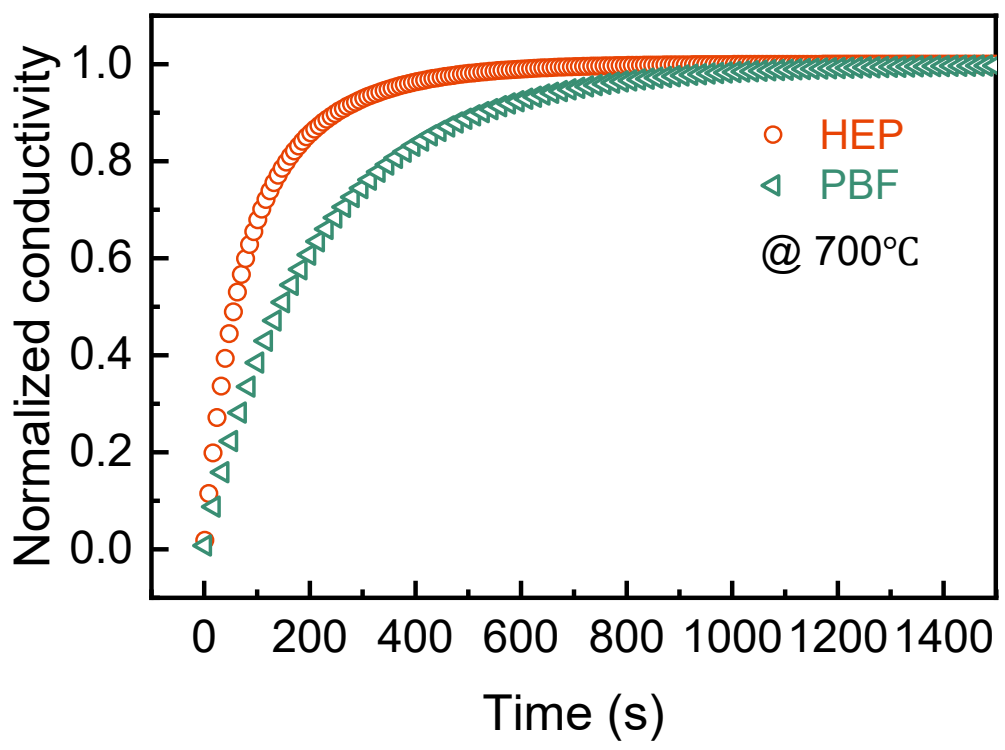

Fig. R38 (also **Fig. S20**) Electrical conductivity relaxation curves of HEP and PBF samples.

Response to Reviewers

Dear Reviewers:

Firstly, we would like to express our sincere gratitude for the considerable time and dedicated efforts that all reviewers have devoted to evaluating our manuscript.

We highly appreciate all the thoughtful, rigorous comments and constructive suggestions provided by the four reviewers, which have substantially improved the scientific rigor, logical completeness, and overall quality of our manuscript. We have carefully and comprehensively revised the manuscript to thoroughly address every comment and concern raised. Our detailed point-to-point responses to each reviewer are presented as follows.

Reviewer #1

It can be accepted in the current form.

Response to Reviewer #1:

We sincerely appreciate the Reviewer #1's positive comments and kind recommendation of this manuscript for publication.

Reviewer #2

Authors have solved all questions I raised. I think it is ready for publication.

Response to Reviewer #2:

We highly appreciate the Reviewers #2' feedback and recommend the journal for acceptance of this paper

Reviewer #3

The revised manuscript shows a clear and significant improvement compared to the original submission, and most of the concerns raised during the previous round of review have been carefully addressed. In particular, the addition of comprehensive impedance spectroscopy data, including DRT analysis under relevant operating conditions, effectively resolves the main issues raised in my previous report regarding electrochemical validation and mechanistic support. At the same time, the authors have responded constructively to the comments of the

other reviewers, incorporating additional control experiments, clarifications, and post-test characterizations that improve the overall clarity and robustness of the work. Overall, the manuscript is now much clearer, more complete, and scientifically stronger.

Response to Reviewer #3:

We very much appreciate the Reviewer #3's valuable comments to improve our manuscript. According to the raised suggestions, our responses to your detailed comments per point are as follows.

Minor Comment 3-1

The weak diffraction feature labeled as "carbon" in the XRD pattern (**Fig. R2**, **Fig S39**) would benefit from a more cautious assignment. While carbon deposition under methane operation is plausible, the identification based on a single low-intensity peak is not fully conclusive. It may be helpful to briefly clarify whether this feature is consistent with graphitic carbon (e.g., position and peak shape), or to acknowledge that other surface species (such as minor carbonate formation) cannot be entirely excluded from XRD alone. A more neutral labeling (e.g., "possible carbonaceous species") or a short clarification in the text would improve the robustness of the interpretation without requiring additional experiments.

Response to 3-1:

We thank the reviewer for this valuable comment and suggestion.

In response to your suggestion, we have updated the original over-simplified label "carbon" to the more neutral and rigorous annotation of "possible carbonaceous species" in the revised figure.

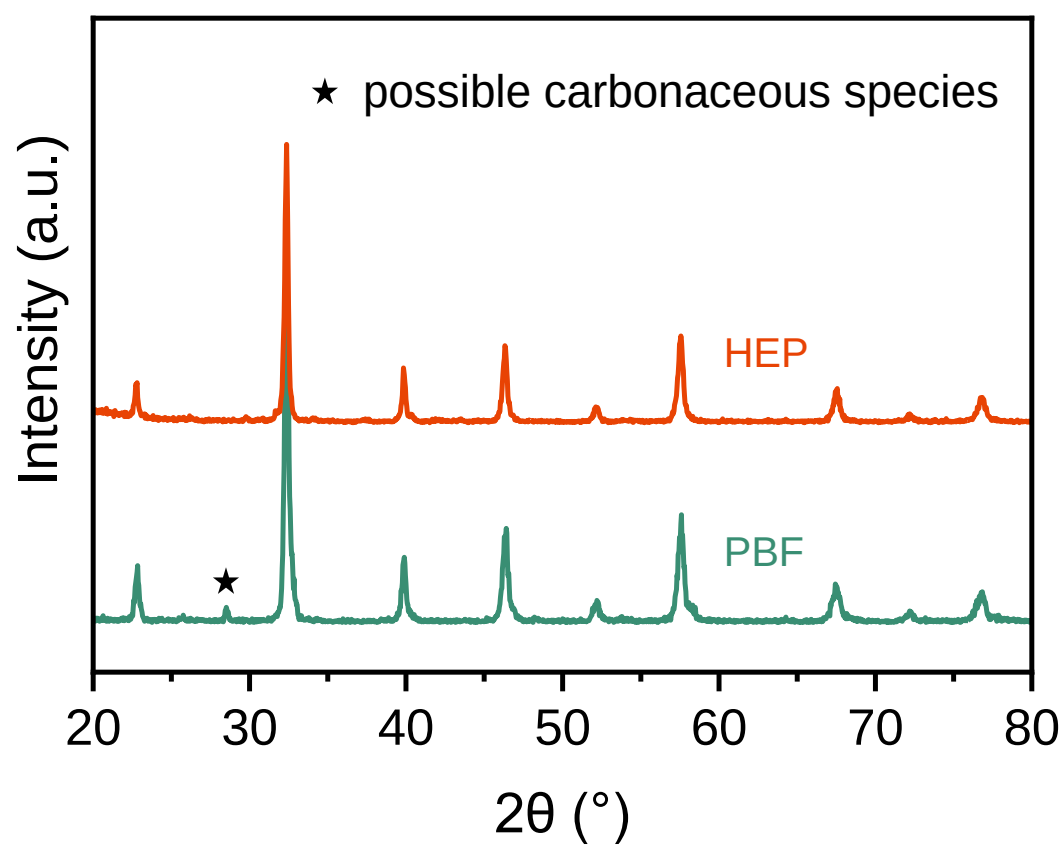

Fig. R2 (also **Fig. S39**) XRD patterns of HEP and PBF anode after long-term stability test.

Minor Comment 3-2

Fig R14 (Fig S24): a) temperature units are missing.

Response to 3-2:

We sincerely appreciate the reviewer for this meticulous reminder regarding the missing temperature unit in **Fig. R14** (**Fig. S24**). We fully acknowledge this oversight in our original figure annotation.

In response to your comment, we have supplemented the complete temperature unit symbol °C for all temperature labels in **Fig. R14**, as shown in the revised figure.

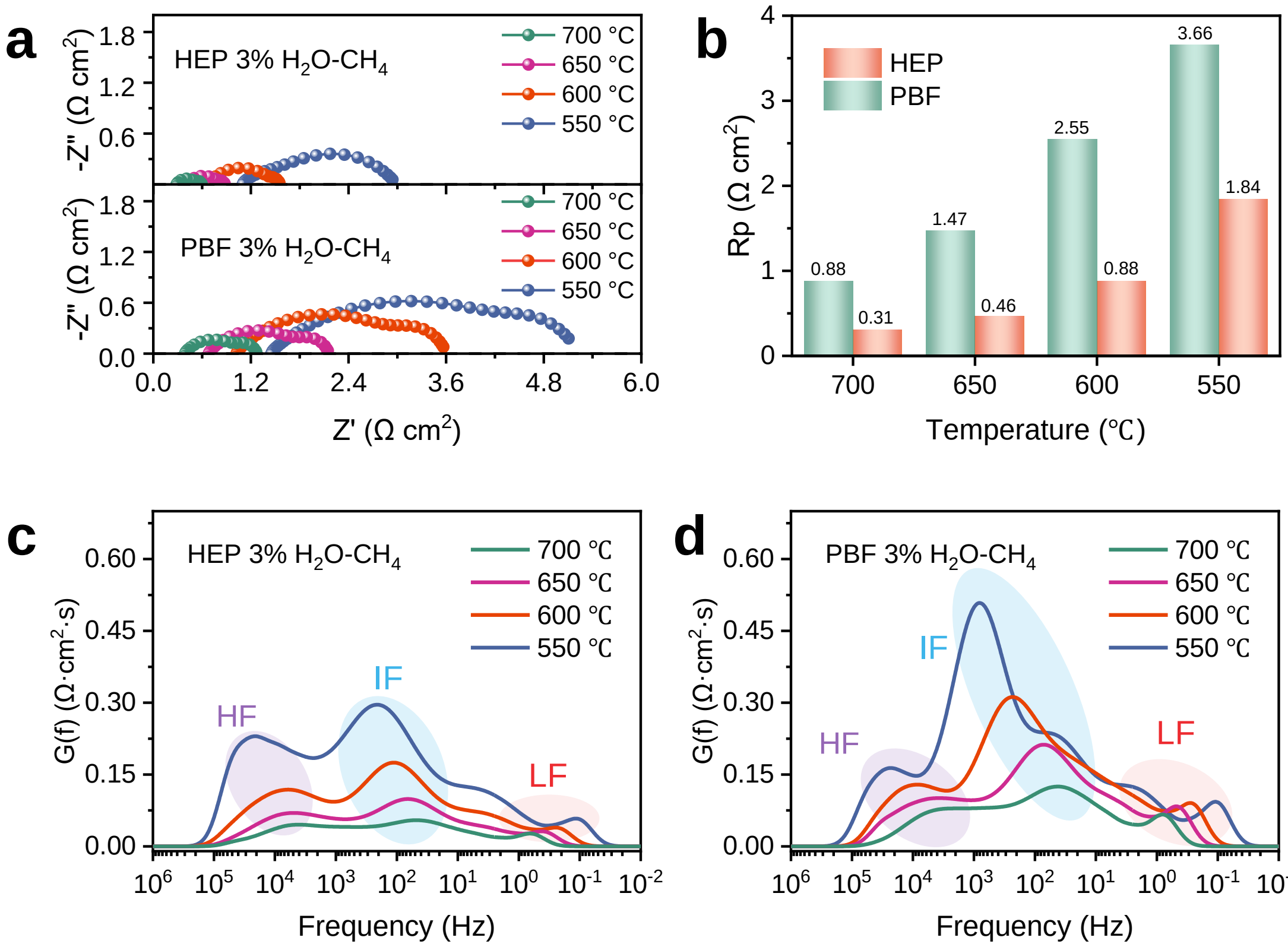

Fig. R14 (also **Fig. S24**) **a** EIS plots of HEP and PBF anodes in 3% H_2O-CH_4. **b** Comparison of EIS using different anodes. DRT fitting results for **c**) HEP and **d**) PBF.

Minor Comment 3-3

Fig R15. A moderate variation in the high-frequency intercept (Rs) across different fuel

atmospheres can be physically reasonable. However, since the electrolyte contribution should remain largely constant at fixed temperature, it would be helpful for the authors to briefly comment on the origin of the observed Rs variations when changing atmospheres.

Response to 3-3:

We sincerely appreciate the reviewer for this insightful comment regarding the variation of the high-frequency intercept (Rs) in **Fig. R15**. We concur that, while the electrolyte contribution should remain relatively constant at a fixed temperature, the observed moderate fluctuation in Rs across different fuel atmospheres is physically reasonable and consistent with general principles of SOFC electrochemistry, as supported by existing literature [Khan, M. Z. et al. *Applied Energy* **407**, 127347 (2026)].

According to recent critical reviews and fundamental EIS theory, the Rs typically reflects the total ohmic resistance of the cell, which may include not only the electrolyte resistance but also potential contributions from gas-phase conduction within the porous anode network, interfacial contact resistances, and subtle changes in the surface charge distribution at the electrode-electrolyte interface. Consequently, at a constant temperature, differences in fuel atmosphere (e.g., H_2, CO, CH_4, humidified CH_4) might induce slight variations in gas properties and interfacial states, which could collectively contribute to the minor Rs variation observed.

We are grateful for the reviewer's professional comment, which helps improve the rigor of our impedance analysis.

Minor Comment 3-4

In Fig. R15c, the x-axis title appears to contain a typo and should not read "Temperature".

Response to 3-4:

We sincerely appreciate the reviewer for this meticulous correction regarding the inappropriate x-axis title in **Fig. R15c**. We fully acknowledge the original typo and logical error, as the x-axis categorizes different fuel atmospheres rather than temperature variables.

In response to your comment, we have revised the original incorrect x-axis label Temperature (°C) to the accurate and rigorous title Fuel atmosphere. All revisions have been fully completed in the revised manuscript.

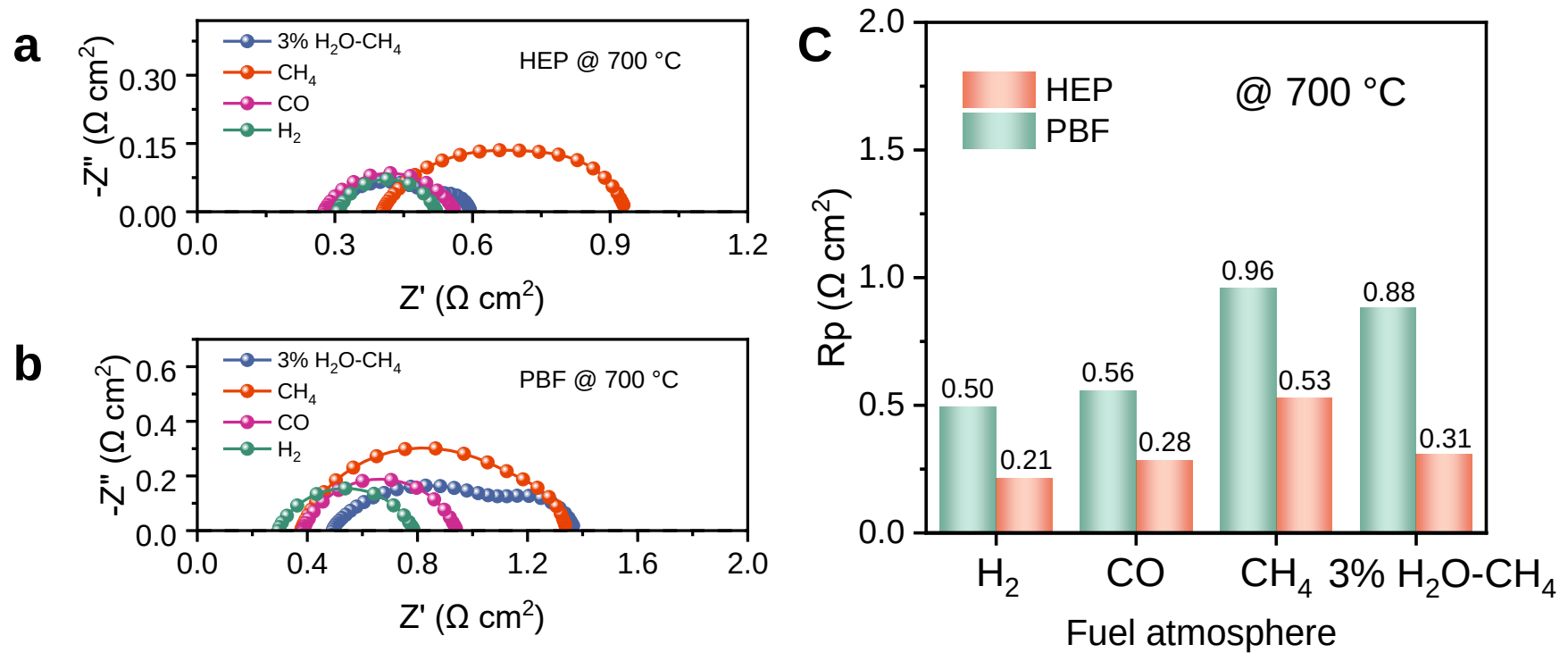

Fig. R15 EIS plots of (**a**) HEP and (**b**) PBF anodes under different atmosphere at 700 °C. **c** Comparison of EIS under different atmosphere at 700 °C for HEP and PBF.

Reviewer #4

The revised manuscript is suggested to be published.

Response to Reviewer #4:

We sincerely appreciate Reviewer #4 for the positive recommendation of our revised manuscript. Thank you very much for your recognition and support.
